# Analysis of head and neck carcinoma progression reveals novel and relevant stage-specific changes associated with immortalisation and malignancy

**DOI:** 10.1038/s41598-019-48229-7

**Published:** 2019-08-19

**Authors:** Ratna Veeramachaneni, Thomas Walker, Timothée Revil, Antoine De Weck, Dunarel Badescu, James O’Sullivan, Catherine Higgins, Louise Elliott, Triantafillos Liloglou, Janet M. Risk, Richard Shaw, Lynne Hampson, Ian Hampson, Simon Dearden, Robert Woodwards, Stephen Prime, Keith Hunter, Eric Kenneth Parkinson, Jiannis Ragoussis, Nalin Thakker

**Affiliations:** 1 Faculty of Biology, Medicine and Health, University of Manchester, Manchester, M13 9PL UK; 2 McGill University and Genome Quebec Innovation Centre, McGill University, Montreal, Quebec H3A 0G1 Canada; 3 Wellcome Trust Centre for Human Genetics, University of Oxford, Oxford, OX3 7BN UK; 4 Department of Cellular Pathology, Manchester University NHS Foundation Trust, Manchester, M13 9WL UK; 5 Department of Molecular and Clinical Cancer Medicine, Institute of Translational Medicine, University of Liverpool, Liverpool, L69 3BX UK; 6 Department of Head and Neck Surgery, Aintree University Hospitals NHS Foundation Trust, Liverpool, L9 7AL UK; 7 Precision Medicine and Genomics, IMED Biotech Unit, Astra Zeneca, Cambridge, CB4 OFZ UK; 8 Department of Oral and Maxillofacial Surgery, Pennine Acute NHS Trust, Manchester, M8 5RB UK; 9 Centre for Immunology and Regenerative Medicine, Institute of Dentistry, Barts and the London School of Medicine and Dentistry, Queen Mary University of London, London, E1 4NS UK; 10 School of Clinical Dentistry, University of Sheffield, Sheffield, S10 2TA UK; 11 Present Address: Novartis Institute for BioMedical Research, Basel, Switzerland

**Keywords:** Cancer genomics, Cancer genomics

## Abstract

We report changes in the genomic landscape in the development of head and neck squamous cell carcinomas HNSCC from potentially premalignant lesions (PPOLS) to malignancy and lymph node metastases. Likely pathological mutations predominantly involved a relatively small set of genes reported previously (*TP53*, *KMT2D*, *CDKN2A*, *PIK3CA*, *NOTCH1 and FAT1*) but also other predicted cancer drivers (*MGA*, *PABPC3*, *NR4A2*, *NCOR1* and *MACF1*). Notably, all these mutations arise early and are present in PPOLs. The most frequent genetic changes, which follow acquisition of immortality and loss of senescence, are of consistent somatic copy number alterations (SCNAs) involving chromosomal regions enriched for genes in known and previously unreported cancer-related pathways. We mapped the evolution of SCNAs in HNSCC progression. One of the earliest SCNAs involved deletions of *CSMD1* (8p23.2). *CSMD1* deletions or promoter hypermethylation were present in all of the immortal PPOLs and occurred at high frequency in the immortal HNSCC cell lines. Modulation of *CSMD1* in cell lines revealed significant suppression of proliferation and invasion by forced expression, and significant stimulation of invasion by knockdown of expression. Known cancer drivers *NOTCH1*, *PPP6C*, *RAC1*, *EIF4G1*, *PIK3CA* showed significant increase in frequency of SCNA in transition from PPOLs to HNSCC that correlated with their expression. In the later stages of progression, HNSCC with and without nodal metastases showed some clear differences including high copy number gains of *CCND1*, hsa-miR-548k and *TP63* in the metastases group.

## Introduction

Globally, head and neck carcinomas account for over 550,000 new cases per annum with a mortality of approximately 275,000 cases per year^1^. By far, the commonest site of cancer within this region is the oral cavity and the commonest type of tumour is squamous cell carcinoma (SCC), which accounts for over 90% of all malignant tumours at this site. HNSCC is associated with high mortality having an overall 5-year survival rate of less than 50%. Furthermore, both the disease and the multimodal treatments options involved are associated with high morbidity^2^.

The molecular pathology of head and neck squamous carcinoma has been extensively studied previously^3^ and the common somatic genetic changes have been characterised^4–8^. There have been some studies of the multistage evolution of these tumours^9^ but this is less well characterised. A small number of tumours arise from pre-existing lesions (known as potentially malignant lesions or PPOLs) such as leukoplakia or erythroplakia, which display variable epithelial dysplasia^10^. However, the vast majority are thought to arise *de novo* from macroscopically normal appearing mucosa or possibly undiagnosed PPOLs. Support for the latter comes from data showing that the transcriptional signatures of PPOLs are retained in unrelated samples of SCC both *in vivo*^11^ and *in vitro*^12^. Nevertheless, it is clear that tumours arise from within a wide field bearing the relevant genetic alterations and that there is a risk of synchronous or metachronous tumours^13,14^. A fuller understanding of the events in evolution of these cancers may permit the development of biomarkers or effective therapeutic interventions possibly targeting not just tumours but also the early changes in the field. The first multi-step model proposed for carcinogenesis in HNSCC^15^ suggested typical alterations associated with progression from normal mucosa to invasive carcinoma, with dysplasia reflecting an earlier stage of cancer progression.

We, and others, have previously shown that both SCCs and PPOLs yield either mortal and immortal cells *in vitro*^16,17^ and sometimes, mixtures of the two^16–18^. The status of these mortal cells is unclear. Unlike the immortal cells, they lack inactivation of *TP53* and *CDKNA*, but our limited previous investigations show that they are genetically stable. Nevertheless, they often have extended replicative lifespans^16,17^, possess neoplastic phenotypes, such as resistance to suspension-induced terminal differentiation^16^ and have expression signatures which are distinct from both immortal cells and normal cells^12^. Furthermore, these characteristics are present in mortal cells from both PPOLs and SCCs^12^ suggesting the presence of distinct pathways for the development of mortal and immortal SCC. Our preliminary work established that the mortal PPOL were cytogenetically diploid and had low levels of LOH^16^ but the immortal PPOLs have never been subjected to extensive genomic analysis. In addition, whilst extensive genetic analysis of HNSCC has been carried out in recent years^4–6,8^, including the identification of key driver mutations, the stages in the cancer progression, at which they occur and the resulting phenotypes are still unknown. There are numerous previous studies of PPOLs limited to determining the frequency of alterations in specific gene or specific genetic regions. Exceptions to this are the study by Bhattacharya and colleagues^9^ and Wood and colleagues^19^, which reported a comprehensive analysis of copy number variation in primary PPOLs and HNSCC although in the later study, the PPOL analyses was confined to metachronous lesions. A very recent study reported the status of SCNAs and common driver mutations in several keratinocyte cultures derived from metachronous normal and dysplastic lesions characterised for replicative lifespan^20^. Here we extend their findings by a combination of exome/targeted sequencing, methylation and SNP/CGH array analyses, using our unique panel of fully characterised mortal cultures and immortal cell lines derived from both PPOLS and HNSCC, to show that these genetic alterations are mostly associated with cellular immortalisation and increase with the stage of tumour progression in this class of SCC keratinocyte. The principal focus of this study is to characterise in detail changes in the evolution and progression of HNSCC rather than focussing on HNSCC as a group which have already been well characterised with respect to mutation and genomic landscapes. In doing so we identify common alterations of the candidate tumour suppressor gene *CSMD1* not highlighted previously and provide preliminary evidence of its function. Furthermore, alterations of *CSMD1* were common in PPOL cultures and therefore may be early events in SCC development.

## Results

### Genes target for mutations in HNSCC are altered early in cancer development

Several recent genomic sequencing studies have fully characterised mutations in HNSCC^4–7^. In order to map these previously identified mutations to progression of HNSCC, a small previously well-characterised panel of samples consisting of 3 PPOL mortal cultures, 7 PPOL cell lines (from lesions known to have progressed as well as lesions that had not progressed) 1 mortal culture derived from HNSCC and 11 HNSCC cell lines^17,21,22^ were selected for exome-sequencing or targeted sequencing of the top 40 genes identified as altered in these cancers^4^ using HaloPlex Target Enrichment System (Agilent, Santa Clara, CA, USA). The sample details are given in Supplementary Data S1.

For exome sequencing, approximately 6 gigabases of sequence mapped to the human genome with an average of 65.7% (Range 33.8% to 86.1%) of the targeted exome covered at twenty-fold or higher (shown in Supplementary Data S2). For HaloPlex sequencing, approximately 800 megabases of sequence mapped to the human genome with an average of 94.8% of the targeted exons covered at twenty-fold or higher (shown in Supplementary Data S3).

**Figure 1:**
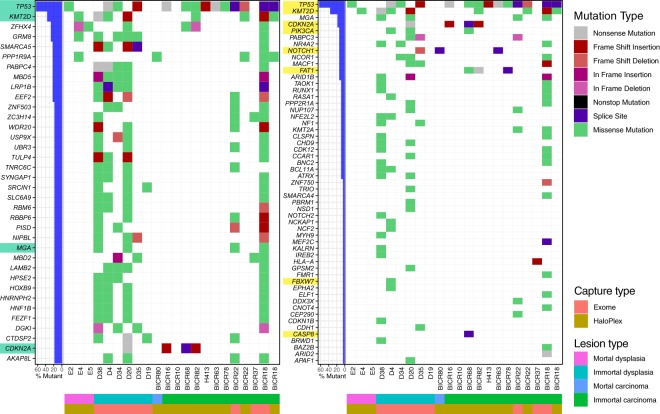
Mutation landscape plot of filtered variants present from exome and HaloPlex captures. On the left, the genes shown contain a distinct mutation in at least three samples, while for the plot on the right, the genes shown are the genes known to be HNSCC drivers according to IntOGen. The bar plot on the left of both mutational landscape plot shows the percentage of samples that contain a variant in the specified genes, with HNSCC drivers highlighted in green (left) and genes present in the HaloPlex capture highlighted in yellow (right). The waterfall plot illustrates the single most damaging variant found per gene and per sample, with colors indicating mutation types. The order of the mutation type legend shows the priority of the mutation selected when a gene contains multiple variants. M-PPOL: mortal potentially premalignant lesion cultures; IM-PPOL: immortal PPOL cell lines (progressive: P and non-progressive: NP); HNSCC: Head and neck squamous-cell carcinoma cultures (mortal: M and immortal: IM).

For calling and filtering pathological mutations, we used the strategy described in Material and Methods. The filtering of the sequence variants is shown in Supplementary Data 4. The filtering was stringent and therefore, it is possible that some genuine pathological mutations may have been excluded. However, the full dataset is available through Dryad Digitial Repository (10.5061/dryad.314k5k5).

Given the small numbers of samples examined in our study, we further targeted our analyses to cancer drivers identified by IntOGen (Release 2014.12) The HaloPlex sequencing panel included 8 of the 10 most frequently mutated HNSCC driver genes. Limiting analyses to known cancer drivers also effectively excluded possible false positives that can arise due to DNA replication timing and low transcriptional activity^23^. Thus, our significant driver mutations (Fig. 1) mirror those identified by Lawrence and colleagues, 2013^23^ following correction for these factors. The mutational landscape of PPOL and HNSCC is shown schematically in Fig. 1. Full list of variants is provided in Supplementary Data S5.

Mutations were rare in mortal cultures (Fig. 1). One missense variant each of *TP53* and *KMT2D* were observed in 2 PPOL cultures and one high impact *NOTCH1* mutation was observed in HNSCC culture BICR80.

As with previous studies^4–7^, the mutation analyses revealed a small set of genes (*TP53*, *KMT2D CDKN2A*, *PIK3CA*, *NOTCH1*, and *FAT1*) as the most common targets for likely deleterious sequence mutations in immortal PPOL and HNSCC cell lines (Fig. 1). Mutation of *TP53* and *CDKN2A* as an early event in head and neck carcinogenesis is well established^17,24–26^. In the present study, however, we demonstrate for the first time that mutations of *KMTD2*, *PIK3CA* and *NOTCH1* also occur early. Furthermore, except for *NOTCH1*, the mutations are not only present in progressing lesions (D19, D20, D35) but also in non-progressing lesions (D4, D34, D38). Relatively frequent mutations were also detected in several other IntOgen-defined cancer drivers such as *MGA*, *PABPC3*, *NR4A2*, *NCOR1* and *MACF1* (Fig. 1); of these *PABPC3*, *NR4A2*, *NCOR1* and *MACF1* are detected as mutational cancer drivers in head and neck cancer in IntOgen.

### Somatic copy number changes in evolution of HNSCC

Somatic copy number alterations in 7 PPOL cell lines, 11 mortal cell cultures derived from PPOL and 28 HNSCC cell lines (detailed in Supplementary Datas S1 and S6) were derived using array based technologies. The full dataset of Nexus Copy Number v5.1 (BioDiscovery, Inc., CA, USA) data are available at Dryad Digital Repository (10.5061/dryad.314k5k5).

#### Mortal keratinocyte cultures derived from PPOL are genetically stable

**Figure 2:**
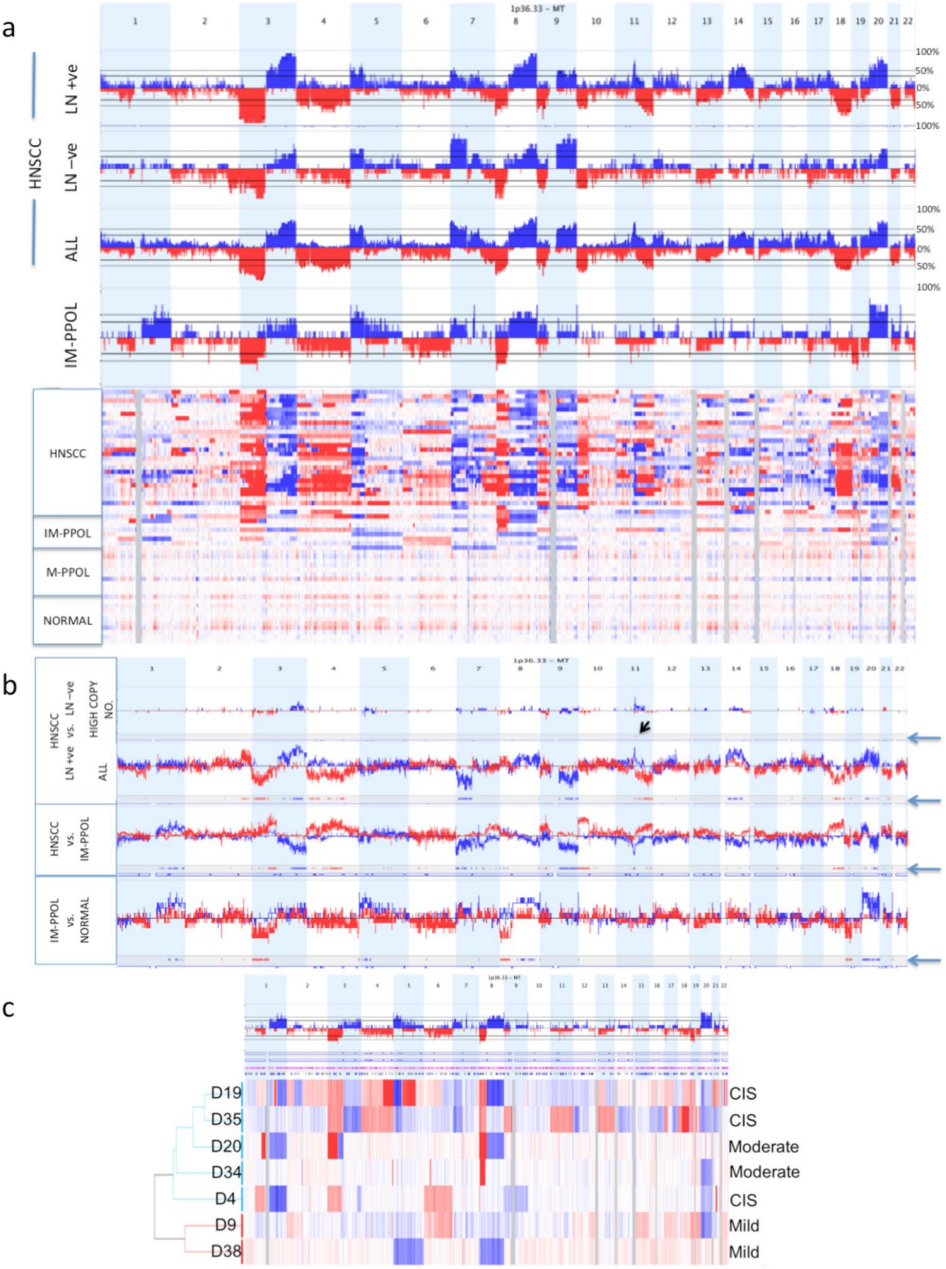
Somatic copy number changes in evolution of HNSCC. (**a**) Density and frequency plots showing copy number gains (blue) and losses (red) in normal fibroblasts, mortal PPOLs (M-PPOL), immortal PPOLs (IM-PPOL) and HNSCC cell lines with and without lymph node metastases (LN + ve and LN − ve respectively). (**b)** Subtraction frequency plots showing differences in copy number gains (blue) and losses (red) between (i) normal fibroblasts, and mortal PPOLs (M-PPOL), (ii) immortal PPOLs (IM-PPOL) and all HNSCC cell lines and (iii) HNSCC cell lines with and without lymph node metastases (LN + ve and LN − ve respectively). Both high and all copy number changes are shown for LN + ve and LN − ve HNSCC cell lines. Blue arrows on the right indicate lanes with regions of difference showing statistical significance (p < 0.05). (**c)** Density and frequency plots showing copy number gains (blue) and losses (red) in individual immortal PPOL cell lines showing hierarchical clustering and grade of dysplasia (CIS, carcinoma in-situ).

Mortal cultures derived from PPOL were genetically stable, showed very few copy number changes and no significant differences compared to matched fibroblasts. These data that are consistent with their diploid chromosome complement and previous limited loss of heterozygosity analysis^16^ (Fig. 2a and Supplementary Data S7).

#### Progressive somatic copy number changes follow immortalisation and loss of senescence in evolution of HNSCC

Our previous studies show that all immortal PPOL cell lines show inactivation of *TP53* and *CDKNA*^16–18^ that confer immortality and loss of senescence. In this study, we show that these cell lines showed in addition, consistent focal and arm-wide SCNAs that define some of the earliest changes in the development of HNSCC that follow immortalisation and loss of senescence (Fig. 2a). Overall, consistent with previous studies^9,19^, compared to normal fibroblasts, there were significant (P < 0.05) losses on chromosome 3p, 8p and 9p and gain of chromosome 20 (Fig. 2b). However, there was clear heterogeneity in the nature of the changes observed in the 7 immortal PPOL samples (Fig. 2c) with some specimens showing more frequent changes than others. This enabled us to further delineate the changes in the earliest stages of the HNSCC development.

Meaningful testing for significant differences between subsets of PPOLS was not possible due to the small sample size and thus, hierarchical clustering was used to group samples by SCNAs (Fig. 2c). The samples clustered into two main groups that suggested a correlation of genomic changes with grade of dysplasia with higher-grade dysplasias showing more changes than lower-grade dysplasias (Fig. 2c). Furthermore, three cell lines (D19, D20, D35) from PPOLs that progressed to carcinomas (progressive PPOLs or P-PPOL), clustered in a discrete subgroup distinct from cell lines (D34, D4) derived from non-progressive lesions (N-PPOL). This was remarkably similar to the clustering previously observed for these cell lines by gene expression profiles^12^ indicating these signatures may at least in part reflect the underlying somatic copy number changes.

P-PPOLs were characterised by consistent arm-level losses of chromosome arms 3p and 8p with homozygous deletions of *FHIT* (3p14.1) and *CSMD1* (8p23.2) (shown in Supplementary Data S8). In addition, two of the three P-PPOLs (D19, D35) showed further arm-level SCNAs on several other chromosomes (+3q, +5p, +7p +8q, −13p, −13q, −18p −18q, +20, a pattern similar to HNSCC cell lines indicating a more advanced stage in progression.

The pattern of SCNAs in cell lines derived from lesions that had not progressed to date (NP-PPOLs, D34, D4, D9 and D38) reflected their earlier stage of evolution (Fig. 2c). These cells harboured focal SCNAs involving the *CSMD1* (3 of 4 cell lines) and *FHIT* (2 of 4 cell lines) on chromosome 8p23.2 and 3p14.1 respectively, and showed arm-level gains of chromosome 20 (3 of 4 cell lines) together with variable and largely focal SCNA of several other chromosomal arms.

Progression to HNSCC was characterised by two main features: (i) an increased frequency of SCNAs and/or extension of SCNA regions involved in immortal PPOLs and (ii) involvement of further regions not involved in PPOLs (Fig. 2). Thus, there were statistically significant increases in frequency of losses of proximal part of Chr3p, and Chr18q and gains of Chr20q. In addition to this, there were SCNAs involving new regions including loss of Chr4q and Chr10p together with gains of Chr5p, Chr9q, Chr14q and Chr11q. The overall changes in HNSCC cell lines were similar to those described for HPV–ve tumours in previous studies^3–9^.

Some genes (e.g., *CDKN2A*, *CDKN2B*) that showed homozygous deletions in PPOLs, showed an increase in frequency of the same in HNSCCs. There were in addition, homozygous deletions of further genes such as *PTPRD*, *LRP1B* and *LINGO2*, which showed frequent hemizygous deletions in the PPOL cell lines suggesting further selection in progression to HNSCC. High copy number gains, largely absent in PPOLs, were more frequent and principally centred on chromosome 11q.

The aggregate HNSCC cell line data concealed differences between cell lines from tumours with and without lymph nodal metastases (LN + ve and LN − ve respectively). Lymph node metastases status of 17 of 28 HNSCC lines was known (10 LN + ve; 7 LN − ve). The LN + ve cell lines showed higher frequency of high copy (>2) number gains of a 1.76 Mb region at chromosome 11q13.2-q13.3 (p < 0.05) that encompasses 10 genes including *CCND1* and the microRNA hsa-miR-548k (Fig. 2a). Similarly, a higher frequency of high copy number gains on two regions on chromosome arm 3q was observed involving *NAALADL2* (chromosome band 3q26.32), and *TP63* and *CLDN1* (chromosome band 3q28). Hierarchical clustering of all HNSCC cell lines by high-copy number aberrations also revealed two major groupings defined by the presence or absence of the high-copy number amplicon on chromosome band 11q13.2-q13.4 (p < 0.01, q bound <0.1) (shown in Supplementary Data S9). In the group lacking the amplicon, only 2 of 8 cell lines with known nodal staging, were derived from LN + ve cancers and each of these involved a single node less than 2 cm (TNM stage N1). By contrast, in the group with the amplicon, 7 of 9 cell lines were derived from LN + ve cancers and 4 of the 7 tumours were graded as TNM stage N2 or higher. These results are consistent with the recent findings linking the 11q13.3 amplicon with poor survival in HNSCC patients^27^.

We used several approaches to identify genes that are potential or likely target of the SCNAs and these are detailed in Supplementary Datas 10–12. These approaches identified genes already known to be implicated in HNSCC as well promising candidates that merit further investigation.

### *FAT1 and CSMD1* are early targets in HNSCC

In the PPOLs, the most common target genes for SCNA included the *FHIT*, *CSMD1*, *CDKN2A* and *CDKN2B* as described above and these are observed in both progressing and non-progressing lines defining them as earliest defined changes along with inactivation of *TP53*. In addition, we identified less frequent homozygous and hemizygous deletions of *FAT1* (14% and 43% respectively, Supplementary Data S12). Although, loss-of-function mutations of *FAT1* in HNSCC are reported in this study as well as previous studies^4–6,8^, identification of homozygous deletions of *FAT1* in PPOLs establish inactivation of *FAT1* as an additional early change in HNSCC development.

The functional roles in carcinogenesis of genes that are target for these early changes, are generally well characterised except for *CSMD1*. Thus, we explored whether *CSMD1* functions as a tumour suppressor in HNSCC. *CSMD1* is deleted in many tumour types^28^ and also shows rare somatic mutations^29^. We observed both homozygous and hemizygous deletions of *CSMD1* (5/28 and 21/28 respectively) in HNSCC cell lines (shown in Supplementary Data S13).

**Figure 3:**
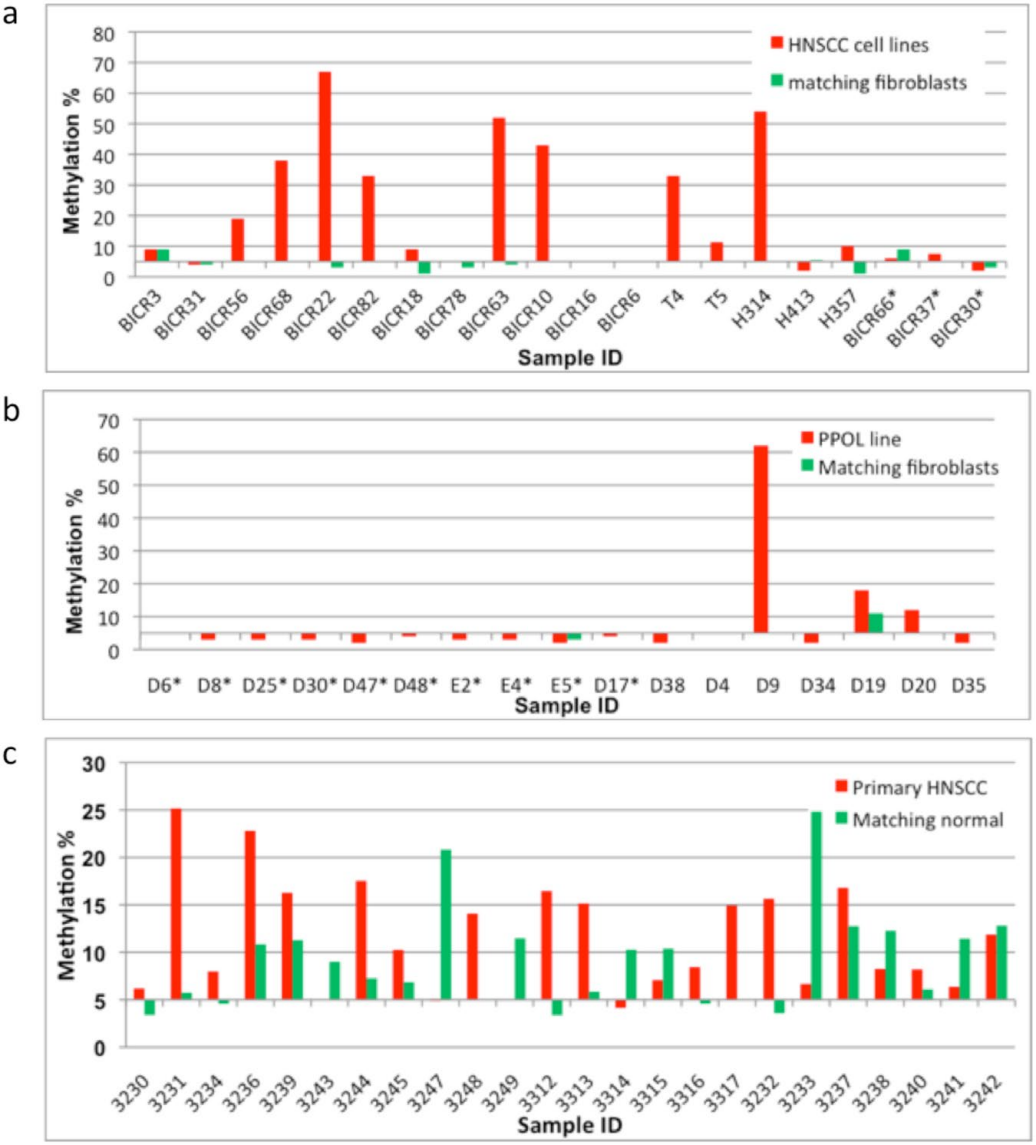
Clustered column chart representing pyrosequencing analyses of the *CSMD1* promoter region. Sample ID is shown on the X-axis and mean methylation percentage is represented on the Y-axis. For each sample, a mean methylation percentage greater than 5% was considered as significant promoter methylation. **(a)** Mortal and immortal HNSCC cell lines. Significant promoter methylation was observed in12 of 17 (~70%) immortal HNSCC cell lines but not in any of the mortal HNSCC cell lines (indicated by *). The highest levels of promoter methylation were in cell lines with hemizygous deletions (BICR56, BICR22, BICR82, BICR10 and T4) or no deletions (BICR63, BICR68 and H314). **(b)** PPOL cell lines/cultures. Significant promoter methylation was observed 3 of 7 (~43%) immortal PPOL lines but not in any of the mortal PPOL cultures (indicated by *). The highest level of promoter methylation was in a single immortal cell line (D9) that had no deletions at the *CSMD1* locus. (**c**) Primary HNSCCs and matching normal tissues. Significant promoter methylation was observed in 15 of 24 (approximately 63%) cases.

Only one immortal PPOL (D38) had sustained a potentially deleterious missense mutation (R3193K) in *CSMD1* and no likely pathogenic mutations were observed in HNSCC. *CSMD1* promoter methylation analyses by pyrosequencing revealed hypermethylation in 9 of 12 (75%) HNSCC cell lines with matching normal samples (and several other HNSCC cell lines without matching normal tissues) (Fig. 3a), 3 of 7 (~43%) PPOL cell lines (Fig. 3b), and 15 of 24 (~63%) primary HNSCCs (Fig. 3c). The highest level of promoter hypermethylation was seen in immortal HNSCC cell lines with either hemizygous deletions (BICR56, BICR22, BICR82, BICR10 and T4) or absence of deletions (BICR63, BICR68 and H314) (detailed in Supplementary Data S13), and in the only immortal PPOL cell line (D9) that lacked *CSMD1* deletions (Fig. 3, and Supplementary Data S8). Additionally, the frequency of promoter methylation in primary tumours (~63%) was not too dissimilar to the frequency of hemizygous deletions (~75%) in our HNSCC cell lines. These findings suggest that frequent inactivation of *CSMD1* in HNSCC occurs by deletion and/or promoter hypermethylation.

**Figure 4:**
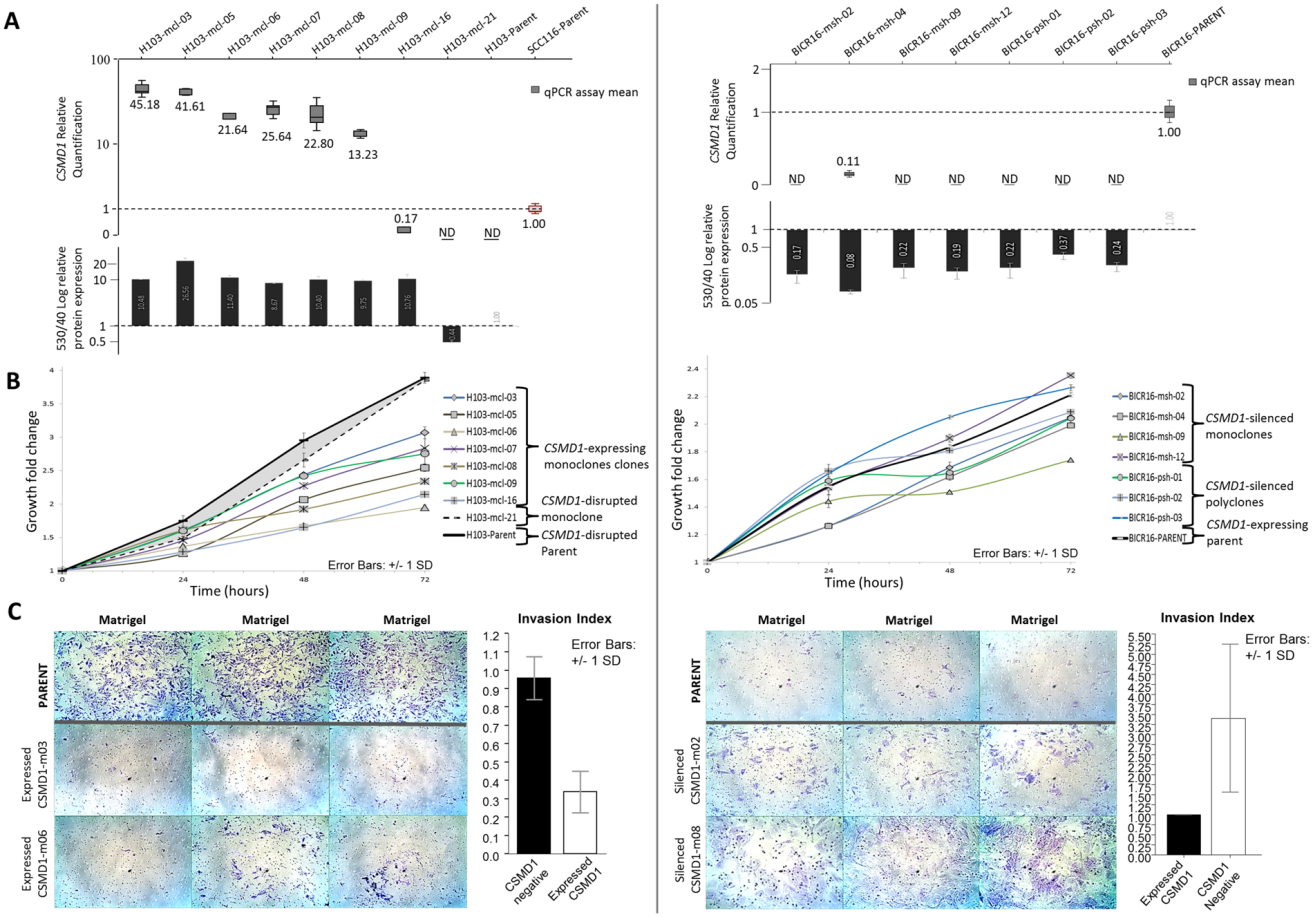
Phenotypic effects of *CSMD1* expression modulation in HNSCC. Left panel: forced *CSMD1* expression in the *CSMD1* non-expressing H103 cell line. Right panel: silencing of *CSMD1* in the *CSMD1*-expressing BICR16 cell line. (**A)** *CSMD1* mRNA transcript quantification by RT-qPCR and protein quantification by flow cytometry for generated clones, presented as fold change normalised to the reference cell line. Left: H103 *CSMD1*-expressing clones and H103 *CSMD1*-negative parent cells and *CSMD1*-disrupted clone H103-mcl-21, normalised to SCC116 *CSMD1* expressing cells (red outline). Right: BICR16 *CSMD1*-silenced clones normalised to *CSMD1*-expressing BICR16 parent cell line. Boxplots represent RQ normalised to reference cells. Each box plot is the relative quantification (RQ) of two plates each of triplicate target and reference gene CT values plus and minus log-transformed standard deviations and so incorporates intra-plate variance. Standard boxes depict the first-third quartiles, whiskers depict ±1.5IQR with median values indicated below. Bar charts represent CSMD1 protein fold change normalised to reference cells. Error bars are ±standard deviation. (**B)** Effects of modulation of *CSMD1* expression on cell proliferation. Left: *CSMD1*-expressing H103 monoclones compared to *CSMD1*-negative H103 parent and control cells. *CSMD1* expression resulted in a reduced growth rate compared to *CSMD1*-negative parent and H103-mcl-21 cells (p = 0.0053). Right: Cell proliferation of *CSMD1*-silenced BICR16 clones compared to *CSMD1*-expressing BICR16 parent cells. *CSMD1* silencing did not result in a significant change in growth rate compared to *CSMD1*-expressing parent cells (p = 0.239). Plots represent triplicate points from duplicate 96AQ assays for 96 hours with growth rates normalised to achieve relative fold change values for 0–72 hrs. Error bars are ±1 SD. (**C**) Effects of modulation of *CSMD1* expression on gel invasion. Three trans-well chambers of 2 representative clones and parent cells are displayed (for full dataset see Supplementary Data 14). The invasion index of generated clones vs. parent is depicted as bar charts (white and black bars respectively). Left: *CSMD1*-expressing clones vs. *CSMD1*-negative H103 parent cells. *CSMD1* expression results in a marked decrease of gel invasion (p = 5.975 × 10^−5^). Right: *CSMD1*-silenced clones vs. *CSMD1*-expressing BICR16 parent cells. *CSMD1* silencing results in a marked increase in gel invasion (p = 1.822 × 10^−05^).

Stable transfection of full-length *CSMD1* cDNA into the H103 cell line, which lacks endogenous expression of *CSMD1* expression, resulted in a significant inhibition of proliferation (p = 0.0053) and invasion (p = 5.98 × 10^−5^ - Matrigel *in vitro* assay). Results from a representative clone are shown in Fig. 4 (with data from all clones shown in Fig. S9). *CSMD1* expression was also silenced by stable transfection with an shRNA vector in the BICR16 cell line, which has a very low but detectable level of endogenous *CSMD1* expression. This cell line has complete hemizygous deletion of the *CSMD1* together with several regions of small homozygous deletions (detailed in Supplementary Data S13) although the functional state of the protein is unknown. The stable *CSMD1*-silenced clones showed a more variable effect on both proliferation and invasion; clones displayed a significant increase in invasion compared to parent cells (p = 1.82 × 10^−5^) but loss of *CSMD1* did not have a significant effect on the rate of proliferation (p = 0.239). Data from representative clones are shown in Fig. 4 (with data from all clones show in Supplementary Data S14). It is possible that the inconsistent proliferation results with gene silencing are due to the fact that these cell lines have acquired the necessary cancer traits with some CSMD1 expression and that these traits are not significantly impacted by additional knockdown of residual and possibly hypofunctional CSMD1. Overall, however, these data support a role for *CSMD1* as a tumour suppressor gene inactivated in the very early stages of HNSCC development.

Homozygous deletions of other genes (*NCKAP5*, *SORBS2*, *CCSER1*) not previously implicated in HNSCC were also identified in GISTIC peak regions in PPOLs. *NCKAP5* was excluded as a likely common target by further analyses (detailed in Supplementary Data S15) but the other genes remain to be characterised.

#### Identification of key genes in progression from PPOLs to HNSCC by integrative analyses

**Figure 5:**
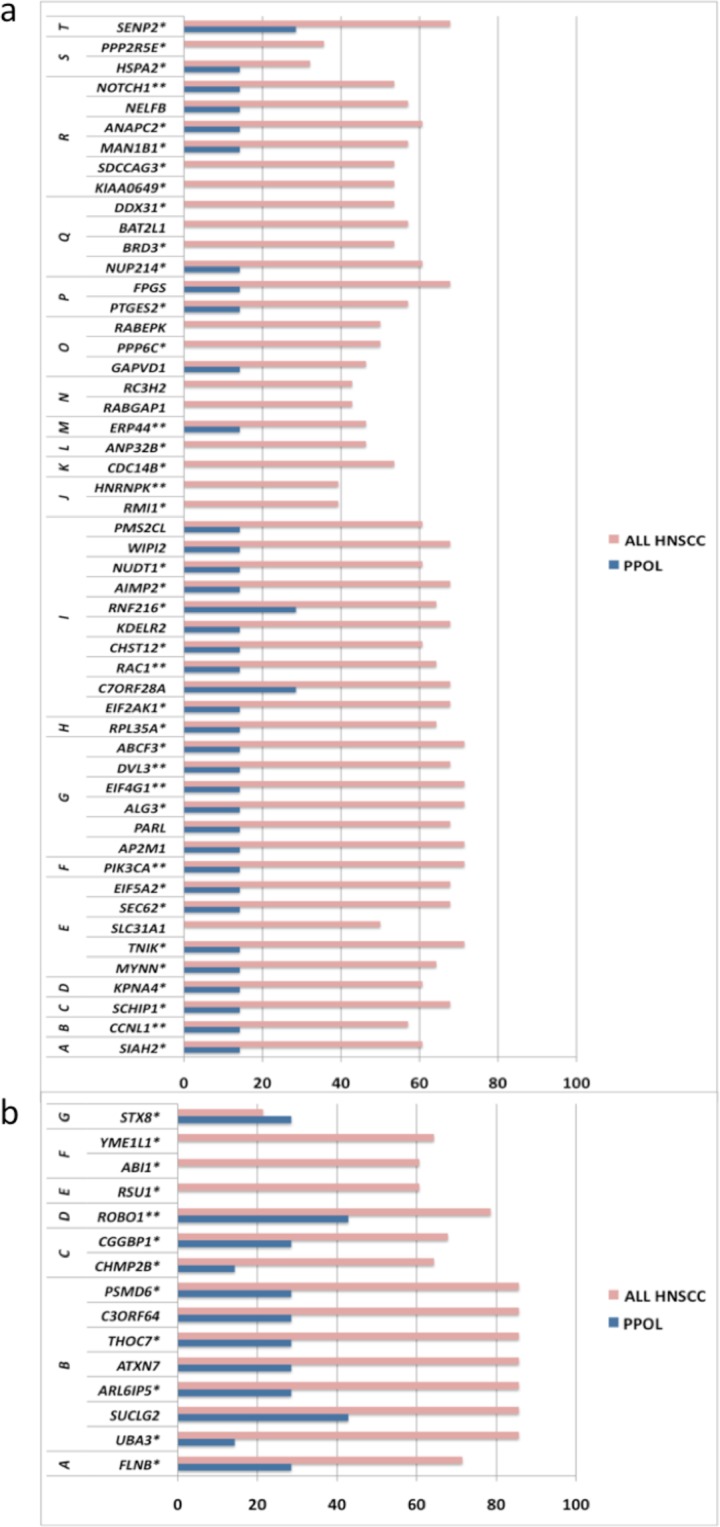
Frequency of SCNA of genes from chromosomal regions that show a significant difference in SCNAs between PPOLs and HNSCC and correlation with expression. (**a**) Copy number gains. (**b**) Copy number losses. Genes reported to be associated with any cancer previously by PUBMED search (‘Gene name, Cancer’). **Genes reported to be associated with HNSCC previously by PUBMED search (‘Gene name, HNSCC’, oral cancer). Chromosomal regions showing differences between PPOLS and HNSCC are indicated by letters on the left; in top panel (a), the regions are: A, chr3:151842842–152984767; B, chr3:157160169–158933894; C, chr3:160599782–161161335; D, chr3:161705726–162566403; E, chr3:170691970–173911476; F, chr3:177707736–180451354; G, chr3:185035692–185529164; H, chr3:198578155–199298372; I, chr7:1631815–7317208; J, chr9:85367221–85868737; K, chr9:97596715–98774190; L, chr9:99427940–100373274; M, chr9:101504820–101852863; N, chr9:123376788–125083831; O, chr9:126825879–127177239; P, chr9:129518474–129927677; Q, chr9:132985780–136636113; R, chr9:137291321–139534231; S, Chr14:62865875–63862093; T, chr3:186575268–187080482. In bottom panel (b), the regions are: A, chr3:57677987–58154068; B, chr3:61422777–73764765: C, chr3:87035206–88461236; D, Chr3:78706269–79206160; E, chr10:16585949–17022250; F, chr10:26833288–28028304; G, chr3:161705726–162566403; chr17:8028078–9207567. The regions are ranked A onwards in order of decreasing statistical significance.

Gene expression array data were available for 29 samples^12^. We used integrative analyses to further delineate key genes in transition from PPOLS to HNSCC. We identified genes that showed significant correlation of copy number with gene expression, from genomic regions that showed statistically significant difference in frequency of SCNA/LOH between PPOLs and HNSCC cell lines (detailed in Supplementary Data S16). This identified 67 genes (Fig. 5) of which 50 have been previously reported to be show some association with cancer (including *NOTCH1* and *PIK3CA*) using PUBMED search. Nine of the 50 genes have been previously associated with HNSCC including *DVL3*, and 5 genes (*NOTCH1*, *PPP6C*, *RAC1*, *EIF4G1*, *PIK3CA*) were identified as mutational cancer drivers in IntOGen (Release 2014.12).

Surprisingly, many genes in GISTIC regions that showed high frequency of SCNAs in HNSCC including known HNSCC cancer drivers did not show correlation with gene expression. *A priori* it may be expected that expression of tumour suppressor genes is less likely to show correlation with SCNAs as there are multiple ways of sustaining loss-of-function whilst gain-of-function changes are more limited and often through copy number gains. However, oncogenes such as *MYC* also failed to show correlation. We tested whether integrative analysis was a reliable method to predict *in vivo* protein expression by analyses of two genes (*BCL2L1* encoding an apoptosis regulator and *CLDN1* encoding a component of tight junctions in epithelia) that showed a high frequency of copy number gains in HNSCC but were discordant with respect to correlation of copy number with gene expression in this study. Both *CLDN1* and *BCL2L1* showed significantly increased expression (p < 0.0001) by immunohistochemistry in HNSCC compared to normal tissues and PPOL (shown in Supplementary Data S17) indicating that correlation of SCNA with transcript expression in integrative analyses may not be a reliable surrogate indicator of functional importance of a gene.

#### Peak regions of SCNA in PPOLs and HNSCCs show significant progressive enrichment of genes involved in cancer pathways

As a further means for systematically identifying relevant genes in the GISTIC regions and the biological pathways involved in HNSCC evolution, we looked for enrichment of genes in relevant KEGG pathways in the GISTIC peak regions.

**Figure 6:**
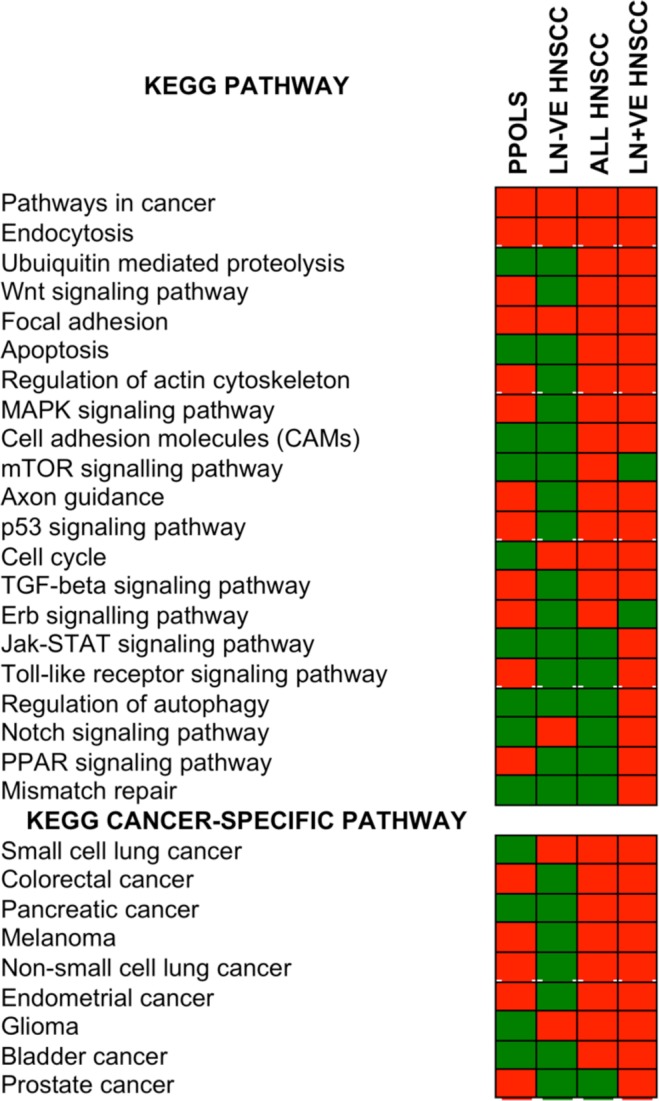
Enrichment of genes in cancer–relevant pathways in GISTIC extended regions in PPOLs and HNSCCs. Statistically significant enrichment (adjusted P < 0.01) for specific pathways is indicated by a red box and lack of enrichment by a green box. The data are shown in detail in Supplementary Data 18. The ranges for adjusted P values corrected for multiple testing were: ‘All HNSCC’, 1.97E-12-0.0098; ‘LN + ve HNSCC’, 5.76E-14-0.0083; ‘LN − ve HNSCC’, 7.52E-06-0.0074; PPOL, 5.34E-09-0.0007. In each section, the pathways were ranked from top to bottom in order of level of significance in the ‘All HNSCC’ group with highest level of significance at the top.

Significant (adj. P < 0.01) enrichment of genes in KEGG ‘pathways in cancer’ as well as other specific cancer pathways was observed in both PPOLs and HNSCC cell lines (this is shown schematically in Fig. 6 and detailed in Supplementary Data S18). The enrichment was observed even if the analysis was limited to 1466 genes in GISTIC regions that showed significant correlation with expression after correction for multiple testing (adj. p < 0.05) in immortal PPOL and HNSCC cell lines for which expression data was available.

The increase in both the size and the number of the SCNA regions in HNSCC compared to PPOLs, and the differences in SCNAs between LN + ve and LN − ve HNSCC cell lines, were reflected in the progressive increase and/or differences in the enrichment for relevant KEGG pathways genes (Fig. 6). Some of the pathways identified such as TP53 signalling and the cell cycle and genes therein, have been well characterised in HNSCC. However, other pathways such as ubiquitin–proteosome pathway and/or many of the genes within these pathways have not been implicated in HNSCC previously.

**Figure 7:**
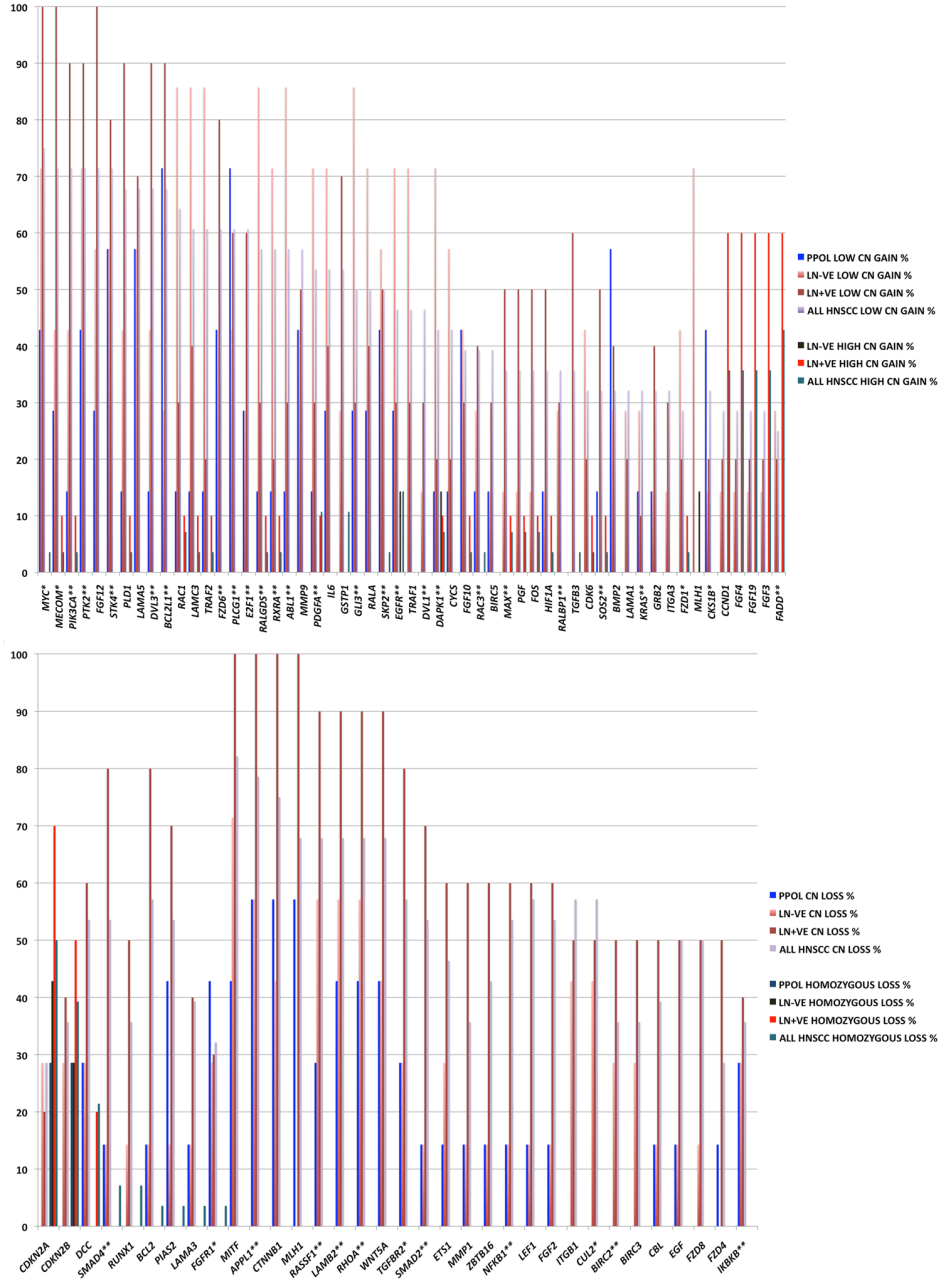
Frequency of SCNAs of genes involved in KEGG ‘All pathways in cancer’ pathways that are significantly enriched in the GISTIC regions in PPOLs and HNSCC. Copy number gains (top panel) and copy number losses (bottom panel) in PPOLs, all HNSCC, and HNSCCs with and without nodal metastases (LN + ve and LN − ve respectively). **Genes showing significant correlation with expression in integrative analyses after correction for multiple testing (adj. p < 0.05). *Genes showing nominal significance (p < 0.05) only are indicated by a single asterisk. Only genes showing at least 40% frequency of SCNA in at least one subgroup, are shown.

**Figure 8:**
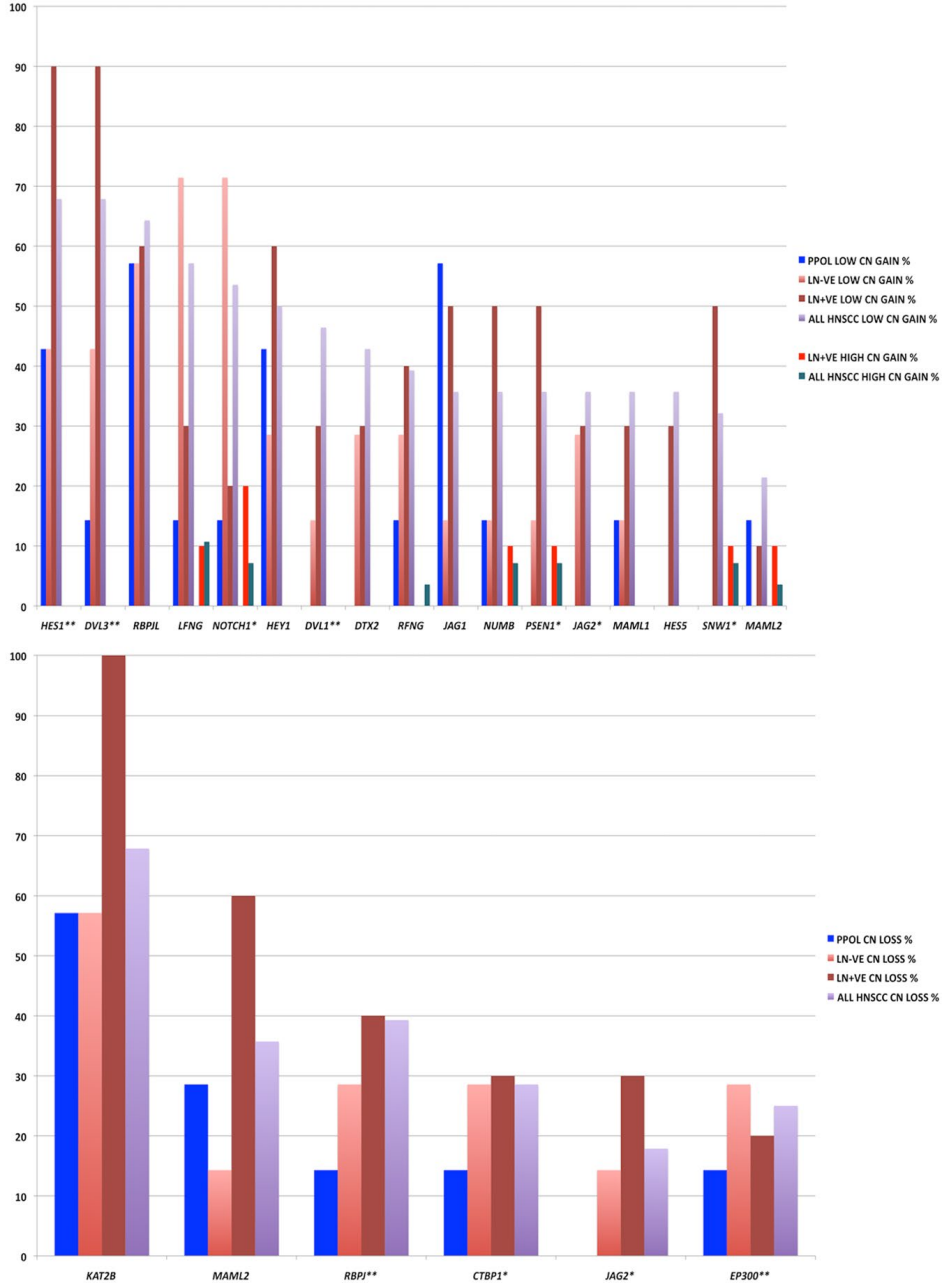
Frequency of SCNAs of genes involved in KEGG NOTCH signalling pathway that are significantly enriched in the GISTIC regions in PPOLs and HNSCC. Copy number gains (top panel) and copy number losses (bottom panel) in PPOLs, all HNSCC, and HNSCCs with and without nodal metastases (LN + ve and LN − ve respectively). **Genes showing significant correlation with expression in integrative analyses after correction for multiple testing (adj. p < 0.05). *Genes showing nominal significance (p < 0.05) only are indicated by a single asterisk. Only genes showing at least 40% frequency of SCNA in at least one subgroup, are shown.

Similarly, the pathway analyses identified many of the genes already known to be implicated in HNSCC confirming the utility of this approach. Significantly, in addition, this allowed identification of many additional cancer-relevant genes (e.g., *RHOA*, *PDL1*, *E2F1*) from regions of frequent SCNA (Figs 7 and 8 and further pathways shown in Supplementary Data S19).

The examination in our cell line panel, of the frequencies of SCNAs involving genes in individual pathways, allowed us to map multiple pathway changes to stages in HNSCC progression (Fig. 8 and further pathways shown in Supplementary Data S19). For example, enrichment in SCNAs of genes in the *NOTCH1* pathway was observed in both LN − ve and LN + ve cell lines (Fig. 8). However, there were differences in the frequencies of SCNAs of the individual genes in the pathway suggesting different alterations of the pathway. LN + ve cell lines showed almost universal amplification of *HES1* and *DVL3* and universal loss of *KAT2B*. LN + ve cell lines also showed a relatively low frequency (20%) of high and low copy number gains of *NOTCH1*. Gain of *DVL3* and loss of *KAT2B* together with a relatively high frequency of gain of *NUMB* and loss of *MAML2* may be expected to disrupt *NOTCH1* signalling but high frequency gains of the downstream targets *HES1* and *HEY1* may negate these changes or alternatively suggest complex amplification and inhibition of subsets of NOTCH signalling elements. Clearly, these genes act in multiple pathways and it is difficult to determine the effect of gain or loss of any single gene without functional analyses. Thus, copy number gains of *DVL3* may be of significance in WNT signalling pathway or in the cross-talk between the two pathways. Amplification of *HES1* and *DVL3* and loss of *KAT2B* were also observed in LN − ve cell lines as well as PPOLs albeit at a lower frequency. Instead, the LN − ve cell lines were characterised by a high frequency, low copy number gains of *NOTCH1* and *LFNG*. In our small sample set, cell lines with mutations and amplifications of the *NOTCH1* locus were mutually exclusive. Some genes such as *MAML1* and *MAML2* showed both copy number gains and losses possibly indicating further heterogeneity in NOTCH pathway aberration.

## Discussion

Many recent studies have reported genetic and epigenetic changes in HNSCC^4–8^. Here, we have focussed on mapping the genetic changes to the stages of evolution of HNSCCs from the earliest stages to transition to malignancy and progression to metastases. Given this focus and the need for well-characterised panel of cell lines from premalignant lesions and HNSCCs with and without lymph node metastases, the sample size is necessarily small. Thus, the prevalence of the changes observed in this study must be regarded with caution. In addition, the changes observed in cell lines may not be fully representative of primary tumour because of clonal selection and continuing evolution in culture. However, the patterns of changes observed in our study are consistent with previous studies of primary HNSCC tumours^3–9^. Our findings are also consistent with findings of Beroukhim *et al*.^28^ study of 3131 primary tumours of different sites. One major advantage of using cell lines is that extensive heterogeneity of primary tumour samples can mask SCNAs^30,31^, whereas cell lines give a cleaner results^32^.

Given the relatively low frequency (~30% or less) of sequence mutations of genes other than *TP53* both in the current and previous studies, together with prevalence of consistent and frequent SCNA changes across wide range of tumours^28^, it is likely that the selection processes in the clonal evolution of these tumours is driven by these SCNAs. Additionally, we show here that the earliest changes are often characterised by focal SCNAs (for example, deletions at chromosome 8p23 involving *CSMD1*) with further selection for loss of whole arm or large region of arm during progression or evolution. This suggests that regions of SCNAs harbour multiple genes that collectively provide selective growth advantage. This is supported by demonstration of enrichment of genes in the cancer relevant pathways in regions of peak SCNAs identified in HNSCC. Several of these genes have previously individually been associated with HNSCC but the present study is the first to systematically identify these genes and others, in the context of cancer-relevant pathways and map them to cancer progression.

In the present study, we confirm previous observations^17,24–26^, that loss-of-function of *TP53* (primarily through sequence mutations) and *CDKN2A* (through SCNA, promoter hypermethylation and sequence mutations) are the earliest changes in HNSCC development. Furthermore, we demonstrate for the first time that deleterious sequence mutations in previously identified HNSCC cancer drivers *KMT2D*, *PIK3CA*, and *NOTCH1*, are also present in PPOLs and represent early but less common changes. Our study also provides indication that other genes such as *MGA*, *PABPC3*, *NR4A2*, *NCOR1* and *MACF1* are targets for deleterious mutations early in HNSCC progression. We provide evidence that inactivation of *CSMD1* occurs very early in HNSCC and provide functional evidence to support a tumour suppressor role. This is consistent with findings in breast cancer^33^. *CSMD1* encodes for a predicted transmembrane protein with a multidomain extracellular structure that is likely to act as a multi-ligand receptor mediating endocytosis of ligands. However, this remains to be characterised. One previous study has suggested a role for *CSMD1* in reducing proliferation and invasive potential possibly through *SMAD* pathway^34^. Whilst we did not detect mutations in *FAT1* (a gene known to be mutated in HNSCC) in PPOL, we did identify hemizygous and homozygous deletions of the gene confirming that this gene is also an early target for inactivation in HNSCC development.

In contrast to study by Bhattacharya and colleagues^9^, this study did not find evidence of significant subset of immortal PPOLs or HNSCCs that didn’t show loss at 8pter-p23.1 together with gains on 3q24-qter, 8q12-q24.2, and chromosome 20. However, our sample size is relatively small and there may be a selection in culture of the cells with these genetic alterations. Interestingly, in the previous study^9^ the subgroup lacking these genetic alterations showed genetic stability, lack of *TP53* mutations and a much-reduced predisposition to metastasis. It is interesting to speculate whether these correspond to ‘mortal’ PPOLs and HNSCC cultures, which are genetically stable and also lack *TP53* mutations.

We were able to demonstrate significant differences between LN + ve and LN − ve HNSCC cell lines particularly with respect to a higher frequency of high copy gains on chromosome arm 11q centred around *CCND1* and mir-548k, and chromosome arm 3q centred on *TP63*.

Our findings with respect to mutations and SCNA are similar but not identical to those reported by Wood and colleagues^19^ in synchronous dysplasia and HNSCC. In our study, we were able to show progressive changes with transformation to malignancy and lymphovascular spread. We note that in their study only a minority of low-grade dysplasias showed changes that were present in high-grade dysplasia and HNSCC, and they suggested that SCNAs were not necessary for the low-grade dysplasias to develop. We interpret this with some caution as unsurprisingly, low-grade dysplasia can be very difficult to distinguish from normal tissues with absolute certainty and agreement between histopathologists is generally weak in grading low-grade dysplasia. Thus, some low-grade dysplasia lesions may represent genuine dysplasia whilst others may represent normal tissues. Regardless of this, what is clear from our study is that the SCNAs arise after the breakdown of cellular senescence. The mortal PPOLs do not display SCNAs. Additionally, the loss of expression of *CDKN2A* is nearly ubiquitous in our PPOL panel^17^ and in PPOL tissues *in vivo*^12,25,35^.

*NOTCH1* mutations^4,5^ and *NOTCH1* pathway alterations have been reported in HNSCC^6^. We have extended these findings and demonstrate that *NOTCH1* mutations are present in one of the three progressive PPOLs but not in any of the non-progressive lesions. This indicates that *NOTCH1* inactivation is a relatively early event but still consistent with previous observations that suggest that *NOTCH1* inactivation plays a key role in progression to invasive carcinoma of already initiated cells^36^. Our data are also consistent with that of Agrawal and colleagues^5^ who have shown no association or mutual exclusivity of *NOTCH1* and *TP53* mutations. Interestingly, the results of the present study demonstrate that SCNAs of several genes in the NOTCH1 signalling pathway including *NOTCH1*, are more frequent then loss-of-function mutations of *NOTCH1*. Furthermore, many (but not all) of these SCNAs are gain of function changes in the NOTCH1 pathway. This is consistent with recent observations in primary HNSCC demonstrating over-expression of both ligands and receptors in this pathway^37,38^. Although mainly loss-of-function mutations in *NOTCH1* have been reported in HNSCC to date, activating mutations in HNSCC in a Chinese population have also been described recently^39^. Our data support the suggested dual oncogenic and tumour suppressive role for *NOTCH1* in HNSCC. However, further functional analyses are necessary to confirm this. Additionally, the recent finding of a higher frequency of *NOTCH1* mutations in aged oesophageal mucosa compared to tumours, raises the possibility that *NOTCH1* mutations do not play a significant role in HNSCC^40^. Some SCNA’s of *NOTCH1* pathway genes such as copy number gains of *DVL3* may also be significant in the context of cross-talk with the WNT signalling pathway.

In this study, we identified a potentially deleterious *NOTCH1* variant in one mortal HNSCC lines. If the mutation in the mortal line which has a wild type p53^36^ and no detectable CNVs (this study) or LOH^16^ is pathological as predicted, it would suggest that *NOTCH1* mutations are independent of genomic instability and consistent with the recent finding of frequent deleterious *NOTCH1* mutations in aged oesophageal mucosa^40^.

The presence of *NOTCH1* mutations in this line also offers a plausible explanation for the poor differentiation of this line in both suspension^16,41^ and surface culture^16^ and is also consistent with recent data showing that the knockdown of *NOTCH1* expression in human keratinocytes recreates a poorly differentiated epithelium reminiscent of dysplasia^42^. Furthermore, *NOTCH1* has been shown to mediate keratinocyte stratification^41,43^ and stem cell maintenance^44^. However, *NOTCH1* deletion has also been shown to promote tumourigenesis and tumour progression through paracrine effects^45^, the former of which would also be consistent with *NOTCH1* mutations in PPOLs. Moreover, this last observation would be consistent with the reports of *NOTCH2* and *NOTCH3* mutations in human SCC^4^ because loss-of-function of these paralogues promotes tumourigenesis in mouse skin in a paracrine fashion but does not replicate the effect of *NOTCH1* deletion on keratinocyte differentiation^45^. It has also been reported that *NOTCH1* is a TP53 target gene^41^ and as most of the immortal PPOL and HNSCC lines have TP53 mutations this could be an additional mechanism of its inactivation and is consistent with the altered regulation of hairy enhancer of split 2 (*HES2*) in these lines^12,41^.

In conclusion, we have further characterised specific genetic changes that mark progression in head and neck squamous cell carcinogenesis. Although genomic landscapes and progression models of SCCHN^4–7,15^ have been published previously, we have been able to use our well characterised cell line panels to tentatively assign genetic changes, including novel ones, to specific stages of progression in transcriptionally distinct mortal and immortal classes^10,12^ of the disease and also to cell function. The findings of this study, in addition to adding to our knowledge of potential druggable alterations in HNSCC, increase the understanding of early stages of HNSCC evolution, which may contribute to better control of premalignancy and prevention of progression to frank malignancy.

## Materials and Methods

### Samples

All methods were carried out in accordance with relevant guidelines and regulations. The use of patient samples in parts of this study was approved by the UK National Research Ethics Service (REF No: 08/H1006/21 and EC.47.01). Primary tumour samples were collected with full informed consent under research ethics approval EC.47.01. Anonymised archival samples used in immunohistochemistry analyses were used with consent exemption granted by research committee approval 08/H1006/21 under the UK Human Tissues Act 2004.

Details of the samples are shown in Supplementary Data 1. For SNP array, the sample set consisted of 16 HNSCC cell lines, 7 PPOL cell lines and 11 mortal cell cultures derived from PPOL. DNA from matching fibroblasts was available for 6 HNSCC cell lines, 1 immortal PPOL cell line and 2 mortal PPOL cultures. The sample culture conditions were as described previously^16^ and DNA was prepared from cell lines using standard protocols. The sample set for array CGH consisted of 12 HNSCC cell lines.

### SNP array analyses

SNP genotyping of the primary HNSCC panel was performed using the Illumina HumanHap550 Genotyping Beadchip and Infinium Assay II as per standard protocols. DNA from the cell lines was quantitated with NanoDrop (Thermo Scientific) and 750 ng was used per assay.

### Array CGH

ArrayCGH data were kindly provided by Dr Simon Deardon, AstraZeneca, UK.

### Data analyses

The average genotype call rate was 98.25%; genotype data from two samples with a call rate of <95% in BeadStudio v3.1 (Illumina) were excluded from analyses. Over 75% of samples had GenTrain score (measure of reliability based on the total array of calls for a given SNP) of ≥0.7 and none were below 0.4. Data were pre-processed in GenomeStudio v2009.1 (Illumina) and imported into Nexus Copy Number v5.1 (BioDiscovery, Inc., CA, USA) and OncoSNP v2.7^46^ for further analyses. ArrayCGH data from a second HNSCC panel were also imported into Nexus Copy Number v7.5 (BioDiscovery, Inc., CA, USA).

The robust variance sample QC calculation (a measure of probe to probe variance after major outliers due to copy number breakpoints are removed from the calculation) in Nexus Copy Number v7.5 (BioDiscovery, Inc.), was used to assess the quality of the samples. Data for samples with a score >0.2 were excluded and the score for the remaining samples was in the range 0.03–0.20. For identification of copy number and copy neutral changes, the BioDiscovery’s SNPRank Segmentation Algorithm was used with significance level of 1 × 10^−6^ and a minimum number of probes per segment of 5. Thresholds for determining copy number variation were set at −1 for homozygous deletion, −0.18 for hemizygous deletion, 0.18 for gain (single copy gain) and 0.6 for high gain (2 or more copies). An area was considered to be showing LOH if 95% of the probes in the region had a B allele frequency of >0.8 or <0.2 (homozygous frequency and value thresholds of 0.95 and 0.8 respectively). Allelic imbalance was defined as 95% of the probes in the region showing a B allele frequency of between 0.2 and 0.4 or 0.6 and 0.8 (i.e. heterozygous imbalance threshold of 0.4).

Areas of the genome with a statistically high frequency of aberration (Q-bound value <=0.5–0.25 and G-score cut-off <=1) after correction for multiple testing using FDR correction (Benjamini & Hochberg), were identified using the GISTIC (Genomic Identification of Significant Targets in Cancer) approach^47^.

Group comparisons were made in Nexus with differences in frequency of specific events at any chromosomal location tested for significance by two-tailed Fisher’s Exact Probability Test with an accepted significance level of p < 0.01 at a defined level of percentage difference. More stringent Q-bound values based on two-tailed Fisher’s Exact Probability Test corrected for multiple testing using Benjamini-Hochberg FDR correction (Benjamini & Hochberg) as well as a minimum of set percent difference in frequencies between the two groups; significance was accepted at <0.25.

### Promoter methylation analyses

Genomic DNA (approximately 1 mg each) from all the HNSCC and PPOL cell lines and matched primary HNSCC samples used were subjected to bisulfite treatment using the EZ DNA methylation^TM^ kit (Zymo Research, USA) according to the manufacturer’s protocol. 30 ng each of the bisulfite-treated DNA was used for the pyrosequencing reaction.

PCR and sequencing primers for the pyrosequencing methylation analyses of the CpG rich promoter region were designed using the pyro-Q-CpG software for *CSMD1*. The forward primer was biotinylated and was used in low concentration (5 pmol) along with amplification cycles of 45 to exhaust the primers. The forward and reverse primer sequences used in the study were *CSMD1*-F-5′gtagttttagatagatagagtttagttt3′, R-5′acaaatctcctttctcca3′ with its sequencing primer 5′ aaatctcctttctccaacct3′. Optimized annealing temperature for *CSMD1* PCR primer pairs is 54 °C. Using bisulfite-treated DNA as a template, regions of interest were amplified by standard PCR cycling conditions in a 96-well plate using Qiagen’s Hot start Taq Polymerase to avoid nonspecific amplification. The specificity of the PCR products was then verified by agarose gel electrophoresis. For the pyrosequencing reaction, the PCR product was made single stranded by immobilizing the incorporated biotinylated primer on streptavidin-coated beads. The sequence run and analysis were done on the PyroMark™Q96 MD pyrosequencer (Qiagen) according to the manufacturer’s instructions. The sequence runs were analysed using the Pyro Q-CpG software. The peak heights observed represented the quantitative proportion of the alleles. The software generated methylation values for each CpG site and also the mean methylation percentage for all the CpG sites analyzed.

### Generation of stable *CSMD1*-expression modulated clones

A panel of nine OSCC cell lines was profiled for *CSMD1* transcript and protein expression status (data not shown). This identified the *CSMD1*-expressing cell line BICR16 and the *CSMD1* non-expressing cell line H103.

BICR16 cells were used to generate stable *CSMD1*-silenced monoclone and polyclone lines with HuSH 29mer pRS shRNA (Origene, Rockville, MD, USA). The degree of silenced *CSMD1* expression level was confirmed by RT-qPCR and flow cytometry assays. Primer sensitivity assays determined the limit of *CSMD1* transcript detection at five ORF copies per light-cycler well.

Stable forced *CSMD1*-expressing monoclone cells were generated by transfection of 15.5 kb pCMV6-*CSMD1* expression plasmid (Origene, Rockville, MD, USA) into the *CSMD1*-deleted cell line H103. The plasmid was linearized at the SexAI restriction site within a predetermined 14% non-essential region and used to generate seven *CSMD1*-expressing monoclone cell lines using standard methods. The degree of forced *CSMD1* expression was confirmed by RT-qPCR and flow cytometry assays.

Cell proliferation was determined with CellTiter 96® Aqueous Cell Proliferation MTS Assay (Promega, Southampton, UK) as per the manufacturer’s protocols. Cell growth was calculated as a percentage growth change from the 24-hour time point and population-doubling times were determined. Gel-invasion was assayed using trans-well BD Bio-coat Matrigel Invasion Chambers and control wells (BD Biosciences, Oxford) as per manufacturer’s protocols. Optimal seeding densities were determined empirically. Triplicate Matrigel invasion chambers were used for each clone from a minimum of two different Matrigel batches. Three fields of view were captured for each Matrigel or control chamber (outer, middle, centre areas, each at 120° rotation from one another). Percentage invasion was calculated for each clone and expressed as an invasion index (ratio of clone to parent percentage invasion). Statistical analysis was performed in IBM SPSS 20 & 22 (Wilcoxon Sign-Rank Test) with alpha levels set at 0.05.

### Integrative analysis

Before correlating the SCNA and gene expression values corrections were applied for polyploidy and heterogeneity.

When considered a by-product of instability rather than a response of biological significance, the copy number (CN) values were altered to remove the ubiquitous chromosomal amplification observed in polyploid samples. To do so, all the CN values inferred for SNPs in chromosome arms with mean CN larger than 2.5 were reduced by one unit. The reduction was however rejected in the cases of heterozygous copy neutral calls (CN2 LOH0), copy losses (CN1) and homozygous deletions (CN0). These states were not altered.

Due to heterogeneity, the gene expression values obtained did not represent the expression of the CN-altered cells solely. For each genomic region, a non-negligible proportion of cells do not harbour any alteration. The proportion of cells with normal heterozygous copy number in each region was estimated. When investigating the correlation between SCNAs and mRNA expression the weighted mean CN and LOH values of this mixture were used.

### Exome sequencing

Targeted enrichment and sequencing were performed on 1–3 µg of DNA extracted from the cell lines. Enrichment was performed using the SureSelect Human All Exon 50 MB v4 Kit (Agilent, Santa Clara, CA, USA) for the Illumina system. Sequencing was carried out on a HiSeq 2500 sequencer (Illumina Inc, San Diego, CA, USA), following the manufacturer’s protocols.

### HaloPlex sequencing

Targeted enrichment and sequencing were performed on 225 ng of DNA extracted from the cell lines. Enrichment was performed using a custom HaloPlex Kit (Agilent, Santa Clara, CA, USA) targeting 41 genes. Sequencing was undertaken on a MiSeq sequencer (Illumina Inc, San Diego, CA, USA), following the manufacturer’s protocols.

### Sequence data analysis

Raw paired-end reads were trimmed using Trimmomatic v0.33 to a minimum length of 30 nucleotides. Illumina Truseq adapters were removed in palindrome mode. A minimum Phred quality score of 30 was required for the 3′ end. Single end reads as well as paired end reads that failed previous minimum quality controls were discarded. Individual read groups were aligned, using bwa v0.7.12 with default parameters, to the UCSC hg19 reference human genome from Illumina iGenomes web site. Trimming rates and insert length were controlled on each read group based on metrics reported by Trimmomatic, and Picard v1.128 respectively.

Aligned reads from multiple read groups belonging to the same sample were indexed, sorted and merged using sambamba v0.5.4. Amplification duplicates were removed using Picard.

Various quality controls parameters were used including the obtained target coverage of the Nextera Rapid Capture exome library v1.2, mapping rates and duplication rates, based on metrics collected for each sample using Samtools v1.2, Picard v1.141, bedtools v2.25.0, and aggregated using custom Python v2.7.9 codes.

We applied GATK v3.5.0 base quality score recalibration and indel realignment^14^ with standard parameters. We performed SNP and INDEL discovery and genotyping across each cohort of samples simultaneously using standard hard filtering parameters according to GATK Best Practices recommendations.

All variants were annotated with functional prediction using SnpEff v4.2. Additionally, functional annotation of variants found in two public databases (NCBI dbSNP v144 and dbNSFP v2.9) was added using SnpSift, part of the same software package. Multiallelic variants were decomposed and normalized using vt. A GEMINI v0.18.3; a database was created^15^, and variants selected according to functional rules. Finally, they were manually validated against read alignments, using Integrative Genomics Viewer software (IGV) v2.3. Coverage metrics were calculated using bedtools.

### Variant filtering and mutation landscape plots

#### Variant filtering and mutation landscape plots

Variants with ExAC (non TCGA) population alternate allele frequency of 0.5% and above were removed from the VCFs created in the HaplotypeCaller analysis. Further probable germline variants were removed if they were labeled as germline in ClinVar database or if present in dbSNP, except if labeled as pathogenic in ClinVar or if present in COSMIC. The final resulting VCFs were then converted to MAFs using vcf2maf v1.6.16. Variants that were non-coding or silent were removed, as those found in more than two samples, unless they were found only in the exome and HaloPlex capture of a same sample. Finally, to remove other potential rare germline variants or artefacts, we removed variants that were found in genes labeled as “High” in the “all cancer disease-causing genes” of the gene damage index (GDI)^48^, unless these were known HNSCC mutational cancer drivers according to IntOGen^49,50^. Mutation landscape plots were then created using the waterfall function in GenVisR^51^.

In total, 461 689 variants were detected in the 9 exome captures and 5337 variants in the 17 HaloPlex captures. Removing variants that had a population alternate allele frequency of 0.5% in the ExAC (non TCGA) database as well as additional germline filtering (described in Materials and Methods) gave 56 520 and 1189 variants. Keeping only coding variants with CADD scores of at least 10, that were found in a maximum of 2 samples brought these numbers down to 6436 and 68. However, a number of these were in genes known to cause a high amount of false positive somatic variants. Thus, we removed variants in genes that were labeled as “High” in the “all cancer disease-causing genes” of the gene damage index (GDI)^48^, unless these were known HNSCC mutational cancer drivers according to IntOGen^49,50^. This led to a final number of variants of 5766 for the exome captures and 59 for the HaloPlex captures.

## Supplementary information

Supplementary Data S1-4 & S6-19

Supplementary Data S5

## Data Availability

The data for this study is available through Dryad Digitial Repository (10.5061/dryad.314k5k5).
