# Supplementary material for: Analysis of head and neck carcinoma progression reveals novel and relevant stage-specific changes associated with immortalisation and malignancy
Source: Sci Rep. 2019 Aug 19;9:11992. doi: 10.1038/s41598-019-48229-7 (PMC6700135; doi:10.1038/s41598-019-48229-7)

**SUPPLEMENTARY DATA S1-S4, S6-S19**

**Analysis of head and neck carcinoma progression reveals novel and relevant stage-specific changes associated with immortalisation and malignancy.**

Ratna Veeramachaneni, Thomas Walker, Timothée Revil, Antoine De Weck, , Dunarel Badescu, James O’Sullivan, Catherine Higgins, Louise Elliott, Triantafillos Liloglou, Janet M. Risk, Richard Shaw, Lynne Hampson, Ian Hampson, Simon Dearden, Robert Woodwards, Stephen Prime, Keith Hunter, Eric Kenneth Parkinson, Jiannis Ragoussis, Nalin Thakker.

**Supplementary Data- Contents**

| **No** | **Supplementary Data** | **Page** |
| --- | --- | --- |
| S1 | Details of sTUDY Samples | 3 |
| S2 | Cumulative distribution of coverage showing the fraction of targeted bases that were covered by at least a certain depth in EXOME SEQUENCING | 6 |
| S3 | Cumulative distribution of coverage showing the fraction of targeted bases that were covered by at least a certain depth in HALOPLEX SEQUENCING | 7 |
| S4 | FILTERING OF SEQUENCE VARIANTS DETECTED BY EXOME AND HALOPLEX CAPTURE | 8 |
| S6 | COMPARISION OF SCNAs IN TWO HNSCC PANELS | 9 |
| S7 | SUBTRACTION KARYOGRAM OF MORTAL PPOLS AND MATCHING FIBROBLASTS | 10 |
| S8 | ImmORTAL PPOL CELL LINES SCNA DENSITY PLOTS FOR CHROMOSOMES 3, 8, 9 AND 20 | 11 |
| S9 | Hierarchical Cluster Analysis of Immortal HNSCC Cell Lines For High Copy Number SCNA - Association With Lymph Node Metastases | 12 |
| S10 | GENES IN GISTIC PEAK REGIONS IN PPOLS AND HNSCC CELL LINES | 13 |
| S11 | copy number gains and losses of the top ranked genes in top ranked peaks regions of focal deletions identified by Beroukhim et al., 2010 | 25 |
| S12 | FREQUENCY OF COPY NUMBER ALTERATIONS of INTOGEN-DERIVED CANCER DRIVERS GENES IN PPOLS and HNSCC CELL LINES | 29 |
| S13 | DELETIONS IN HNSCC CELL LINES AT THE CSDM1 LOCUS | 40 |
| S14 | Modulation of CSMD1 Expression in Immortal HNSCC Cell Lines | 41 |
| S15 | ANALYSIS OF NCKAP5 | 42 |
| S16 | Integrative Analysis of Somatic Copy Number Changes and Gene Expression | 49 |
| S17 | Analysis of CLDN1 and BCL2L1 Expression in Primary HNSCC and PPOL | 55 |
| S18 | KEGG PATHWAY GENE ENRICHMENT IN GISTIC REGIONS | 59 |
| S19 | SCNA of genes in cancer realted kegG pathways enriched in gistic regions | 60 |

**Supplementary Data S1 – Details of Study Samples**

|  | Biopsy | |  |  | Patient |  | Culture |
| --- | --- | --- | --- | --- | --- | --- | --- |
| Name | Site | lesion | stage | smoking | age | sex | Lifespan (PDs) |
| Mortal PMOLs |  |  |  |  |  |  |  |
| D6 | PT | Leukoplakia | mod/severe | y | 55 | M | 25 |
| D8 | FOM | Leukoplakia | mild/mod | y | 71 | M | 9 |
| D25 | FOM | Leukoplakia | severe | y | 58 | M | 28 |
| D30 | FOM | Leukoplakia | mild | y | 52 | M | 30 |
| D47 | FOM | Leukoplakia | mod | y | 82 | F | 20 |
| D48 | FOM/VT | Leukoplakia | mod/severe | y | 62 | F | 25.5 |
| D17 (EL) | BM | Leukoplakia | mild/mod | y | 61 | M | 61 |
| E1 | T | Erythroplakia | CIS | U | U | M | 24 |
| E2 | ALV | Erythroplakia | CIS | U | U | M | 17 |
| E4 | LT | Erythroplakia | CIS | U | 65 | F | 41 |
| E5 | LT | Erythroplakia | severe | U | 61 | M | 31 |
| Immortal PMOLs |  |  |  |  |  |  |  |
| D4 | FOM/VT | Leukoplakia | CIS | y | 51 | M | >100 |
| D9 | VT | Leukoplakia | mild/mod | n | 84 | M | >100 |
| D34 | LT | Leukoplakia | mod | n | 54 | F | >100 |
| D38 | LT | Leukoplakia | mild | n | 55 | F | >100 |
| D19 | LT | erythroleukoplakia | Severe/CIS | y | 53 | M | >100 |
| D20 | LT | Leukoplakia | moderate | n | 50 | M | >100 |
| D35 | FOM/VT | erythroleukoplakia | Severe/CIS | y | 68 | M | >100 |
| Mortal OSCCs |  |  |  |  |  |  |  |
| BICR 66 (B66) | RM/T | Carcinoma | T2N0M0 | y | 56 | M | 37 |
| BICR 80 (B80) | LNX | Carcinoma | T4N2CM0 | y | 71 | M | 62 |
| BICR 37 (B37) | Primary:T | Metastasis | T4N2CM0 | U | U | F | 32 |
| Immortal OSCCs |  |  |  |  |  |  |  |
| BICR 3 (B3) | ALV | Carcinoma | T2N0M0 | y | 56 | F | >130 |
| BICR 31 (B31) | T | Carcinoma | T4N2BM0 | U | U | M | >130 |
| BICR 56 (B56) | T | Carcinoma | T4N1M0 | y | 59 | F | >130 |
| BICR 68 (B56) | BT | Carcinoma | T4N0M0 | n | 75 | F | >130 |
| T4 (T4) | FOM | Carcinoma | T4N0 | U | U | F | >130 |
| T5 (T5) | BM | Carcinoma | T2N2 | Y | 59 | F | >130 |
| BICR 78 (B78) | ALV | Carcinoma | T4N1M0 | U | 68 | M | >130 |
| BICR 63 (B63) | T | Carcinoma | T2N2BM0 | y | 70 | M | >130 |
| BICR10 (B10) | BM | Recurrence | T4N0M0 | n | 84 | F | >130 |
| BICR16 (B16) | T | Recurrence | T2N0M0 | y | 49 | M | >130 |
| BICR82 (B82) | MX | Recurrence | N.D. | y | 48 | M | >130 |
| BICR 22 (B22) | Primary:T | Metastasis | T4N3M0 | U | 88 | M | >130 |
| BICR 18 (B18) | Primary:LN | Metastasis | T4N1M0 | U | U | U | >130 |

**Table 1: Clinical data of the PPOLs and HNSCC cell lines.** Key: ALV, alveolus; BM, buccal mucosa; BT, base of tongue; CIS, carcinoma in situ; FOM, floor of mouth; HP, hypopharynx; LN, lymph node; LNX, larynx; LT, lateral tongue; MX, maxilla; N/A, not applicable; N.D., not determined; normal adjacent, apparently normal mucosa from patient with SCC; PT, posterior tongue; RM, retromolar trigone; T, tongue; U, information not available; VT, ventral tongue. The tumour names given in brackets are the abbreviations used in the text (adapted from Hunter, *et al*., 2006)

| **Sample ID** | **Description** |
| --- | --- |
| E2N | Matching fibroblasts of E2 |
| E5N | Matching fibroblasts of E5 |
| D19N | Matching fibroblasts of D19 |
| BICR80N | Matching fibroblasts of BICR80 |
| BICR37N | Matching fibroblasts of BICR37 |
| BICR66N | Matching fibroblasts of BICR66 |
| BICR3N | Matching fibroblasts of BICR3 |
| BICR31N | Matching fibroblasts of BICR31 |
| BICR56N | Matching fibroblasts of BICR56 |
| BICR82N | Matching fibroblasts of BICR82 |
| BICR68N | Matching fibroblasts of BICR68 |
| BICR18N | Matching fibroblasts of BICR18 |
| BICR78N | Matching fibroblasts of BICR78 |
| BICR63N | Matching fibroblasts of BICR63 |
| BICR22N | Matching fibroblasts of BICR22 |
| H314K | HNSCC cell line |
| H413K | HNSCC cell line |
| H357K | HNSCC cell line |
| H314F | Matching fibroblasts of H314K |
| H413F | Matching fibroblasts of H413K |
| H357F | Matching fibroblasts of H357K |

**Table 2: Matching fibroblast for the HNSCC and PPOL cell line panels**

| **Patient ID** | **Tumour** | **Normal** |
| --- | --- | --- |
| **3230** | 2184 | 2185 |
| **3231** | 2188 | 2189 |
| **3234** | 2200 | 2201 |
| **3236** | 2208 | 2209 |
| **3239** | 2224 | 2225 |
| **3243** | 2240 | 2241 |
| **3244** | 2244 | 2245 |
| **3245** | 2248 | 2249 |
| **3247** | 2289 | 2290 |
| **3248** | 2293 | 2294 |
| **3249** | 2297 | 2298 |
| **4000** | 2257 | 2258 |
| **3313** | 2563 | 2564 |
| **3314** | 2569 | 2570 |
| **3315** | 2575 | 2576 |
| **3316** | 2581 | 2582 |
| **3317** | 2587 | 2588 |
| **3232** | 2192 | 2193 |
| **3233** | 2196 | 2197 |
| **3238** | 2220 | 2221 |
| **3240** | 2228 | 2229 |
| **3241** | 2232 | 2233 |
| **3242** | 2236 | 2237 |
|  |  |  |
| **Patient ID** | **Tumour** | **Normal** |
| **3230** | 2184 | 2185 |
| **3231** | 2188 | 2189 |
| **3234** | 2200 | 2201 |
| **3236** | 2208 | 2209 |
| **3239** | 2224 | 2225 |
| **3243** | 2240 | 2241 |
| **3244** | 2244 | 2245 |
| **3245** | 2248 | 2249 |
| **3247** | 2289 | 2290 |
| **3248** | 2293 | 2294 |
| **3249** | 2297 | 2298 |
| **4000** | 2257 | 2258 |
| **3313** | 2563 | 2564 |
| **3314** | 2569 | 2570 |
| **3315** | 2575 | 2576 |
| **3316** | 2581 | 2582 |
| **3317** | 2587 | 2588 |
| **3232** | 2192 | 2193 |
| **3233** | 2196 | 2197 |
| **3238** | 2220 | 2221 |
| **3240** | 2228 | 2229 |
| **3241** | 2232 | 2233 |
| **3242** | 2236 | 2237 |
|  |  |  |
| **Patient ID** | **Tumour** | **Normal** |
| **3230** | 2184 | 2185 |
| **3231** | 2188 | 2189 |
| **3234** | 2200 | 2201 |
| **3236** | 2208 | 2209 |
| **3239** | 2224 | 2225 |
| **3243** | 2240 | 2241 |
| **3244** | 2244 | 2245 |
| **3245** | 2248 | 2249 |
| **3247** | 2289 | 2290 |
| **3248** | 2293 | 2294 |
| **3249** | 2297 | 2298 |
| **4000** | 2257 | 2258 |
| **3313** | 2563 | 2564 |
| **3314** | 2569 | 2570 |
| **3315** | 2575 | 2576 |
| **3316** | 2581 | 2582 |
| **3317** | 2587 | 2588 |
| **3232** | 2192 | 2193 |
| **3233** | 2196 | 2197 |
| **3238** | 2220 | 2221 |
| **3240** | 2228 | 2229 |
| **3241** | 2232 | 2233 |
| **3242** | 2236 | 2237 |
|  |  |  |

| **No** | **Gender** | **Age** | **Site** | **Subsite** | **pT** | **pN** | **ECS** | **HPV status** |
| --- | --- | --- | --- | --- | --- | --- | --- | --- |
| 3230 | M | 73 | OSCC | Tongue | 2 | 1 | Y |  |
| 3231 | F | 72 | OSCC | missing | missing | missing | missing |  |
| 3232 | M | 44 | OSCC | AFOM | 1 | 0 | _ |  |
| 3233 | M | 55 | OSCC | AFOM | 2 | 2c | N |  |
| 3234 | M | 62 | OSCC | Tongue | 1 | x | _ |  |
| 3236 | F | 71 | OSCC | Mand Alveolus | 2 | 0 | _ |  |
| 3237 | M | 59 | OSCC | Retromolar | 2 | 2a | N |  |
| 3238 | M | 71 | OSCC | Mand Alveolus | 2 | 0 | _ |  |
| 3239 | M | 54 | OSCC | AFOM | 2 | 2b | N |  |
| 3240 | F | 39 | OSCC | Maxilla | 3 | 0 | _ |  |
| 3241 | F | 74 | OSCC | AFOM | 2 | 2b | Y |  |
| 3242 | F | 70 | OSCC | Tongue | 1 | 1 | N |  |
| 3243 | M | 80 | OSCC | Mand Alveolus | 2 | 0 | _ |  |
| 3244 | F | 51 | OPSCC | BOT | 2 | 0 | _ | negative |
| 3245 | M | 62 | OSCC | AFOM | 2 | 2b | N |  |
| 3247 | M | 54 | OSCC | Retromolar | 4 | 1 | N |  |
| 3248 | M | 67 | OSCC | AFOM | 2 | 2b | Y |  |
| 3249 | F | 74 | OPSCC | Tonsil | 2 | 1 | Y | negative |
| 3312 | M | 50 | OSCC | AFOM | 2 | 0 | _ |  |
| 3313 | F | 64 | OSCC | AFOM | 4 | 0 | _ |  |
| 3314 | M | missing | OSCC | AFOM | 2 | 0 | _ |  |
| 3315 | M | 52 | OSCC | AFOM | 2 | 0 | _ |  |
| 3316 | F | 44 | OSCC | Tongue | 2 | 0 | _ |  |
| 3317 | M | 42 | OPSCC | Tonsil | 3 | 2b | Y | positive |
|  |  |  |  |  |  |  |  |  |
| 3479 | M | 68 | OSCC | AFOM | 4 | 0 | _ |  |
| 3480 | M | 52 | OSCC | Tongue | 3 | 2b | N | negative |
| 3481 | M | 73 | OPSCC | Soft Palate | 2 | 1 | N | negative |
| 3494 | F | 49 | OPSCC | Soft Palate | 2 | 0 | _ |  |
| 3495 | M | 32 | OSCC | Tongue | 2 | 1 | N |  |
| 3497 | F | 74 | OSCC | Tongue | 2 | 0 | _ |  |
| 3499 | F | 81 | OSCC | Tongue | 4 | 2c | missing |  |
| 3500 | M | 61 | OSCC | Tongue | 2 | 0 | _ |  |
| 3501 | M | 75 | OSCC | Tongue | 3 | 0 | _ |  |
| 3502 | F | 67 | OSCC | Retromolar | 4 | 0 | _ |  |
| 3505 | M | 82 | OSCC | Buccal | 1 | 2b | Y |  |
| 3510 | M | 65 | OSCC | AFOM | 4 | 0 | _ |  |

**Table 3: Primary HNSCC and matching adjacent normal mucosa samples used in pyrosequencing methylation and gene expression analyses**

**SUPPLEMENTARY DATA S2 –** **Cumulative distribution of coverage showing the fraction of targeted bases (y-axis) that were covered by at least a certain depth (x-axis) in EXOME SEQUENCING**

**
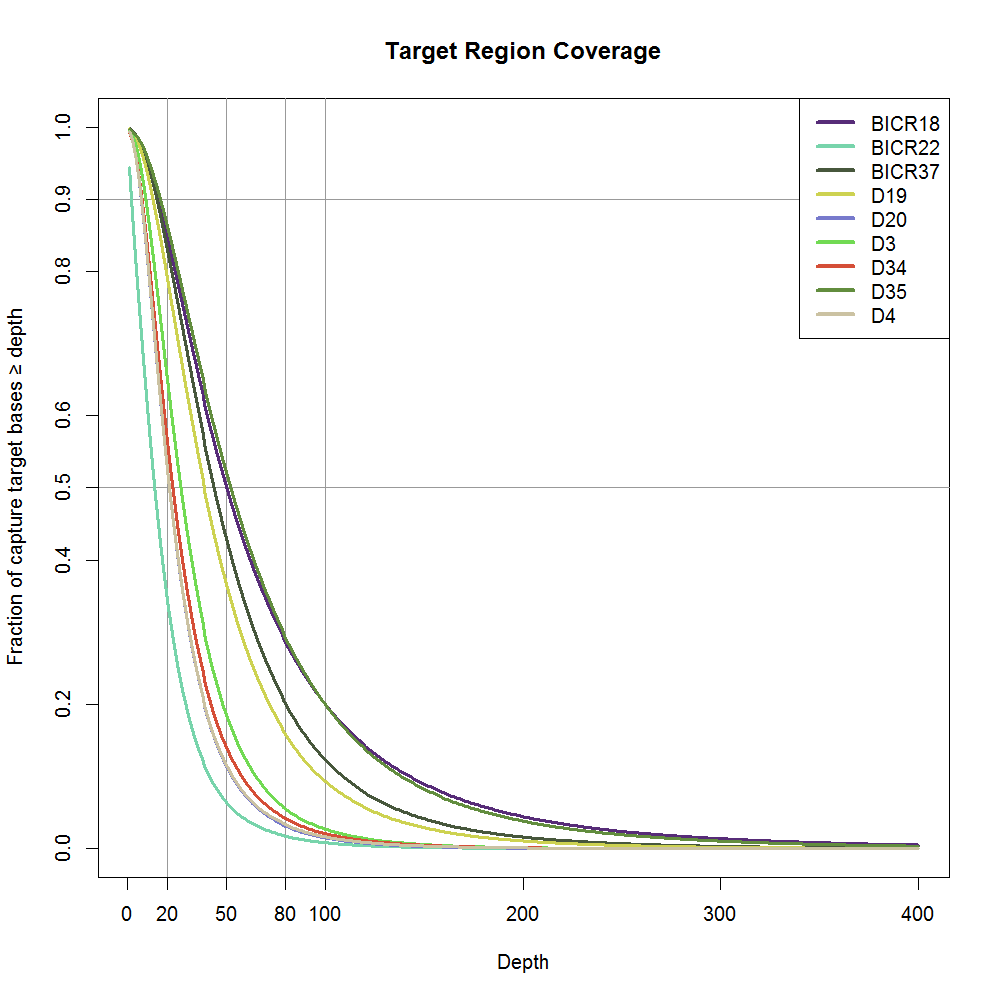

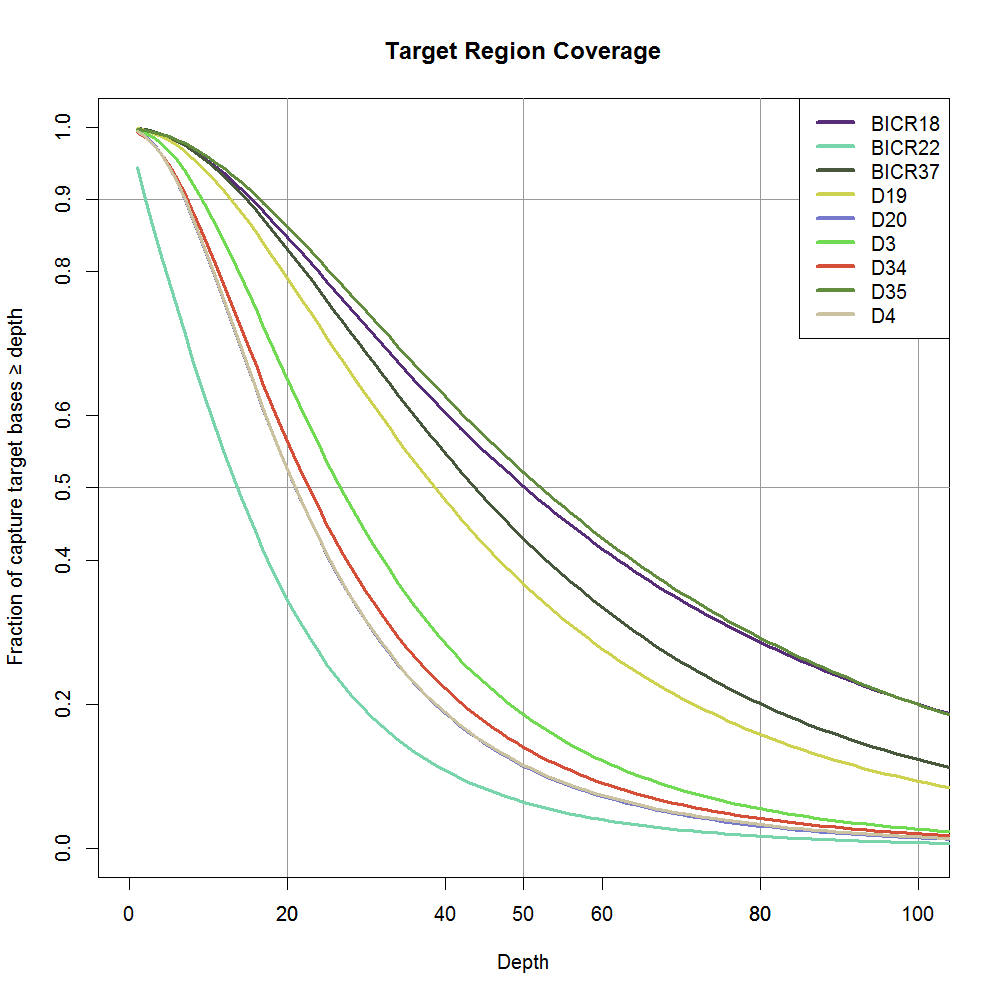
**

Cumulative distribution of coverage showing the fraction of targeted bases (y-axis) that were covered by at least a certain depth (x-axis). Left panel shows the full plot and the right panel shows an expanded plot to show x100 depth coverage.

**SUPPLEMENTARY DATA S3 –** **Cumulative distribution of coverage showing the fraction of targeted bases (y-axis) that were covered by at least a certain depth (x-axis) in haloplex SEQUENCING**

**
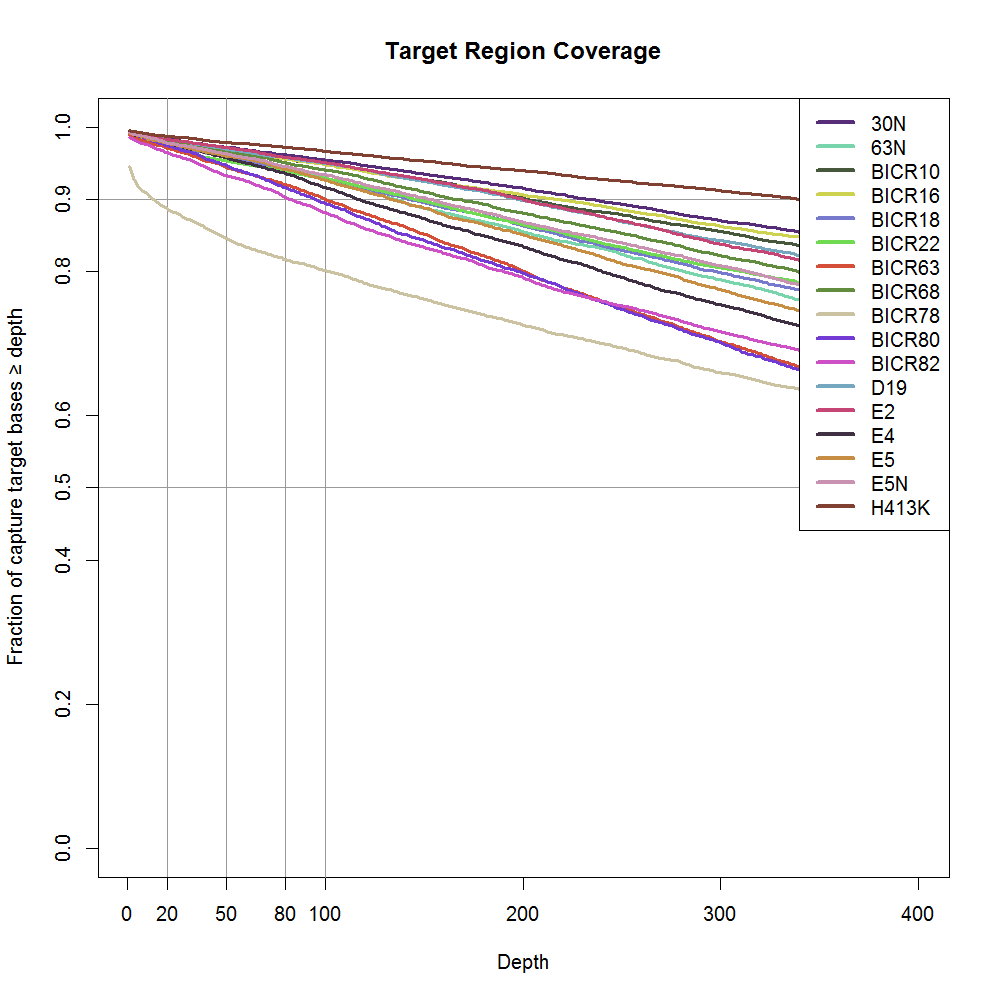

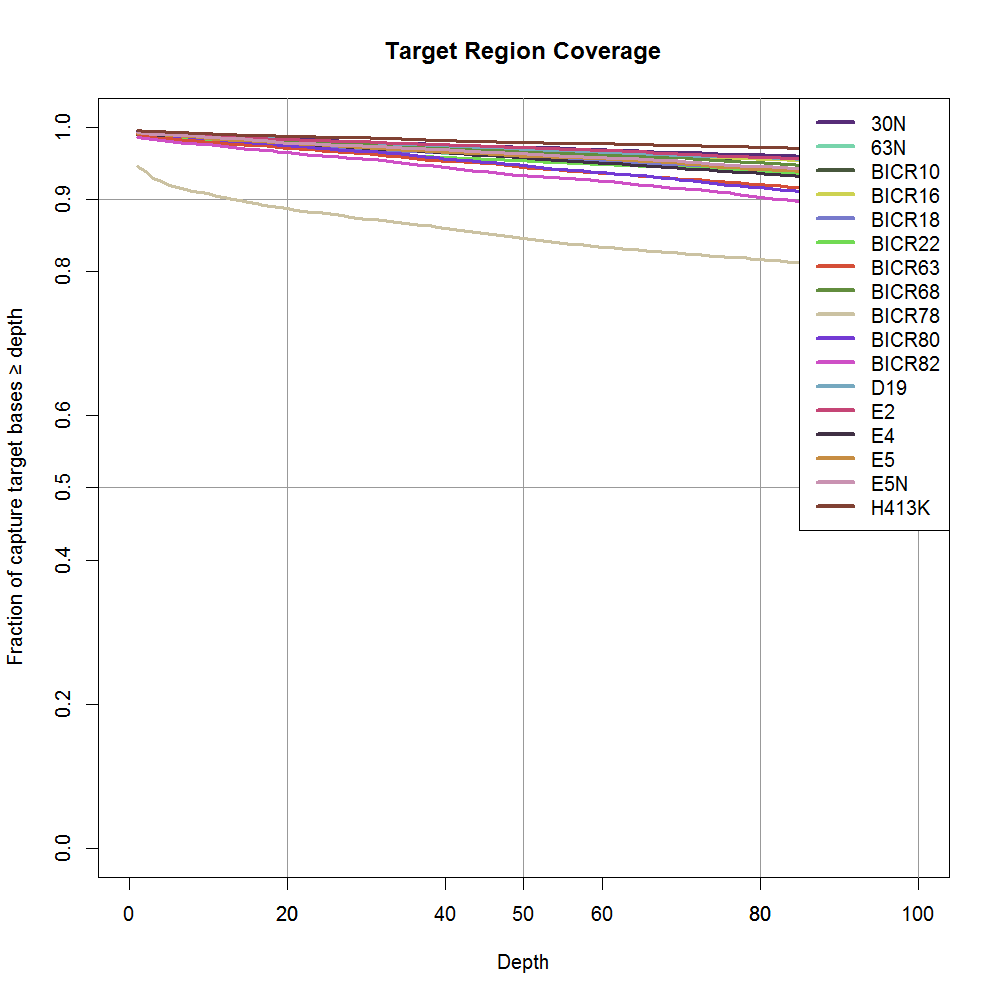
**

Sequence capture efficiency of HaloPlex sequencing panel. Left: The cumulative distribution of coverage showing the fraction of targeted bases (y-axis) that were covered by at least a certain depth (x-axis) is shown. Right: Zoomed-in 0-100 fold coverage range is shown in order to illustrate that with the exception of sample BICR78, all samples were covered at 80x for 90% of the interrogated sequences.

**SUPPLEMENTARY DATA S4– FILTERING OF SEQUENCE VARIANTS DETECTED BY EXOME AND HALOPLEX CAPTURE**

| **Capture** | **Unfiltered** | **ExAC filtering** | **Germline filtering** | **Coding variants** | **Unique variants** | **No High GDI genes** |
| --- | --- | --- | --- | --- | --- | --- |
| Exomes | 461689 | 157440 | 56520 | 9701 | 5085 | 4493 |
| HaloPlex | 5042 | 2008 | 1189 | 248 | 63 | 53 |
| Total | 466731 | 159448 | 57709 | 9949 | 5148 | 4546 |


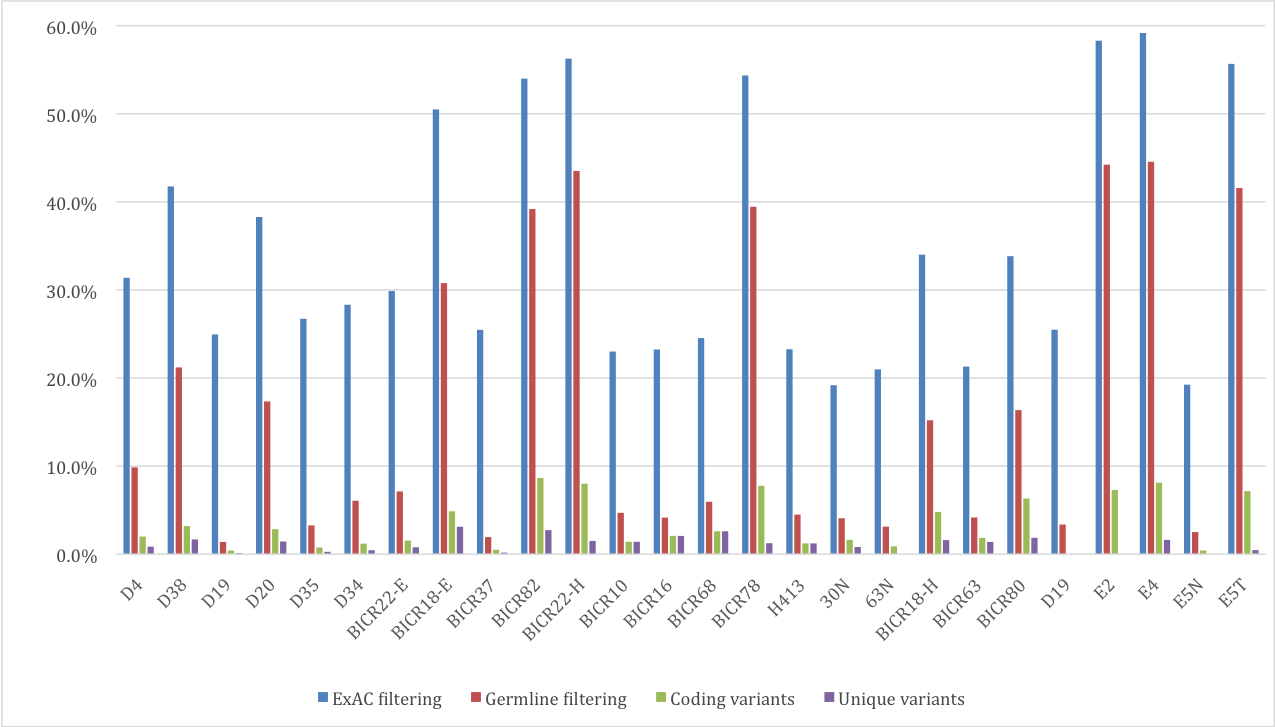


**SUPPLEMENTARY DATA S6 - COMPARISION OF SCNAs IN TWO HNSCC PANELS**


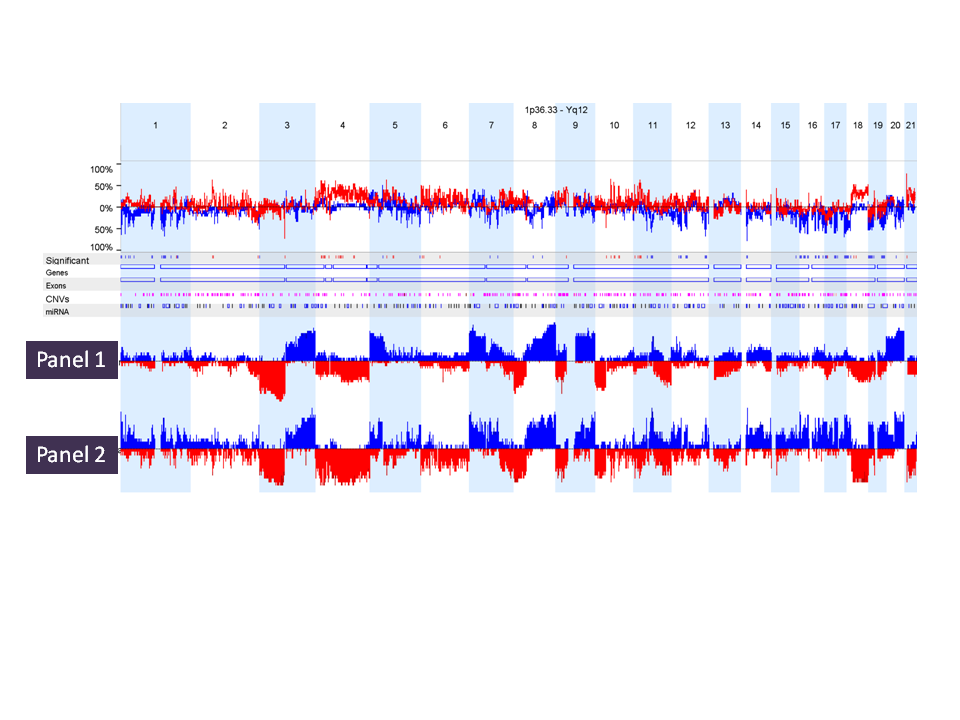


Karyograms of two HNSCC panels used in the study. Panel 1 was subject to SNP array analyses and Panel 2 was subject to Array CGH. Although there were some small differences overall the data were comparable and were merged for further analyses

**SUPPLEMENTARY DATA S7 – SUBTRACTION KARYOGRAM OF MORTAL PPOLS AND MATCHING FIBROBLASTS**


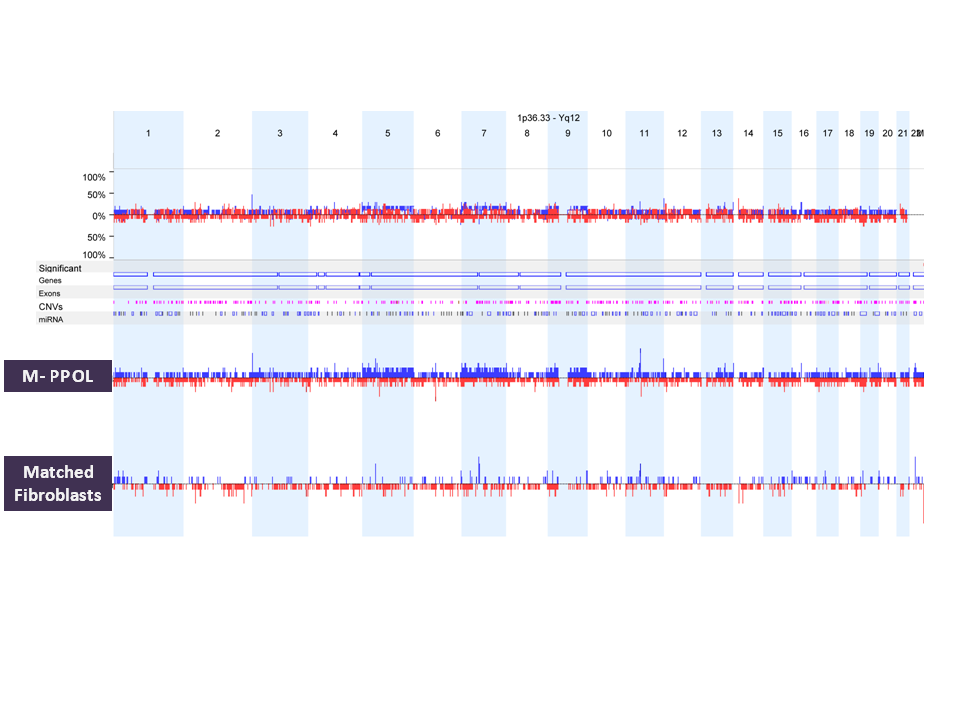


**Subtraction karyogram showing lack of significant difference in copy number gains (blue) and losses (red) between mortal PPOLs (M-PPOLs) and matched fibroblasts.**

**supplementary data S8 – ImmORTAL PPOL CELL LINES SCNA DENSITY PLOTS FOR CHROMOSOMES 3, 8, 9 AND 20**





*





*





*





**Immortal PPOL SCNA density plots for chromosomes 3, 8, 9 and 20 (from top to bottom). D19, D20 and D35 are derived from progressive PPOL lesions. Asterisk (*) below density plots for chromosomes 3, 8 and 9 indicate positions of *FHIT*, *CSMD1* and *CDKN2A* respectively.**

**SUPPLEMENTARY DATA S9 - Hierarchical Cluster Analysis of Immortal HNSCC Cell Lines For High Copy Number SCNA - Association With Lymph Node Metastases**

**
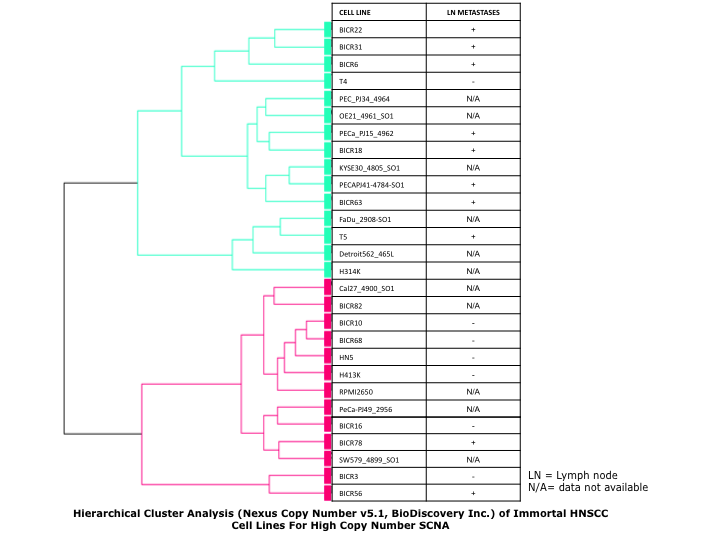
**

**SUPPLEMENTARY DATA S10 - GISTIC PEAK REGIONS IN PPOLS AND HNSCC CELL LINES**

**Table 1. GISTIC peak regions in PPOLs with varying significance thresholds** (Q bound value)

| **Gene Symbol** | **Chromosome** | **Start** | **End** | **Length** | **Q<0.05** |
| --- | --- | --- | --- | --- | --- |
| NCKAP5 | chr2 | 133145841 | 134042501 | 896661 |  |
| SORBS2 | chr4 | 186743591 | 187114864 | 371274 |  |
| TLR3 | chr4 | 187227302 | 187243246 | 15945 |  |
| FAM149A | chr4 | 187302988 | 187330811 | 27824 |  |
| CYP4V2 | chr4 | 187349667 | 187371611 | 21945 |  |
| KLKB1 | chr4 | 187385665 | 187416619 | 30955 |  |
| F11 | chr4 | 187424111 | 187447829 | 23719 |  |
| MTNR1A | chr4 | 187691802 | 187713531 | 21730 |  |
| FAT1 | chr4 | 187745930 | 187881981 | 136052 |  |
| PLEKHG4B | chr5 | 193372 | 243087 | 49716 |  |
| LRRC14B | chr5 | 244625 | 248468 | 3844 |  |
| CCDC127 | chr5 | 257874 | 271297 | 13424 |  |
| SDHA | chr5 | 271355 | 309814 | 38460 |  |
| CSMD1 | chr8 | 2780282 | 4839736 | 2059455 |  |
| ZNF16 | chr8 | 146126547 | 146147078 | 20532 |  |
| ZNF252 | chr8 | 146169778 | 146199089 | 29312 |  |
| TMED10P | chr8 | 146191054 | 146195086 | 4033 |  |
| C8orf77 | chr8 | 146199000 | 146202235 | 3236 |  |
| C8orf33 | chr8 | 146248627 | 146252220 | 3594 |  |
| C9orf53 | chr9 | 21957137 | 21957752 | 616 |  |
| CDKN2A | chr9 | 21957750 | 21984490 | 26741 |  |
| CDKN2B | chr9 | 21992901 | 21999312 | 6412 |  |
| CDKN2BAS | chr9 | 21984789 | 22111091 | 126303 |  |
| DEFB125 | chr20 | 16350 | 25296 | 8947 |  |
| DEFB126 | chr20 | 71251 | 74392 | 3142 |  |
|  |  |  |  |  |  |
| Gene Symbol | Chromosome | Start | End | Length | Q<0.1 |
| FAM110C | chr2 | 28815 | 36588 | 7774 |  |
| NCKAP5 | chr2 | 133145841 | 134042501 | 896661 |  |
| SORBS2 | chr4 | 186743591 | 187114864 | 371274 |  |
| TLR3 | chr4 | 187227302 | 187243246 | 15945 |  |
| FAM149A | chr4 | 187302988 | 187330811 | 27824 |  |
| CYP4V2 | chr4 | 187349667 | 187371611 | 21945 |  |
| KLKB1 | chr4 | 187385665 | 187416619 | 30955 |  |
| F11 | chr4 | 187424111 | 187447829 | 23719 |  |
| MTNR1A | chr4 | 187691802 | 187713531 | 21730 |  |
| FAT1 | chr4 | 187745930 | 187881981 | 136052 |  |
| PLEKHG4B | chr5 | 193372 | 243087 | 49716 |  |
| LRRC14B | chr5 | 244625 | 248468 | 3844 |  |
| CCDC127 | chr5 | 257874 | 271297 | 13424 |  |
| SDHA | chr5 | 271355 | 309814 | 38460 |  |
| CSMD1 | chr8 | 2780282 | 4839736 | 2059455 |  |
| ZNF16 | chr8 | 146126547 | 146147078 | 20532 |  |
| ZNF252 | chr8 | 146169778 | 146199089 | 29312 |  |
| TMED10P | chr8 | 146191054 | 146195086 | 4033 |  |
| C8orf77 | chr8 | 146199000 | 146202235 | 3236 |  |
| C8orf33 | chr8 | 146248627 | 146252220 | 3594 |  |
| C9orf53 | chr9 | 21957137 | 21957752 | 616 |  |
| CDKN2A | chr9 | 21957750 | 21984490 | 26741 |  |
| CDKN2B | chr9 | 21992901 | 21999312 | 6412 |  |
| CDKN2BAS | chr9 | 21984789 | 22111091 | 126303 |  |
| TUBB8 | chr10 | 82827 | 85504 | 2678 |  |
| ZMYND11 | chr10 | 170423 | 290576 | 120154 |  |
| DEFB125 | chr20 | 16350 | 25296 | 8947 |  |
| DEFB126 | chr20 | 71251 | 74392 | 3142 |  |
| NCRNA00176 | chr20 | 62136140 | 62141758 | 5619 |  |
| SOX18 | chr20 | 62149522 | 62151423 | 1902 |  |
| TCEA2 | chr20 | 62158882 | 62174144 | 15263 |  |
| RGS19 | chr20 | 62174978 | 62181768 | 6791 |  |
| C20orf201 | chr20 | 62185176 | 62186156 | 981 |  |
| OPRL1 | chr20 | 62181931 | 62202440 | 20510 |  |
| NPBWR2 | chr20 | 62207626 | 62208628 | 1003 |  |
| MYT1 | chr20 | 62266270 | 62344050 | 77781 |  |
| PCMTD2 | chr20 | 62357491 | 62378023 | 20533 |  |
| S100B | chr21 | 46842958 | 46849463 | 6506 |  |
| PRMT2 | chr21 | 46879954 | 46909291 | 29338 |  |
|  |  |  |  |  |  |
| Gene Symbol | Chromosome | Start | End | Length | Q<0.25 |
| NME7 | chr1 | 167368392 | 167603810 | 235419 |  |
| TDRD5 | chr1 | 177827647 | 177927021 | 99375 |  |
| ZNF648 | chr1 | 180290327 | 180297470 | 7144 |  |
| FAM110C | chr2 | 28815 | 36588 | 7774 |  |
| NCKAP5 | chr2 | 133145841 | 134042501 | 896661 |  |
| FHIT | chr3 | 59710075 | 61212173 | 1502099 |  |
| CCDC50 | chr3 | 192529567 | 192599152 | 69586 |  |
| FAM190A | chr4 | 91267706 | 92742392 | 1474687 |  |
| SORBS2 | chr4 | 186743591 | 187114864 | 371274 |  |
| TLR3 | chr4 | 187227302 | 187243246 | 15945 |  |
| FAM149A | chr4 | 187302988 | 187330811 | 27824 |  |
| CYP4V2 | chr4 | 187349667 | 187371611 | 21945 |  |
| KLKB1 | chr4 | 187385665 | 187416619 | 30955 |  |
| F11 | chr4 | 187424111 | 187447829 | 23719 |  |
| MTNR1A | chr4 | 187691802 | 187713531 | 21730 |  |
| FAT1 | chr4 | 187745930 | 187881981 | 136052 |  |
| PLEKHG4B | chr5 | 193372 | 243087 | 49716 |  |
| LRRC14B | chr5 | 244625 | 248468 | 3844 |  |
| CCDC127 | chr5 | 257874 | 271297 | 13424 |  |
| SDHA | chr5 | 271355 | 309814 | 38460 |  |
| PDE4D | chr5 | 58300622 | 59819682 | 1519061 |  |
| TBP | chr6 | 170705345 | 170723882 | 18538 |  |
| PDCD2 | chr6 | 170728374 | 170735673 | 7300 |  |
| CSMD1 | chr8 | 2780282 | 4839736 | 2059455 |  |
| EYA1 | chr8 | 72272221 | 72437021 | 164801 |  |
| ZNF16 | chr8 | 146126547 | 146147078 | 20532 |  |
| ZNF252 | chr8 | 146169778 | 146199089 | 29312 |  |
| TMED10P | chr8 | 146191054 | 146195086 | 4033 |  |
| C8orf77 | chr8 | 146199000 | 146202235 | 3236 |  |
| C8orf33 | chr8 | 146248627 | 146252220 | 3594 |  |
| PTPRD | chr9 | 8304246 | 10602723 | 2298478 |  |
| C9orf53 | chr9 | 21957137 | 21957752 | 616 |  |
| CDKN2A | chr9 | 21957750 | 21984490 | 26741 |  |
| CDKN2B | chr9 | 21992901 | 21999312 | 6412 |  |
| CDKN2BAS | chr9 | 21984789 | 22111091 | 126303 |  |
| TUBB8 | chr10 | 82827 | 85504 | 2678 |  |
| ZMYND11 | chr10 | 170423 | 290576 | 120154 |  |
| OR4C11 | chr11 | 55127492 | 55128425 | 934 |  |
| OR4P4 | chr11 | 55162409 | 55163348 | 940 |  |
| OR4S2 | chr11 | 55174955 | 55175891 | 937 |  |
| OR4C6 | chr11 | 55189218 | 55190148 | 931 |  |
| FAM138D | chr12 | 18207 | 19673 | 1467 |  |
| IQSEC3 | chr12 | 46309 | 157884 | 111576 |  |
| LOC100133469 | chr14 | 106009499 | 106022574 | 13076 |  |
| NCLN | chr19 | 3136874 | 3160573 | 23700 |  |
| CELF5 | chr19 | 3175700 | 3248073 | 72374 |  |
| DEFB125 | chr20 | 16350 | 25296 | 8947 |  |
| DEFB126 | chr20 | 71251 | 74392 | 3142 |  |
| NCRNA00176 | chr20 | 62136140 | 62141758 | 5619 |  |
| SOX18 | chr20 | 62149522 | 62151423 | 1902 |  |
| TCEA2 | chr20 | 62158882 | 62174144 | 15263 |  |
| RGS19 | chr20 | 62174978 | 62181768 | 6791 |  |
| C20orf201 | chr20 | 62185176 | 62186156 | 981 |  |
| OPRL1 | chr20 | 62181931 | 62202440 | 20510 |  |
| NPBWR2 | chr20 | 62207626 | 62208628 | 1003 |  |
| MYT1 | chr20 | 62266270 | 62344050 | 77781 |  |
| PCMTD2 | chr20 | 62357491 | 62378023 | 20533 |  |
| S100B | chr21 | 46842958 | 46849463 | 6506 |  |
| PRMT2 | chr21 | 46879954 | 46909291 | 29338 |  |

**Table 2. GISTIC peak regions in HNSCCs with varying significance thresholds (Q bound value)**

| Gene Symbol | Chromosome | Start | End | Length | Q<0.05 |
| --- | --- | --- | --- | --- | --- |
| LCE3C | chr1 | 150839761 | 150840186 | 426 |  |
| LRP1B | chr2 | 140705465 | 142605740 | 1900276 |  |
| LOC728323 | chr2 | 242679516 | 242751142 | 71627 |  |
| CNTN4 | chr3 | 2117246 | 3074645 | 957400 |  |
| FHIT | chr3 | 59710075 | 61212173 | 1502099 |  |
| CCDC50 | chr3 | 192529567 | 192599152 | 69586 |  |
| FAM190A | chr4 | 91267706 | 92742392 | 1474687 |  |
| PDZD2 | chr5 | 31834787 | 32146795 | 312009 |  |
| GOLPH3 | chr5 | 32160580 | 32210182 | 49603 |  |
| PDE4D | chr5 | 58300622 | 59819682 | 1519061 |  |
| HLA-DRB5 | chr6 | 32593131 | 32605984 | 12854 |  |
| EGFR | chr7 | 55054218 | 55242525 | 188308 |  |
| GATS | chr7 | 99636215 | 99707791 | 71577 |  |
| SPDYE3 | chr7 | 99743260 | 99757754 | 14495 |  |
| PMS2L1 | chr7 | 99756198 | 99771866 | 15669 |  |
| PILRB | chr7 | 99771672 | 99803388 | 31717 |  |
| PILRA | chr7 | 99809003 | 99835658 | 26656 |  |
| ZCWPW1 | chr7 | 99836430 | 99864238 | 27809 |  |
| MEPCE | chr7 | 99865464 | 99869677 | 4214 |  |
| C7orf47 | chr7 | 99870847 | 99872030 | 1184 |  |
| C7orf61 | chr7 | 99892173 | 99899830 | 7658 |  |
| TSC22D4 | chr7 | 99902077 | 99914838 | 12762 |  |
| C7orf51 | chr7 | 99919485 | 99930358 | 10874 |  |
| AGFG2 | chr7 | 99974769 | 100003779 | 29011 |  |
| SAP25 | chr7 | 100007788 | 100009206 | 1419 |  |
| LRCH4 | chr7 | 100009569 | 100021712 | 12144 |  |
| FBXO24 | chr7 | 100021891 | 100036676 | 14786 |  |
| PCOLCE | chr7 | 100037817 | 100043734 | 5918 |  |
| MOSPD3 | chr7 | 100047660 | 100050936 | 3277 |  |
| TFR2 | chr7 | 100055974 | 100077109 | 21136 |  |
| ACTL6B | chr7 | 100078661 | 100092020 | 13360 |  |
| GNB2 | chr7 | 100109298 | 100114727 | 5430 |  |
| GIGYF1 | chr7 | 100115065 | 100124806 | 9742 |  |
| POP7 | chr7 | 100141611 | 100143059 | 1449 |  |
| EPO | chr7 | 100156358 | 100159259 | 2902 |  |
| ZAN | chr7 | 100169184 | 100233355 | 64172 |  |
| MLL3 | chr7 | 151462942 | 151764023 | 301082 |  |
| CSMD1 | chr8 | 2780282 | 4839736 | 2059455 |  |
| ADAM5P | chr8 | 39291338 | 39379532 | 88195 |  |
| ADAM3A | chr8 | 39427721 | 39499627 | 71907 |  |
| C8orf77 | chr8 | 146199000 | 146202235 | 3236 |  |
| C8orf33 | chr8 | 146248627 | 146252220 | 3594 |  |
| C9orf53 | chr9 | 21957137 | 21957752 | 616 |  |
| CDKN2A | chr9 | 21957750 | 21984490 | 26741 |  |
| NOTCH1 | chr9 | 138508716 | 138560059 | 51344 |  |
| PARD3 | chr10 | 34440102 | 35143929 | 703828 |  |
| CD44 | chr11 | 35116992 | 35210525 | 93534 |  |
| SLC1A2 | chr11 | 35229327 | 35397681 | 168355 |  |
| PAMR1 | chr11 | 35409951 | 35503752 | 93802 |  |
| FJX1 | chr11 | 35596310 | 35598995 | 2686 |  |
| TRIM44 | chr11 | 35640928 | 35787506 | 146579 |  |
| OR4P4 | chr11 | 55162409 | 55163348 | 940 |  |
| OR4S2 | chr11 | 55174955 | 55175891 | 937 |  |
| OR4C6 | chr11 | 55189218 | 55190148 | 931 |  |
| ANO1 | chr11 | 69602055 | 69713299 | 111245 |  |
| FADD | chr11 | 69726916 | 69731156 | 4241 |  |
| MIR548K | chr11 | 69807708 | 69807824 | 117 |  |
| PPFIA1 | chr11 | 69794470 | 69908150 | 113681 |  |
| CTTN | chr11 | 69922259 | 69960338 | 38080 |  |
| SHANK2 | chr11 | 69991609 | 70613456 | 621848 |  |
| PPP2R5E | chr14 | 62911107 | 63079832 | 168726 |  |
| FSCN2 | chr17 | 77110011 | 77114631 | 4621 |  |
| C17orf70 | chr17 | 77117387 | 77129871 | 12485 |  |
| NPLOC4 | chr17 | 77134356 | 77214543 | 80188 |  |
| TSPAN10 | chr17 | 77219753 | 77226184 | 6432 |  |
| PDE6G | chr17 | 77227893 | 77234012 | 6120 |  |
| C17orf90 | chr17 | 77242470 | 77244023 | 1554 |  |
| CCDC137 | chr17 | 77244165 | 77251341 | 7177 |  |
| ARL16 | chr17 | 77258628 | 77261359 | 2732 |  |
| HGS | chr17 | 77261424 | 77279552 | 18129 |  |
| MRPL12 | chr17 | 77280811 | 77284953 | 4143 |  |
| SLC25A10 | chr17 | 77289775 | 77298447 | 8673 |  |
| DYSFIP1 | chr17 | 77384656 | 77386215 | 1560 |  |
| P4HB | chr17 | 77394322 | 77411833 | 17512 |  |
| TCF4 | chr18 | 51040559 | 51406858 | 366300 |  |
| DEFB127 | chr20 | 86185 | 87804 | 1620 |  |
| CHMP4B | chr20 | 31862770 | 31905834 | 43065 |  |
|  |  |  |  |  |  |
| Gene Symbol | Chromosome | Start | End | Length | Q<0.25 |
| LCE3C | chr1 | 150839761 | 150840186 | 426 |  |
| OR2T34 | chr1 | 246803724 | 246804681 | 958 |  |
| OR2T10 | chr1 | 246822753 | 246823692 | 940 |  |
| OR2T11 | chr1 | 246856101 | 246857052 | 952 |  |
| OR2T35 | chr1 | 246868210 | 246869182 | 973 |  |
| LRP1B | chr2 | 140705465 | 142605740 | 1900276 |  |
| PARD3B | chr2 | 205118760 | 206188782 | 1070023 |  |
| LOC728323 | chr2 | 242679516 | 242751142 | 71627 |  |
| CNTN4 | chr3 | 2117246 | 3074645 | 957400 |  |
| SCN5A | chr3 | 38564556 | 38666168 | 101613 |  |
| SCN10A | chr3 | 38713840 | 38810505 | 96666 |  |
| SCN11A | chr3 | 38862263 | 38967056 | 104794 |  |
| FHIT | chr3 | 59710075 | 61212173 | 1502099 |  |
| CCDC50 | chr3 | 192529567 | 192599152 | 69586 |  |
| FAM190A | chr4 | 91267706 | 92742392 | 1474687 |  |
| SORBS2 | chr4 | 186743591 | 187114864 | 371274 |  |
| PDZD2 | chr5 | 31834787 | 32146795 | 312009 |  |
| GOLPH3 | chr5 | 32160580 | 32210182 | 49603 |  |
| PDE4D | chr5 | 58300622 | 59819682 | 1519061 |  |
| NSD1 | chr5 | 176492685 | 176659820 | 167136 |  |
| HLA-H | chr6 | 29963361 | 29966835 | 3475 |  |
| HCG2P7 | chr6 | 29974786 | 29978408 | 3623 |  |
| HCG4P6 | chr6 | 30000347 | 30001407 | 1061 |  |
| HLA-DRB5 | chr6 | 32593131 | 32605984 | 12854 |  |
| EGFR | chr7 | 55054218 | 55242525 | 188308 |  |
| GATS | chr7 | 99636215 | 99707791 | 71577 |  |
| SPDYE3 | chr7 | 99743260 | 99757754 | 14495 |  |
| PMS2L1 | chr7 | 99756198 | 99771866 | 15669 |  |
| PILRB | chr7 | 99771672 | 99803388 | 31717 |  |
| PILRA | chr7 | 99809003 | 99835658 | 26656 |  |
| ZCWPW1 | chr7 | 99836430 | 99864238 | 27809 |  |
| MEPCE | chr7 | 99865464 | 99869677 | 4214 |  |
| C7orf47 | chr7 | 99870847 | 99872030 | 1184 |  |
| C7orf61 | chr7 | 99892173 | 99899830 | 7658 |  |
| TSC22D4 | chr7 | 99902077 | 99914838 | 12762 |  |
| C7orf51 | chr7 | 99919485 | 99930358 | 10874 |  |
| AGFG2 | chr7 | 99974769 | 100003779 | 29011 |  |
| SAP25 | chr7 | 100007788 | 100009206 | 1419 |  |
| LRCH4 | chr7 | 100009569 | 100021712 | 12144 |  |
| FBXO24 | chr7 | 100021891 | 100036676 | 14786 |  |
| PCOLCE | chr7 | 100037817 | 100043734 | 5918 |  |
| MOSPD3 | chr7 | 100047660 | 100050936 | 3277 |  |
| TFR2 | chr7 | 100055974 | 100077109 | 21136 |  |
| ACTL6B | chr7 | 100078661 | 100092020 | 13360 |  |
| GNB2 | chr7 | 100109298 | 100114727 | 5430 |  |
| GIGYF1 | chr7 | 100115065 | 100124806 | 9742 |  |
| POP7 | chr7 | 100141611 | 100143059 | 1449 |  |
| EPO | chr7 | 100156358 | 100159259 | 2902 |  |
| ZAN | chr7 | 100169184 | 100233355 | 64172 |  |
| MLL3 | chr7 | 151462942 | 151764023 | 301082 |  |
| CSMD1 | chr8 | 2780282 | 4839736 | 2059455 |  |
| ADAM5P | chr8 | 39291338 | 39379532 | 88195 |  |
| ADAM3A | chr8 | 39427721 | 39499627 | 71907 |  |
| C8orf77 | chr8 | 146199000 | 146202235 | 3236 |  |
| C8orf33 | chr8 | 146248627 | 146252220 | 3594 |  |
| GLIS3 | chr9 | 3814127 | 4290035 | 475909 |  |
| C9orf53 | chr9 | 21957137 | 21957752 | 616 |  |
| CDKN2A | chr9 | 21957750 | 21984490 | 26741 |  |
| NOTCH1 | chr9 | 138508716 | 138560059 | 51344 |  |
| PARD3 | chr10 | 34440102 | 35143929 | 703828 |  |
| OR52N5 | chr11 | 5755441 | 5756473 | 1033 |  |
| CD44 | chr11 | 35116992 | 35210525 | 93534 |  |
| SLC1A2 | chr11 | 35229327 | 35397681 | 168355 |  |
| PAMR1 | chr11 | 35409951 | 35503752 | 93802 |  |
| FJX1 | chr11 | 35596310 | 35598995 | 2686 |  |
| TRIM44 | chr11 | 35640928 | 35787506 | 146579 |  |
| OR4P4 | chr11 | 55162409 | 55163348 | 940 |  |
| OR4S2 | chr11 | 55174955 | 55175891 | 937 |  |
| OR4C6 | chr11 | 55189218 | 55190148 | 931 |  |
| ANO1 | chr11 | 69602055 | 69713299 | 111245 |  |
| FADD | chr11 | 69726916 | 69731156 | 4241 |  |
| MIR548K | chr11 | 69807708 | 69807824 | 117 |  |
| PPFIA1 | chr11 | 69794470 | 69908150 | 113681 |  |
| CTTN | chr11 | 69922259 | 69960338 | 38080 |  |
| SHANK2 | chr11 | 69991609 | 70613456 | 621848 |  |
| TRPC6 | chr11 | 100827504 | 100959869 | 132366 |  |
| ANGPTL5 | chr11 | 101266614 | 101292463 | 25850 |  |
| KIAA1377 | chr11 | 101290955 | 101377003 | 86049 |  |
| C11orf70 | chr11 | 101423408 | 101459356 | 35949 |  |
| YAP1 | chr11 | 101486419 | 101609364 | 122946 |  |
| BIRC3 | chr11 | 101693403 | 101713675 | 20273 |  |
| BIRC2 | chr11 | 101723175 | 101754611 | 31437 |  |
| TMEM123 | chr11 | 101772265 | 101828985 | 56721 |  |
| MMP7 | chr11 | 101896448 | 101906688 | 10241 |  |
| MMP20 | chr11 | 101952775 | 102001273 | 48499 |  |
| MMP27 | chr11 | 102067624 | 102081678 | 14055 |  |
| MMP8 | chr11 | 102087735 | 102100895 | 13161 |  |
| DDX12 | chr12 | 9461554 | 9492035 | 30482 |  |
| PPP2R5E | chr14 | 62911107 | 63079832 | 168726 |  |
| LOC727924 | chr15 | 19779395 | 19872450 | 93056 |  |
| SOLH | chr16 | 517856 | 544637 | 26782 |  |
| C16orf11 | chr16 | 550422 | 555530 | 5109 |  |
| NHLRC4 | chr16 | 557032 | 559496 | 2465 |  |
| PIGQ | chr16 | 560004 | 574110 | 14107 |  |
| RAB40C | chr16 | 579357 | 619274 | 39918 |  |
| WFIKKN1 | chr16 | 621012 | 624117 | 3106 |  |
| C16orf13 | chr16 | 624429 | 626348 | 1920 |  |
| FAM195A | chr16 | 631849 | 638475 | 6627 |  |
| WDR90 | chr16 | 639363 | 657830 | 18468 |  |
| RHOT2 | chr16 | 658133 | 664172 | 6040 |  |
| RHBDL1 | chr16 | 666075 | 668268 | 2194 |  |
| STUB1 | chr16 | 670115 | 672769 | 2655 |  |
| JMJD8 | chr16 | 671667 | 674440 | 2774 |  |
| WDR24 | chr16 | 674702 | 680401 | 5700 |  |
| EDC4 | chr16 | 66464499 | 66475907 | 11409 |  |
| NRN1L | chr16 | 66476281 | 66477772 | 1492 |  |
| PSKH1 | chr16 | 66484675 | 66521082 | 36408 |  |
| CTRL | chr16 | 66520973 | 66523266 | 2294 |  |
| PSMB10 | chr16 | 66525907 | 66528254 | 2348 |  |
| LCAT | chr16 | 66531287 | 66535516 | 4230 |  |
| SLC12A4 | chr16 | 66534877 | 66560098 | 25222 |  |
| DPEP3 | chr16 | 66567066 | 66571953 | 4888 |  |
| DPEP2 | chr16 | 66578795 | 66590865 | 12071 |  |
| DDX28 | chr16 | 66612679 | 66615271 | 2593 |  |
| DUS2L | chr16 | 66614704 | 66670685 | 55982 |  |
| WWOX | chr16 | 76691051 | 77804065 | 1113015 |  |
| FAM57A | chr17 | 582596 | 592825 | 10230 |  |
| GEMIN4 | chr17 | 594410 | 602251 | 7842 |  |
| ELP2P | chr17 | 602649 | 605326 | 2678 |  |
| GLOD4 | chr17 | 609299 | 632321 | 23023 |  |
| RNMTL1 | chr17 | 632262 | 642491 | 10230 |  |
| TRPV1 | chr17 | 3415489 | 3459454 | 43966 |  |
| SHPK | chr17 | 3458304 | 3486365 | 28062 |  |
| CTNS | chr17 | 3486510 | 3513146 | 26637 |  |
| FSCN2 | chr17 | 77110011 | 77114631 | 4621 |  |
| C17orf70 | chr17 | 77117387 | 77129871 | 12485 |  |
| NPLOC4 | chr17 | 77134356 | 77214543 | 80188 |  |
| TSPAN10 | chr17 | 77219753 | 77226184 | 6432 |  |
| PDE6G | chr17 | 77227893 | 77234012 | 6120 |  |
| C17orf90 | chr17 | 77242470 | 77244023 | 1554 |  |
| CCDC137 | chr17 | 77244165 | 77251341 | 7177 |  |
| ARL16 | chr17 | 77258628 | 77261359 | 2732 |  |
| HGS | chr17 | 77261424 | 77279552 | 18129 |  |
| MRPL12 | chr17 | 77280811 | 77284953 | 4143 |  |
| SLC25A10 | chr17 | 77289775 | 77298447 | 8673 |  |
| DYSFIP1 | chr17 | 77384656 | 77386215 | 1560 |  |
| P4HB | chr17 | 77394322 | 77411833 | 17512 |  |
| ADCYAP1 | chr18 | 894943 | 902173 | 7231 |  |
| FAM38B | chr18 | 10660243 | 11138761 | 478519 |  |
| TCF4 | chr18 | 51040559 | 51406858 | 366300 |  |
| DEFB127 | chr20 | 86185 | 87804 | 1620 |  |
| TMEM90B | chr20 | 24397834 | 24595167 | 197334 |  |
| CHMP4B | chr20 | 31862770 | 31905834 | 43065 |  |
| NCRNA00160 | chr21 | 35017974 | 35031349 | 13376 |  |
| RUNX1 | chr21 | 35081967 | 35343465 | 261499 |  |
| C21orf96 | chr21 | 35332104 | 35333593 | 1490 |  |
| Sep-05 | chr22 | 18081986 | 18092297 | 10312 |  |
| GP1BB | chr22 | 18091065 | 18092297 | 1233 |  |
| TBX1 | chr22 | 18124225 | 18151112 | 26888 |  |
| GNB1L | chr22 | 18155933 | 18222462 | 66530 |  |
| APOBEC3A | chr22 | 37683472 | 37689134 | 5663 |  |
| SPRY3 | chrY | 57509844 | 57524511 | 14668 |  |
| VAMP7 | chrY | 57623340 | 57685826 | 62487 |  |
| IL9R | chrY | 57739639 | 57752876 | 13238 |  |

**Table 3. GISTIC peak regions in LN-ve HNSCCs with Q bound value<0.25**

| **Gene Symbol** | **Chromosome** | **Start** | **End** | **Length** | **Q<0.25** |
| --- | --- | --- | --- | --- | --- |
| LCE3D | chr1 | 150818483 | 150819604 | 1122 |  |
| LCE3C | chr1 | 150839761 | 150840186 | 426 |  |
| LCE3B | chr1 | 150852910 | 150853198 | 289 |  |
| VSTM2A | chr7 | 54577512 | 54604442 | 26931 |  |
| SEC61G | chr7 | 54787433 | 54794433 | 7001 |  |
| EGFR | chr7 | 55054218 | 55242525 | 188308 |  |
| PDK4 | chr7 | 95050744 | 95063861 | 13118 |  |
| DYNC1I1 | chr7 | 95239753 | 95565671 | 325919 |  |
| MIR661 | chr8 | 145091346 | 145091435 | 90 |  |
| PLEC | chr8 | 145061308 | 145122901 | 61594 |  |
| PARP10 | chr8 | 145123307 | 145132623 | 9317 |  |
| GRINA | chr8 | 145136213 | 145139571 | 3359 |  |
| SPATC1 | chr8 | 145158569 | 145174002 | 15434 |  |
| OPLAH | chr8 | 145178154 | 145187572 | 9419 |  |
| EXOSC4 | chr8 | 145205509 | 145207539 | 2031 |  |
| GPAA1 | chr8 | 145209511 | 145213107 | 3597 |  |
| CYC1 | chr8 | 145221947 | 145224416 | 2470 |  |
| SHARPIN | chr8 | 145225523 | 145231128 | 5606 |  |
| MAF1 | chr8 | 145231292 | 145234503 | 3212 |  |
| KIAA1875 | chr8 | 145234616 | 145245206 | 10591 |  |
| C8orf30A | chr8 | 145264659 | 145267608 | 2950 |  |
| HEATR7A | chr8 | 145274906 | 145388831 | 113926 |  |
| SCXA | chr8 | 145393504 | 145395033 | 1530 |  |
| SCXB | chr8 | 145393504 | 145395033 | 1530 |  |
| C8orf30A | chr8 | 145408687 | 145411636 | 2950 |  |
| SCXA | chr8 | 145461410 | 145462939 | 1530 |  |
| SCXB | chr8 | 145461410 | 145462939 | 1530 |  |
| BOP1 | chr8 | 145456863 | 145485928 | 29066 |  |
| HSF1 | chr8 | 145486077 | 145509193 | 23117 |  |
| DGAT1 | chr8 | 145509054 | 145521375 | 12322 |  |
| SCRT1 | chr8 | 145525261 | 145530751 | 5491 |  |
| C8ORFK29 | chr8 | 145547694 | 145549313 | 1620 |  |
| FBXL6 | chr8 | 145549898 | 145552940 | 3043 |  |
| GPR172A | chr8 | 145553032 | 145555754 | 2723 |  |
| ADCK5 | chr8 | 145568538 | 145589261 | 20724 |  |
| MIR939 | chr8 | 145590171 | 145590253 | 83 |  |
| MIR1234 | chr8 | 145596283 | 145596367 | 85 |  |
| CPSF1 | chr8 | 145589253 | 145605541 | 16289 |  |
| SLC39A4 | chr8 | 145608605 | 145613081 | 4477 |  |
| VPS28 | chr8 | 145619807 | 145624735 | 4929 |  |
| NFKBIL2 | chr8 | 145624970 | 145640620 | 15651 |  |
| CYHR1 | chr8 | 145646122 | 145661839 | 15718 |  |
| EGFL7 | chr9 | 138677197 | 138686951 | 9755 |  |
| MIR126 | chr9 | 138684874 | 138684958 | 85 |  |
| AGPAT2 | chr9 | 138687415 | 138701732 | 14318 |  |
| FAM69B | chr9 | 138726844 | 138738990 | 12147 |  |
| SIRPA | chr20 | 1822812 | 1868540 | 45729 |  |
| GATA5 | chr20 | 60471947 | 60484421 | 12475 |  |
| C20orf200 | chr20 | 60551882 | 60559213 | 7332 |  |
| VAMP7 | chrY | 57623340 | 57685826 | 62487 |  |
| IL9R | chrY | 57739639 | 57752876 | 13238 |  |

**Table 4. GISTIC peak regions in LN+ve HNSCCs with Q bound value<0.25**

| **Gene Symbol** | **Chromosome** | **Start** | **End** | **Length** | **Q,0.25** |
| --- | --- | --- | --- | --- | --- |
| LCE3C | chr1 | 150839761 | 150840186 | 426 |  |
| LRP1B | chr2 | 140705465 | 142605740 | 1900276 |  |
| PARD3B | chr2 | 205118760 | 206188782 | 1070023 |  |
| LOC728323 | chr2 | 242679516 | 242751142 | 71627 |  |
| CNTN4 | chr3 | 2117246 | 3074645 | 957400 |  |
| SCN5A | chr3 | 38564556 | 38666168 | 101613 |  |
| SCN10A | chr3 | 38713840 | 38810505 | 96666 |  |
| SCN11A | chr3 | 38862263 | 38967056 | 104794 |  |
| SFMBT1 | chr3 | 52913666 | 53055110 | 141445 |  |
| CCDC50 | chr3 | 192529567 | 192599152 | 69586 |  |
| SEC24B | chr4 | 110574419 | 110681064 | 106646 |  |
| PDZD2 | chr5 | 31834787 | 32146795 | 312009 |  |
| NSD1 | chr5 | 176492685 | 176659820 | 167136 |  |
| HLA-DRB5 | chr6 | 32593131 | 32605984 | 12854 |  |
| HLA-DRB6 | chr6 | 32628467 | 32635757 | 7291 |  |
| HLA-DRB1 | chr6 | 32654524 | 32665540 | 11017 |  |
| MICALL2 | chr7 | 1440520 | 1465635 | 25116 |  |
| INTS1 | chr7 | 1476438 | 1510544 | 34107 |  |
| MAFK | chr7 | 1536893 | 1549205 | 12313 |  |
| TMEM184A | chr7 | 1548396 | 1562592 | 14197 |  |
| PSMG3 | chr7 | 1573494 | 1576194 | 2701 |  |
| KIAA1908 | chr7 | 1576234 | 1595785 | 19552 |  |
| GATS | chr7 | 99636215 | 99707791 | 71577 |  |
| SPDYE3 | chr7 | 99743260 | 99757754 | 14495 |  |
| PMS2L1 | chr7 | 99756198 | 99771866 | 15669 |  |
| PILRB | chr7 | 99771672 | 99803388 | 31717 |  |
| PILRA | chr7 | 99809003 | 99835658 | 26656 |  |
| ZCWPW1 | chr7 | 99836430 | 99864238 | 27809 |  |
| MEPCE | chr7 | 99865464 | 99869677 | 4214 |  |
| C7orf47 | chr7 | 99870847 | 99872030 | 1184 |  |
| C7orf61 | chr7 | 99892173 | 99899830 | 7658 |  |
| TSC22D4 | chr7 | 99902077 | 99914838 | 12762 |  |
| C7orf51 | chr7 | 99919485 | 99930358 | 10874 |  |
| AGFG2 | chr7 | 99974769 | 100003779 | 29011 |  |
| SAP25 | chr7 | 100007788 | 100009206 | 1419 |  |
| LRCH4 | chr7 | 100009569 | 100021712 | 12144 |  |
| FBXO24 | chr7 | 100021891 | 100036676 | 14786 |  |
| PCOLCE | chr7 | 100037817 | 100043734 | 5918 |  |
| MOSPD3 | chr7 | 100047660 | 100050936 | 3277 |  |
| TFR2 | chr7 | 100055974 | 100077109 | 21136 |  |
| ACTL6B | chr7 | 100078661 | 100092020 | 13360 |  |
| GNB2 | chr7 | 100109298 | 100114727 | 5430 |  |
| GIGYF1 | chr7 | 100115065 | 100124806 | 9742 |  |
| POP7 | chr7 | 100141611 | 100143059 | 1449 |  |
| EPO | chr7 | 100156358 | 100159259 | 2902 |  |
| ZAN | chr7 | 100169184 | 100233355 | 64172 |  |
| EPHB4 | chr7 | 100238122 | 100263079 | 24958 |  |
| MLL3 | chr7 | 151462942 | 151764023 | 301082 |  |
| CSMD1 | chr8 | 2780282 | 4839736 | 2059455 |  |
| ADAM5P | chr8 | 39291338 | 39379532 | 88195 |  |
| ADAM3A | chr8 | 39427721 | 39499627 | 71907 |  |
| MIR661 | chr8 | 145091346 | 145091435 | 90 |  |
| PLEC | chr8 | 145061308 | 145122901 | 61594 |  |
| PARP10 | chr8 | 145123307 | 145132623 | 9317 |  |
| GRINA | chr8 | 145136213 | 145139571 | 3359 |  |
| SPATC1 | chr8 | 145158569 | 145174002 | 15434 |  |
| OPLAH | chr8 | 145178154 | 145187572 | 9419 |  |
| EXOSC4 | chr8 | 145205509 | 145207539 | 2031 |  |
| GPAA1 | chr8 | 145209511 | 145213107 | 3597 |  |
| CYC1 | chr8 | 145221947 | 145224416 | 2470 |  |
| SHARPIN | chr8 | 145225523 | 145231128 | 5606 |  |
| MAF1 | chr8 | 145231292 | 145234503 | 3212 |  |
| KIAA1875 | chr8 | 145234616 | 145245206 | 10591 |  |
| C8orf30A | chr8 | 145264659 | 145267608 | 2950 |  |
| HEATR7A | chr8 | 145274906 | 145388831 | 113926 |  |
| SCXA | chr8 | 145393504 | 145395033 | 1530 |  |
| SCXB | chr8 | 145393504 | 145395033 | 1530 |  |
| C8orf30A | chr8 | 145408687 | 145411636 | 2950 |  |
| GLIS3 | chr9 | 3814127 | 4290035 | 475909 |  |
| C9orf53 | chr9 | 21957137 | 21957752 | 616 |  |
| CDKN2A | chr9 | 21957750 | 21984490 | 26741 |  |
| NOTCH1 | chr9 | 138508716 | 138560059 | 51344 |  |
| MYEOV | chr11 | 68818197 | 68821330 | 3134 |  |
| CCND1 | chr11 | 69165053 | 69178423 | 13371 |  |
| ORAOV1 | chr11 | 69189512 | 69199346 | 9835 |  |
| FGF19 | chr11 | 69222186 | 69228287 | 6102 |  |
| FGF4 | chr11 | 69296977 | 69299352 | 2376 |  |
| FGF3 | chr11 | 69333916 | 69343129 | 9214 |  |
| ANO1 | chr11 | 69602055 | 69713299 | 111245 |  |
| FADD | chr11 | 69726916 | 69731156 | 4241 |  |
| MIR548K | chr11 | 69807708 | 69807824 | 117 |  |
| PPFIA1 | chr11 | 69794470 | 69908150 | 113681 |  |
| CTTN | chr11 | 69922259 | 69960338 | 38080 |  |
| SHANK2 | chr11 | 69991609 | 70613456 | 621848 |  |
| TRPC6 | chr11 | 100827504 | 100959869 | 132366 |  |
| ANGPTL5 | chr11 | 101266614 | 101292463 | 25850 |  |
| KIAA1377 | chr11 | 101290955 | 101377003 | 86049 |  |
| C11orf70 | chr11 | 101423408 | 101459356 | 35949 |  |
| YAP1 | chr11 | 101486419 | 101609364 | 122946 |  |
| BIRC3 | chr11 | 101693403 | 101713675 | 20273 |  |
| BIRC2 | chr11 | 101723175 | 101754611 | 31437 |  |
| TMEM123 | chr11 | 101772265 | 101828985 | 56721 |  |
| MMP7 | chr11 | 101896448 | 101906688 | 10241 |  |
| MMP20 | chr11 | 101952775 | 102001273 | 48499 |  |
| MMP27 | chr11 | 102067624 | 102081678 | 14055 |  |
| MMP8 | chr11 | 102087735 | 102100895 | 13161 |  |
| CACNA1C | chr12 | 2032676 | 2677376 | 644701 |  |
| RPSAP52 | chr12 | 64438069 | 64507021 | 68953 |  |
| HMGA2 | chr12 | 64504506 | 64646338 | 141833 |  |
| GPHN | chr14 | 66043877 | 66718278 | 674402 |  |
| FAM71D | chr14 | 66725898 | 66765020 | 39123 |  |
| MPP5 | chr14 | 66777773 | 66872289 | 94517 |  |
| SEC14L1 | chr17 | 72648599 | 72724774 | 76176 |  |
| FAM38B | chr18 | 10660243 | 11138761 | 478519 |  |
| RPRD1A | chr18 | 31823789 | 31901371 | 77583 |  |
| SLC39A6 | chr18 | 31942491 | 31963355 | 20865 |  |
| ELP2 | chr18 | 31963884 | 32008605 | 44722 |  |
| MOCOS | chr18 | 32021477 | 32102683 | 81207 |  |
| FHOD3 | chr18 | 32131699 | 32614016 | 482318 |  |
| RIT2 | chr18 | 38577189 | 38949655 | 372467 |  |
| SYT4 | chr18 | 39101854 | 39111613 | 9760 |  |
| DEFB125 | chr20 | 16350 | 25296 | 8947 |  |
| DEFB126 | chr20 | 71251 | 74392 | 3142 |  |
| DEFB127 | chr20 | 86185 | 87804 | 1620 |  |
| NTSR1 | chr20 | 60810633 | 60864568 | 53936 |  |
| GSTTP1 | chr22 | 22670594 | 22677258 | 6665 |  |
| LOC391322 | chr22 | 22703116 | 22704043 | 928 |  |
| GSTT1 | chr22 | 22706138 | 22714284 | 8147 |  |
| GSTTP2 | chr22 | 22715937 | 22731899 | 15963 |  |

**SUPPLEMENTARY DATA S11 - copy number gains and losses of the top ranked genes in top ranked peaks regions of focal deletions identified by Beroukhim et al., 2010**

Table 1. Single and homozygous copy number loss of the top ranked genes in top ranked peaks regions of focal deletions identified by Beroukhim et al., 2010 in a panel of 3131 cell lines. Only genes showing >30% frequency of hemizygous deletions in any one group and/or any homozygous deletions are shown. ^1^Peak rank from Beroukhim et al., 2010; ^2^top rank gene within the peak; ^3^includes cell lines for which lymph node metastases status was not available. Genes indicated in bold are those for which expression array data was available from Hunter et al. 2006. *Show correlation of SCNA/LOH with gene expression with nominal significance (p<0.05). **Show correlation of CAN/LOH gene expression after correction for multiple testing (FDR<0.05).

Table 2. Amplification of the top ranked genes in top ranked peaks regions of focal amplifications identified by Beroukhim et al., 2010 in a panel of 3131 cell lines. Only genes showing >30% frequency of low copy number (<2) amplification in any one group and/or any high copy number gain are shown. ^1^Peak rank from Beroukhim et al., 2010; ^2^top rank gene within the peak; ^3^includes cell lines for which lymph node metastases status was not available. Genes indicated in bold are those for which expression array data was available from Hunter et al. 2006. *Show correlation of SCNA/LOH with gene expression with nominal significance (p<0.05). **Show correlation of CAN/LOH gene expression after correction for multiple testing (FDR<0.05).

|  |  |  |  | **Single Copy Loss**  **Frequency (%)** | | | | **Homozygous Loss**  **Frequency (%)** | | | |
| --- | --- | --- | --- | --- | --- | --- | --- | --- | --- | --- | --- |
|  |  |  |  | **PMOL** | **OSCC** | | | **PMOL** | **OSCC** | | |
| **Rank^1^** | **Chromosome**  **Band** | **Known**  **Target** | **GRAIL Top Target^2^** | **N=7** | **LN+**  **N=10** | **LN-**  **N=7** | **All^3^**  **N=28** | **N=7** | **LN+**  **N=10** | **LN-**  **N=7** | **All^3^**  **N=28** |
| **1** | 9p21.3 | *CDKN2A/B* | ***CDKN2A****** | 0 | 20 | 29 | 29 | 29 | 70 | 43 | 50 |
| **2** | 3p14.2 | *FHIT* | ***FHIT*** | 71 | 100 | 71 | 86 | 14 | 20 | 29 | 32 |
| **3** | 16q23.1 | *WWOX* | ***WWOX*** | 29 | 40 | 29 | 32 | 0 | 0 | 14 | 7 |
| **4** | 9p24.1 | *PTPRD* | ***PTPRD*** | 43 | 60 | 57 | 61 | 0 | 30 | 14 | 25 |
| **7** | 13q14.2 | *RB1* | ***RB1****** | 14 | 40 | 29 | 32 | 0 | 0 | 0 | 0 |
| **8** | 2q22.1 | *LRP1B* | ***LRP1B*** | 29 | 50 | 57 | 50 | 0 | 20 | 29 | 29 |
| **9** | 4q35.2 |  | *FRG2* | 29 | 40 | 29 | 32 | 0 | 0 | 0 | 0 |
| **10** | 5q11.2 | *PDE4D* | *PLK2*  *PDE4D* | 14  57 | 10  20 | 0  29 | 13.7  29 | 0  0 | 0  0 | 0  29 | 4  14 |
| **12** | 7q34 | *TRB* | ***PRSS1*** | 0 | 20 | 14 | 25 | 0 | 0 | 0 | 0 |
| **14** | 19p13.3 |  | ***GZMM***  ***THEG***  ***PPAP2C******  *C19orf20* | 29  29  29  29 | 40  30  20  40 | 14  14  14  0 | 25  25  21  21 | 0  0  0  0 | 0  0  0  0 | 0  0  0  0 | 0  0  0  0 |
| **16** | 8p23.2 | *CSMD1* | *CSMD1* | 71 | 70 | 86 | 75 | 29 | 20 | 14 | 18 |
| **19** | 18q23 |  | *PARD6G* | 14 | 60 | 14 | 50 | 0 | 0 | 0 | 4 |
| **23** | 11q25 | *OPCML NTM* | ***OPCML***  *NTM* | 14  14 | 70  70 | 29  29 | 57  57 | 0  0 | 10  10 | 0  0 | 11  11 |
| **24** | 13q12.11 |  | *LATS2* | 14 | 40 | 29 | 32 | 0 | 0 | 0 | 0 |
| **25** | 22q13.33 |  | *TUBGCP6* | 14 | 30 | 14 | 14 | 0 | 0 | 0 | 4 |
| **30** | 8p23.3 |  | *ZNF596* | 14 | 60 | 57 | 54 | 0 | 0 | 0 | 4 |
| **34** | 11q23.1 | *ATM* | ***CADM1***  ***ATM*** | 14  14 | 70  60 | 43  29 | 61  43 | 0  0 | 10  0 | 0  0 | 11  0 |
| **35** | 9p24.3 |  | *FOXD4* | 14 | 40 | 14 | 36 | 0 | 0 | 0 | 0 |
| **40** | 3p26.3 |  | ***CHL1*** | 57 | 80 | 43 | 57 | 0 | 10 | 0 | 11 |
| **47** | 8p11.22 |  | ***ZMAT4***  ***C8orf4*** | 43  43 | 60  50 | 43  43 | 47  43 | 0  0 | 0  0 | 0  0 | 4  4 |
| **49** | 10p15.3 |  | *TUBB8* | 0 | 40 | 29 | 50 | 0 | 0 | 0 | 0 |
| **50** | 1p31.1 | *NEGR1* | ***NEGR1*** | 14 | 40 | 0 | 29 | 0 | 0 | 0 | 4 |
| **51** | 13q31.3 | *GPC6* | ***DCT***  *GPC6* | 14  14 | 20  40 | 14  14 | 18  29 | 0  0 | 0  0 | 0  0 | 0  0 |
| **61** | 7q35 | *CNTNAP2* | ***CNTNAP2****** | 0 | 40 | 29 | 46 | 0 | 0 | 0 | 4 |
| **62** | 14q32.12 |  | *PRIMA1* | 0 | 30 | 14 | 18 | 0 | 0 | 0 | 0 |
| **66** | 19p12 |  | ***ZNF492***  *ZNF99* | 29  14 | 50  50 | 14  14 | 36  40 | 0  0 | 0  0 | 0  0 | 0  0 |
| **67** | 12q23.1 | *ANKS1B* | ***ANKS1B*** | 14 | 30 | 14 | 29 | 0 | 0 | 0 | 4 |
|  | 4p16.3 |  | ***ZNF141*** | 14 | 40 | 14 | 36 | 0 | 0 | 0 | 0 |
| **68** | 8p21.2 |  | ***DPYSL2******  ***STMN4*** | 57  57 | 50  50 | 71  57 | 61  57 | 0  0 | 10  0 | 0  0 | 4  0 |
| **74** | 11q14.1 | *DLG2* | *TMEM126A*  *CCDC89*  ***CCDC90B****** | 14  14  14 | 50  50  50 | 14  14  0 | 32  32  29 | 0  0  0 | 0  0  0 | 0  0  0 | 0  0  0 |
| **79** | 21q22.2 | *DSCAM*  *ERG* | ***DSCAM***  ***ERG*** | 14  0 | 50  40 | 29  14 | 46  36 | 0  0 | 0  0 | 0  0 | 0  0 |
| **80** | 18q21.2 | *SMAD4*  *DCC* | ***SMAD4******  ***DCC*** | 14  29 | 80  60 | 14  29 | 54  54 | 0  0 | 0  20 | 0  0 | 7  21 |

**Table 1**

|  | | | | | **Low Copy Number Gain**  **Frequency (%)** | | | | **High Copy Number Gain**  **Frequency (%)** | | | |
| --- | --- | --- | --- | --- | --- | --- | --- | --- | --- | --- | --- | --- |
|  |  |  |  |  | **PMOL** | **OSCC** | | | **PMOL** | **OSCC** | | |
| **Rank^1^** |  | **Chromosome**  **band** | **Known**  **target** | **GRAIL Top**  **Target^2^** | **N=7** | **LN+**  **N=10** | **LN-**  **N=7** | **All^3^**  **N=28** | **N=7** | **LN+**  **N=10** | **LN-**  **N=7** | **All^3^**  **N=28** |
| **1** |  | 8q24.21 | *MYC* | ***MYC****** | 43 | 100 | 71 | 75 | 0 | 0 | 0 | 4 |
| **2** |  | 11q13.2 | *CCND1* | *ORAOV1* | 0  0 | 20  20 | 14  14 | 29  29 | 0  0 | 60  60 | 0  0 | 36  36 |
| **5** |  | 14q13.3 | *NKX2-1* | ***NKX2-1*** | 14 | 60 | 0 | 36 | 0 | 0 | 0 | 0 |
| **7** |  | 7p11.2 | *EGFR* | ***EGFR******* | 29 | 30 | 71 | 46 | 0 | 0 | 14 | 14 |
| **13** |  | 12q15 |  | *LRRC10* | 0 | 20 | 14 | 11 | 0 | 10 | 0 | 4 |
| **14** |  | 12q14.3 | *HMGA2* | ***HMGA2****** | 14 | 40 | 14 | 18 | 0 | 10 | 0 | 4 |
| **16** |  | 5p15.33 | *TERT* | ***TERT*** | 29 | 60 | 57 | 54 | 0 | 0 | 0 | 0 |
| **17** |  | 3q26.2 | *PRKCI*** | ***PRKCI******* | 14 | 80 | 43 | 64 | 0 | 20 | 0 | 7 |
| **19** |  | 20q13.2 |  | ***ZNF217******* | 43 | 60 | 29 | 57 | 0 | 0 | 0 | 0 |
| **26** |  | 11q14.1 |  | ***GAB2*** | 0 | 20 | 14 | 29 | 0 | 20 | 0 | 7 |
| **27** |  | 20q13.33 |  | ***BIRC7****** | 43 | 60 | 43 | 61 | 0 | 0 | 0 | 0 |
| **30** |  | 8q21.13 |  | *ZNF704*  *ZBTB10* | 43  43 | 70  70 | 29  29 | 57  54 | 0  0 | 0  0 | 0  0 | 0  0 |
| **33** |  | 20q11.21 | *BCL2L1* | ***ID1***  ***BCL2L1******* | 57  71 | 80  90 | 29  29 | 61  68 | 0  0 | 0  0 | 0  0 | 0  0 |
| **39** |  | 17q25.1 |  | ***GRB2****** | 14 | 40 | 14 | 32 | 0 | 0 | 0 | 0 |
| **43** |  | 8q24.11 |  | ***NOV*** | 43 | 90 | 57 | 71 | 0 | 0 | 0 | 0 |
| **49** |  | 3q28 | *PIK3CA* | ***PIK3CA******* | 14 | 90 | 43 | 71 | 0 | 10 | 0 | 4 |
| **50** |  | 1p36.33 |  | ***TP73*** | 0 | 30 | 14 | 0 | 0 | 0 | 0 | 0 |
| **54** |  | 8q22.3 |  | *RRM2B* | 43 | 80 | 43 | 61 | 0 | 0 | 0 | 0 |
| **67** |  | 8q24.3 |  | ***PTP4A3***  *MAFA*  *PARP10* | 43  43  57 | 100  90  90 | 71  71  57 | 82  75  79 | 0  0  0 | 0  0  10 | 0  0  14 | 0  0  7 |

**Table 2**

**SUPPLEMENTARY DATA S12 - FREQUENCY OF COPY NUMBER ALTERATIONS OF INTOGEN-DERIVED CANCER DRIVER GENES IN PPOLS and HNSCC CELL LINES**

The following tables show frequency of SCNAs in genes identified as cancer drivers through IntOgen analyses (version 2014.12; [http://www.intogen.org](http://www.intogen.org/)).

Abbreviations:

CN- copy number

PPOL- potentially premalignant oral lesions

HNSCC- head and neck squamous cell carcinoma

LN +VE- Lymph node metastases positive

LN-VE- Lymph node metastases negative

Table 1: Cancer drivers showing low copy number gains (≤ 2) ordered by frequency in LN+ve HNSCC cell lines with minimum frequency of 40%

Table 2: Cancer drivers showing low copy number gains (≤2) ordered by frequency in LN-ve HNSCC cell lines with minimum frequency of 40%

Table 3: Cancer drivers showing high copy number gains (>2) ordered by frequency in LN+ve HNSCC cell lines with minimum frequency of 10%

Table 4: Cancer drivers showing high copy number gains (>2) ordered by frequency in LN-ve HNSCC cell lines with minimum frequency of 10%

Table 5: Cancer drivers showing hemizygous loss ordered by frequency in LN+ve HNSCC cell lines with minimum frequency of 40%

Table 6: Cancer drivers showing hemizygous loss ordered by frequency in LN-ve HNSCC cell lines with minimum frequency of 40%

Table 7: Cancer drivers showing homozygous loss ordered by frequency in LN+ve HNSCC cell lines.

**Table 1: Cancer drivers showing low copy number gains (≤ 2) ordered by frequency in LN+ve HNSCC cell lines with minimum frequency of 40%**

| **TERM** | **PPOL**  **%** | **LN-VE**  **%** | **LN +VE**  **%** | **ALL HNSCC**  **%** |
| --- | --- | --- | --- | --- |
| MECOM | 28.57 | 42.86 | 100 | 71.43 |
| MYC | 42.86 | 71.43 | 100 | 75 |
| NDRG1 | 42.86 | 57.14 | 100 | 71.43 |
| PIK3CA | 14.29 | 42.86 | 90 | 71.43 |
| FXR1 | 28.57 | 42.86 | 90 | 71.43 |
| TBL1XR1 | 14.29 | 42.86 | 90 | 67.86 |
| MUC20 | 28.57 | 57.14 | 90 | 71.43 |
| EIF4G1 | 14.29 | 42.86 | 90 | 71.43 |
| RAD21 | 42.86 | 57.14 | 90 | 71.43 |
| DLG1 | 28.57 | 57.14 | 90 | 67.86 |
| RFC4 | 14.29 | 42.86 | 90 | 67.86 |
| EIF4A2 | 14.29 | 42.86 | 90 | 67.86 |
| TFDP2 | 14.29 | 28.57 | 80 | 53.57 |
| ARFGEF2 | 57.14 | 57.14 | 80 | 71.43 |
| STK4 | 57.14 | 57.14 | 80 | 71.43 |
| CHD6 | 57.14 | 42.86 | 80 | 67.86 |
| RASA2 | 14.29 | 28.57 | 80 | 53.57 |
| XRN1 | 14.29 | 28.57 | 70 | 50 |
| PABPC1 | 42.86 | 57.14 | 70 | 64.29 |
| ARFGEF1 | 42.86 | 42.86 | 70 | 60.71 |
| PIK3CB | 14.29 | 28.57 | 70 | 50 |
| CRNKL1 | 71.43 | 14.29 | 70 | 46.43 |
| ATR | 14.29 | 28.57 | 60 | 46.43 |
| STAG1 | 14.29 | 14.29 | 60 | 42.86 |
| PSMA6 | 14.29 | 0 | 60 | 35.71 |
| TRIO | 42.86 | 57.14 | 60 | 60.71 |
| CCT5 | 28.57 | 57.14 | 60 | 53.57 |
| GNAS | 71.43 | 42.86 | 60 | 60.71 |
| PLCG1 | 71.43 | 42.86 | 60 | 60.71 |
| DHX35 | 28.57 | 42.86 | 60 | 57.14 |
| PLCB1 | 71.43 | 28.57 | 60 | 46.43 |
| FOXA2 | 71.43 | 28.57 | 60 | 42.86 |
| PLXNA1 | 42.86 | 28.57 | 60 | 46.43 |
| NCK1 | 14.29 | 14.29 | 60 | 42.86 |
| MAX | 0 | 14.29 | 50 | 35.71 |
| MLH3 | 0 | 14.29 | 50 | 35.71 |
| SOS2 | 14.29 | 0 | 50 | 32.14 |
| GNG2 | 0 | 0 | 50 | 32.14 |
| ZFP36L1 | 0 | 14.29 | 50 | 42.86 |
| ARID4A | 14.29 | 0 | 50 | 32.14 |
| MAP3K11 | 0 | 28.57 | 50 | 39.29 |
| CHD9 | 14.29 | 0 | 50 | 28.57 |
| FOXA1 | 14.29 | 0 | 50 | 28.57 |
| HDAC9 | 42.86 | 85.71 | 50 | 60.71 |
| CSNK2A1 | 57.14 | 28.57 | 50 | 42.86 |
| ASXL1 | 28.57 | 28.57 | 50 | 53.57 |
| CTTN | 0 | 14.29 | 40 | 28.57 |
| MEN1 | 0 | 14.29 | 40 | 39.29 |
| AHR | 42.86 | 85.71 | 40 | 53.57 |
| ACTG1 | 14.29 | 28.57 | 40 | 42.86 |
| STIP1 | 0 | 14.29 | 40 | 35.71 |
| GNAI1 | 42.86 | 42.86 | 40 | 39.29 |
| TNPO1 | 42.86 | 14.29 | 40 | 17.86 |
| CEP290 | 28.57 | 14.29 | 40 | 17.86 |
| EFTUD2 | 0 | 14.29 | 40 | 32.14 |
| NFATC4 | 0 | 0 | 40 | 28.57 |
| SPTAN1 | 14.29 | 85.71 | 40 | 60.71 |
| ARFGAP1 | 14.29 | 42.86 | 40 | 53.57 |
| SRGAP1 | 14.29 | 14.29 | 40 | 17.86 |
| KALRN | 14.29 | 14.29 | 40 | 28.57 |

**Table 2: Cancer drivers showing low copy number gains (≤2) ordered by frequency in LN-ve HNSCC cell lines with minimum frequency of 40%**

| **TERM** | **PPOL**  **%** | **LN-VE**  **%** | **LN +VE**  **%** | **ALL HNSCC**  **%** |
| --- | --- | --- | --- | --- |
| HDAC9 | 42.86 | 85.71 | 50 | 60.71 |
| AHR | 42.86 | 85.71 | 40 | 53.57 |
| SPTAN1 | 14.29 | 85.71 | 40 | 60.71 |
| RAC1 | 14.29 | 85.71 | 30 | 64.29 |
| SYK | 14.29 | 85.71 | 20 | 50 |
| PTCH1 | 0 | 85.71 | 20 | 46.43 |
| SVEP1 | 14.29 | 85.71 | 20 | 50 |
| RGS3 | 0 | 85.71 | 20 | 46.43 |
| MYC | 42.86 | 71.43 | 100 | 75 |
| ACTB | 28.57 | 71.43 | 30 | 53.57 |
| PCSK5 | 0 | 71.43 | 30 | 46.43 |
| TSC1 | 0 | 71.43 | 30 | 50 |
| EGFR | 28.57 | 71.43 | 30 | 46.43 |
| TJP2 | 14.29 | 71.43 | 30 | 42.86 |
| ADCY1 | 14.29 | 71.43 | 30 | 46.43 |
| PPP6C | 0 | 71.43 | 30 | 50 |
| SCAI | 14.29 | 71.43 | 30 | 50 |
| NOTCH1 | 14.29 | 71.43 | 20 | 53.57 |
| KLF4 | 14.29 | 71.43 | 20 | 42.86 |
| RAD23B | 0 | 71.43 | 20 | 42.86 |
| NTRK2 | 0 | 71.43 | 10 | 39.29 |
| NDRG1 | 42.86 | 57.14 | 100 | 71.43 |
| MUC20 | 28.57 | 57.14 | 90 | 71.43 |
| RAD21 | 42.86 | 57.14 | 90 | 71.43 |
| DLG1 | 28.57 | 57.14 | 90 | 67.86 |
| ARFGEF2 | 57.14 | 57.14 | 80 | 71.43 |
| STK4 | 57.14 | 57.14 | 80 | 71.43 |
| PABPC1 | 42.86 | 57.14 | 70 | 64.29 |
| TRIO | 42.86 | 57.14 | 60 | 60.71 |
| CCT5 | 28.57 | 57.14 | 60 | 53.57 |
| PTGS1 | 0 | 57.14 | 30 | 42.86 |
| MECOM | 28.57 | 42.86 | 100 | 71.43 |
| PIK3CA | 14.29 | 42.86 | 90 | 71.43 |
| FXR1 | 28.57 | 42.86 | 90 | 71.43 |
| TBL1XR1 | 14.29 | 42.86 | 90 | 67.86 |
| EIF4G1 | 14.29 | 42.86 | 90 | 71.43 |
| RFC4 | 14.29 | 42.86 | 90 | 67.86 |
| EIF4A2 | 14.29 | 42.86 | 90 | 67.86 |
| CHD6 | 57.14 | 42.86 | 80 | 67.86 |
| ARFGEF1 | 42.86 | 42.86 | 70 | 60.71 |
| GNAS | 71.43 | 42.86 | 60 | 60.71 |
| PLCG1 | 71.43 | 42.86 | 60 | 60.71 |
| DHX35 | 28.57 | 42.86 | 60 | 57.14 |
| GNAI1 | 42.86 | 42.86 | 40 | 39.29 |
| ARFGAP1 | 14.29 | 42.86 | 40 | 53.57 |
| AKAP9 | 0 | 42.86 | 30 | 35.71 |
| CUX1 | 0 | 42.86 | 10 | 35.71 |

**Table 3: Cancer drivers showing high copy number gains (>2) ordered by frequency in LN+ve HNSCC cell lines with minimum frequency of 10%**

| **TERM** | **LN-VE**  **%** | **LN +VE**  **%** | **ALL HNSCC**  **%** |
| --- | --- | --- | --- |
| CCND1 | 0 | 60 | 35.71 |
| CTTN | 0 | 40 | 35.71 |
| XRN1 | 0 | 20 | 7.14 |
| ATR | 0 | 20 | 7.14 |
| NOTCH1 | 0 | 20 | 7.14 |
| ACTB | 0 | 10 | 14.29 |
| INPPL1 | 0 | 10 | 10.71 |
| MAX | 0 | 10 | 7.14 |
| MLH3 | 0 | 10 | 7.14 |
| RAC1 | 0 | 10 | 7.14 |
| PCSK5 | 14.29 | 10 | 7.14 |
| CUX1 | 0 | 10 | 7.14 |
| MECOM | 0 | 10 | 3.57 |
| PIK3CA | 0 | 10 | 3.57 |
| FXR1 | 0 | 10 | 3.57 |
| TBL1XR1 | 0 | 10 | 3.57 |
| TFDP2 | 0 | 10 | 3.57 |
| STAG1 | 0 | 10 | 3.57 |
| SOS2 | 0 | 10 | 3.57 |
| GNG2 | 0 | 10 | 3.57 |
| ZFP36L1 | 0 | 10 | 3.57 |
| ARID4A | 0 | 10 | 3.57 |
| AKAP9 | 0 | 10 | 3.57 |
| TSC1 | 0 | 10 | 3.57 |
| CEBPA | 0 | 10 | 3.57 |
| ZNF814 | 0 | 10 | 3.57 |
| SYK | 0 | 10 | 3.57 |
| PTCH1 | 0 | 10 | 3.57 |
| SRGAP3 | 0 | 10 | 3.57 |
| VHL | 0 | 10 | 3.57 |
| NTRK2 | 0 | 10 | 3.57 |
| BMPR2 | 0 | 10 | 3.57 |
| NUP107 | 0 | 10 | 3.57 |
| MED17 | 0 | 10 | 3.57 |
| XPO1 | 0 | 10 | 3.57 |

**Table 4: Cancer drivers showing high copy number gains (>2) ordered by frequency in LN-ve HNSCC cell lines with minimum frequency of 10%**

| **TERM** | **LN-VE**  **%** | **LN +VE**  **%** | **ALL HNSCC**  **%** |
| --- | --- | --- | --- |
| PCSK5 | 14.29 | 10 | 7.14 |
| EGFR | 14.29 | 0 | 14.29 |
| ERBB2 | 14.29 | 0 | 3.57 |
| KLF4 | 14.29 | 0 | 3.57 |
| RAD23B | 14.29 | 0 | 3.57 |

**Table 5: Cancer drivers showing hemizygous loss ordered by frequency in LN+ve HNSCC cell lines with minimum frequency of 40%**

| **TERM** | **PPOL**  **%** | **LN-VE**  **%** | **LN+VE**  **%** | **ALL HNSCC**  **%** |
| --- | --- | --- | --- | --- |
| FOXP1 | 42.86 | 85.71 | 100 | 89.27 |
| SETD2 | 57.14 | 57.14 | 100 | 75 |
| CTNNB1 | 57.14 | 42.86 | 100 | 75 |
| ITGA9 | 57.14 | 42.86 | 100 | 67.86 |
| MLH1 | 57.14 | 42.86 | 100 | 67.86 |
| ROBO2 | 42.86 | 85.71 | 90 | 82.14 |
| RBM5 | 57.14 | 57.14 | 90 | 67.86 |
| RHOA | 42.86 | 57.14 | 90 | 67.86 |
| CLASP2 | 57.14 | 42.86 | 90 | 64.29 |
| WNT5A | 42.86 | 42.86 | 90 | 67.86 |
| CAPN7 | 57.14 | 28.57 | 90 | 57.14 |
| MYD88 | 42.86 | 28.57 | 90 | 60.71 |
| SMAD4 | 14.29 | 14.29 | 80 | 53.57 |
| NEDD4L | 28.57 | 14.29 | 80 | 57.14 |
| PBRM1 | 57.14 | 57.14 | 80 | 64.29 |
| BAP1 | 57.14 | 57.14 | 80 | 64.29 |
| SRGAP3 | 57.14 | 28.57 | 80 | 53.57 |
| TGFBR2 | 28.57 | 28.57 | 80 | 57.14 |
| TCF4 | 28.57 | 28.57 | 70 | 53.57 |
| SMAD2 | 14.29 | 14.29 | 70 | 53.57 |
| ACAD8 | 14.29 | 28.57 | 60 | 53.57 |
| TET2 | 28.57 | 42.86 | 60 | 64.29 |
| HSPA8 | 14.29 | 42.86 | 60 | 50 |
| VHL | 42.86 | 28.57 | 60 | 46.43 |
| ATM | 14.29 | 28.57 | 60 | 42.86 |
| CASP1 | 0 | 14.29 | 60 | 35.71 |
| MLL | 14.29 | 14.29 | 60 | 42.86 |
| MSR1 | 57.14 | 85.71 | 50 | 57.14 |
| RUNX1 | 0 | 14.29 | 50 | 35.71 |
| FAT1 | 42.86 | 42.86 | 50 | 53.57 |
| PGR | 14.29 | 28.57 | 50 | 35.71 |
| NKX3-1 | 57.14 | 71.43 | 50 | 60.71 |
| BNC2 | 14.29 | 57.14 | 50 | 42.86 |
| GATA3 | 0 | 57.14 | 50 | 64.29 |
| EPC1 | 0 | 57.14 | 50 | 60.71 |
| PSIP1 | 14.29 | 42.86 | 50 | 39.29 |
| CUL2 | 0 | 42.86 | 50 | 57.14 |
| IRF2 | 42.86 | 42.86 | 50 | 46.43 |
| PABPC3 | 14.29 | 28.57 | 50 | 35.71 |
| DHX15 | 14.29 | 14.29 | 50 | 39.29 |
| SEC24D | 0 | 14.29 | 50 | 46.43 |
| MED17 | 14.29 | 0 | 50 | 28.57 |
| RPSAP58 | 28.57 | 0 | 50 | 32.14 |
| PCDH18 | 28.57 | 28.57 | 40 | 46.43 |
| POLR2B | 14.29 | 28.57 | 40 | 42.86 |
| STARD13 | 14.29 | 42.86 | 40 | 32.14 |
| VIM | 0 | 42.86 | 40 | 53.57 |
| LCP1 | 14.29 | 28.57 | 40 | 32.14 |
| RB1 | 14.29 | 28.57 | 40 | 32.14 |
| FRG1 | 28.57 | 28.57 | 40 | 32.14 |
| FLT3 | 28.57 | 28.57 | 40 | 32.14 |
| BRCA2 | 28.57 | 28.57 | 40 | 28.57 |
| WASF3 | 14.29 | 28.57 | 40 | 32.14 |
| CLOCK | 14.29 | 14.29 | 40 | 39.29 |
| KIT | 14.29 | 14.29 | 40 | 39.29 |
| FIP1L1 | 28.57 | 14.29 | 40 | 39.29 |
| G3BP2 | 28.57 | 14.29 | 40 | 46.43 |
| ITSN1 | 14.29 | 14.29 | 40 | 28.57 |
| FBXW7 | 28.57 | 14.29 | 40 | 46.43 |
| HNRPDL | 14.29 | 14.29 | 40 | 46.43 |
| BRWD1 | 14.29 | 14.29 | 40 | 35.71 |
| KDR | 14.29 | 0 | 40 | 35.71 |
| SEC31A | 14.29 | 0 | 40 | 42.86 |

**Table 6: Cancer drivers showing hemizygous loss ordered by frequency in LN-ve HNSCC cell lines with minimum frequency of 40%**

| TERM | PPOL  % | LN-VE  % | LN+VE  % | ALL HNSCC  % |
| --- | --- | --- | --- | --- |
| FOXP1 | 42.86 | 85.71 | 100 | 89.27 |
| ROBO2 | 42.86 | 85.71 | 90 | 82.14 |
| MSR1 | 57.14 | 85.71 | 50 | 57.14 |
| NKX3-1 | 57.14 | 71.43 | 50 | 60.71 |
| ATIC | 0 | 71.43 | 10 | 21.43 |
| SETD2 | 57.14 | 57.14 | 100 | 75 |
| RBM5 | 57.14 | 57.14 | 90 | 67.86 |
| RHOA | 42.86 | 57.14 | 90 | 67.86 |
| PBRM1 | 57.14 | 57.14 | 80 | 64.29 |
| BAP1 | 57.14 | 57.14 | 80 | 64.29 |
| BNC2 | 14.29 | 57.14 | 50 | 42.86 |
| GATA3 | 0 | 57.14 | 50 | 64.29 |
| EPC1 | 0 | 57.14 | 50 | 60.71 |
| FN1 | 0 | 57.14 | 10 | 17.86 |
| IDH1 | 0 | 57.14 | 0 | 14.29 |
| EEF1B2 | 0 | 57.14 | 0 | 14.29 |
| CUL3 | 0 | 57.14 | 0 | 14.29 |
| CTNNB1 | 57.14 | 42.86 | 100 | 75 |
| ITGA9 | 57.14 | 42.86 | 100 | 67.86 |
| MLH1 | 57.14 | 42.86 | 100 | 67.86 |
| CLASP2 | 57.14 | 42.86 | 90 | 64.29 |
| WNT5A | 42.86 | 42.86 | 90 | 67.86 |
| TET2 | 28.57 | 42.86 | 60 | 64.29 |
| HSPA8 | 14.29 | 42.86 | 60 | 50 |
| FAT1 | 42.86 | 42.86 | 50 | 53.57 |
| PSIP1 | 14.29 | 42.86 | 50 | 39.29 |
| CUL2 | 0 | 42.86 | 50 | 57.14 |
| IRF2 | 42.86 | 42.86 | 50 | 46.43 |
| STARD13 | 14.29 | 42.86 | 40 | 32.14 |
| VIM | 0 | 42.86 | 40 | 53.57 |
| KLF6 | 0 | 42.86 | 30 | 46.43 |
| MAP2K4 | 28.57 | 42.86 | 10 | 25 |
| EPHA4 | 0 | 42.86 | 10 | 14.29 |

**Table 7: Cancer drivers showing homozygous loss ordered by frequency in LN+ve HNSCC cell lines.**

| **TERM** | **PPOL**  **%** | **LN -VE**  **%** | **LN+VE**  **%** | **ALL HNSCC**  **%** |
| --- | --- | --- | --- | --- |
| CDKN2A | 28.57 | 42.86 | 70 | 50 |
| MLL3 | 0 | 0 | 20 | 10.71 |
| NSD1 | 0 | 0 | 20 | 7.14 |
| ROBO2 | 0 | 0 | 10 | 14.29 |
| MSR1 | 0 | 0 | 10 | 10.71 |
| ACAD8 | 0 | 0 | 10 | 7.14 |
| ERBB2IP | 0 | 0 | 10 | 7.14 |
| TCF4 | 14.29 | 0 | 0 | 10.71 |
| FOXP1 | 0 | 14.29 | 0 | 7.14 |
| SMAD4 | 0 | 0 | 0 | 7.14 |
| RUNX1 | 0 | 0 | 0 | 7.14 |
| PCDH18 | 0 | 0 | 0 | 7.14 |
| CDC73 | 0 | 0 | 0 | 7.14 |
| NEDD4L | 0 | 0 | 0 | 3.57 |
| FAT1 | 14.29 | 0 | 0 | 3.57 |
| PGR | 0 | 0 | 0 | 3.57 |
| POLR2B | 0 | 0 | 0 | 3.57 |
| WHSC1L1 | 0 | 0 | 0 | 3.57 |
| FGFR1 | 0 | 0 | 0 | 3.57 |
| PLXNB2 | 0 | 0 | 0 | 3.57 |
| MEF2C | 0 | 0 | 0 | 3.57 |
| MAP3K1 | 0 | 0 | 0 | 3.57 |

**SUPPLEMENTARY DATA 13 – DELETIONS IN HNSCC CELL LINES AT THE *CSDM1* LOCUS**





**Deletions in HNSCC cell lines at *CSMD1* locus. Red areas indicate extent of deletions. Dark red/thicker lines indicate regions of homozygous deletions. Blue lines indicate regions of amplification and grey lines a lack of SCNA.**

**Supplementary Data 14 - Modulation of *CSMD1* Expression in Immortal HNSCC Cell Lines**


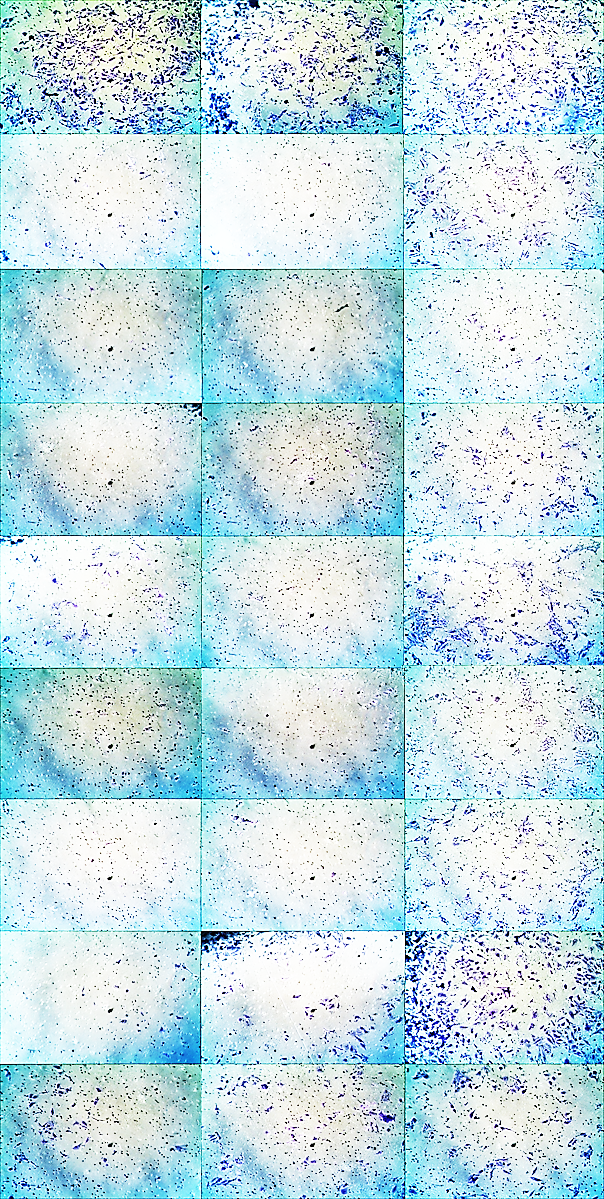


**A**

**B**


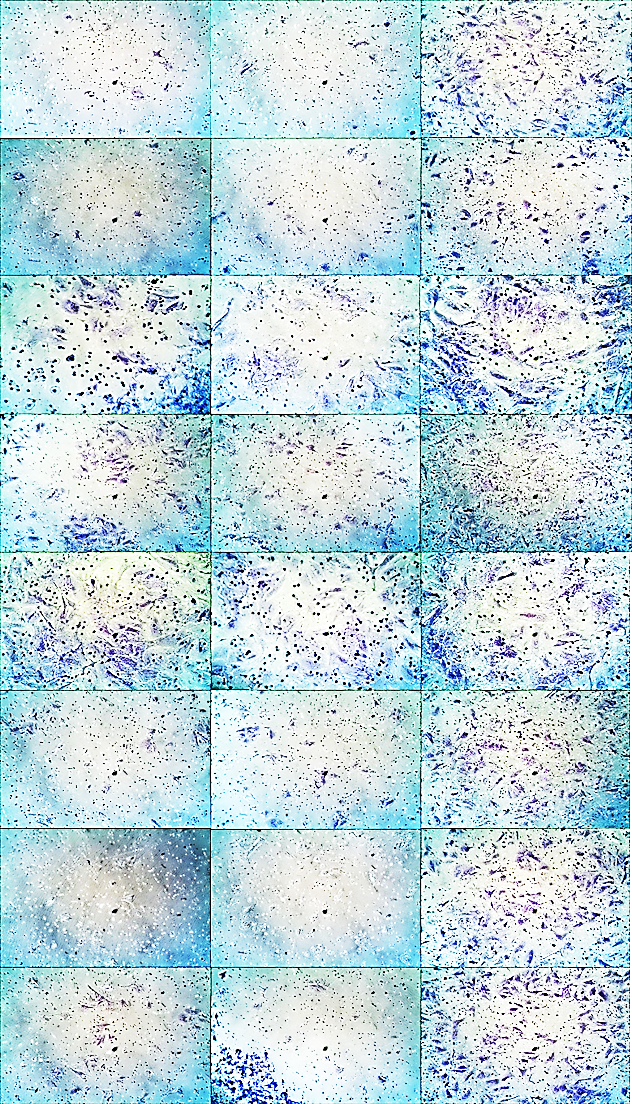


**A. Two trans-well chambers of all *CSMD1*-expressing clones and *CSMD1*-negative H103 parent cell line are displayed together with no matrigel controls. *CSMD1* expression results in a marked decrease of gel invasion (p=5.975x10^-5^). B. Two trans-well chambers of all *CSMD1*-silenced clones and *CSMD1*-expressing BICR16 parent cell line are displayed together with no matrigel controls. *CSMD1* silencing results in a variable marked increase in gel invasion (p=1.822x10^-05^).**

**SUPPLEMENATRY DATA S15 - ANALYSIS OF *NCKAP5***

Deletions in *NCKAP5* in our dataset are shown in Figure 1. Homozygous deletions were present in both immortal PPOL and HNSCC cell lines and the homozygous deletions eliminated one or more exons in 4 out of the 5 cell lines (Fig 1) with the remaining cell line sustaining two intronic deletions. We looked for deletions of *NCKAP5* in our array-CGH dataset of 347 tumour cell lines (unpublished) and in Tumorscape (Release 1.6). In our dataset (not shown), only colorectal cell lines bore homozygous deletions of *NCKAP5* and these were rare (2 of 161 or ~1.2%). Both deletions involved exon 3. In Tumorscape, *NCKAP5* is significantly focally deleted across the entire dataset of 3131 tumours but is not located within a focal peak region of deletion. It is significantly focally deleted in 2 of 14 independent subtypes analyzed in Tumorscape including all epithelial cancers (Q=4.33E6, Frequency=0.12). It is not within the focal peak region of deletion in any of the individual tumour types and deletions are not frequent (<20%), and homozygous deletions are exceedingly rare (<1%). Analyses of variants listed in COSMIC data show few pathological mutations as well as misense mutations predicted to be pathological (Table 1).

We analysed expression of *NCKAP5* transcript in small number of paired tumour and normal HNSCC samples as well as a panel of other tumour types (Figure 2) Marked reduced expression compared to normal tissues was seen in about ~26% of the tumours with remainder showing little change or more commonly overexpression. Analyses of expression using TissueScan Cancer and Normal Cancer Survey 96-I cDNA Arrays (Origene, Maryland, USA) showed reduced expression in tumours of thyroid, lung, liver and kidney (Figure 3).

Thus, *NCKAP5* is unlikely to be frequent target for inactivation in HNSCC but the related pathways may be more significant. NCKAP5 has been shown to interact with NCK1 (STRING Release 9.1), an adaptor protein important in ligand-induced activation of receptor tyrosine kinases and also APC (BioGRID Release 3.3). Our IntOGene (Release 2014.12) analyses showed a low frequency of pan-cancer gain-of-function mutations in *NCK1*. In this study, *NCK1* was within an extended GISTIC region and copy number gains were seen in 60% of LN+ve cell lines with relatively low frequency (~14%) gains in PPOL and LN-ve cell lines.

**Figure 1. Hemizygous and homozygous deletions at *NCKAP5* locus**

BICR10

BICR82

D35

D4


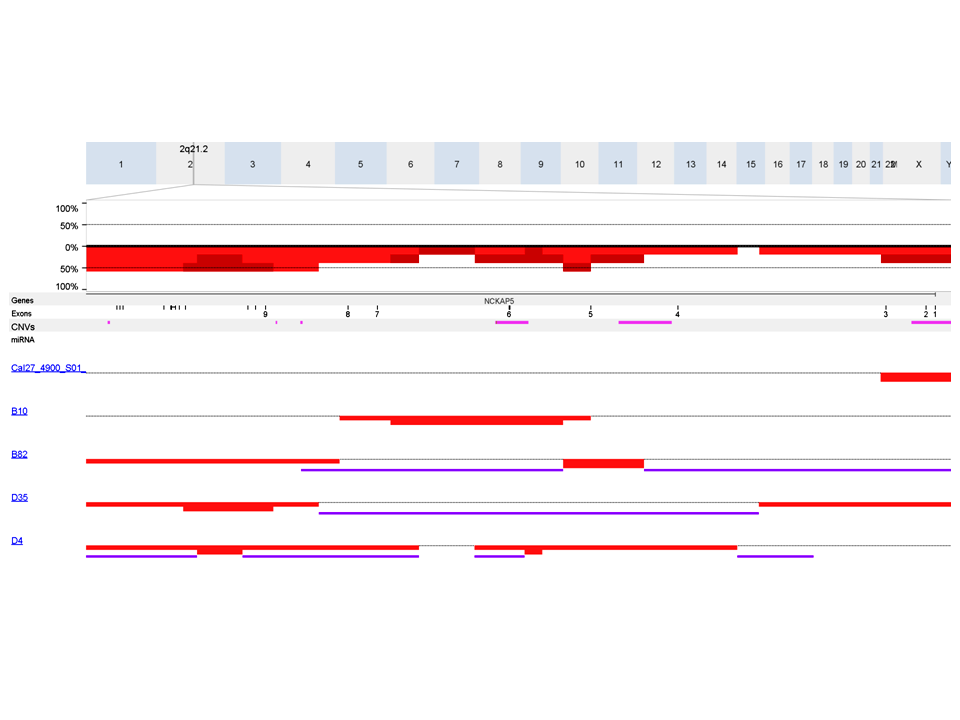


Hemizygous (thin red line) and homozygous deletions (thickred line).

**Table 1. Analysis of *NCKAP5*** mutations listed in COSMIC

| **NCKAP5 (PolyPhen-2)** | | | **PANTHER** | **SIFT** | **Tissue Type** | **Histology** |
| --- | --- | --- | --- | --- | --- | --- |
| **Amino acid position** | **Prediction** | **Score** | P_deleterious_ |  |  |  |
| p.K183R | benign | 0.058 | 0.13865 | TOLERATED | haematopoietic and lymphoid tissue | lymphoid neoplasm |
| p.A198V | benign | 0.032 | 0.72406 | DAMAGING | colon | carcinoma |
| p.P316H | benign | 0.023 | 0.10065 | TOLERATED | colon | carcinoma |
| p.T556M | benign | 0.001 | 0.24661 | TOLERATED | colon | carcinoma |
| p.Q560R | benign | 0.01 | 0.36801 | TOLERATED | Not specified | not specified |
| p.V754A | benign | 0 | 0.09554 | TOLERATED | caecum | carcinoma |
| p.S890G | benign | 0.05 | 0.65865 | DAMAGING | colon | carcinoma |
| p.T1056I | benign | 0.012 | 0.45163 | TOLERATED | Not specified | malignant melanoma |
| p.A1137D | benign | 0.435 | 0.35013 | TOLERATED | rectum | carcinoma |
| p.D1192G | benign | 0.01 | 0.517 | TOLERATED | colon | carcinoma |
| p.I1201V | benign | 0 | 0.19913 | TOLERATED | prostate | carcinoma |
| p.A1329S | benign | 0.101 | 0.09918 | TOLERATED | autonomic ganglia | neuroblastoma |
| p.S1346F | benign | 0.002 | 0.15902 | TOLERATED | caecum | carcinoma |
| p.A1537V | benign | 0.021 | 0.35217 | TOLERATED | rectum | carcinoma |
| p.A1863T | benign | 0.007 | 0.15675 | TOLERATED | colon | carcinoma |
| p.L36P | probably damaging | 1 | 0.61759 | *DAMAGING | colon | carcinoma |
| p.S172R | probably damaging | 0.96 | 0.28558 | TOLERATED | Not specified | not specified |
| p.R212Q | probably damaging | 0.993 | 0.7559 | DAMAGING | colon | carcinoma |
| p.T354M | probably damaging | 1 | 0.6481 | *DAMAGING | colon | carcinoma |
| p.D357N | probably damaging | 1 | 0.41471 | *DAMAGING | skin | malignant melanoma |
| p.S382I | probably damaging | 0.986 | 0.74926 | *DAMAGING | caecum | carcinoma |
| p.G404W | probably damaging | 1 | 0.94376 | *DAMAGING | colon | carcinoma |
| p.T538I | possibly damaging | 0.617 | 0.3067 | TOLERATED | colon | carcinoma |
| p.C544Y | possibly damaging | 0.617 | 0.35693 | TOLERATED | brain | atypical teratoid-rhabdoid tumour |
| p.L620F | probably damaging | 0.959 | 0.52218 | *DAMAGING | Not specified | malignant melanoma |
| p.S662Y | probably damaging | 0.999 | 0.84437 | *DAMAGING | rectum | carcinoma |
| p.K813I | probably damaging | 1 | 0.79085 | DAMAGING | skin | malignant melanoma |
| p.H874R | possibly damaging | 0.779 | 0.14482 | TOLERATED | breast | carcinoma |
| p.K1272Q | probably damaging | 1 | 0.56744 | TOLERATED | larynx | carcinoma |
| p.D1284Y | probably damaging | 0.993 | 0.64532 | DAMAGING | colon | carcinoma |
| p.T1288M | probably damaging | 0.961 | 0.52143 | TOLERATED | caecum | carcinoma |
| p.G1293W | probably damaging | 1 | 0.96618 | DAMAGING | prostate | carcinoma |
| p.S1311Y | probably damaging | 1 | 0.90495 | DAMAGING | caecum | carcinoma |
| p.P1558R | possibly damaging | 0.834 | 0.18314 | TOLERATED | rectum | carcinoma |
| p.S1650Y | probably damaging | 1 | 0.66785 | DAMAGING | rectum | carcinoma |
| p.H356H | silent mutation |  |  | TOLERATED | caecum | carcinoma |
| p.T610T | silent mutation |  |  | TOLERATED | caecum | carcinoma |
| p.C540C | silent mutation |  |  | TOLERATED | prostate | carcinoma |
| p.A1674A | silent mutation |  |  | TOLERATED | rectum | carcinoma |
| p.D1681D | silent mutation |  |  | TOLERATED | breast | carcinoma |
| p.S1872S | silent mutation |  |  | TOLERATED | colon | carcinoma |
| p.L1478* | Nonsense mutation |  |  | DAMAGING | colon | carcinoma |
| p.C1654* | Nonsense mutation |  |  | DAMAGING | caecum | carcinoma |
| p.C452fs*21 | Frameshift deletion | |  | DAMAGING | colon | carcinoma |

*Stransky et al. , Science 333 (6046) 1157-1160

**Figure 2. Expression analyses of *NCKAP5* in HNSCC**

**
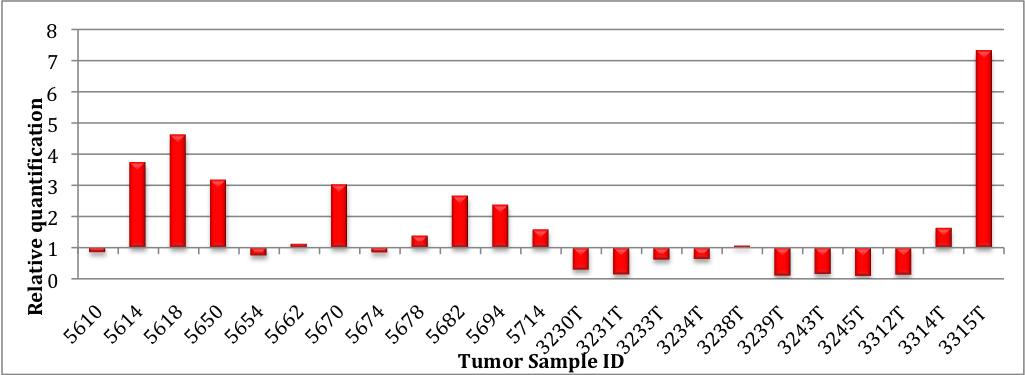
**


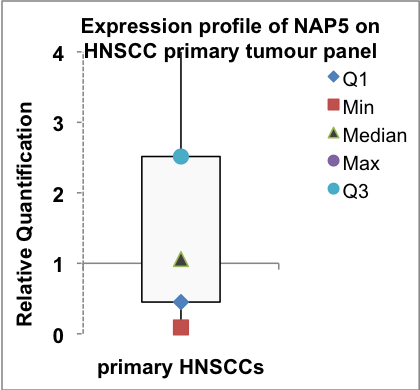


**Analysis of *NCKAP5* .** Relative quantification of expression shown as fold change was done by comparing the tumour samples against the available normal samples (which is set at value 1).

**Top Panel: Expression profile of *NCKAP5* in primary HNSCC and normal oral mucosa.** The y-axis represents the change in the relative fold expression of the tumour samples over the mean value of the normal tissue. A relative decrease in expression of *NCKAP5* was seen in 48% (11/23) of primary HNSCCs when compared with their adjacent normal mucosa samples. However, significant reduction was observed in only 26% (6/23) of primary HNSCCs

**Bottom Panel:** **Expression profile of *NCKAP5* in primary HNSCCs compared with the normal tissue.** The x-axis represents the primary HNSCC samples in the study. The y-axis represents the change in the relative fold expression compared to the mean expression in the normal samples. The mean expression values for the normal samples is averaged and normalized to 1 (Line on horizontal x-axis at value 1) against which, expression values for the primary HNSCCs are compared and an average fold change calculated. The box-plots show the first quartile, minimum, median, maximum and third quartile for each group of samples.

**Figure 3. Expression analyses of *NCKAP5* in other tumour types**


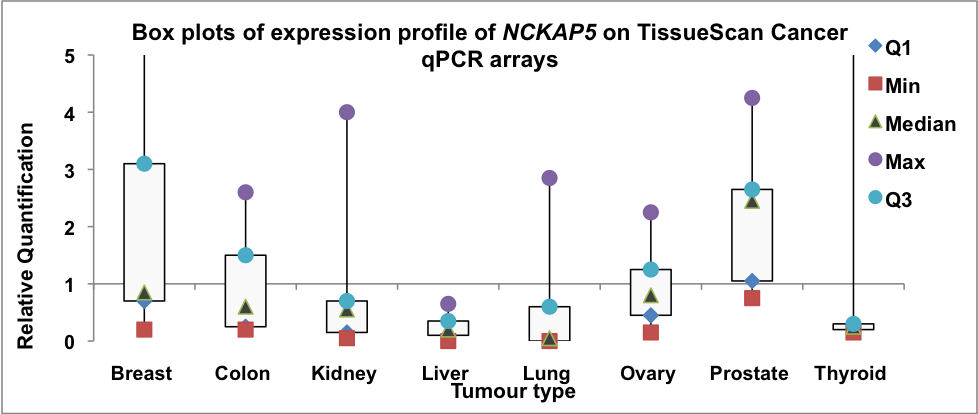


**Normalised expression profile of *NCKAP5* on TissueScan Cancer and Normal Tissue Cancer Survey 96-I cDNA Arrays (Origene Maryland, USA). The panel includes 12 tumour and 3 normal tissue samples from each of 8 different sites. The y-axis represents the change in the relative fold expression compared to the mean expression in the 3 normal tissues for each tumour type. The box-plots show the first quartile, minimum, median, maximum and third quartile for each group of samples. There is a relative decrease in expression of *NCKAP5* in all tumours except for those from breast and prostate.**

**Supplementary Data S16 - Integrative Analysis of Somatic Copy Number Changes and Gene Expression**

Table 1. Copy number gains in PPOLS and HNSCC of genes that show correlation with expression in SCNA regions showing significant difference between PPOLS and HNSCCs.

| **Chromosomal coordinates** | **Genes ordered by strength of correlation** | **Copy number gain** | | | | **High copy number gain** | | | |
| --- | --- | --- | --- | --- | --- | --- | --- | --- | --- |
|  |  | **Frequency (%)** | | | | **Frequency (%)** | | | |
|  |  | **PPOL** | **OSCC** | | | **PPOL** | **OSCC** | | |
|  |  |  | **LN+** | **LN-** | **All^3^** |  | **LN+** | **LN-** | **All^3^** |
|  |  | **N=7** | **N=10** | **N=7** | **N=28** | **N=7** | **N=10** | **N=7** | **N=28** |
| chr3:57677987-58154068 | *FLNB** | 0 | 0 | 0 | 0 | 0 | 0 | 0 | 0 |
| chr3:61422777-73764765 | *UBA3** | 14.29 | 0 | 0 | 0 | 0 | 0 | 0 | 0 |
|  | *SUCLG2* | 14.29 | 0 | 0 | 0 | 0 | 0 | 0 | 0 |
|  | *ARL6IP5** | 14.29 | 0 | 0 | 0 | 0 | 0 | 0 | 0 |
|  | *ATXN7* | 14.29 | 0 | 0 | 0 | 0 | 0 | 0 | 0 |
|  | *THOC7** | 14.29 | 0 | 0 | 0 | 0 | 0 | 0 | 0 |
|  | *C3ORF64* | 14.29 | 0 | 0 | 0 | 0 | 0 | 0 | 0 |
|  | *PSMD6** | 14.29 | 0 | 0 | 0 | 0 | 0 | 0 | 0 |
| chr3:87035206-88461236 | *CHMP2B** | 14.29 | 0 | 14.29 | 7.14 | 0 | 0 | 0 | 0 |
|  | *CGGBP1** | 14.29 | 0 | 14.29 | 3.57 | 0 | 0 | 0 | 0 |
| chr3:151842842-152984767 | *SIAH2** | 14.29 | 80 | 28.57 | 60.71 | 0 | 0 | 0 | 0 |
| chr3:157160169-158933894 | *CCNL1*** | 14.29 | 70 | 42.86 | 57.14 | 0 | 10 | 0 | 7.14 |
| chr3:160599782-161161335 | *SCHIP1** | 14.29 | 90 | 42.86 | 67.86 | 0 | 10 | 0 | 3.57 |
| chr3:161705726-162566403 | *KPNA4** | 14.29 | 70 | 42.86 | 60.71 | 0 | 10 | 0 | 3.57 |
| chr3:170691970-173911476 | *MYNN** | 14.29 | 80 | 42.86 | 64.29 | 0 | 10 | 0 | 3.57 |
|  | *TNIK** | 14.29 | 100 | 42.86 | 71.43 | 0 | 10 | 0 | 3.57 |
|  | *SLC31A1* | 0 | 30 | 85.71 | 50 | 0 | 0 | 0 | 0 |
|  | *SEC62** | 14.29 | 90 | 42.86 | 67.86 | 0 | 0 | 0 | 0 |
|  | *EIF5A2** | 14.29 | 90 | 42.86 | 67.86 | 0 | 10 | 0 | 3.57 |
| chr3:177707736-180451354 | *PIK3CA*** | 14.29 | 90 | 42.86 | 71.43 | 0 | 10 | 0 | 3.57 |
| chr3:185035692-185529164 | *AP2M1* | 14.29 | 90 | 42.86 | 71.43 | 0 | 0 | 0 | 0 |
|  | *PARL* | 14.29 | 90 | 42.86 | 67.86 | 0 | 0 | 0 | 0 |
|  | *ALG3** | 14.29 | 90 | 42.86 | 71.43 | 0 | 0 | 0 | 3.57 |
|  | *EIF4G1*** | 14.29 | 90 | 42.86 | 71.43 | 0 | 0 | 0 | 3.57 |
|  | *DVL3*** | 14.29 | 90 | 42.86 | 67.86 | 0 | 0 | 0 | 0 |
|  | *ABCF3** | 14.29 | 90 | 42.86 | 71.43 | 0 | 0 | 0 | 0 |
| chr3:198578155-199298372 | *RPL35A** | 14.29 | 90 | 42.86 | 64.29 | 0 | 0 | 0 | 0 |
| chr7:1631815-7317208 | *EIF2AK1** | 14.29 | 40 | 85.71 | 67.86 | 0 | 10 | 0 | 7.14 |
|  | *C7ORF28A* | 28.57 | 40 | 85.71 | 67.86 | 0 | 10 | 0 | 7.14 |
|  | *RAC1*** | 14.29 | 30 | 85.71 | 64.29 | 0 | 10 | 0 | 7.14 |
|  | *CHST12** | 14.29 | 30 | 71.43 | 60.71 | 0 | 10 | 0 | 10.71 |
|  | *KDELR2* | 14.29 | 40 | 85.71 | 67.86 | 0 | 10 | 0 | 10.71 |
|  | *RNF216** | 28.57 | 40 | 71.43 | 64.29 | 0 | 10 | 0 | 14.29 |
|  | *AIMP2** | 14.29 | 40 | 85.71 | 67.86 | 0 | 10 | 0 | 7.14 |
|  | *NUDT1** | 14.29 | 30 | 71.43 | 60.71 | 0 | 10 | 0 | 7.14 |
|  | *WIPI2* | 14.29 | 40 | 85.71 | 67.86 | 0 | 10 | 0 | 7.14 |
|  | *PMS2CL* | 14.29 | 30 | 85.71 | 60.71 | 0 | 10 | 0 | 7.14 |
| chr9:85367221-85868737 | *RMI1** | 0 | 20 | 71.43 | 39.29 | 0 | 10 | 0 | 3.57 |
|  | *HNRNPK*** | 0 | 20 | 71.43 | 39.29 | 0 | 10 | 0 | 3.57 |
| chr9:97596715-98774190 | *CDC14B** | 0 | 40 | 71.43 | 53.57 | 0 | 10 | 14.29 | 7.14 |
| chr9:99427940-100373274 | *ANP32B** | 0 | 20 | 85.71 | 46.43 | 0 | 0 | 0 | 0 |
| chr9:101504820-101852863 | *ERP44*** | 14.29 | 30 | 71.43 | 46.43 | 0 | 0 | 14.29 | 3.57 |
| chr9:123376788-125083831 | *RABGAP1* | 0 | 30 | 57.14 | 42.86 | 0 | 0 | 0 | 0 |
|  | *RC3H2* | 0 | 30 | 57.14 | 42.86 | 0 | 0 | 0 | 0 |
| chr9:126825879-127177239 | *GAPVD1* | 14.29 | 30 | 71.43 | 46.43 | 0 | 0 | 0 | 0 |
|  | *PPP6C** | 0 | 30 | 71.43 | 50 | 0 | 0 | 0 | 0 |
|  | *RABEPK* | 0 | 30 | 71.43 | 50 | 0 | 0 | 0 | 0 |
| chr9:129518474-129927677 | *PTGES2** | 14.29 | 40 | 85.71 | 57.14 | 0 | 0 | 0 | 0 |
|  | *FPGS* | 14.29 | 50 | 85.71 | 67.86 | 0 | 0 | 0 | 0 |
| chr9:132985780-136636113 | *NUP214** | 14.29 | 40 | 85.71 | 60.71 | 0 | 10 | 0 | 3.57 |
|  | *BRD3** | 0 | 20 | 71.43 | 53.57 | 0 | 10 | 0 | 3.57 |
|  | *BAT2L1* | 0 | 40 | 71.43 | 57.14 | 0 | 10 | 0 | 3.57 |
|  | *DDX31** | 0 | 40 | 71.43 | 53.57 | 0 | 0 | 0 | 0 |
| chr9:137291321-139534231 | *KIAA0649** | 0 | 20 | 71.43 | 53.57 | 0 | 10 | 0 | 3.57 |
|  | *SDCCAG3** | 0 | 20 | 71.43 | 53.57 | 0 | 10 | 0 | 3.57 |
|  | *MAN1B1** | 14.29 | 20 | 85.71 | 57.14 | 0 | 10 | 0 | 7.14 |
|  | *ANAPC2** | 14.29 | 20 | 85.71 | 60.71 | 0 | 10 | 0 | 3.57 |
|  | *NELFB* | 14.29 | 20 | 85.71 | 57.14 | 0 | 10 | 0 | 3.57 |
|  | *NOTCH1*** | 14.29 | 20 | 71.43 | 53.57 | 0 | 20 | 0 | 7.14 |
| Chr14:62865875-63862093 | *HSPA2** | 14.29 | 50 | 14.29 | 32.14 | 0 | 10 | 0 | 7.14 |
|  | *PPP2R5E** | 0 | 50 | 14.29 | 35.71 | 0 | 10 | 0 | 7.14 |
| Chr3:78706269-79206160 | *ROBO1*** | 14.29 | 0 | 0 | 3.57 | 0 | 0 | 0 | 0 |
| chr3:186575268-187080482 | *SENP2** | 28.57 | 90 | 42.86 | 67.86 | 0 | 0 | 0 | 0 |
| chr10:16585949-17022250 | *RSU1** | 0 | 0 | 0 | 0 | 0 | 0 | 0 | 0 |
| chr10:26833288-28028304 | *ABI1** | 0 | 20 | 0 | 10.71 | 0 | 0 | 0 | 0 |
|  | *YME1L1** | 0 | 20 | 0 | 10.71 | 0 | 0 | 0 | 0 |
| chr17:8028078-9207567 | *STX8** | 0 | 20 | 0 | 17.86 | 0 | 0 | 0 | 0 |

Genes showing significant correlation with gene expression in integrative analyses after correction for multiple testing (adj. p<0.05), are indicated by double asterisks (**); genes showing nominal significance (p<0.05) only, are indicated by a single asterisk (*).

**Table 2. Copy number losses in PPOLS and HNSCC of genes that show correlation with expression in SCNA regions showing significant difference between PPOLS and HNSCCs.**

| **Chromosomal coordinates** | **Genes ordered by strength of correlation** | **Single Copy Loss** | | | | **Homozygous Loss** | | | |
| --- | --- | --- | --- | --- | --- | --- | --- | --- | --- |
|  |  | **Frequency (%)** | | | | **Frequency (%)** | | | |
|  |  | **PPOL** | **HNSCC** | | | **PPOL** | **HNSCC** | | |
|  |  |  | **LN+** | **LN-** | **All^3^** |  | **LN+** | **LN-** | **All^3^** |
|  |  | **N=7** | **N=10** | **N=7** | **N=28** | **N=7** | **N=10** | **N=7** | **N=28** |
| chr3:57677987-58154068 | *FLNB** | 28.57 | 90 | 42.86 | 71.43 | 0 | 0 | 0 | 0 |
| chr3:61422777-73764765 | *UBA3** | 14.29 | 100 | 71.43 | 85.71 | 0 | 0 | 0 | 0 |
|  | *SUCLG2* | 42.86 | 90 | 85.71 | 85.71 | 0 | 10 | 0 | 14.29 |
|  | *ARL6IP5** | 28.57 | 100 | 71.43 | 85.71 | 0 | 0 | 0 | 0 |
|  | *ATXN7* | 28.57 | 100 | 85.71 | 85.71 | 0 | 0 | 0 | 0 |
|  | *THOC7** | 28.57 | 100 | 85.71 | 85.71 | 0 | 0 | 0 | 0 |
|  | *C3ORF64* | 28.57 | 100 | 71.43 | 85.71 | 0 | 0 | 0 | 0 |
|  | *PSMD6** | 28.57 | 100 | 85.71 | 85.71 | 0 | 0 | 0 | 0 |
| chr3:87035206-88461236 | *CHMP2B** | 14.29 | 80 | 71.43 | 64.29 | 0 | 10 | 0 | 7.14 |
|  | *CGGBP1** | 28.57 | 90 | 71.43 | 67.86 | 0 | 10 | 0 | 7.14 |
| chr3:151842842-152984767 | *SIAH2** | 0 | 0 | 0 | 0 | 0 | 0 | 0 | 0 |
| chr3:157160169-158933894 | *CCNL1*** | 0 | 0 | 0 | 0 | 0 | 0 | 0 | 0 |
| chr3:160599782-161161335 | *SCHIP1** | 0 | 0 | 0 | 0 | 0 | 0 | 0 | 0 |
| chr3:161705726-162566403 | *KPNA4** | 0 | 0 | 0 | 0 | 0 | 0 | 0 | 0 |
| chr3:170691970-173911476 | *MYNN** | 0 | 0 | 0 | 0 | 0 | 0 | 0 | 0 |
|  | *TNIK** | 0 | 0 | 0 | 0 | 0 | 0 | 0 | 0 |
|  | *SLC31A1* | 0 | 0 | 0 | 3.57 | 0 | 0 | 0 | 0 |
|  | *SEC62** | 0 | 0 | 0 | 0 | 0 | 0 | 0 | 0 |
|  | *EIF5A2** | 0 | 0 | 0 | 0 | 0 | 0 | 0 | 0 |
| chr3:177707736-180451354 | *PIK3CA*** | 0 | 0 | 0 | 0 | 0 | 0 | 0 | 0 |
| chr3:185035692-185529164 | *AP2M1* | 0 | 0 | 0 | 0 | 0 | 0 | 0 | 0 |
|  | *PARL* | 0 | 0 | 0 | 0 | 0 | 0 | 0 | 0 |
|  | *ALG3** | 0 | 0 | 0 | 0 | 0 | 0 | 0 | 0 |
|  | *EIF4G1*** | 0 | 0 | 0 | 0 | 0 | 0 | 0 | 0 |
|  | *DVL3*** | 0 | 0 | 0 | 0 | 0 | 0 | 0 | 0 |
|  | *ABCF3** | 0 | 0 | 0 | 0 | 0 | 0 | 0 | 0 |
| chr3:198578155-199298372 | *RPL35A** | 0 | 0 | 0 | 0 | 0 | 0 | 0 | 0 |
| chr7:1631815-7317208 | *EIF2AK1** | 0 | 0 | 0 | 0 | 0 | 0 | 0 | 0 |
|  | *C7ORF28A* | 0 | 0 | 0 | 0 | 0 | 0 | 0 | 0 |
|  | *RAC1*** | 0 | 0 | 0 | 0 | 0 | 0 | 0 | 0 |
|  | *CHST12** | 0 | 0 | 0 | 0 | 0 | 0 | 0 | 0 |
|  | *KDELR2* | 0 | 0 | 0 | 0 | 0 | 0 | 0 | 0 |
|  | *RNF216** | 0 | 0 | 0 | 0 | 0 | 0 | 0 | 0 |
|  | *AIMP2** | 0 | 0 | 0 | 0 | 0 | 0 | 0 | 0 |
|  | *NUDT1** | 0 | 0 | 0 | 0 | 0 | 0 | 0 | 0 |
|  | *WIPI2* | 0 | 0 | 0 | 0 | 0 | 0 | 0 | 0 |
|  | *PMS2CL* | 0 | 0 | 0 | 0 | 0 | 0 | 0 | 0 |
| chr9:85367221-85868737 | *RMI1** | 0 | 0 | 0 | 3.57 | 0 | 0 | 0 | 0 |
|  | *HNRNPK*** | 0 | 0 | 0 | 3.57 | 0 | 0 | 0 | 0 |
| chr9:97596715-98774190 | *CDC14B** | 0 | 0 | 0 | 0 | 0 | 0 | 0 | 0 |
| chr9:99427940-100373274 | *ANP32B** | 0 | 0 | 0 | 0 | 0 | 0 | 0 | 0 |
| chr9:101504820-101852863 | *ERP44*** | 0 | 0 | 0 | 3.57 | 0 | 0 | 0 | 0 |
| chr9:123376788-125083831 | *RABGAP1* | 0 | 0 | 0 | 0 | 0 | 0 | 0 | 0 |
|  | *RC3H2* | 0 | 0 | 0 | 0 | 0 | 0 | 0 | 0 |
| chr9:126825879-127177239 | *GAPVD1* | 0 | 0 | 0 | 0 | 0 | 0 | 0 | 0 |
|  | *PPP6C** | 0 | 0 | 0 | 0 | 0 | 0 | 0 | 0 |
|  | *RABEPK* | 0 | 0 | 0 | 0 | 0 | 0 | 0 | 0 |
| chr9:129518474-129927677 | *PTGES2** | 0 | 0 | 0 | 0 | 0 | 0 | 0 | 0 |
|  | *FPGS* | 0 | 0 | 0 | 0 | 0 | 0 | 0 | 0 |
| chr9:132985780-136636113 | *NUP214** | 0 | 0 | 0 | 0 | 0 | 0 | 0 | 0 |
|  | *BRD3** | 0 | 0 | 0 | 0 | 0 | 0 | 0 | 0 |
|  | *BAT2L1* | 0 | 0 | 0 | 0 | 0 | 0 | 0 | 0 |
|  | *DDX31** | 0 | 0 | 0 | 0 | 0 | 0 | 0 | 0 |
| chr9:137291321-139534231 | *KIAA0649** | 0 | 0 | 0 | 0 | 0 | 0 | 0 | 0 |
|  | *SDCCAG3** | 0 | 0 | 0 | 0 | 0 | 0 | 0 | 0 |
|  | *MAN1B1** | 0 | 0 | 0 | 0 | 0 | 0 | 0 | 0 |
|  | *ANAPC2** | 0 | 0 | 0 | 0 | 0 | 0 | 0 | 0 |
|  | *NELFB* | 0 | 0 | 0 | 0 | 0 | 0 | 0 | 0 |
|  | *NOTCH1*** | 0 | 0 | 0 | 0 | 0 | 0 | 0 | 0 |
| Chr14:62865875-63862093 | *HSPA2** | 0 | 0 | 0 | 0 | 0 | 0 | 0 | 0 |
|  | *PPP2R5E** | 0 | 0 | 0 | 0 | 0 | 0 | 0 | 0 |
| Chr3:78706269-79206160 | *ROBO1*** | 42.86 | 80 | 85.71 | 78.57 | 0 | 10 | 0 | 14.29 |
| chr3:186575268-187080482 | *SENP2** | 0 | 0 | 0 | 0 | 0 | 0 | 0 | 0 |
| chr10:16585949-17022250 | *RSU1** | 0 | 50 | 57.14 | 60.71 | 0 | 0 | 0 | 0 |
| chr10:26833288-28028304 | *ABI1** | 0 | 50 | 57.14 | 60.71 | 0 | 0 | 0 | 0 |
|  | *YME1L1** | 0 | 50 | 57.14 | 64.29 | 0 | 0 | 0 | 0 |
| chr17:8028078-9207567 | *STX8** | 28.57 | 10 | 42.86 | 21.43 | 0 | 0 | 0 | 0 |

Genes showing significant correlation with gene expression in integrative analyses after correction for multiple testing (adj. p<0.05), are indicated by double asterisks (**); genes showing nominal significance (p<0.05) only, are indicated by a single asterisk (*).

**Further Information:** **Correlating gene expression to somatic copy number alterations**

To identify potential drivers of somatic copy number alterations we considered the correlation between gene expression and copy number for each gene across all samples. This analysis is based on the hypothesis that a consistent phenotypic effect should be observed on the gene expression of CNA driver genes, while passenger genes might remain unaffected.

To do so the gene expression data provided by (Hunter et al., 2006) was used and integrated to our copy number data resulting in a dataset of 29 samples with both gene expression and copy number values (18 PPOL and 11 HNSCC cell lines). We used PANP (Warren P., Bioconductor) to reduce the number of genes considered for further analysis to 8255 unique genes expressed in at least one sample.

Before correlating the SCNA and gene expression values corrections were applied for polyploidy and heterogeneity.

When considered a by-product of instability rather than a response of biological significance, the copy number (CN) values were altered to remove the ubiquitous chromosomal amplification observed in polyploid samples. To do so, all the CN values inferred for SNPs in chromosome arms with mean CN larger than 2.5 were reduced by one unit. The reduction was however rejected in the cases of heterozygous copy neutral calls (CN2 LOH0), copy losses (CN1) and homozygous deletions (CN0). These states were not altered.

Due to heterogeneity, the gene expression values obtained did not represent the expression of the CN-altered cells solely. For each genomic region, a non-negligible proportion of cells do not harbour any alteration. The proportion of cells with normal heterozygous copy number in each region was estimated using OncoSNP (Yau C., et al., 2010). When investigating the correlation between SCNAs and mRNA expression the weighted mean CN and LOH values of this mixture were used.

The resulting correlation coefficients across genes displayed a tendency for positive correlation of expression with copy number and negative correlation with LOH (i.e. higher gene expression in amplified regions and lower expression in LOH regions). Furthermore an analysis of auto-correlation of the correlation coefficients along the genome revealed significant level of auto-correlation across the positively correlated genes in the CN analysis, but no auto-correlation in the negatively correlated genes. The reverse was observed in the LOH analysis. Thus only genes displaying higher expression when amplified or lower expression when undergoing loss of heterozygosity were further considered. The significance of the correlation of the filtered list were corrected for multiple testing using a Sidak procedure resulting in 484 CN vs GE and 130 LOH vs GE genes (51 genes overlapping) with p-value below 0.05.

**Identifying regions of differential CN and LOH between PPOLS an HNSCC**

In order to find regions of copy number alterations typical of PPOLS or HNSCC samples, we examine the common genetic pattern of each group and searched for significant differences.

In the case of differential CN events we considered the contingency table displaying the number of samples by group (i.e. PPOL/HNSCC) and amplification status (i.e. amplification/neutral/deletion) and performing a Chi-squared test for each unique genomic region. The same was performed to identify differential LOHs by considering the LOH status instead of the amplification status. This yielded 208 significant CN regions and 192 significant LOH regions (p < 0.05).

**Integrating differentially altered regions with significant CN vs GE genes to identify candidate driver genes**

The genes with expression identified as responding significantly to underlying alterations, both CNA and LOH, are suggested as potential driver genes of the regions with distinct patterns of alteration between PPOL and HNSCC.

The overlap of those two lists identified 23 CN regions with 61 potential driver genes and 6 LOH regions with 6 potential driver genes. Those are proposed as candidate differentiators of carcinoma versus dysplasia.

The genes underlying these regions are presented in Tables 1-2 showing copy number gains (Table 1) and losses (Table 2) these loci in PPOLS and HNSCC overall as well as in LN^+ve^ and LN^–ve^ HNSCC where these data were available.

**References**

[Hunter KD](https://www.ncbi.nlm.nih.gov/pubmed/?term=Hunter KD%5BAuthor%5D&cauthor=true&cauthor_uid=16885335)1, [Thurlow JK](https://www.ncbi.nlm.nih.gov/pubmed/?term=Thurlow JK%5BAuthor%5D&cauthor=true&cauthor_uid=16885335), Fleming J, Drake PJ, Vass JK, [Kalna G](https://www.ncbi.nlm.nih.gov/pubmed/?term=Kalna G%5BAuthor%5D&cauthor=true&cauthor_uid=16885335), [Higham DJ](https://www.ncbi.nlm.nih.gov/pubmed/?term=Higham DJ%5BAuthor%5D&cauthor=true&cauthor_uid=16885335), [Herzyk P](https://www.ncbi.nlm.nih.gov/pubmed/?term=Herzyk P%5BAuthor%5D&cauthor=true&cauthor_uid=16885335), Macdonald DG, Parkinson EK, Harrison PR. Divergent routes to oral cancer. Cancer Res. 2006 Aug 1;66(15):7405-13.

Warren P. panp: Presence-Absence Calls from Negative Strand Matching Probesets. (Bioconductor; <https://bioconductor.riken.jp/packages/3.2/bioc/html/panp.html>).

Christopher Yau, Dmitri Mouradov, Robert N Jorissen, Stefano Colella, Ghazala Mirza, Graham Steers, Adrian Harris, Jiannis Ragoussis, Oliver Sieber, Christopher C Holmes

A statistical approach for detecting genomic aberrations in heterogeneous tumor samples from single nucleotide polymorphism genotyping data. Genome Biol. 2010; 11(9): R92.  Published online 2010 Sep 21. doi: 10.1186/gb-2010-11-9-r92

**SUPPLEMENTARY DATA 17 -** **Analysis of CLDN1 and BCL2L1 Expression in Primary HNSCC and PPOL**

Figure 1. Expression of CLDN1 in PPOLS and HNSC


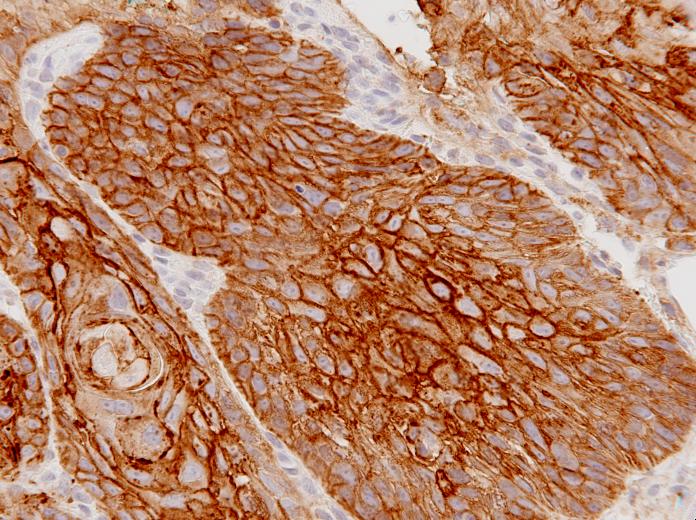

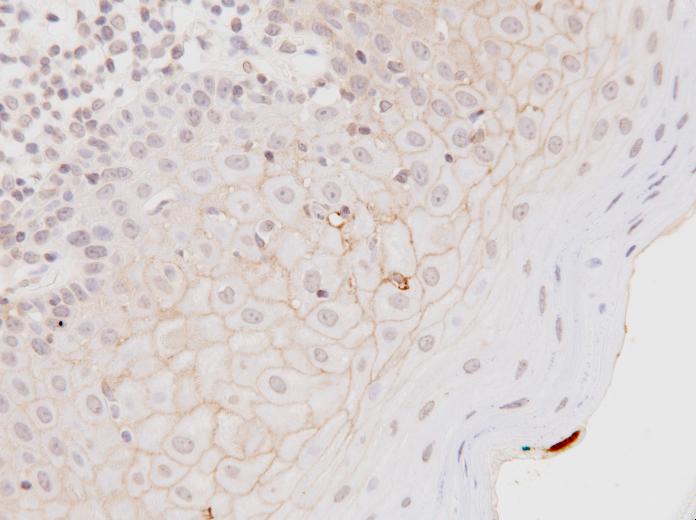

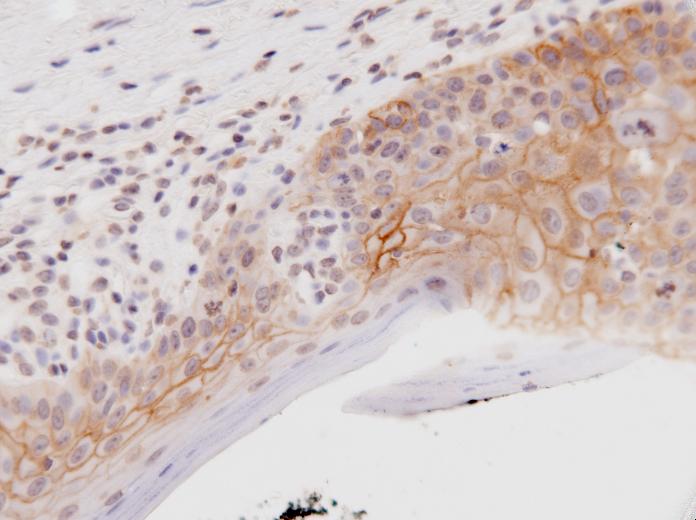

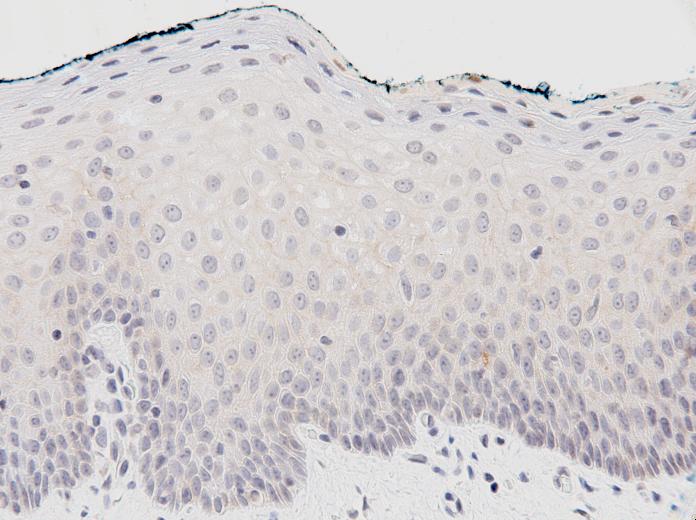


**A**

**B**

**C**

**D**

**Top Panel:** Box plots of optical density (OD) readings of CLDN1 immunostaining of normal oral epithelium (N), Low grade PPOL (LG), High grade PPOL(HG), and HNSCC (T). Within each box the top line represents the third quartile, the middle line represents the median and the bottom line represents the first quartile. Median OD scores for each tissue type were as follows; N = 0.034, LG = 0.035, HG = 0.035, T = 0.077. HNSCC showed significantly higher expression compared to other groups (p<0.00001, Mann Whitney U Test).

**Bottom Panel:** Representative images of CLDN1 immunostaining in normal oral epithelium (A), Low grade PPOL (B), High grade PPOL(C), and HNSCC (D).

Figure 2. Expression of BCL-XL in PPOLS and HNSCC


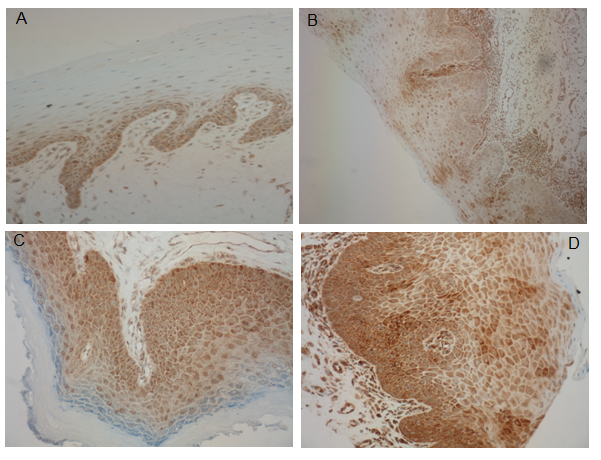


**Top Panel.** Box plots of optical density (OD) readings of Bcl-XL IHC staining in fibroepithelial polyps (FEP), lichen planus (ILP), low grade PPOL (LG), high grade PPOL (HG) and HNSCC (T). Parametric one-way ANOVA testing showed significant differences between the groups (p<0.0001); post hoc T test confirmed significant differences (p<0.0001) between immunostaining density in HNSCC compared to all other groups. There were no differences between the other groups.

**Bottom Panel.** Representative images of BCL-XL immunostaining in normal oral epithelium (A), Low grade PPOL (B), High grade PPOL(C), and HNSCC (D).

**Further information:**

**Analysis of CLDN1 Expression**

**Materials and Methods**

HNSCC and PPOLs diagnosed between 2008 and 2010 were retrieved from the Central Manchester Foundation Trust (CMFT) pathology database. All cases reviewed by a consultant pathologist to confirm the diagnosis. PPOLs were categorised into high grade (HG) including moderate and high dysplasia and low grade (LG) including mild dysplasia, using the information from the patient report and an additional review of the histology by a consultant pathologist. All samples were coded and anonymised.

Sample panel consisted of 42 HNSCC, 19 HG PPOLs biopsies and 24 LG PPOLs. Normal epithelium adjacent to HNSCC within 39 of the 42 tumour blocks was also analysed. Clinical parameters from all of the HNSCC cases were retrieved from each case report. These included the patient sex and age along with the tumour differentiation, stage, lymph node invasion, lympho-vascular invasion, and peri-neural invasion status.

Standard protocols for immunohistochemistry were followed with an optimised, rabbit polyclonal IgG antibody to Claudin 1, (Abcam, Cambridge, UK) at a dilution of 1:500. IHC staining was performed on a Ventana Benchmark XT Automated Immunostaining Module (Ventana Medical Systems, Tucson, AZ). All other reagents used were also supplied from Ventana Medical Systems. Optimisation of Claudin 1 also involved the used of Protease 1 (Ventana, 760-2018). Positive (normal human colon) and negative (minus the primary antibody) controls were processed with each batch.

Intensity and area of immunostaining of Claudin 1 was measured using a semi-automated image analysis method. This incorporated a standard light microscope attached to a CRI Nuance spectral analyzer (CRI, Woburn, MA), which was supported by Nuance Alpha, 1.6.2.368 software. This system used spectral unmixing of the IHC stained sections to identify and measure the areas of DAB positive staining. This method of IHC quantification has been as validated in recent publications (Das, et al. 2004, Taylor and Levenson 2006). Five random, conventional bright-field image cubes of the epithelium in each IHC stained section were collected at 10nm wavelength intervals from 480 nm (blue) to 700 nm (red) at a 40x magnification. In addition 38 of the 42 HNSCC tissue slides also had five random image cubes taken of its adjacent normal epithelium.

The DAB spectral profile of each image cube was unmixed from the haematoxylin spectral profile in each cube using Nuance 2.6.0 software (CRI, Woburn, MA), creating a composite false colour image cube. This enabled the intensity of the DAB staining per pixel of each positively stained site within the image cube to be measured as an average optical density (OD). The average of the ODs recorded in all five images cubes taken per slide was calculated to produce an overall OD score for that particular tissue sample. Further description of this methodology can be found in Byers et al 2008.

Statistical analysis was performed using the statistical software packages Minitab 16 2010 (Minitab Inc, USA) and Medcalc version 12.3.0 (Medcalc Software, Belgium). For all of the statistical tests a p value of < 0.05 was considered to be statistically significant. Basic statistical analysis and Anderson-Darling tests were performed to test the data’s normality in Minitab. The data sets were not normally distributed and so non-parametric statistical analysis was performed. The significance of differences in Claudin 1 immunostaining optical density between the different sample groups was tested for significance using the Kruskal Wallis test for non-parametric data and post-hoc analysis with Mann-Whitney U test. Spearman’s rank correlation coefficient for non-parametric data was used to test for significance of the correlation between Claudin 1 immunostaining and progression towards HNSCC and clinical parameters.

**Analysis of BCL2L1 Expression in Primary HNSCC and PPO**

**Materials and methods**

HNSCC and PPOLs diagnosed between 2012 and 2014 were retrieved from the Central Manchester Foundation Trust (CMFT) pathology database. All samples were coded and anonymised. Sample panel consisted of 36 HNSCC, 29 HG PPOLs biopsies, 27 LG PPOLs, 10 ILP and 10 FEP. Clinical parameters from all of the HNSCC cases were retrieved from each case report. These included the patient sex and age along with the tumour differentiation, stage, lymph node invasion, lympho-vascular invasion, and peri-neural invasion status.

All IHC staining was performed on the Ventana Benchmark XT automated IHC staining module with reagents also supplied by Ventana Medical Systems. Heat mediated antigen retrieval was performed for 36 minutes. Bcl-XL antibody was prepared at a 1/400 dilution and incubated at room temperature for 36 minutes.

Bcl-XL IHC Staining intensity was scored using the CRI Nuance Multi-spectral Imaging System. The DAB and haematoxylin spectral profiles were identified and unmixed in order to accurately measure the intensity of the DAB substrate reaction of bound primary antibody to Bcl-XL only. Five random, bright-field image cubes in each stained section were collected at 10nm wavelength intervals from 500 nm to 720 nm at a 40x magnification. For each cube area, the intensity of the DAB positive staining per pixel was measured as an average optical density (OD) value. The average OD recorded in all five image cubes taken per slide were calculated to produce an overall OD score for that particular tissue block.

Statistical analysis was performed using the statistical software package Minitab version 17.2.1 (Minitab Inc, USA). For all of the statistical tests a p value of < 0.05 was considered to be statistically significant, allowing the null hypothesis to be rejected and the alternative hypothesis to be accepted.

**SUPPLEMENTARY DATA S18**– KEGG PATHWAY GENE ENRICHMENT IN GISTIC REGIONS

| **PATHWAY** | **IMMORTAL**  **PPOLS** | **LN-VE**  **HNSCC** | **LN+VE**  **HNSCC** | **ALL HNSCC** | **ALL GISTIC REGION GENES CORRELATING WITH EXPRESSION IN SAMPLES WHERE DATA AVAILABLE** |
| --- | --- | --- | --- | --- | --- |
| Pathways in cancer | 2.46E-06 | 2.90E-05 | 1.13E-12 | 1.97E-12 | 5.01E-17 |
| Endocytosis | 1.61E-07 | 0.0011 | 1.54E-12 | 2.89E-09 | 9.12E-14 |
| Small cell lung cancer | X | 7.52E-06 | 5.11E-05 | 6.00E-07 | 2.52E-07 |
| Ubuiquitin mediated proteolysis | X | X | 0.0001 | 1.52E-06 | 1.41E-06 |
| Focal adhesion | 0.0002 | 0.0006 | 2.18E-10 | 1.14E-05 | 7.22E-11 |
| Wnt signaling pathway | 0.0007 | X | 1.47E-11 | 1.14E-05 | 1.02E-07 |
| Apoptosis | X | X | 0.001 | 2.96E-05 | 0.0005 |
| Regulation of actin cytoskeleton | 7.62E-05 | X | 2.20E-08 | 3.71E-05 | 1.02E-09 |
| Colorectal cancer | 2.33E-05 | X | 0.0002 | 4.84E-05 | 2.83E-05 |
| Pancreatic cancer | X | X | 0.006 | 5.31E-05 | 1.21E-07 |
| MAPK signaling pathway | 5.34E-09 | X | 1.63E-12 | 0.0001 | 3.38E-09 |
| Phagosome | X | X | 3.67E-13 | 0.0002 | X |
| Cell adhesion molecules (CAMs) | X | X | 2.79E-11 | 0.0002 | X |
| mTOR signalling pathway | X | X | X | 0.0003 | 0.0002 |
| Melanoma | 0.0004 | X | 0.0005 | 0.0005 | 0.0055 |
| Non-small cell lung cancer | 0.0004 | X | X | 0.0005 | 8.92E-06 |
| Axon guidance | 0.0002 | X | 8.40E-08 | 0.0013 | 6.80E-09 |
| p53 signaling pathway | 2.00E-04 | X | 4.73E-05 | 0.0013 | X |
| Cell cycle | X | 0.0046 | 8.46E-06 | 0.002 | 3.38E-09 |
| Endometrial cancer | 0.0004 | X | X | 0.0038 | X |
| Glioma | X | 0.0074 | 0.0024 | 0.0066 | 4.16E-05 |
| TGF-beta signaling pathway | 0.0007 | X | 1.52E-05 | 0.0075 | X |
| Bladder cancer | X | X | 0.0052 | 0.0075 | X |
| Erb signalling pathway | 0.0002 | X | X | 0.0098 | 2.73E-10 |
| Toll-like receptor signaling pathway | 8.45E-05 | X | 1.11E-07 | X | X |
| Prostate cancer | 0.0003 | X | 0.0013 | X | 3.72E-05 |
| PPAR signaling pathway | 0.0007 | X | 0.006 | X | X |
| Notch signaling pathway | X | 0.0066 | 0.0003 | X | 0.0021 |
| Jak-STAT signaling pathway | X | X | 5.76E-14 | X | 0.0019 |
| Regulation of autophagy | X | X | 3.75E-07 | X | X |
| Mismatch repair | X | X | 0.0083 | X | 0.0003 |

**SUPPLEMENTARY DATA S19 - SCNA of genes in cancer realted kegG pathways enriched in gistic regions**

The following bar charts illustrate SCNAs of cancer-relevant pathway genes that are significantly enriched in the GISTIC regions in PPOLs and HNSCC. For each pathway, two charts are shown illustrating frequency of copy number gains (top panel) and losses (bottom panel) in PPOLs, all HNSCC, and HNSCCs with and without nodal metastases (LN+ve and LN-ve respectively). Genes showing significant correlation with expression in integrative analyses after correction for multiple testing (adj. p<0.05), are indicated by double asterisks (**); genes showing nominal significance (p<0.05) only are indicated by a single asterisk (*). Only genes showing at least 40% frequency of SCNA in at least one subgroup are shown

**Figure 1. Pathways in Cancer**


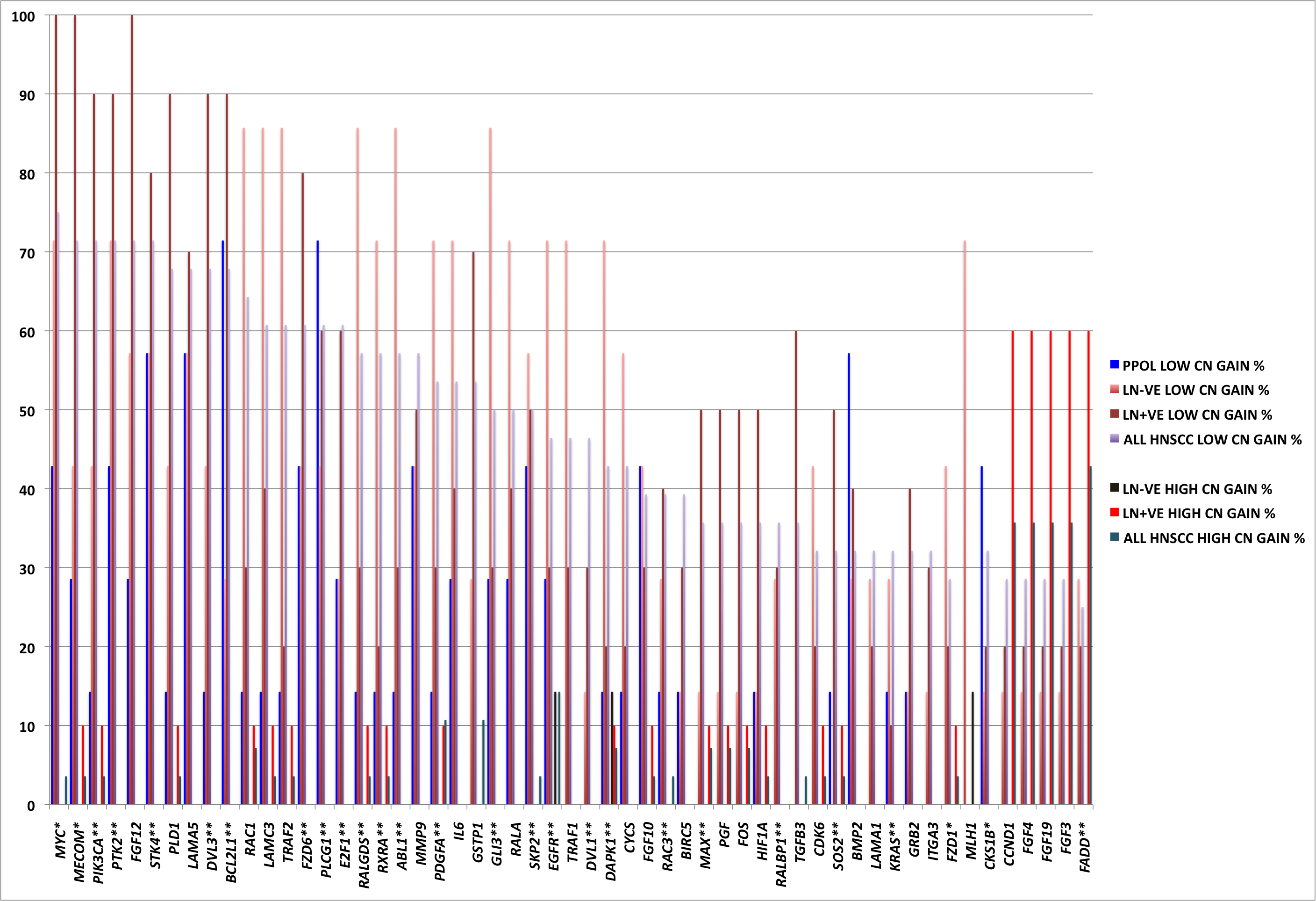


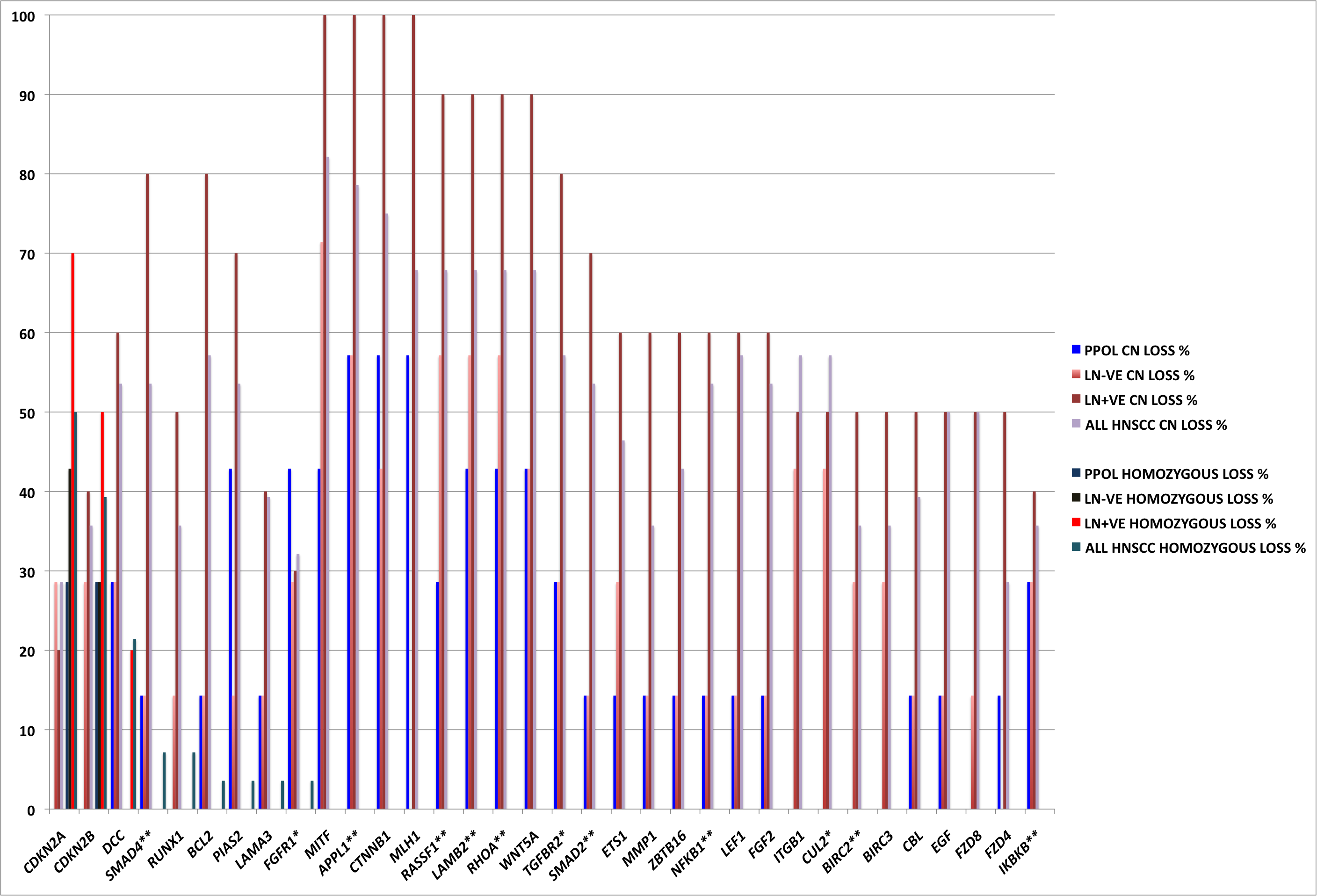


**Figure 2. Apoptosis Pathway**


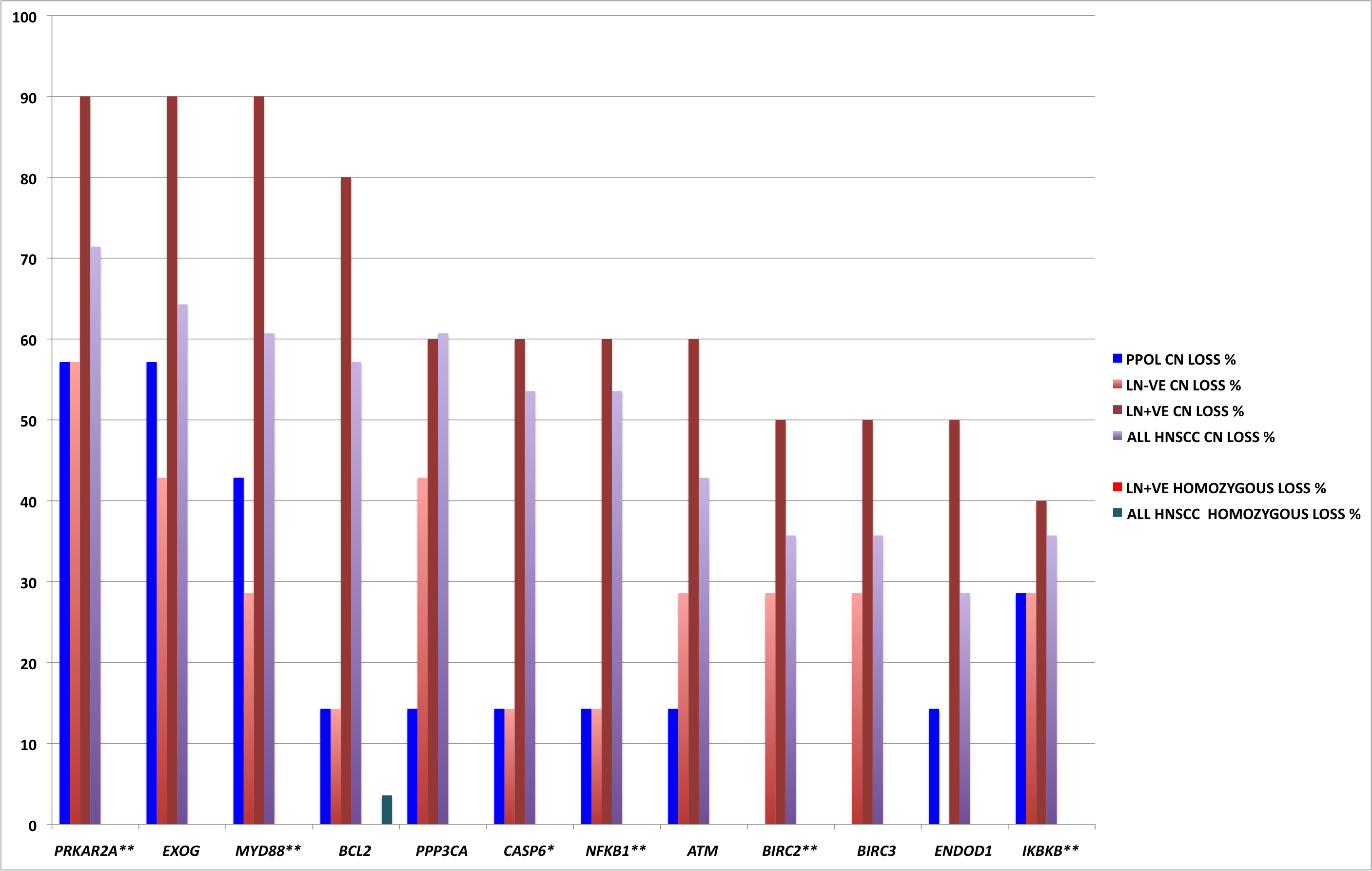


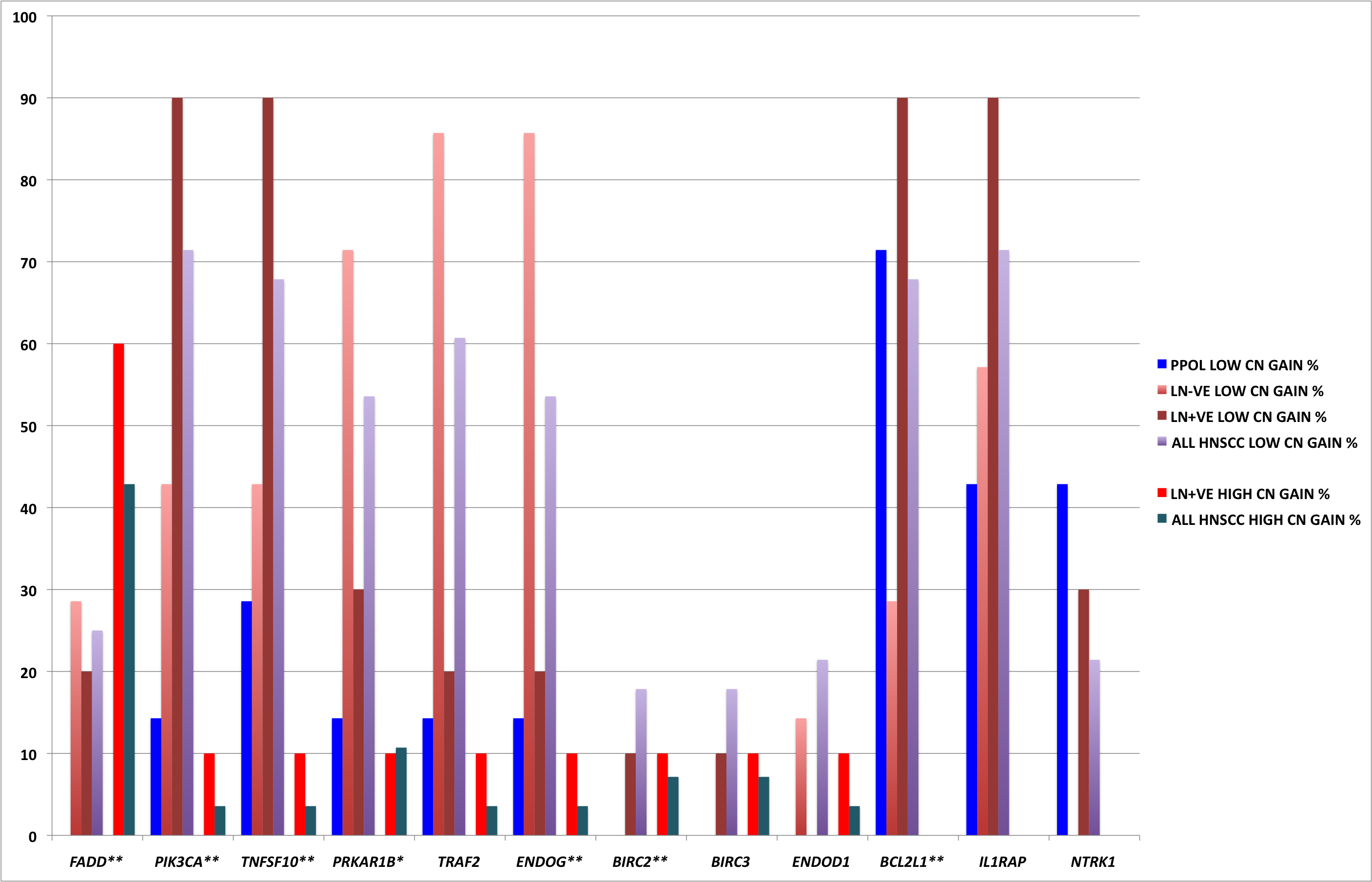


**Figure 3. Axon Guidance Pathway**


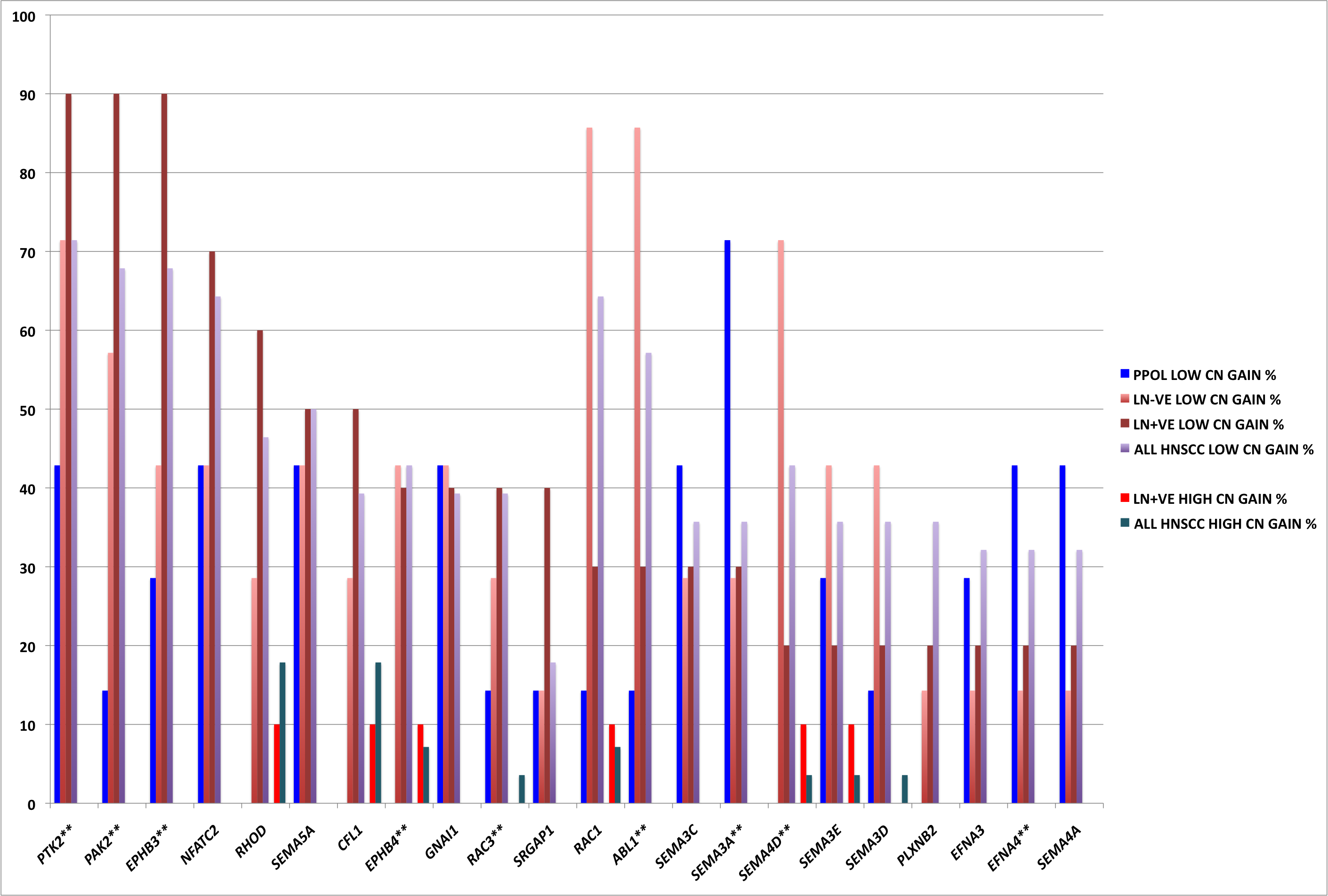


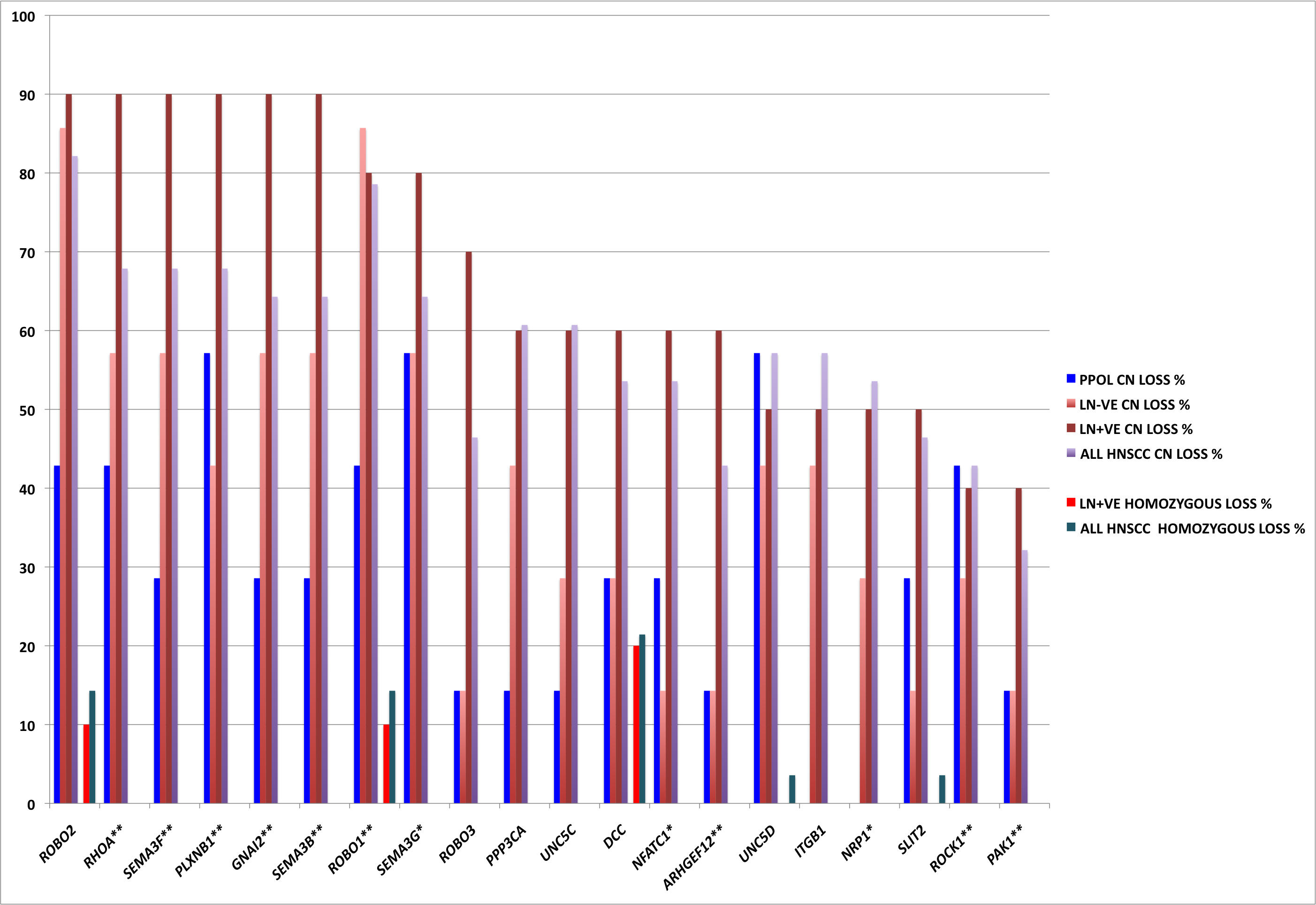


**Figure 4. Cell Adhesion Pathway**


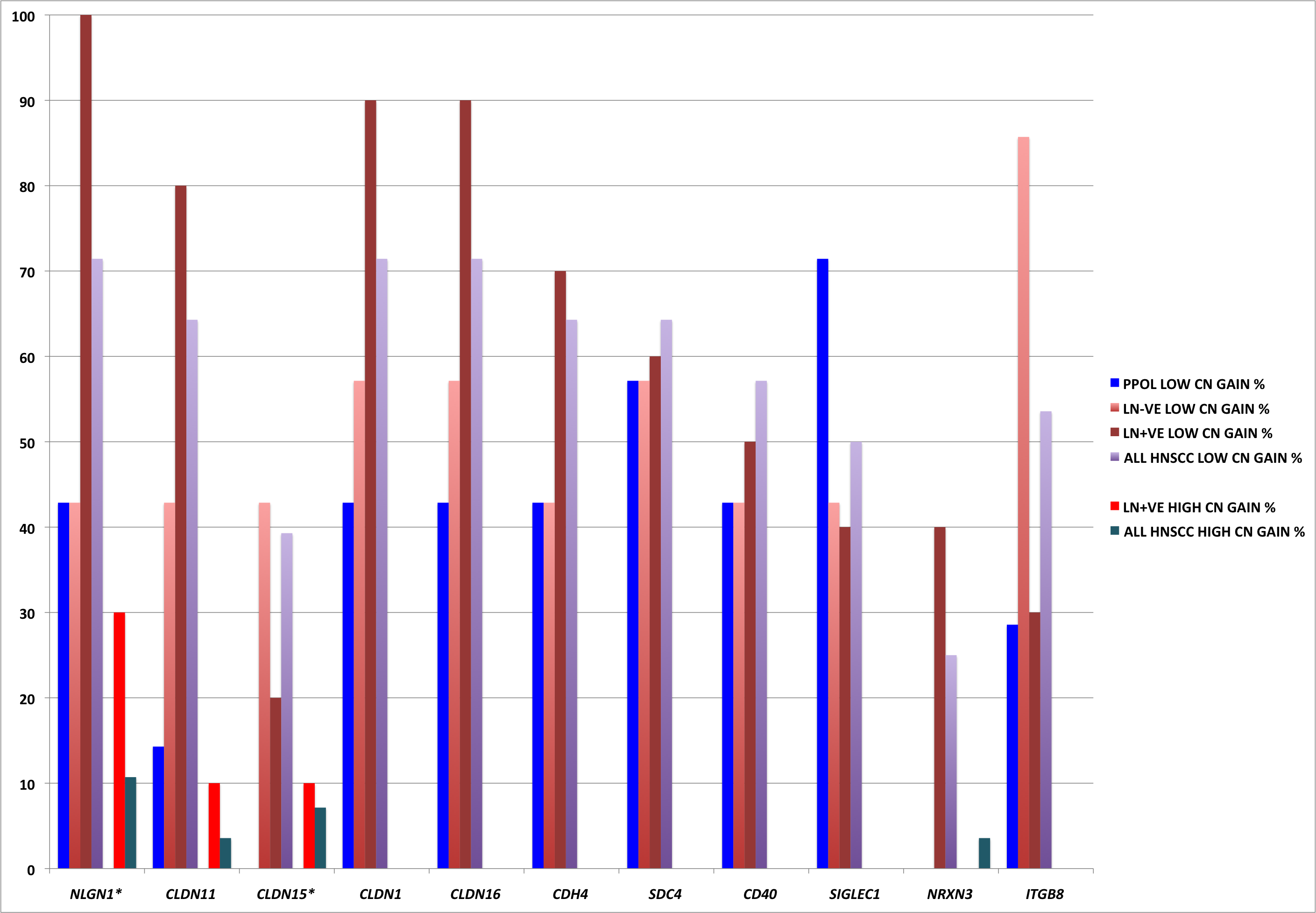


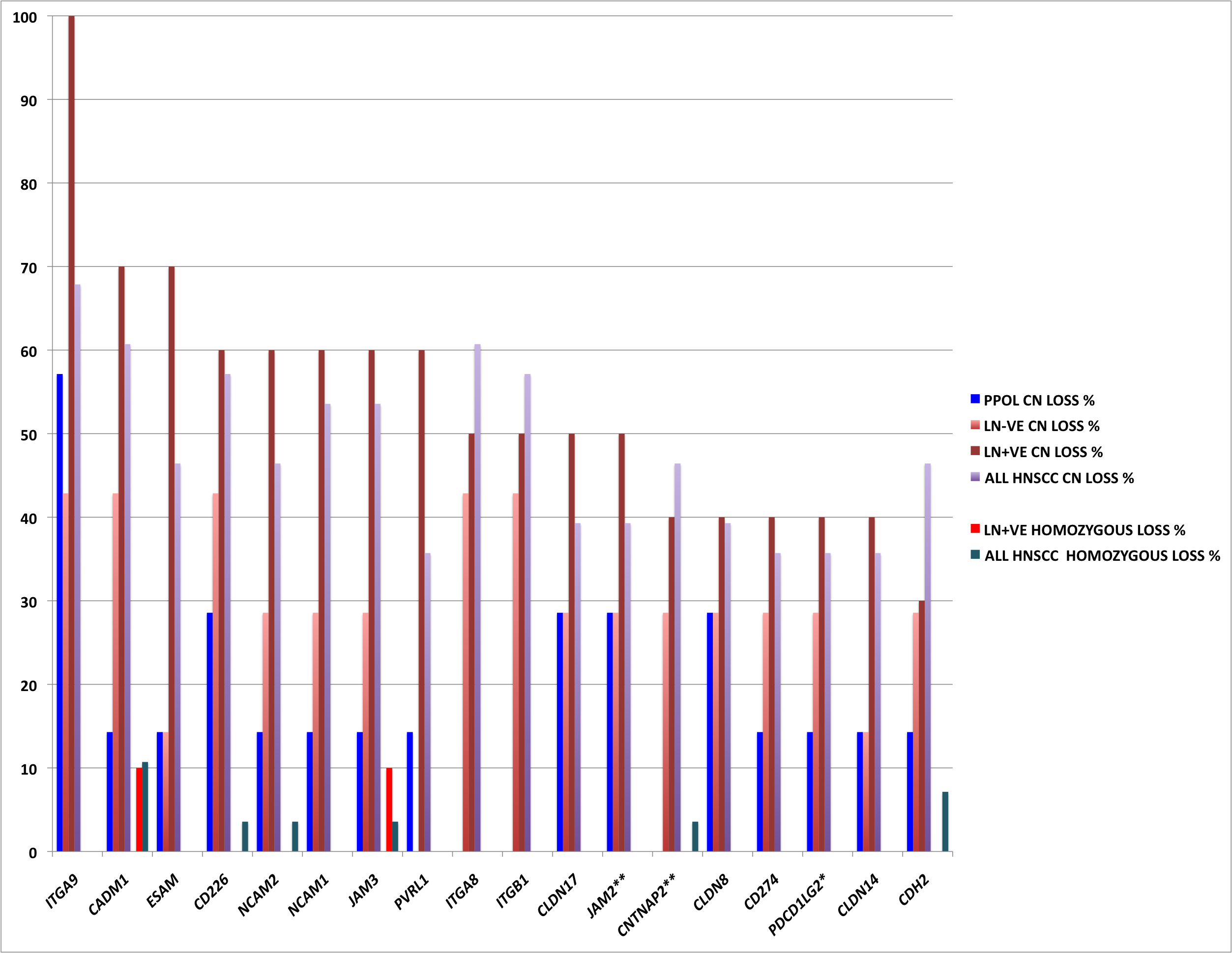


**Figure 5. Cell Cycle Pathway**


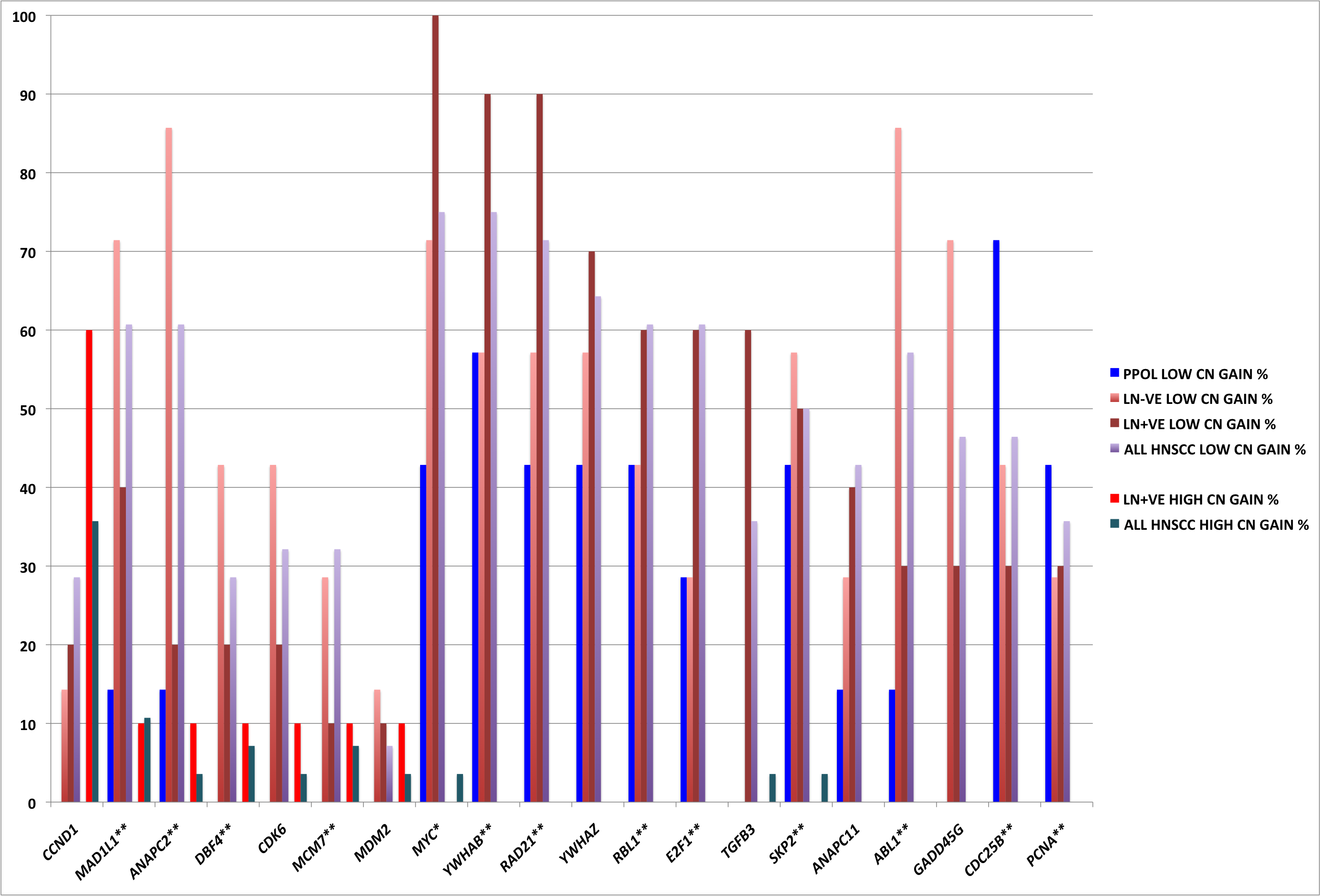


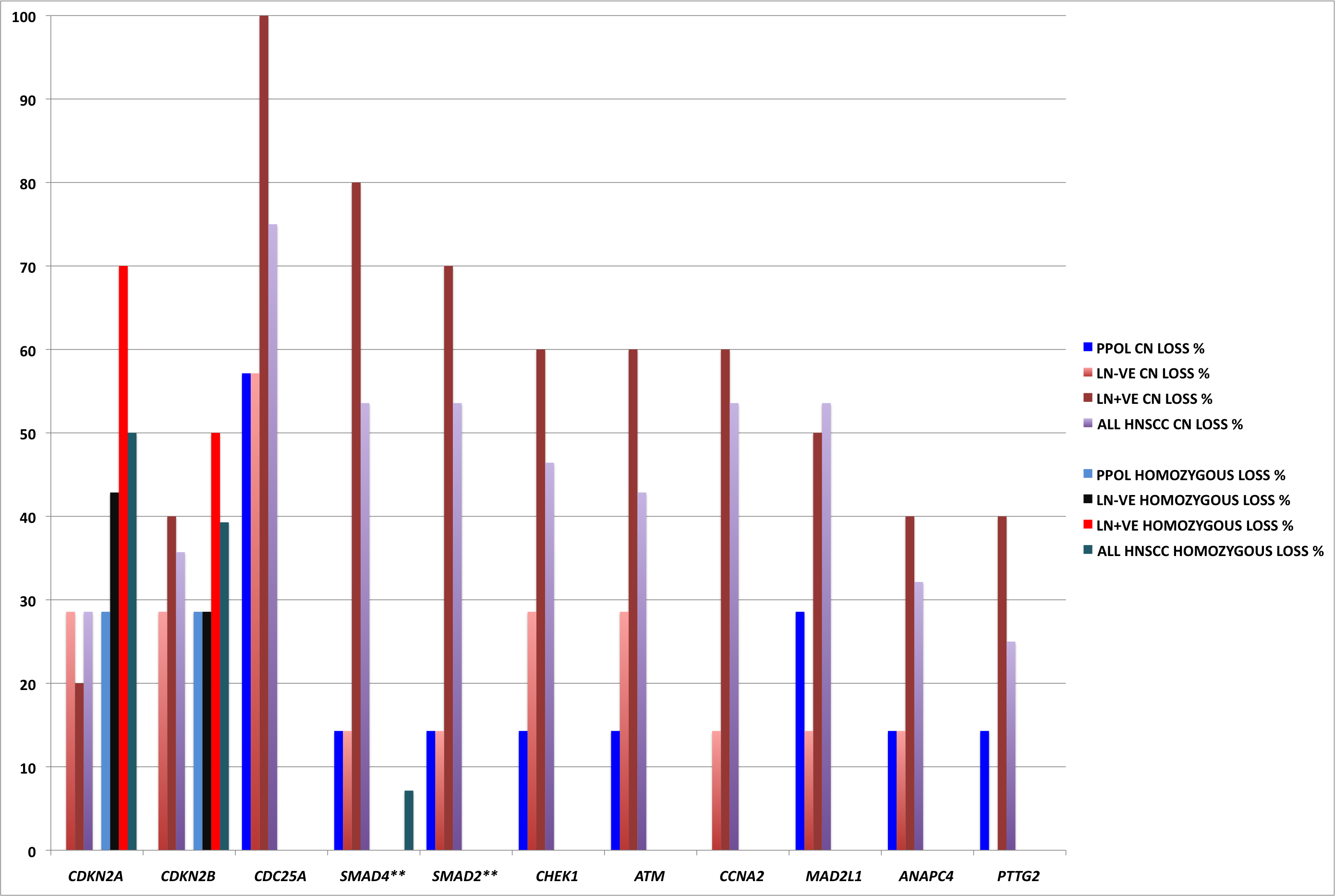


**Figure 6. Endocytosis Pathway**


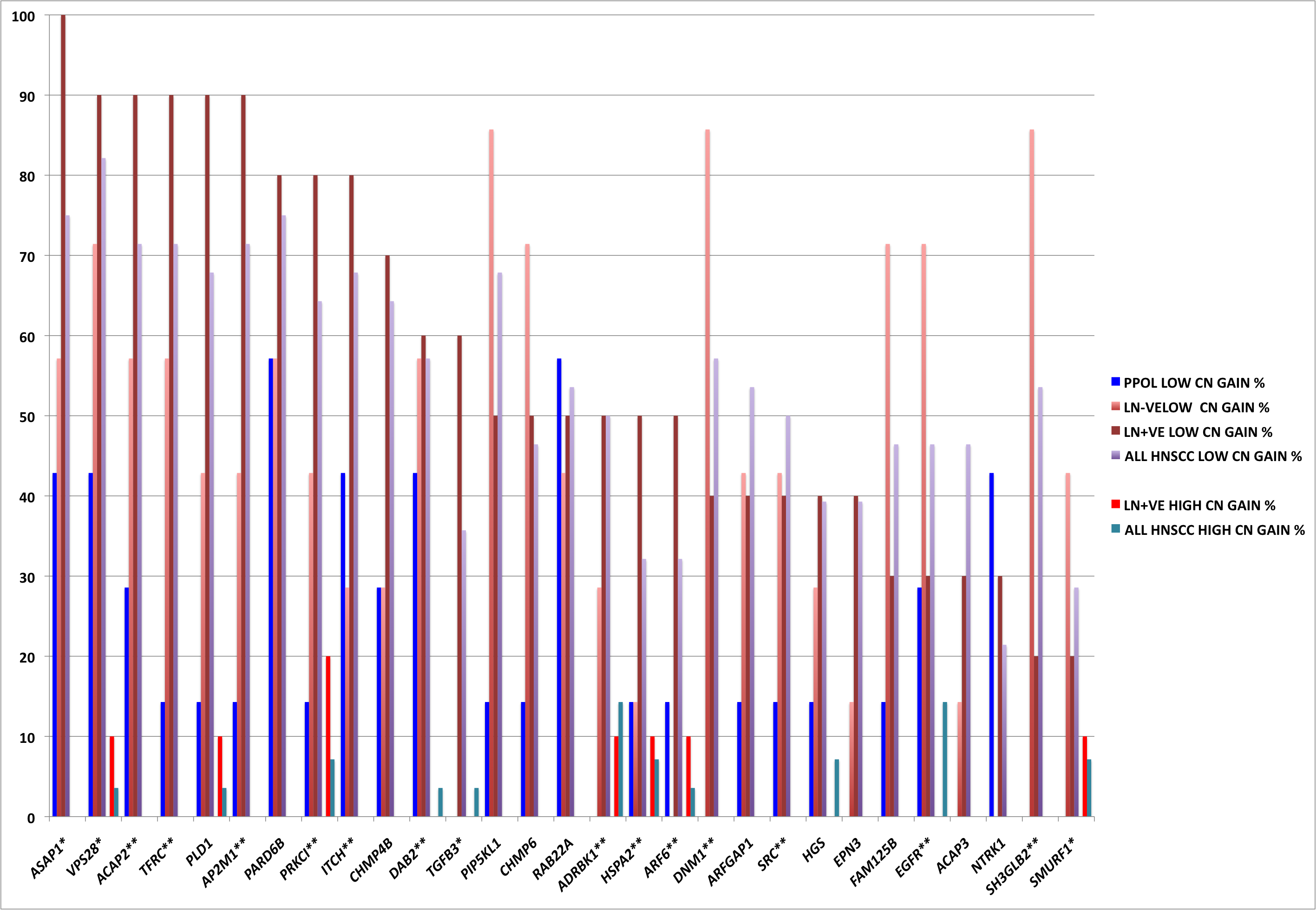


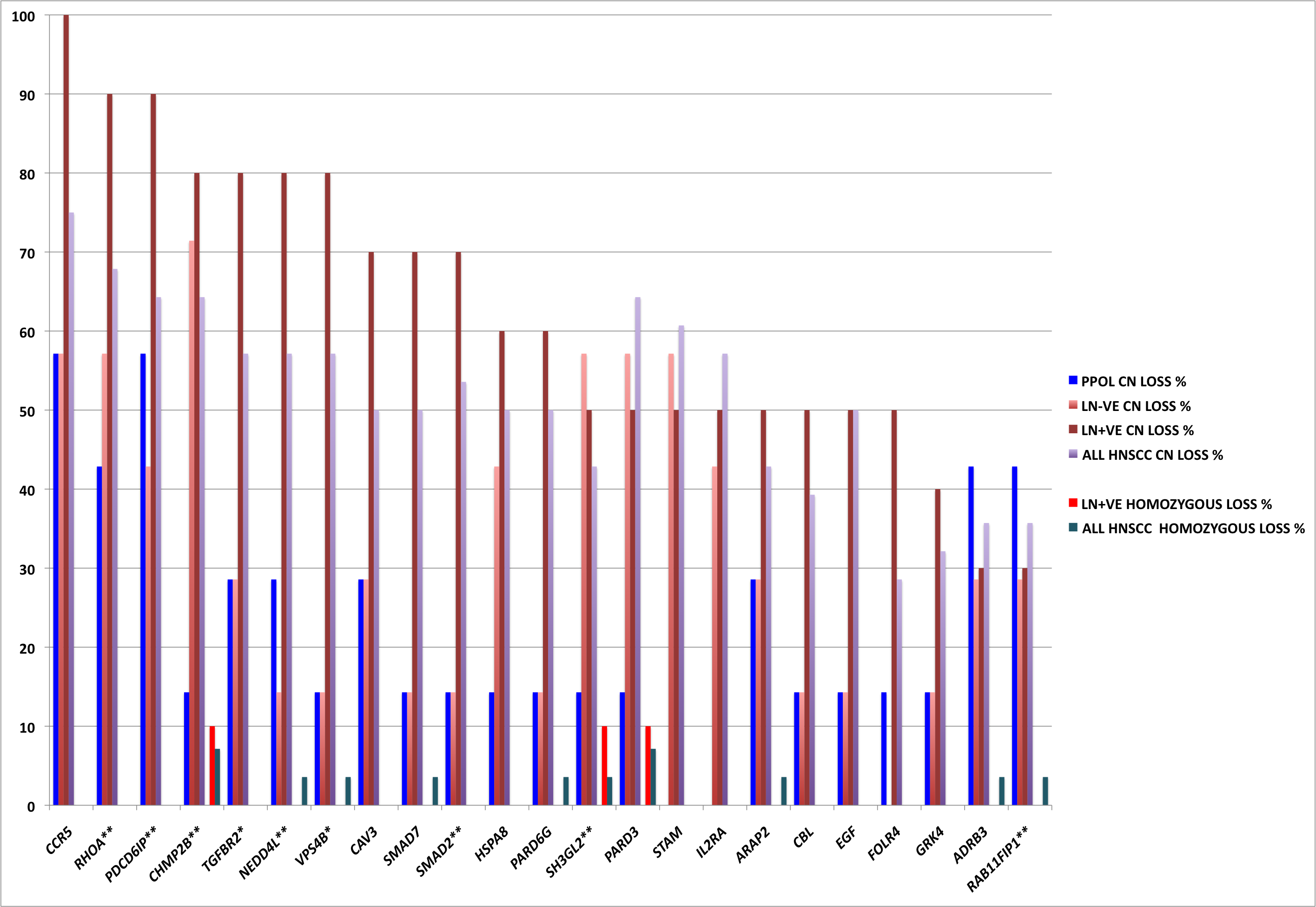


**Figure 7. Jak-STAT Pathway**


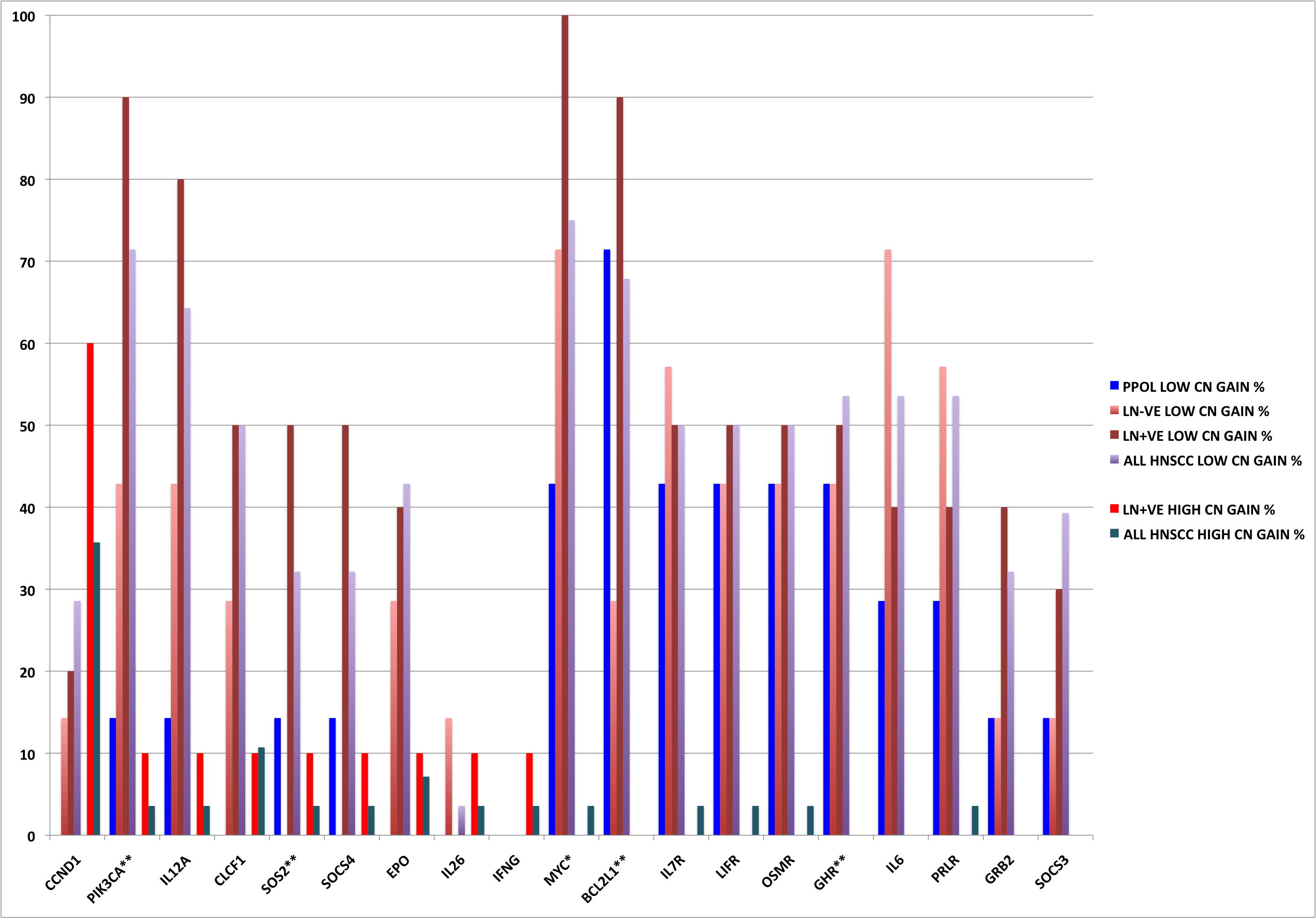


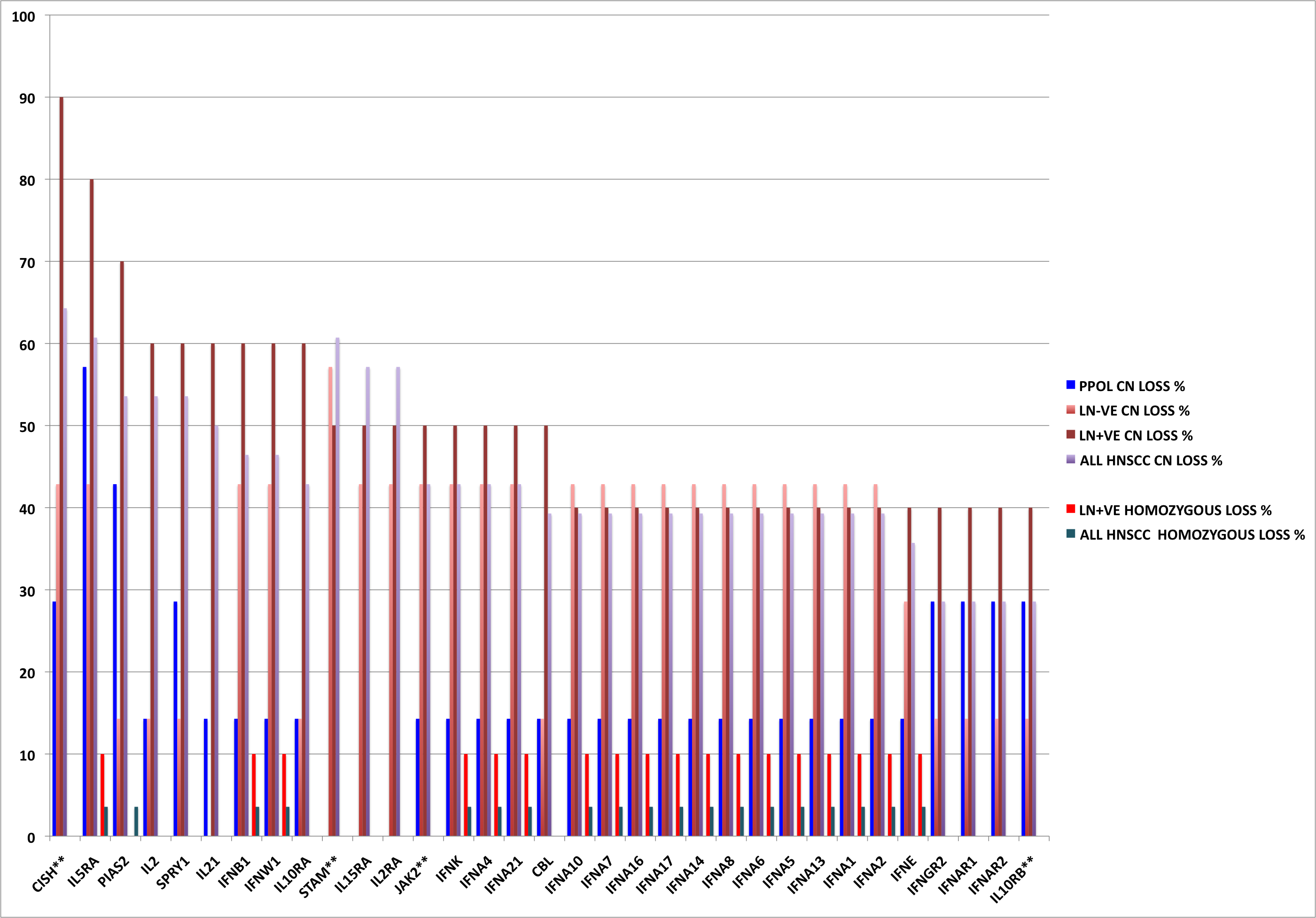


**Figure 8. MAPK Pathway**


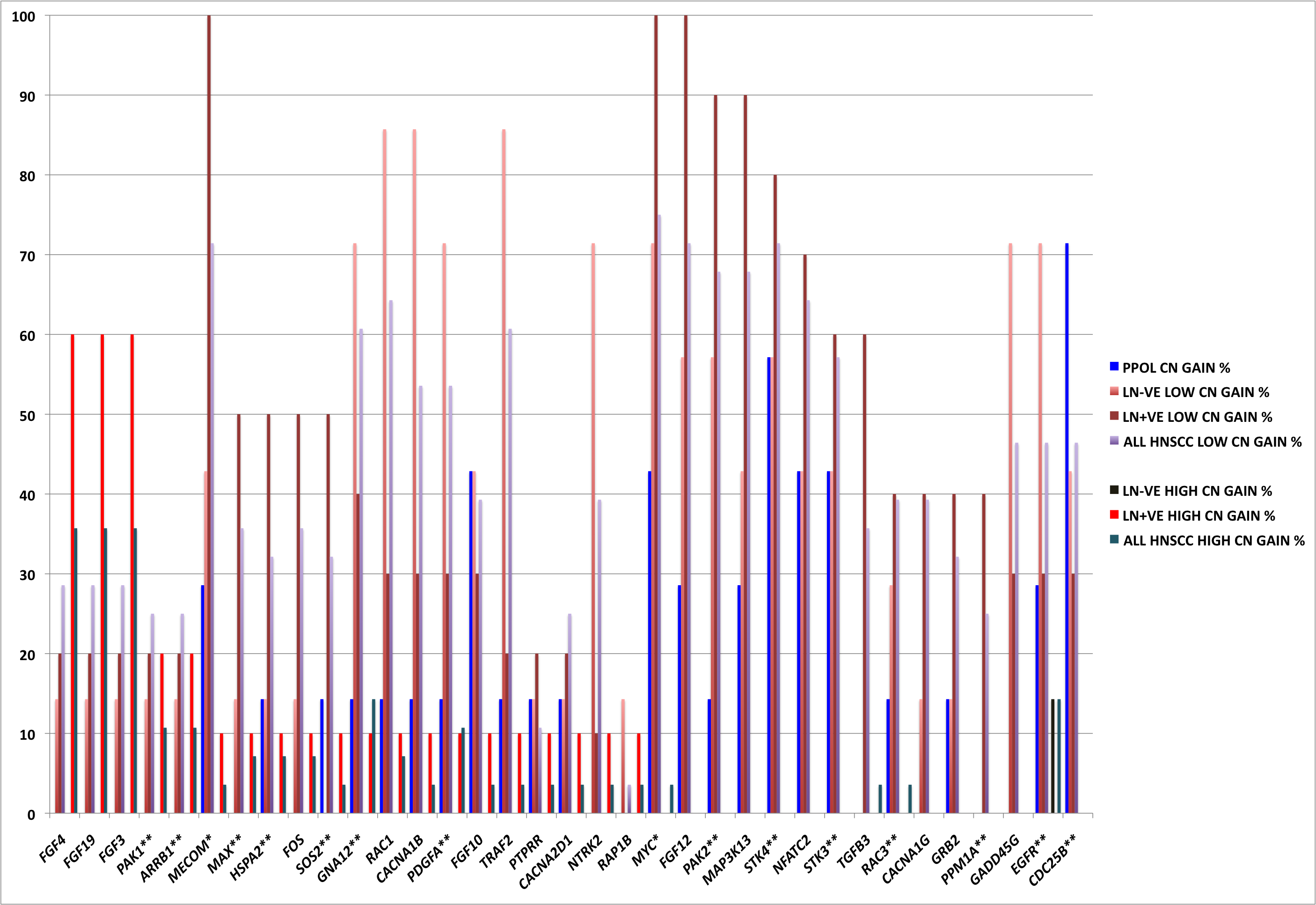


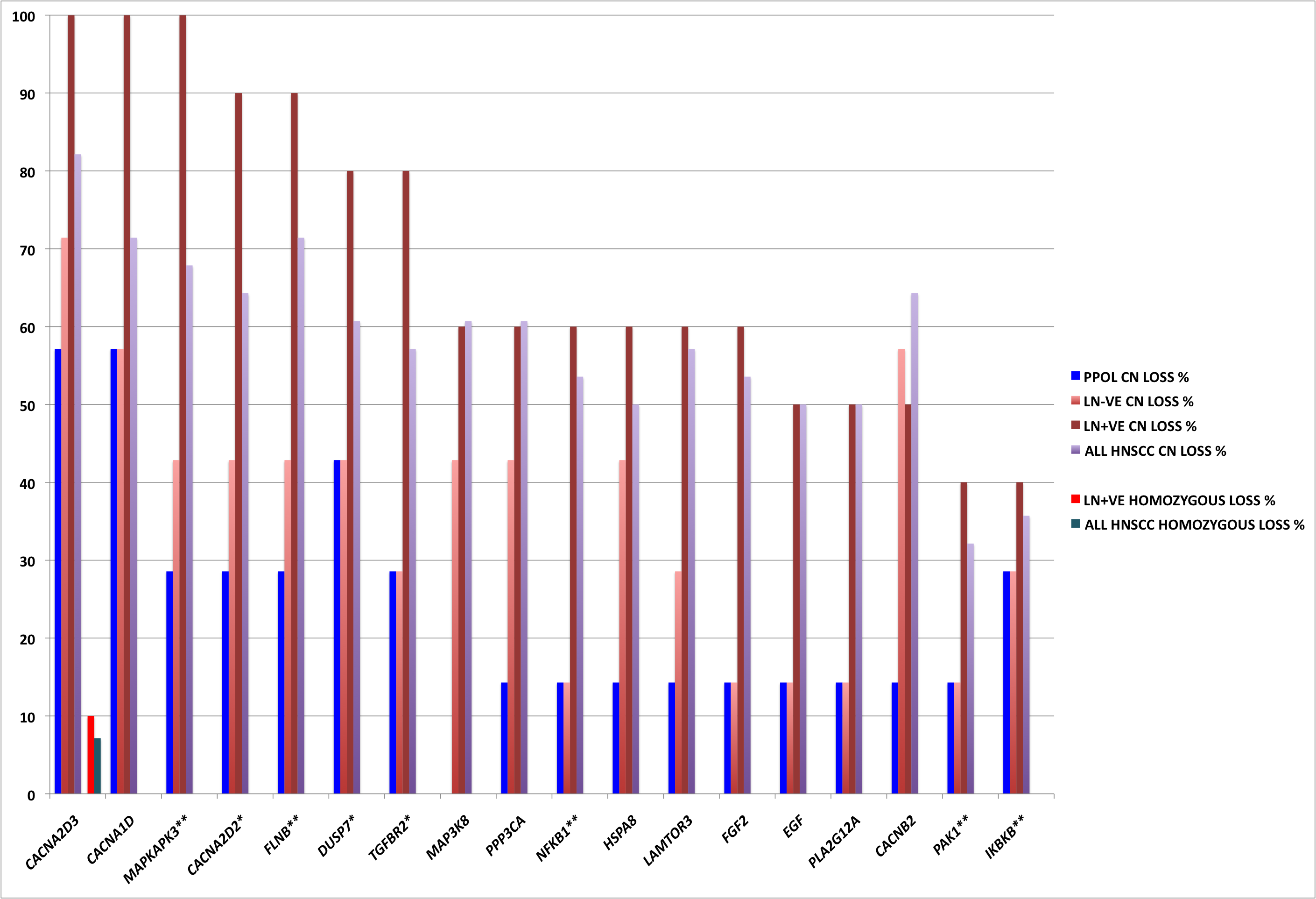


**Figure 9. Ubuiquitin-Proteosome Pathway**


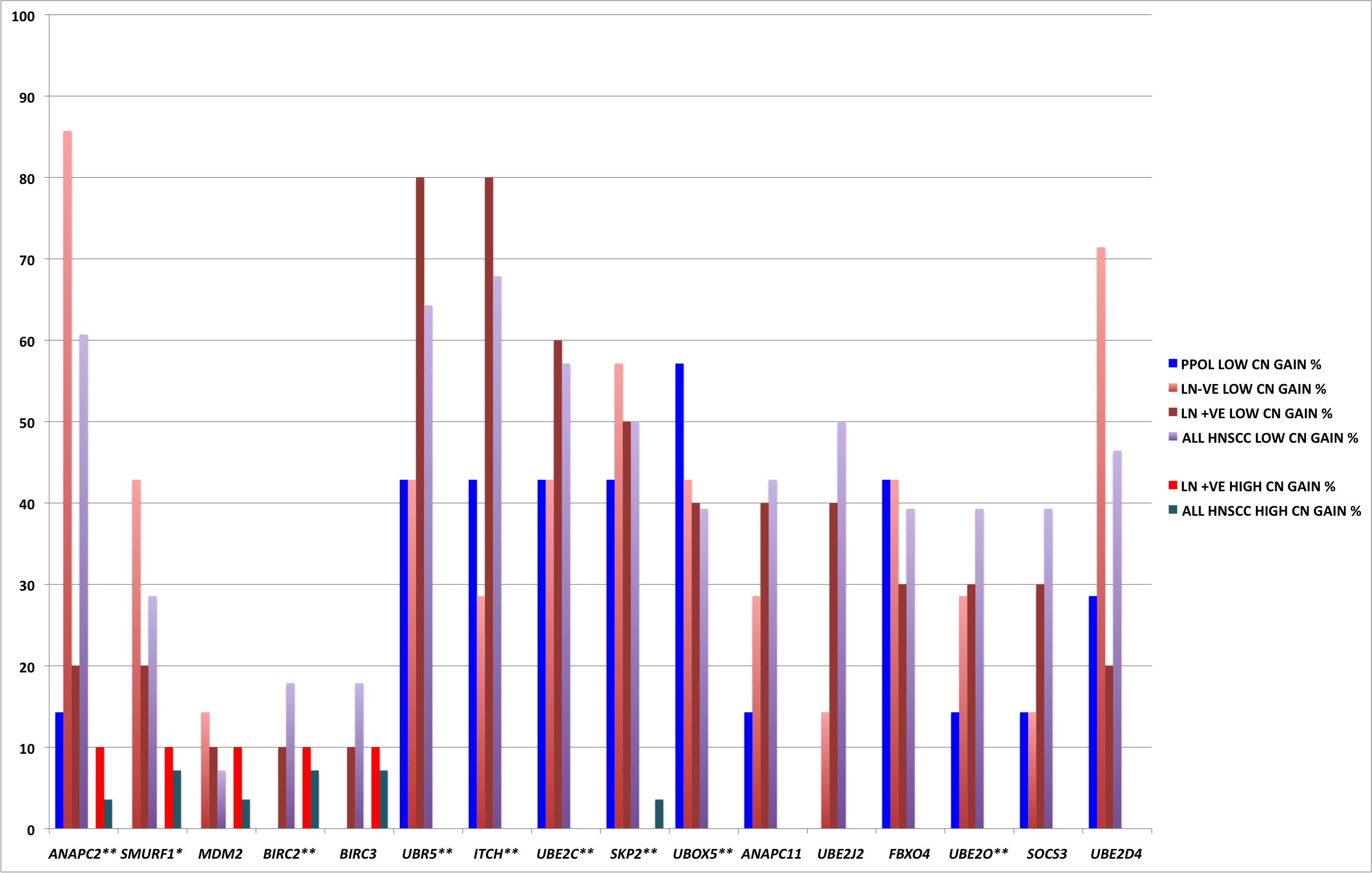


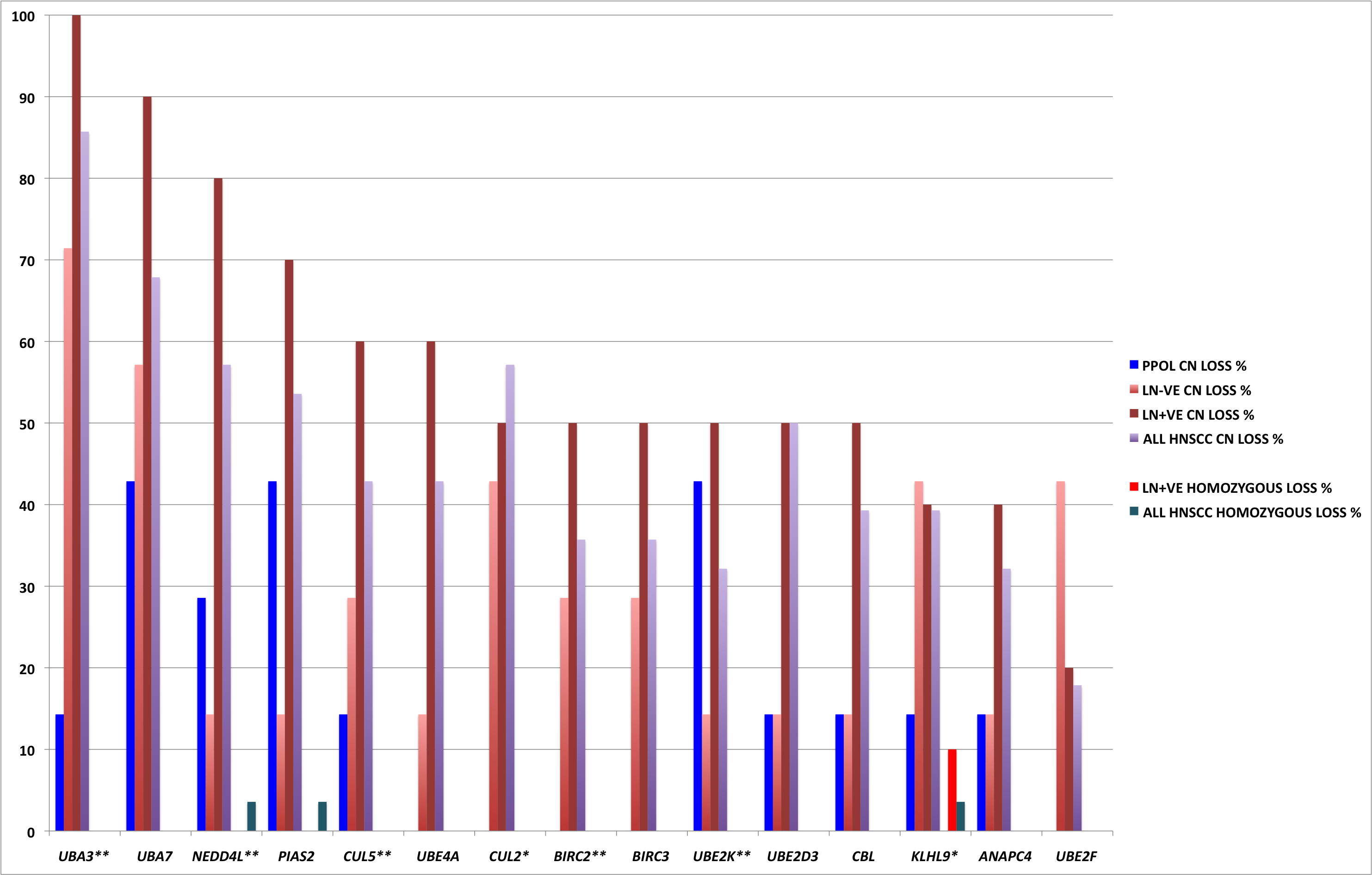


**Figure 10. WNT Pathway**


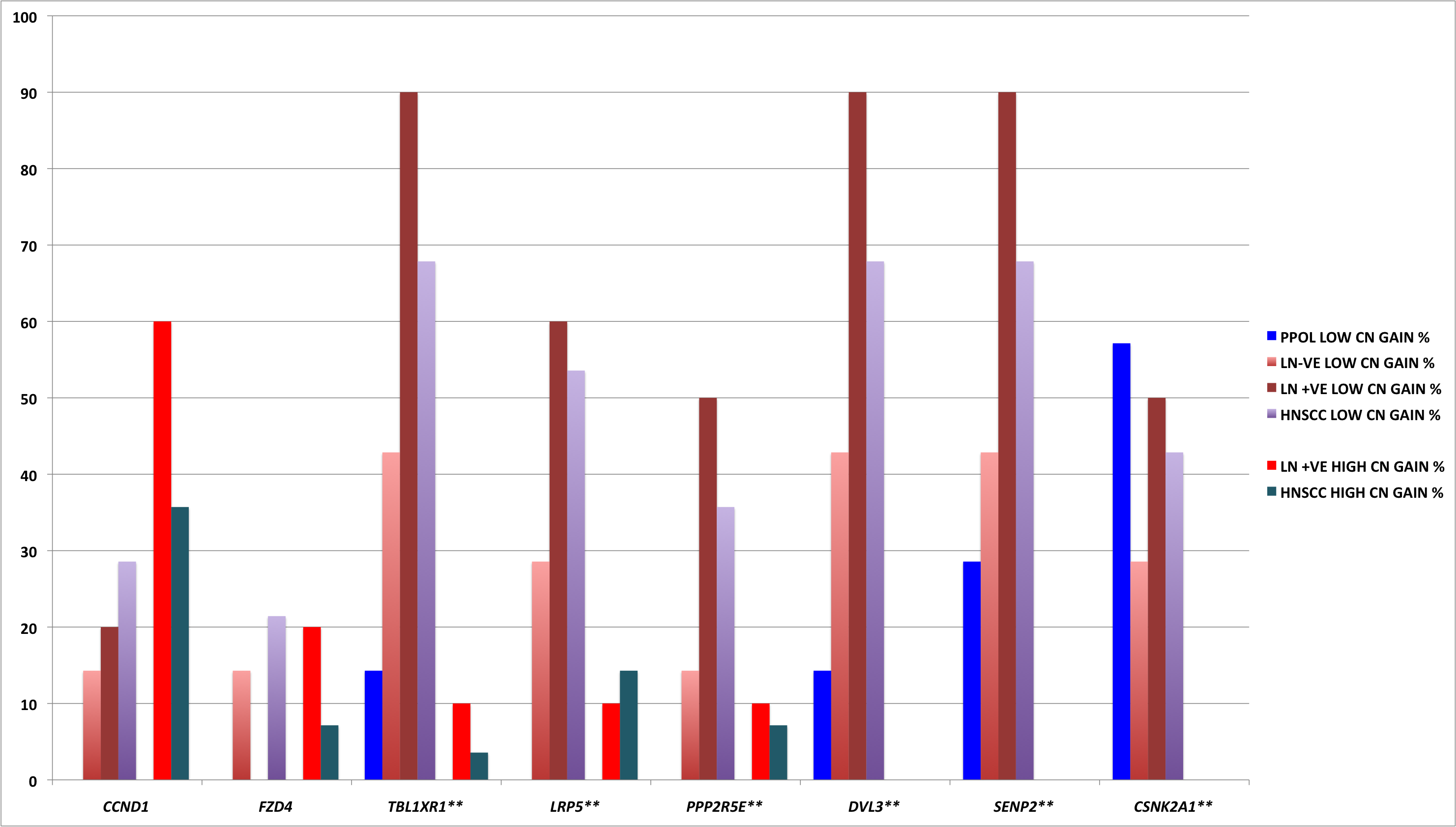


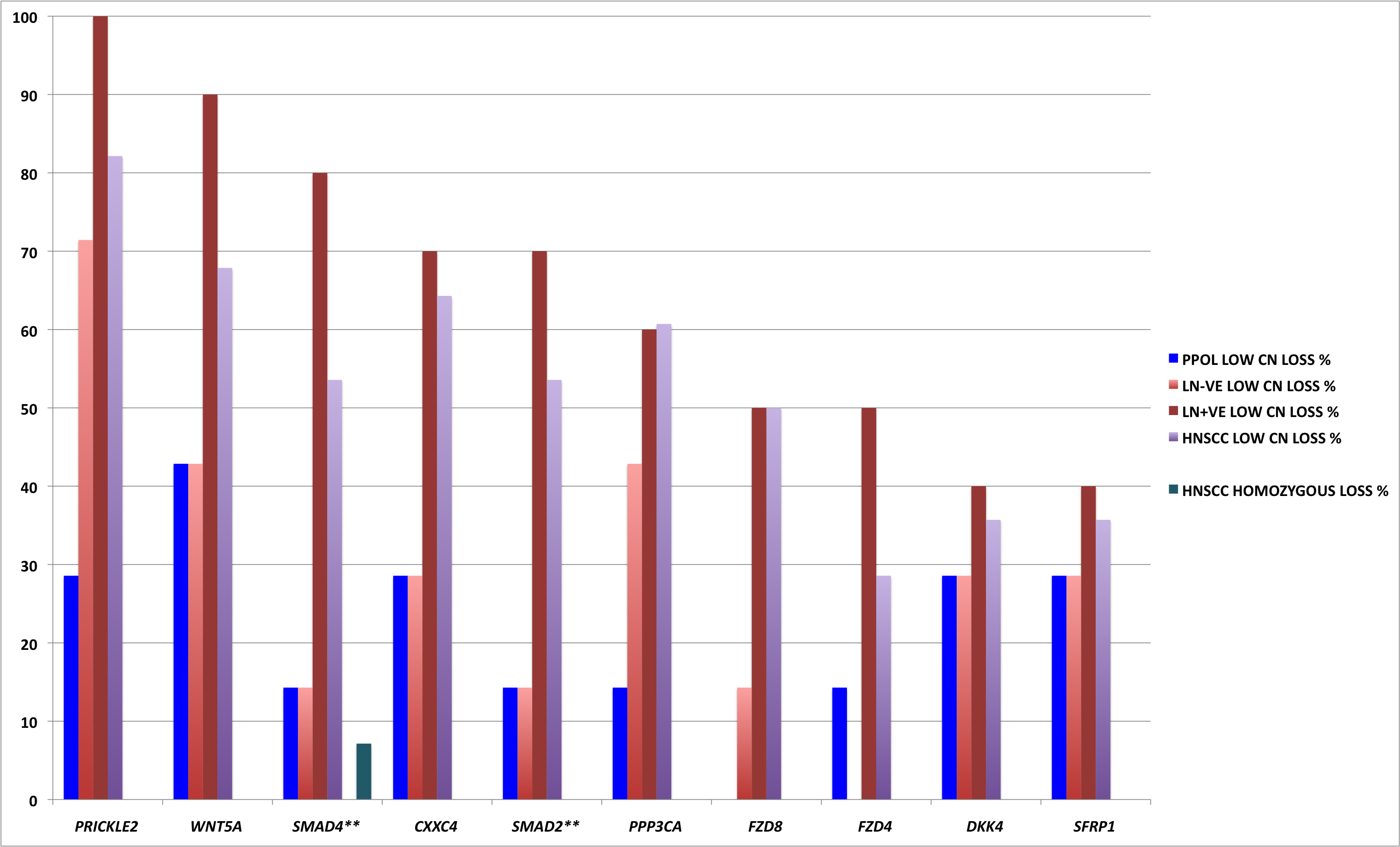


**Figure 10. TGFB Pathway**


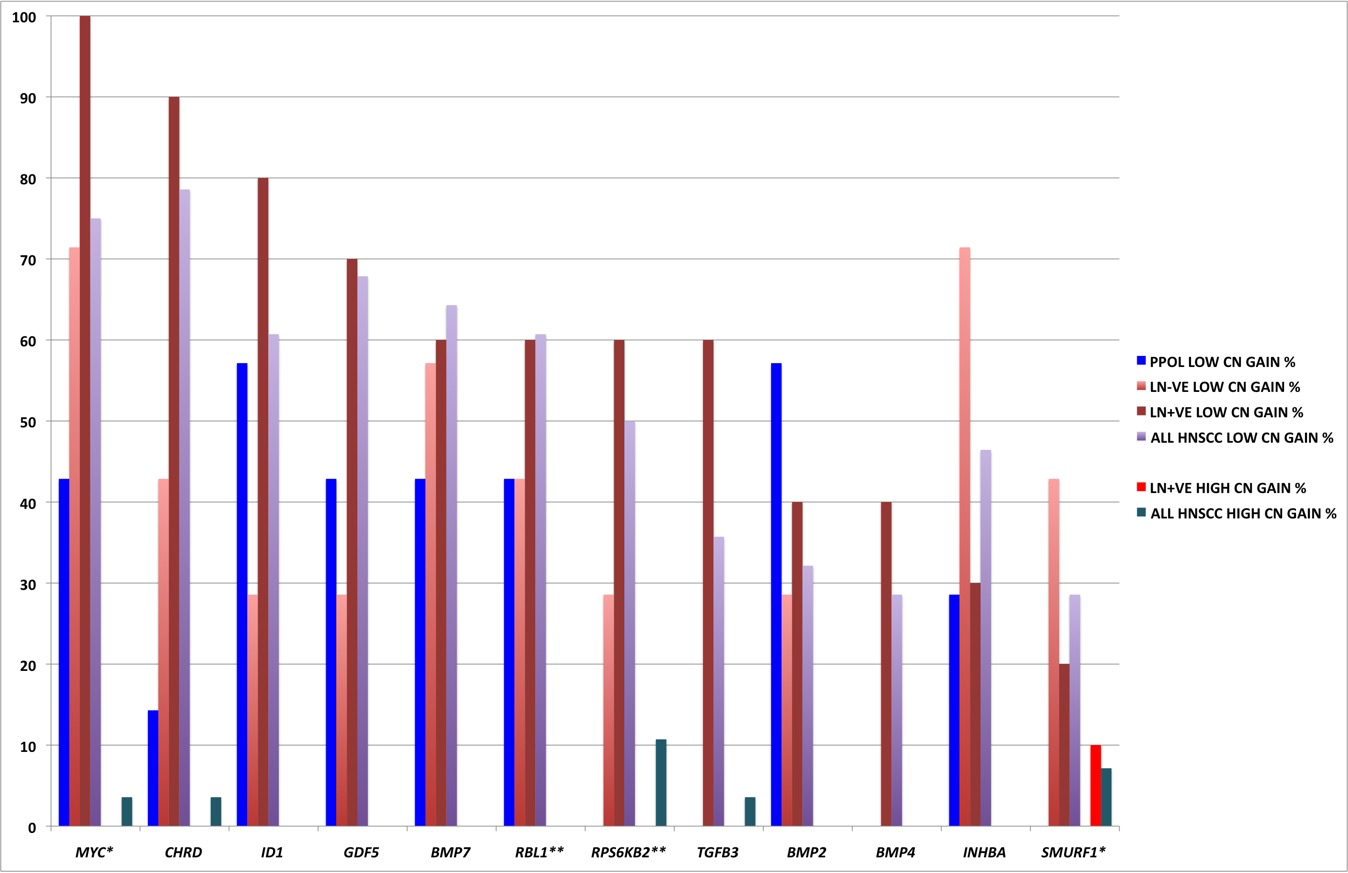


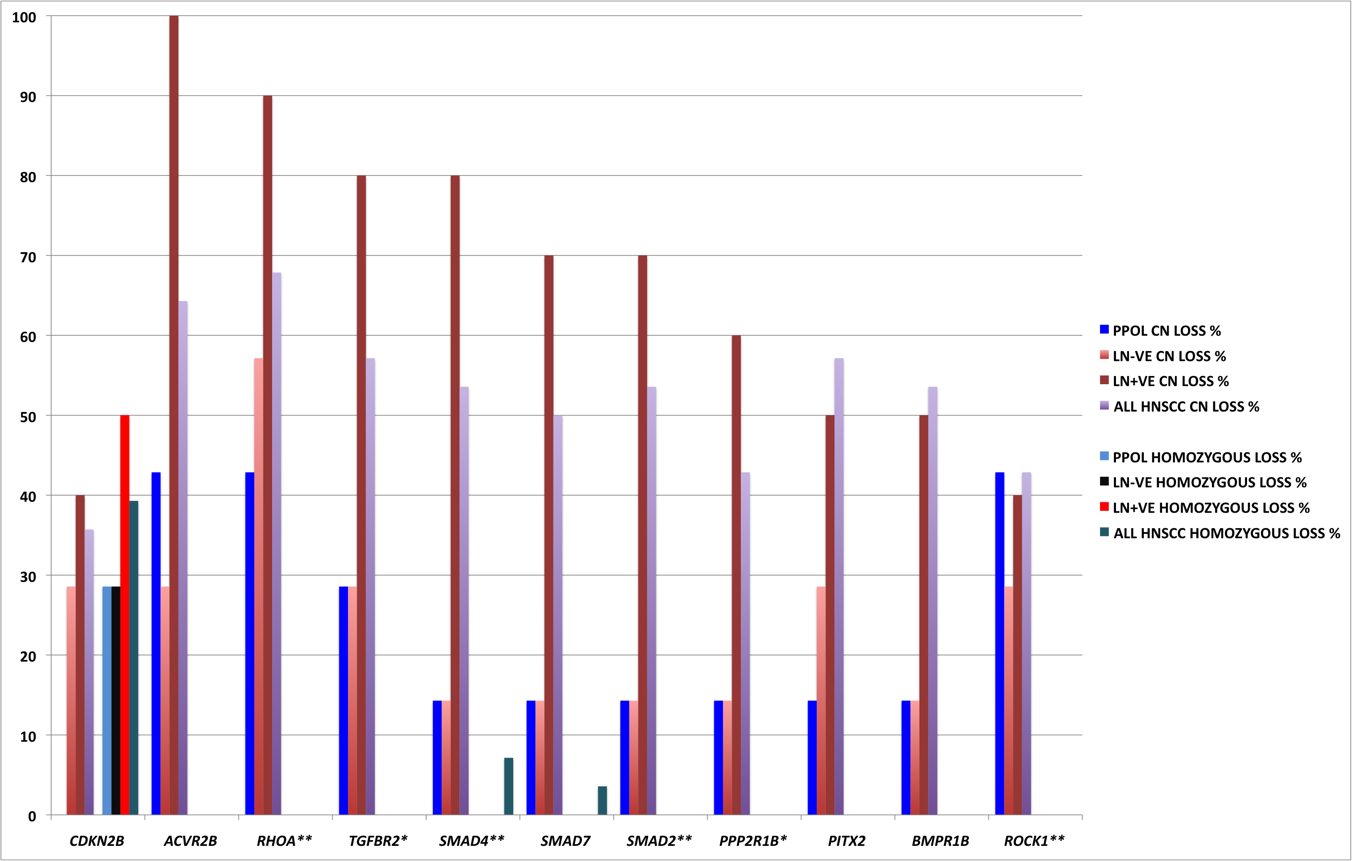

Supplement: Supplementary file 1 — Supplementary Data S1-4 & S6-19 [file 41598_2019_48229_MOESM1_ESM.doc]
