# Supplementary material for: Analysis of head and neck carcinoma progression reveals novel and relevant stage-specific changes associated with immortalisation and malignancy
Source: Sci Rep. 2019 Aug 19;9:11992. doi: 10.1038/s41598-019-48229-7 (PMC6700135; doi:10.1038/s41598-019-48229-7)

SUPPLEMENTARY DATA S1-S4, S6-S19

Analysis of head and neck carcinoma progression reveals novel and relevant stage-specific changes associated with immortalisation and malignancy.

Ratna Veeramachaneni, Thomas Walker, Timothée Revil, Antoine De Weck, , Dunarel Badescu, James O'Sullivan, Catherine Higgins, Louise Elliott, Triantafillos Liloglou, Janet M. Risk, Richard Shaw, Lynne Hampson, Ian Hampson, Simon Dearden, Robert Woodwards, Stephen Prime, Keith Hunter, Eric Kenneth Parkinson, Jiannis Ragoussis, Nalin Thakker

Supplementary Data- Contents

No	SUPPLEMENTARY DATA	PAGE
S1	DETAILS OF STUDY SAMPLES	3
S2	CUMULATIVE DISTRIBUTION OF COVERAGE SHOWING THE FRACTION OF TARGETED BASES THAT WERE COVERED BY AT LEAST A CERTAIN DEPTH IN EXOME SEQUENCING	6
S3	CUMULATIVE DISTRIBUTION OF COVERAGE SHOWING THE FRACTION OF TARGETED BASES THAT WERE COVERED BY AT LEAST A CERTAIN DEPTH IN HALOPLEX SEQUENCING	7
S4	FILTERING OF SEQUENCE VARIANTS DETECTED BY EXOME AND HALOPLEX CAPTURE	8
S6	COMPARISION OF SCNAS IN TWO HNSCC PANELS	9
S7	SUBTRACTION COPY NUMBER FREQUENCY PLOTS OF MORTAL PPOLS AND MATCHING FIBROBLASTS	10
S8	IMMORTAL PPOL CELL LINES SCNA DENSITY PLOTS FOR CHROMOSOMES 3, 8, 9 AND 20	11
S9	HIERARCHICAL CLUSTER ANALYSIS OF IMMORTAL HNSCC CELL LINES FOR HIGH COPY NUMBER SCNA - ASSOCIATION WITH LYMPH NODE METASTASES	12
S10	GENES IN GISTIC PEAK REGIONS IN PPOLS AND HNSCC CELL LINES	13
S11	COPY NUMBER GAINS AND LOSSES OF THE TOP RANKED GENES IN TOP RANKED PEAKS REGIONS OF FOCAL DELETIONS IDENTIFIED BY BEROUKHIM ET AL., 2010	25
S12	FREQUENCY OF COPY NUMBER ALTERATIONS OF INTOGEN-DERIVED CANCER DRIVERS GENES IN PPOLS AND HNSCC CELL LINES	29
S13	DELETIONS IN HNSCC CELL LINES AT THE CSDM1 LOCUS	40
S14	MODULATION OF CSMD1 EXPRESSION IN IMMORTAL HNSCC CELL LINES	41
S15	ANALYSIS OF NCKAP5	42
S16	INTEGRATIVE ANALYSIS OF SOMATIC COPY NUMBER CHANGES AND GENE EXPRESSION	49
S17	ANALYSIS OF CLDN1 AND BCL2L1 EXPRESSION IN PRIMARY HNSCC AND PPOL	55
S18	KEGG PATHWAY GENE ENRICHMENT IN GISTIC REGIONS	59
S19	SCNA OF GENES IN CANCER REALTED KEGG PATHWAYS ENRICHED IN GISTIC REGIONS	60

Supplementary Data S1 – Details of Study Samples

	Biopsy			Patient			Culture
**Name**	**Site**	**lesion**	**stage**	**smoking**	**age**	**sex**	**Lifespan (PDs)**
**Mortal PMOLs**							
D6	PT	Leukoplakia	mod/severe	y	55	M	25
D8	FOM	Leukoplakia	mild/mod	y	71	M	9
D25	FOM	Leukoplakia	severe	y	58	M	28
D30	FOM	Leukoplakia	mild	y	52	M	30
D47	FOM	Leukoplakia	mod	y	82	F	20
D48	FOM/VT	Leukoplakia	mod/severe	y	62	F	25.5
D17 (EL)	BM	Leukoplakia	mild/mod	y	61	M	61
E1	T	Erythroplakia	CIS	U	U	M	24
E2	ALV	Erythroplakia	CIS	U	U	M	17
E4	LT	Erythroplakia	CIS	U	65	F	41
E5	LT	Erythroplakia	severe	U	61	M	31
**Immortal PMOLs**							
D4	FOM/VT	Leukoplakia	CIS	y	51	M	>100
D9	VT	Leukoplakia	mild/mod	n	84	M	>100
D34	LT	Leukoplakia	mod	n	54	F	>100
D38	LT	Leukoplakia	mild	n	55	F	>100
D19	LT	erythroleukoplakia	Severe/CIS	y	53	M	>100
D20	LT	Leukoplakia	moderate	n	50	M	>100
D35	FOM/VT	erythroleukoplakia	Severe/CIS	y	68	M	>100
**Mortal OSCCs**							
BICR 66 (B66)	RM/T	Carcinoma	T2N0M0	y	56	M	37
BICR 80 (B80)	LNX	Carcinoma	T4N2CM0	y	71	M	62
BICR 37 (B37)	Primary:T	Metastasis	T4N2CM0	U	U	F	32
**Immortal OSCCs**							
BICR 3 (B3)	ALV	Carcinoma	T2N0M0	y	56	F	>130
BICR 31 (B31)	T	Carcinoma	T4N2BM0	U	U	M	>130
BICR 56 (B56)	T	Carcinoma	T4N1M0	y	59	F	>130
BICR 68 (B56)	BT	Carcinoma	T4N0M0	n	75	F	>130
T4 (T4)	FOM	Carcinoma	T4N0	U	U	F	>130
T5 (T5)	BM	Carcinoma	T2N2	Y	59	F	>130
BICR 78 (B78)	ALV	Carcinoma	T4N1M0	U	68	M	>130
BICR 63 (B63)	T	Carcinoma	T2N2BM0	y	70	M	>130
BICR10 (B10)	BM	Recurrence	T4N0M0	n	84	F	>130
BICR16 (B16)	T	Recurrence	T2N0M0	y	49	M	>130
BICR82 (B82)	MX	Recurrence	N.D.	y	48	M	>130
BICR 22 (B22)	Primary:T	Metastasis	T4N3M0	U	88	M	>130
BICR 18 (B18)	Primary:LN	Metastasis	T4N1M0	U	U	U	>130

Table 1: Clinical data of the PPOLs and HNSCC cell lines. Key: ALV, alveolus; BM, buccal mucosa; BT, base of tongue; CIS, carcinoma in situ; FOM, floor of mouth; HP, hypopharynx; LN, lymph node; LNX, larynx; LT, lateral tongue; MX, maxilla; N/A, not applicable; N.D., not determined; normal adjacent, apparently normal mucosa from patient with SCC; PT, posterior tongue; RM, retromolar trigone; T, tongue; U, information not available; VT, ventral tongue. The tumour names given in brackets are the abbreviations used in the text (adapted from Hunter, *et al.*, 2006)

Sample ID	Description
E2N	Matching fibroblasts of E2
E5N	Matching fibroblasts of E5
D19N	Matching fibroblasts of D19
BICR80N	Matching fibroblasts of BICR80
BICR37N	Matching fibroblasts of BICR37
BICR66N	Matching fibroblasts of BICR66
BICR3N	Matching fibroblasts of BICR3
BICR31N	Matching fibroblasts of BICR31
BICR56N	Matching fibroblasts of BICR56
BICR82N	Matching fibroblasts of BICR82
BICR68N	Matching fibroblasts of BICR68
BICR18N	Matching fibroblasts of BICR18
BICR78N	Matching fibroblasts of BICR78
BICR63N	Matching fibroblasts of BICR63
BICR22N	Matching fibroblasts of BICR22
H314K	HNSCC cell line
H413K	HNSCC cell line
H357K	HNSCC cell line
H314F	Matching fibroblasts of H314K
H413F	Matching fibroblasts of H413K
H357F	Matching fibroblasts of H357K

Table 2: Matching fibroblast for the HNSCC and PPOL cell line panels

No	Gender	Age	Site	Subsite	pT	pN	ECS	HPV status
3230	M	73	OSCC	Tongue	2	1	Y	
3231	F	72	OSCC	missing	missing	missing	missing	
3232	M	44	OSCC	AFOM	1	0	_	
3233	M	55	OSCC	AFOM	2	2c	N	
3234	M	62	OSCC	Tongue	1	x	_	
3236	F	71	OSCC	Mand Alveolus	2	0	_	
3237	M	59	OSCC	Retromolar	2	2a	N	
3238	M	71	OSCC	Mand Alveolus	2	0	_	
3239	M	54	OSCC	AFOM	2	2b	N	
3240	F	39	OSCC	Maxilla	3	0	_	
3241	F	74	OSCC	AFOM	2	2b	Y	
3242	F	70	OSCC	Tongue	1	1	N	
3243	M	80	OSCC	Mand Alveolus	2	0	_	
3244	F	51	OPSCC	BOT	2	0	_	negative
3245	M	62	OSCC	AFOM	2	2b	N	
3247	M	54	OSCC	Retromolar	4	1	N	
3248	M	67	OSCC	AFOM	2	2b	Y	
3249	F	74	OPSCC	Tonsil	2	1	Y	negative
3312	M	50	OSCC	AFOM	2	0	_	
3313	F	64	OSCC	AFOM	4	0	_	
3314	M	missing	OSCC	AFOM	2	0	_	
3315	M	52	OSCC	AFOM	2	0	_	
3316	F	44	OSCC	Tongue	2	0	_	
3317	M	42	OPSCC	Tonsil	3	2b	Y	positive
3479	M	68	OSCC	AFOM	4	0	_	
3480	M	52	OSCC	Tongue	3	2b	N	negative
3481	M	73	OPSCC	Soft Palate	2	1	N	negative
3494	F	49	OPSCC	Soft Palate	2	0	_	
3495	M	32	OSCC	Tongue	2	1	N	
3497	F	74	OSCC	Tongue	2	0	_	
3499	F	81	OSCC	Tongue	4	2c	missing	
3500	M	61	OSCC	Tongue	2	0	_	
3501	M	75	OSCC	Tongue	3	0	_	
3502	F	67	OSCC	Retromolar	4	0	_	
3505	M	82	OSCC	Buccal	1	2b	Y	
3510	M	65	OSCC	AFOM	4	0	_	

Table 3: Primary HNSCC and matching adjacent normal mucosa samples used in pyrosequencing methylation and gene expression analyses

SUPPLEMENTARY DATA S2 – CUMULATIVE DISTRIBUTION OF COVERAGE SHOWING THE FRACTION OF TARGETED BASES (Y-AXIS) THAT WERE COVERED BY AT LEAST A CERTAIN DEPTH (X-AXIS) IN EXOME SEQUENCING

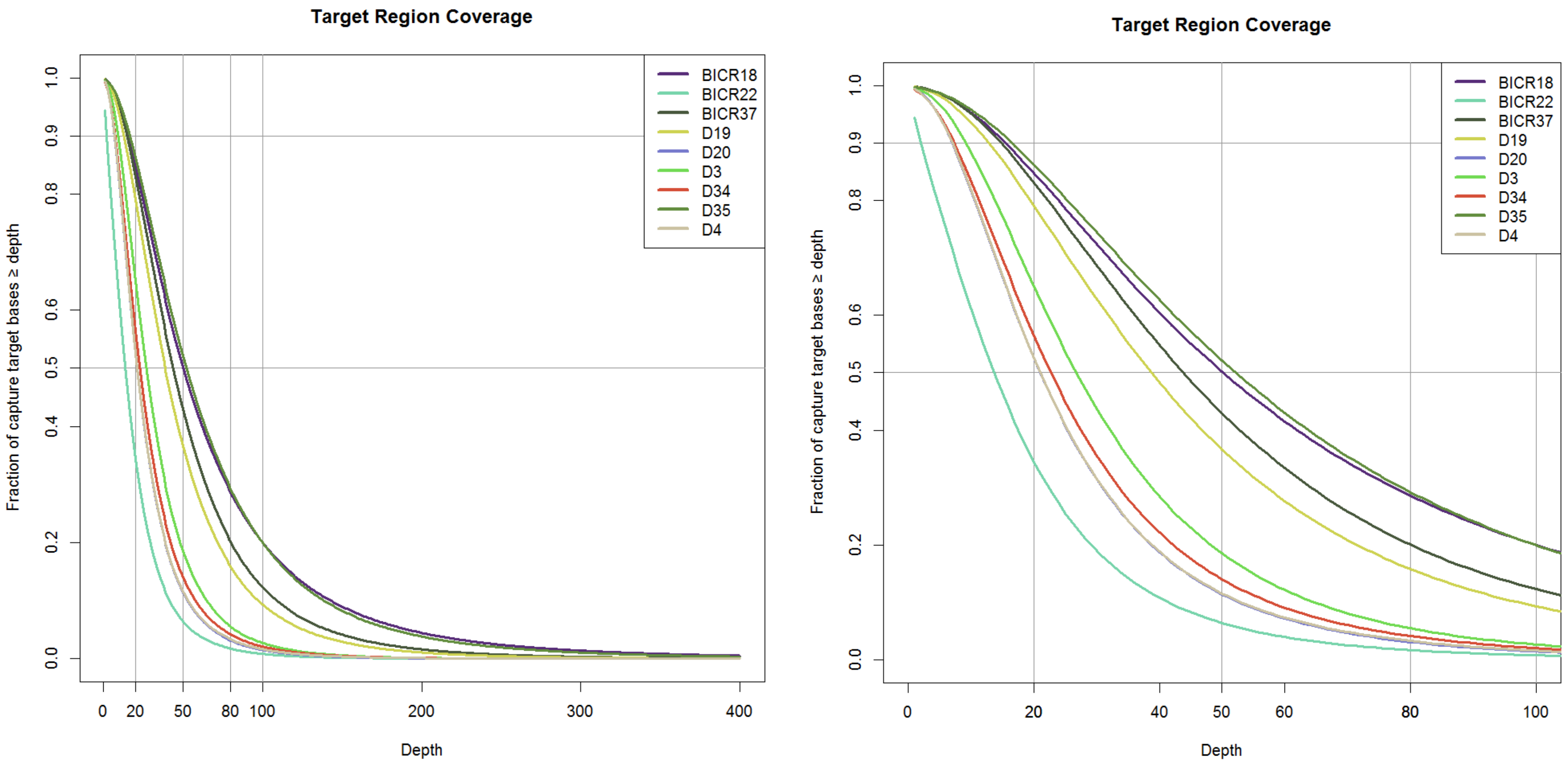

Cumulative distribution of coverage showing the fraction of targeted bases (y-axis) that were covered by at least a certain depth (x-axis). Left panel shows the full plot and the right panel shows an expanded plot to show x100 depth coverage.

SUPPLEMENTARY DATA S3 – CUMULATIVE DISTRIBUTION OF COVERAGE SHOWING THE FRACTION OF TARGETED BASES (Y-AXIS) THAT WERE COVERED BY AT LEAST A CERTAIN DEPTH (X-AXIS) IN HALOPLEX SEQUENCING

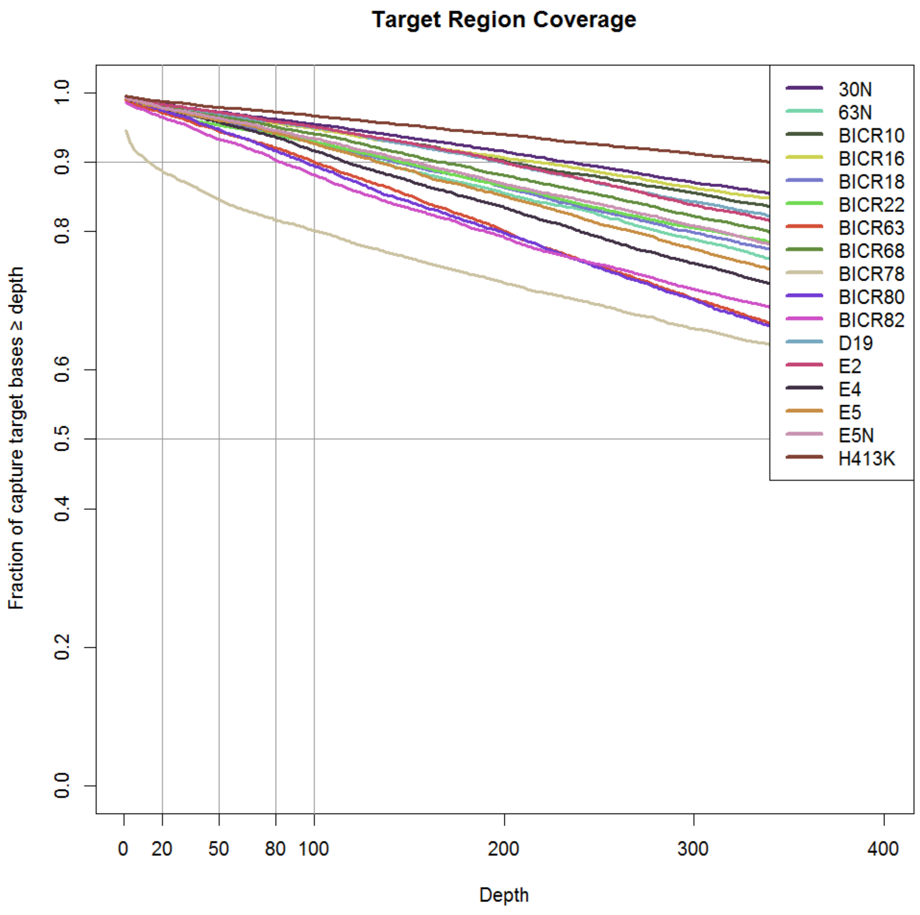

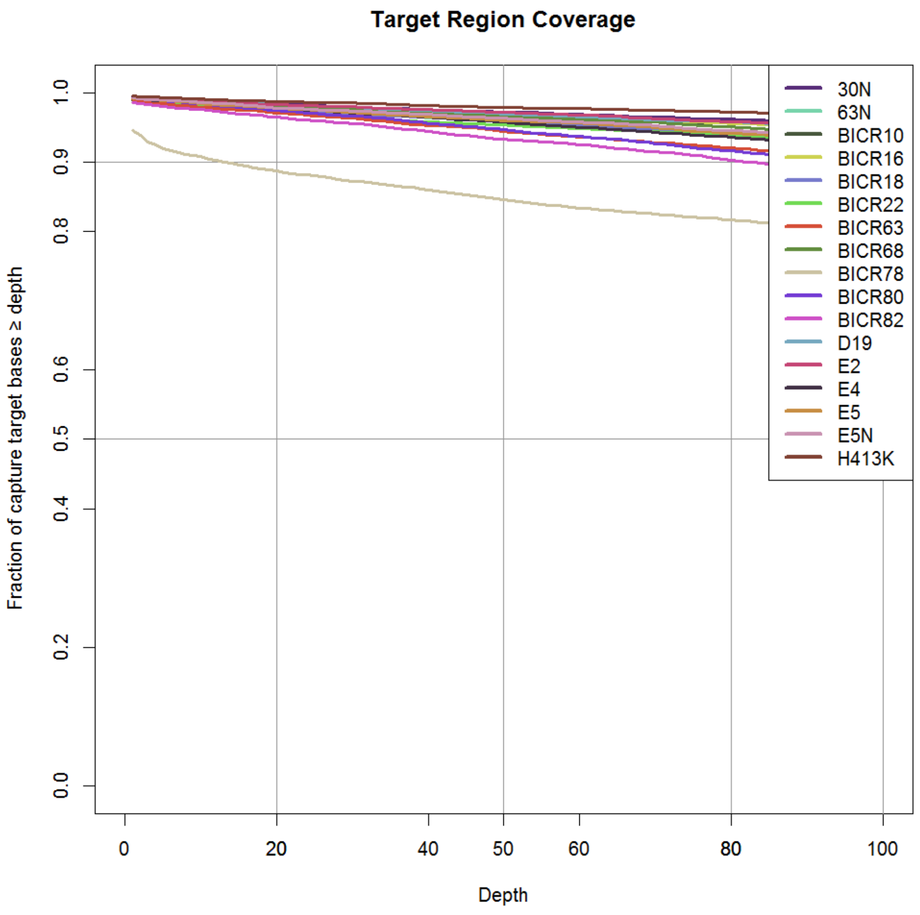

Sequence capture efficiency of HaloPlex sequencing panel. Left: The cumulative distribution of coverage showing the fraction of targeted bases (y-axis) that were covered by at least a certain depth (x-axis) is shown. Right: Zoomed-in 0-100 fold coverage range is shown in order to illustrate that with the exception of sample BICR78, all samples were covered at 80x for 90% of the interrogated sequences.

SUPPLEMENTARY DATA S4– FILTERING OF SEQUENCE VARIANTS DETECTED BY EXOME AND HALOPLEX CAPTURE

Capture	Unfiltered	ExAC filtering	Germline filtering	Coding variants	Unique variants	No High GDI genes
Exomes	461689	157440	56520	9701	5085	4493
HaloPlex	5042	2008	1189	248	63	53
Total	466731	159448	57709	9949	5148	4546

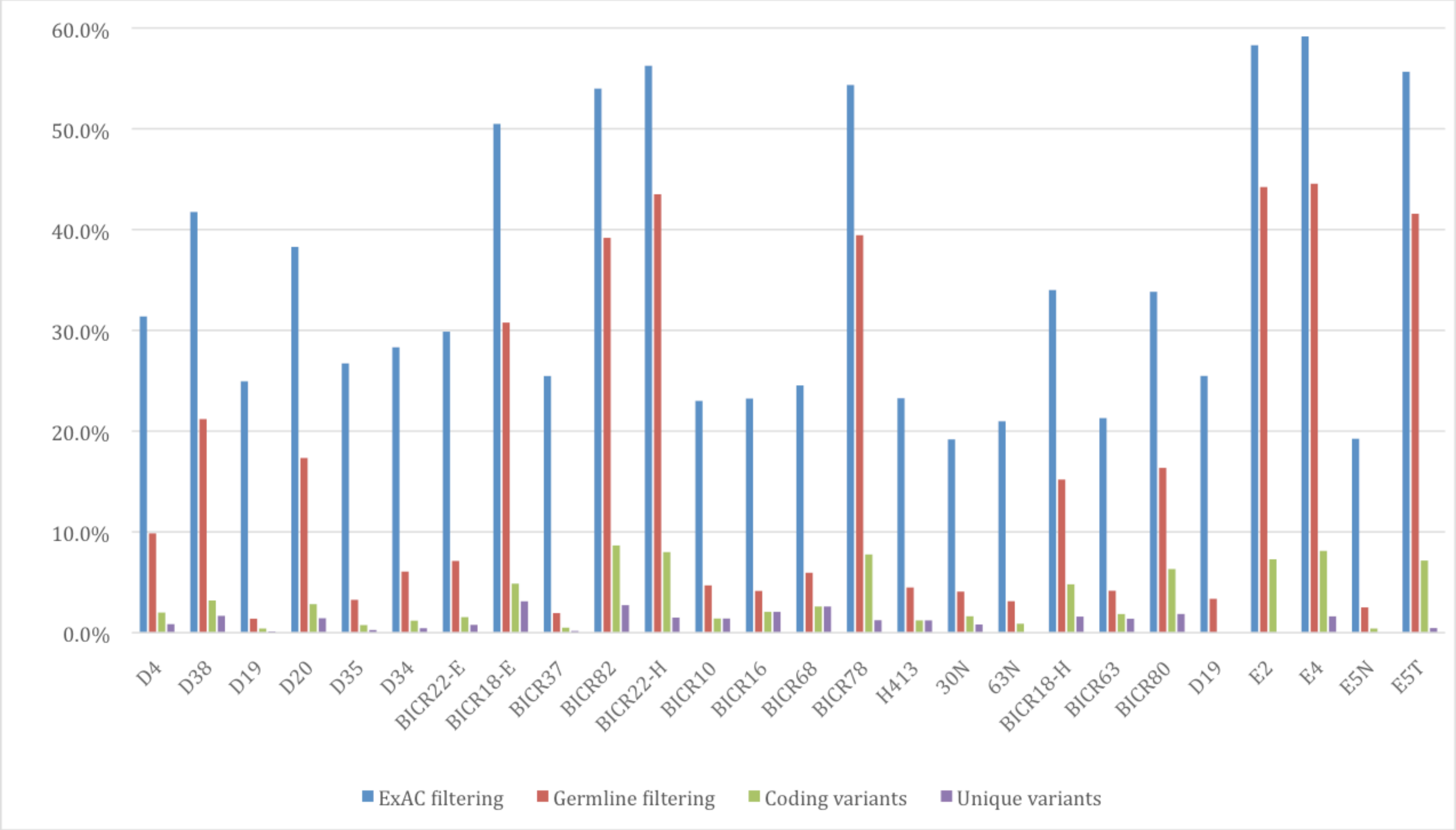

SUPPLEMENTARY DATA S6 - COMPARISION OF SCNAS IN TWO HNSCC PANELS

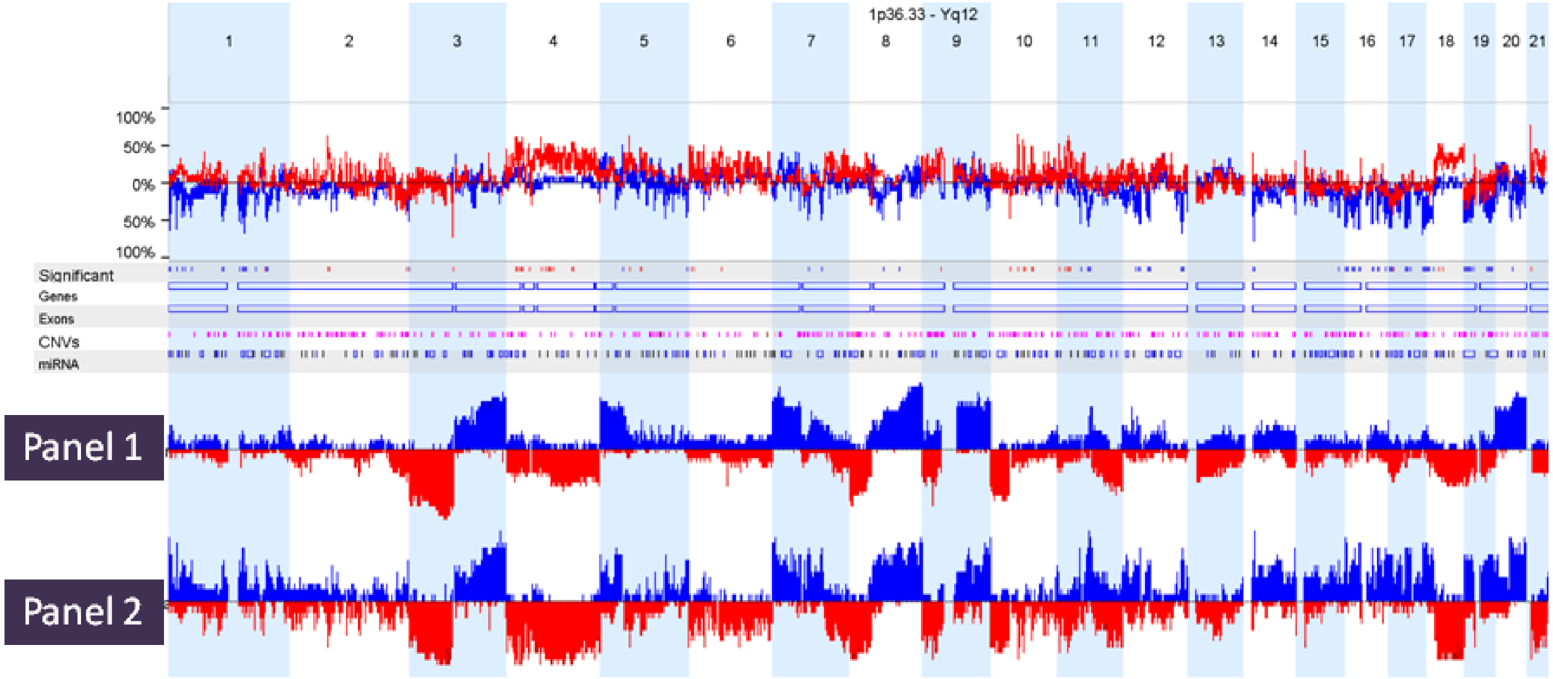

Karyograms of two HNSCC panels used in the study. Panel 1 was subject to SNP array analyses and Panel 2 was subject to Array CGH. Although there were some small differences overall the data were comparable and were merged for further analyses

SUPPLEMENTARY DATA S7 – SUBTRACTION COPY NUMBER FREQUENCY PLOT OF MORTAL PPOLS AND MATCHING FIBROBLASTS

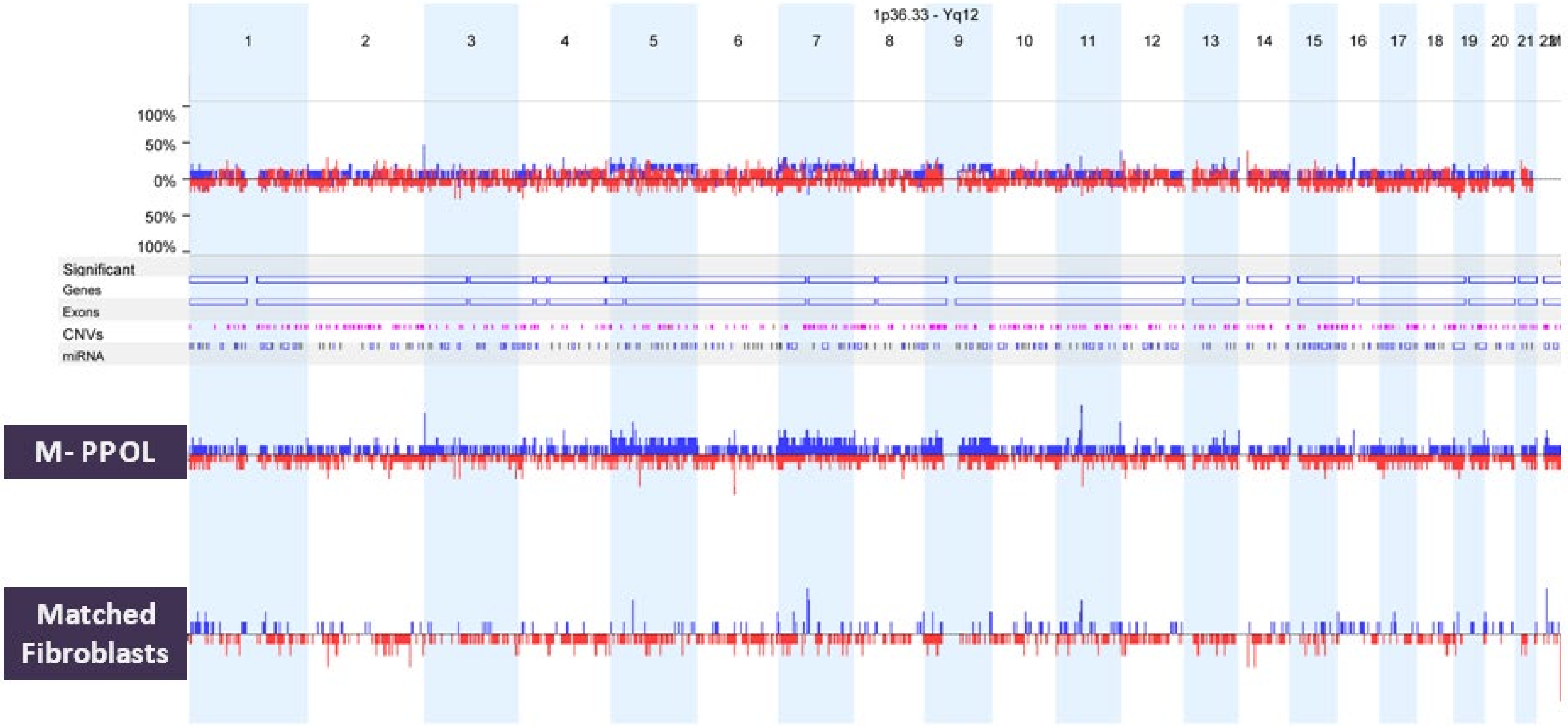

Subtraction karyogram showing lack of significant difference in copy number gains (blue) and losses (red) between mortal PPOLs (M-PPOLs) and matched fibroblasts.

SUPPLEMENTARY DATA S8 – IMMORTAL PPOL CELL LINES SCNA DENSITY PLOTS FOR CHROMOSOMES 3, 8, 9 AND 20

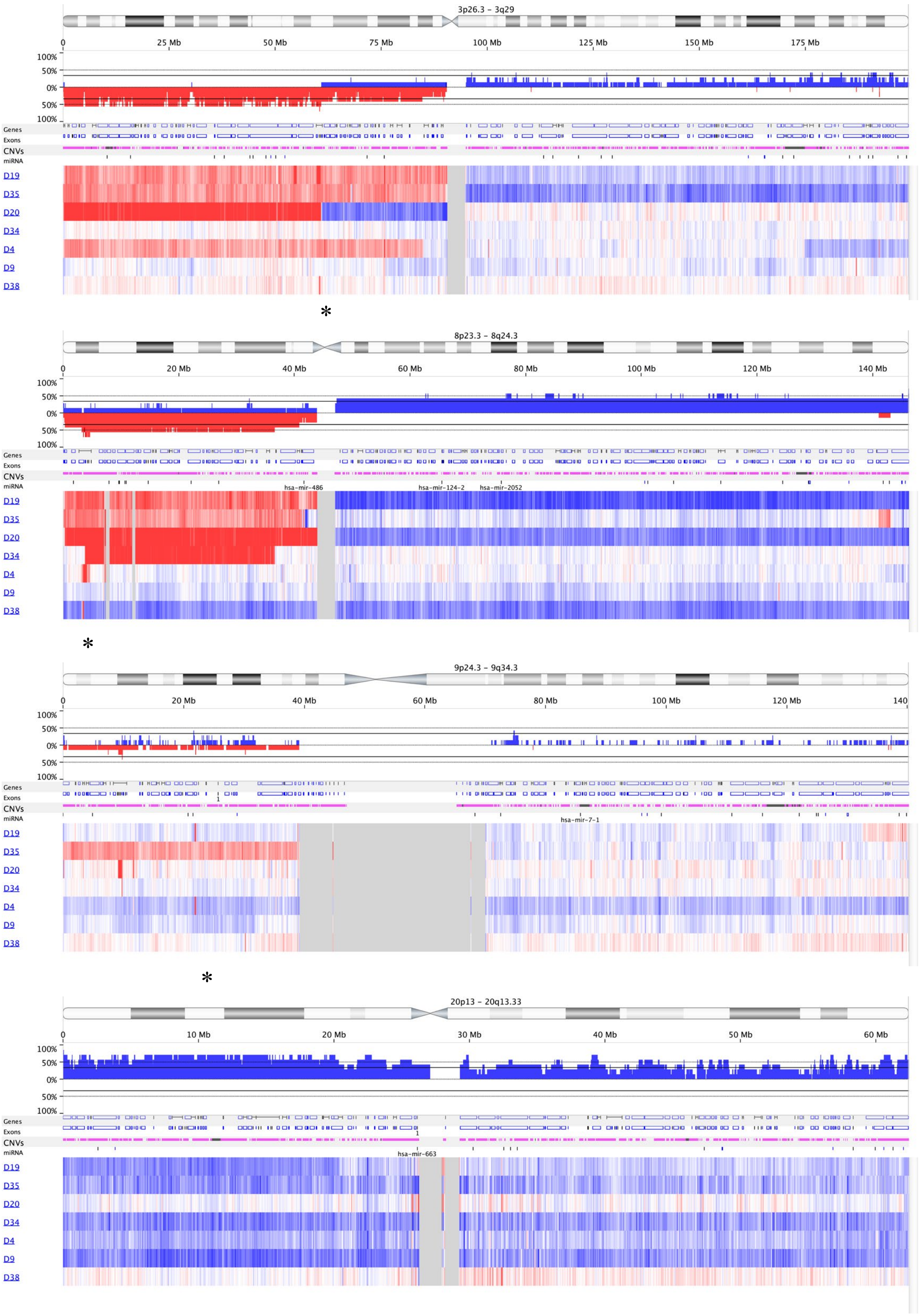

Immortal PPOL SCNA density plots for chromosomes 3, 8, 9 and 20 (from top to bottom). D19, D20 and D35 are derived from progressive PPOL lesions. Asterisk (*) <u>below</u> density plots for chromosomes 3, 8 and 9 indicate positions of *FHIT*, *CSMD1* and *CDKN2A* respectively.

SUPPLEMENTARY DATA S9 - HIERARCHICAL CLUSTER ANALYSIS OF IMMORTAL HNSCC CELL LINES FOR HIGH COPY NUMBER SCNA - ASSOCIATION WITH LYMPH NODE METASTASES

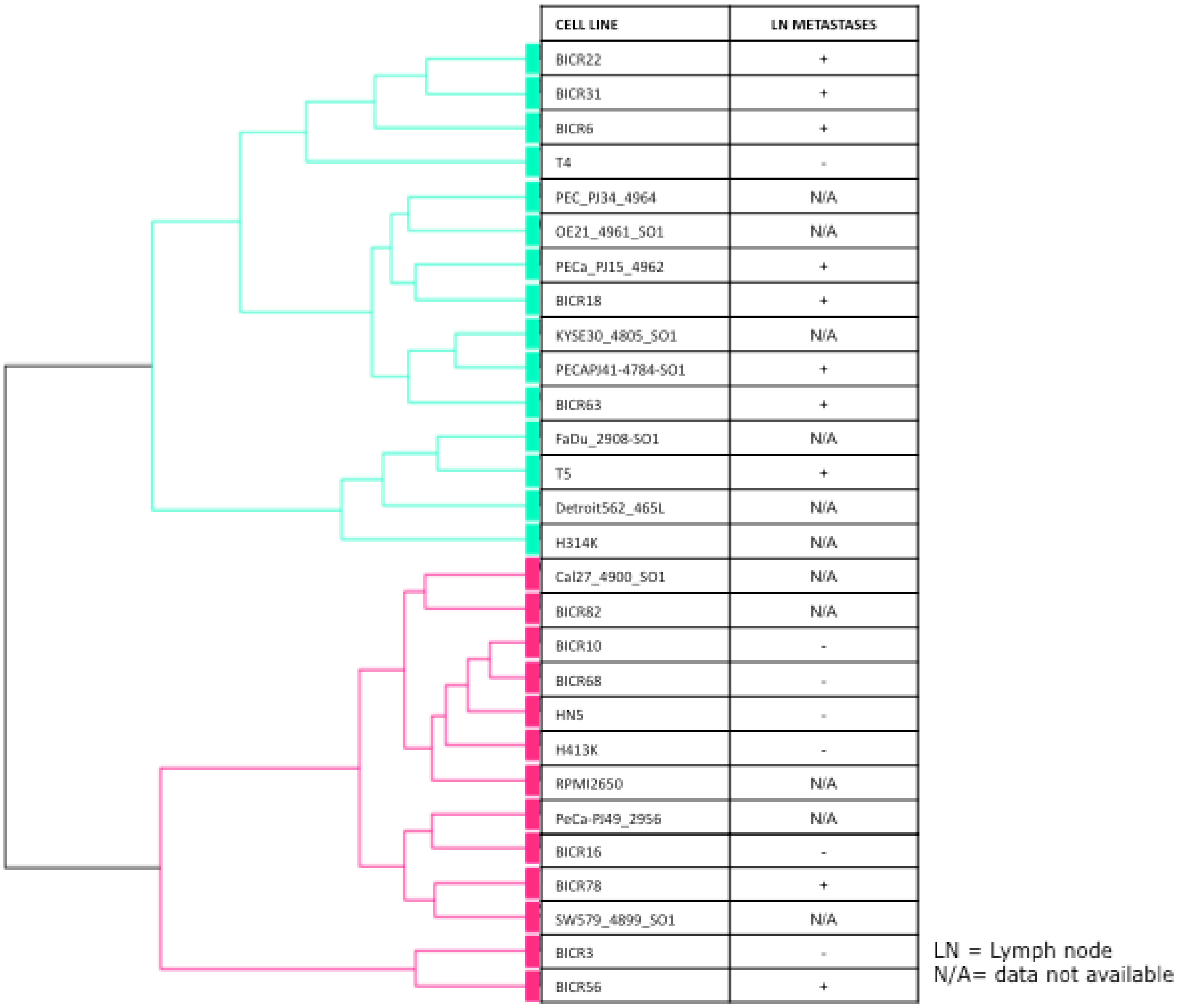

CELL LINE	LN METASTASES
BICR22	+
BICR31	+
BICR6	+
T4	-
PEC_PJ34_4964	N/A
OE21_4961_SO1	N/A
PECa_PJ15_4962	+
BICR18	+
KYSE30_4805_SO1	N/A
PECAPJ41-4784-SO1	+
BICR63	+
FaDu_2908-SO1	N/A
T5	+
Detroit562_465L	N/A
H314K	N/A
Cal27_4900_SO1	N/A
BICR82	N/A
BICR10	-
BICR68	-
HN5	-
H413K	-
RPMI2650	N/A
PeCa-PJ49_2956	N/A
BICR16	-
BICR78	+
SW579_4899_SO1	N/A
BICR3	-
BICR56	+

LN = Lymph node
N/A= data not available

Hierarchical Cluster Analysis (Nexus Copy Number v5.1, BioDiscovery Inc.) of Immortal HNSCC Cell Lines For High Copy Number SCNA

SUPPLEMENTARY DATA S10 - GISTIC PEAK REGIONS IN PPOLS AND HNSCC CELL LINES

Table 1. GISTIC peak regions in PPOLs with varying significance thresholds (Q bound value)

Gene Symbol	Chromosome	Start	End	Length	Q<0.05
NCKAP5	chr2	133145841	134042501	896661	
SORBS2	chr4	186743591	187114864	371274	
TLR3	chr4	187227302	187243246	15945	
FAM149A	chr4	187302988	187330811	27824	
CYP4V2	chr4	187349667	187371611	21945	
KLKB1	chr4	187385665	187416619	30955	
F11	chr4	187424111	187447829	23719	
MTNR1A	chr4	187691802	187713531	21730	
FAT1	chr4	187745930	187881981	136052	
PLEKHG4B	chr5	193372	243087	49716	
LRRC14B	chr5	244625	248468	3844	
CCDC127	chr5	257874	271297	13424	
SDHA	chr5	271355	309814	38460	
CSMD1	chr8	2780282	4839736	2059455	
ZNF16	chr8	146126547	146147078	20532	
ZNF252	chr8	146169778	146199089	29312	
TMED10P	chr8	146191054	146195086	4033	
C8orf77	chr8	146199000	146202235	3236	
C8orf33	chr8	146248627	146252220	3594	
C9orf53	chr9	21957137	21957752	616	
CDKN2A	chr9	21957750	21984490	26741	
CDKN2B	chr9	21992901	21999312	6412	
CDKN2BAS	chr9	21984789	22111091	126303	
DEFB125	chr20	16350	25296	8947	
DEFB126	chr20	71251	74392	3142	

Gene Symbol	Chromosome	Start	End	Length	Q<0.1
FAM110C	chr2	28815	36588	7774	
NCKAP5	chr2	133145841	134042501	896661	
SORBS2	chr4	186743591	187114864	371274	
TLR3	chr4	187227302	187243246	15945	
FAM149A	chr4	187302988	187330811	27824	
CYP4V2	chr4	187349667	187371611	21945	
KLKB1	chr4	187385665	187416619	30955	
F11	chr4	187424111	187447829	23719	
MTNR1A	chr4	187691802	187713531	21730	
FAT1	chr4	187745930	187881981	136052	
PLEKHG4B	chr5	193372	243087	49716	
LRRC14B	chr5	244625	248468	3844	
CCDC127	chr5	257874	271297	13424	
SDHA	chr5	271355	309814	38460	
CSMD1	chr8	2780282	4839736	2059455	
ZNF16	chr8	146126547	146147078	20532	
ZNF252	chr8	146169778	146199089	29312	
TMED10P	chr8	146191054	146195086	4033	
C8orf77	chr8	146199000	146202235	3236	
C8orf33	chr8	146248627	146252220	3594	
C9orf53	chr9	21957137	21957752	616	
CDKN2A	chr9	21957750	21984490	26741	
CDKN2B	chr9	21992901	21999312	6412	
CDKN2BAS	chr9	21984789	22111091	126303	
TUBB8	chr10	82827	85504	2678	
ZMYND11	chr10	170423	290576	120154	

DEFB125	chr20	16350	25296	8947	
DEFB126	chr20	71251	74392	3142	
NCRNA00176	chr20	62136140	62141758	5619	
SOX18	chr20	62149522	62151423	1902	
TCEA2	chr20	62158882	62174144	15263	
RGS19	chr20	62174978	62181768	6791	
C20orf201	chr20	62185176	62186156	981	
OPRL1	chr20	62181931	62202440	20510	
NPBWR2	chr20	62207626	62208628	1003	
MYT1	chr20	62266270	62344050	77781	
PCMTD2	chr20	62357491	62378023	20533	
S100B	chr21	46842958	46849463	6506	
PRMT2	chr21	46879954	46909291	29338	

Gene Symbol	Chromosome	Start	End	Length	Q<0.25
NME7	chr1	167368392	167603810	235419	
TDRD5	chr1	177827647	177927021	99375	
ZNF648	chr1	180290327	180297470	7144	
FAM110C	chr2	28815	36588	7774	
NCKAP5	chr2	133145841	134042501	896661	
FHIT	chr3	59710075	61212173	1502099	
CCDC50	chr3	192529567	192599152	69586	
FAM190A	chr4	91267706	92742392	1474687	
SORBS2	chr4	186743591	187114864	371274	
TLR3	chr4	187227302	187243246	15945	
FAM149A	chr4	187302988	187330811	27824	
CYP4V2	chr4	187349667	187371611	21945	
KLKB1	chr4	187385665	187416619	30955	
F11	chr4	187424111	187447829	23719	
MTNR1A	chr4	187691802	187713531	21730	
FAT1	chr4	187745930	187881981	136052	
PLEKHG4B	chr5	193372	243087	49716	
LRRC14B	chr5	244625	248468	3844	
CCDC127	chr5	257874	271297	13424	
SDHA	chr5	271355	309814	38460	
PDE4D	chr5	58300622	59819682	1519061	
TBP	chr6	170705345	170723882	18538	
PDCD2	chr6	170728374	170735673	7300	
CSMD1	chr8	2780282	4839736	2059455	
EYA1	chr8	72272221	72437021	164801	
ZNF16	chr8	146126547	146147078	20532	
ZNF252	chr8	146169778	146199089	29312	
TMED10P	chr8	146191054	146195086	4033	
C8orf77	chr8	146199000	146202235	3236	
C8orf33	chr8	146248627	146252220	3594	
PTPRD	chr9	8304246	10602723	2298478	
C9orf53	chr9	21957137	21957752	616	
CDKN2A	chr9	21957750	21984490	26741	
CDKN2B	chr9	21992901	21999312	6412	
CDKN2BAS	chr9	21984789	22111091	126303	
TUBB8	chr10	82827	85504	2678	
ZMYND11	chr10	170423	290576	120154	
OR4C11	chr11	55127492	55128425	934	
OR4P4	chr11	55162409	55163348	940	
OR4S2	chr11	55174955	55175891	937	
OR4C6	chr11	55189218	55190148	931	
FAM138D	chr12	18207	19673	1467	
IQSEC3	chr12	46309	157884	111576	

LOC100133469	chr14	106009499	106022574	13076
NCLN	chr19	3136874	3160573	23700
CELF5	chr19	3175700	3248073	72374
DEFB125	chr20	16350	25296	8947
DEFB126	chr20	71251	74392	3142
NCRNA00176	chr20	62136140	62141758	5619
SOX18	chr20	62149522	62151423	1902
TCEA2	chr20	62158882	62174144	15263
RGS19	chr20	62174978	62181768	6791
C20orf201	chr20	62185176	62186156	981
OPRL1	chr20	62181931	62202440	20510
NPBWR2	chr20	62207626	62208628	1003
MYT1	chr20	62266270	62344050	77781
PCMTD2	chr20	62357491	62378023	20533
S100B	chr21	46842958	46849463	6506
PRMT2	chr21	46879954	46909291	29338

Table 2. GISTIC peak regions in HNSCCs with varying significance thresholds (Q bound value)

Gene Symbol	Chromosome	Start	End	Length	Q<0.05
LCE3C	chr1	150839761	150840186	426	
LRP1B	chr2	140705465	142605740	1900276	
LOC728323	chr2	242679516	242751142	71627	
CNTN4	chr3	2117246	3074645	957400	
FHIT	chr3	59710075	61212173	1502099	
CCDC50	chr3	192529567	192599152	69586	
FAM190A	chr4	91267706	92742392	1474687	
PDZD2	chr5	31834787	32146795	312009	
GOLPH3	chr5	32160580	32210182	49603	
PDE4D	chr5	58300622	59819682	1519061	
HLA-DRB5	chr6	32593131	32605984	12854	
EGFR	chr7	55054218	55242525	188308	
GATS	chr7	99636215	99707791	71577	
SPDYE3	chr7	99743260	99757754	14495	
PMS2L1	chr7	99756198	99771866	15669	
PILRB	chr7	99771672	99803388	31717	
PILRA	chr7	99809003	99835658	26656	
ZCWPW1	chr7	99836430	99864238	27809	
MEPCE	chr7	99865464	99869677	4214	
C7orf47	chr7	99870847	99872030	1184	
C7orf61	chr7	99892173	99899830	7658	
TSC22D4	chr7	99902077	99914838	12762	
C7orf51	chr7	99919485	99930358	10874	
AGFG2	chr7	99974769	100003779	29011	
SAP25	chr7	100007788	100009206	1419	
LRCH4	chr7	100009569	100021712	12144	
FBXO24	chr7	100021891	100036676	14786	
PCOLCE	chr7	100037817	100043734	5918	
MOSPD3	chr7	100047660	100050936	3277	
TFR2	chr7	100055974	100077109	21136	
ACTL6B	chr7	100078661	100092020	13360	
GNB2	chr7	100109298	100114727	5430	
GIGYF1	chr7	100115065	100124806	9742	
POP7	chr7	100141611	100143059	1449	
EPO	chr7	100156358	100159259	2902	
ZAN	chr7	100169184	100233355	64172	
MLL3	chr7	151462942	151764023	301082	
CSMD1	chr8	2780282	4839736	2059455	
ADAM5P	chr8	39291338	39379532	88195	
ADAM3A	chr8	39427721	39499627	71907	
C8orf77	chr8	146199000	146202235	3236	
C8orf33	chr8	146248627	146252220	3594	
C9orf53	chr9	21957137	21957752	616	
CDKN2A	chr9	21957750	21984490	26741	
NOTCH1	chr9	138508716	138560059	51344	
PARD3	chr10	34440102	35143929	703828	
CD44	chr11	35116992	35210525	93534	
SLC1A2	chr11	35229327	35397681	168355	
PAMR1	chr11	35409951	35503752	93802	
FJX1	chr11	35596310	35598995	2686	
TRIM44	chr11	35640928	35787506	146579	
OR4P4	chr11	55162409	55163348	940	
OR4S2	chr11	55174955	55175891	937	
OR4C6	chr11	55189218	55190148	931	
ANO1	chr11	69602055	69713299	111245	

FADD	chr11	69726916	69731156	4241	
MIR548K	chr11	69807708	69807824	117	
PPFIA1	chr11	69794470	69908150	113681	
CTTN	chr11	69922259	69960338	38080	
SHANK2	chr11	69991609	70613456	621848	
PPP2R5E	chr14	62911107	63079832	168726	
FSCN2	chr17	77110011	77114631	4621	
C17orf70	chr17	77117387	77129871	12485	
NPLOC4	chr17	77134356	77214543	80188	
TSPAN10	chr17	77219753	77226184	6432	
PDE6G	chr17	77227893	77234012	6120	
C17orf90	chr17	77242470	77244023	1554	
CCDC137	chr17	77244165	77251341	7177	
ARL16	chr17	77258628	77261359	2732	
HGS	chr17	77261424	77279552	18129	
MRPL12	chr17	77280811	77284953	4143	
SLC25A10	chr17	77289775	77298447	8673	
DYSFIP1	chr17	77384656	77386215	1560	
P4HB	chr17	77394322	77411833	17512	
TCF4	chr18	51040559	51406858	366300	
DEFB127	chr20	86185	87804	1620	
CHMP4B	chr20	31862770	31905834	43065	

Gene Symbol	Chromosome	Start	End	Length	Q<0.25
LCE3C	chr1	150839761	150840186	426	
OR2T34	chr1	246803724	246804681	958	
OR2T10	chr1	246822753	246823692	940	
OR2T11	chr1	246856101	246857052	952	
OR2T35	chr1	246868210	246869182	973	
LRP1B	chr2	140705465	142605740	1900276	
PARD3B	chr2	205118760	206188782	1070023	
LOC728323	chr2	242679516	242751142	71627	
CNTN4	chr3	2117246	3074645	957400	
SCN5A	chr3	38564556	38666168	101613	
SCN10A	chr3	38713840	38810505	96666	
SCN11A	chr3	38862263	38967056	104794	
FHIT	chr3	59710075	61212173	1502099	
CCDC50	chr3	192529567	192599152	69586	
FAM190A	chr4	91267706	92742392	1474687	
SORBS2	chr4	186743591	187114864	371274	
PDZD2	chr5	31834787	32146795	312009	
GOLPH3	chr5	32160580	32210182	49603	
PDE4D	chr5	58300622	59819682	1519061	
NSD1	chr5	176492685	176659820	167136	
HLA-H	chr6	29963361	29966835	3475	
HCG2P7	chr6	29974786	29978408	3623	
HCG4P6	chr6	30000347	30001407	1061	
HLA-DRB5	chr6	32593131	32605984	12854	
EGFR	chr7	55054218	55242525	188308	
GATS	chr7	99636215	99707791	71577	
SPDYE3	chr7	99743260	99757754	14495	
PMS2L1	chr7	99756198	99771866	15669	
PILRB	chr7	99771672	99803388	31717	
PILRA	chr7	99809003	99835658	26656	
ZCWPW1	chr7	99836430	99864238	27809	
MEPCE	chr7	99865464	99869677	4214	
C7orf47	chr7	99870847	99872030	1184	
C7orf61	chr7	99892173	99899830	7658	

TSC22D4	chr7	99902077	99914838	12762
C7orf51	chr7	99919485	99930358	10874
AGFG2	chr7	99974769	100003779	29011
SAP25	chr7	100007788	100009206	1419
LRCH4	chr7	100009569	100021712	12144
FBXO24	chr7	100021891	100036676	14786
PCOLCE	chr7	100037817	100043734	5918
MOSPD3	chr7	100047660	100050936	3277
TFR2	chr7	100055974	100077109	21136
ACTL6B	chr7	100078661	100092020	13360
GNB2	chr7	100109298	100114727	5430
GIGYF1	chr7	100115065	100124806	9742
POP7	chr7	100141611	100143059	1449
EPO	chr7	100156358	100159259	2902
ZAN	chr7	100169184	100233355	64172
MLL3	chr7	151462942	151764023	301082
CSMD1	chr8	2780282	4839736	2059455
ADAM5P	chr8	39291338	39379532	88195
ADAM3A	chr8	39427721	39499627	71907
C8orf77	chr8	146199000	146202235	3236
C8orf33	chr8	146248627	146252220	3594
GLIS3	chr9	3814127	4290035	475909
C9orf53	chr9	21957137	21957752	616
CDKN2A	chr9	21957750	21984490	26741
NOTCH1	chr9	138508716	138560059	51344
PARD3	chr10	34440102	35143929	703828
OR52N5	chr11	5755441	5756473	1033
CD44	chr11	35116992	35210525	93534
SLC1A2	chr11	35229327	35397681	168355
PAMR1	chr11	35409951	35503752	93802
FJX1	chr11	35596310	35598995	2686
TRIM44	chr11	35640928	35787506	146579
OR4P4	chr11	55162409	55163348	940
OR4S2	chr11	55174955	55175891	937
OR4C6	chr11	55189218	55190148	931
ANO1	chr11	69602055	69713299	111245
FADD	chr11	69726916	69731156	4241
MIR548K	chr11	69807708	69807824	117
PPFIA1	chr11	69794470	69908150	113681
CTTN	chr11	69922259	69960338	38080
SHANK2	chr11	69991609	70613456	621848
TRPC6	chr11	100827504	100959869	132366
ANGPTL5	chr11	101266614	101292463	25850
KIAA1377	chr11	101290955	101377003	86049
C11orf70	chr11	101423408	101459356	35949
YAP1	chr11	101486419	101609364	122946
BIRC3	chr11	101693403	101713675	20273
BIRC2	chr11	101723175	101754611	31437
TMEM123	chr11	101772265	101828985	56721
MMP7	chr11	101896448	101906688	10241
MMP20	chr11	101952775	102001273	48499
MMP27	chr11	102067624	102081678	14055
MMP8	chr11	102087735	102100895	13161
DDX12	chr12	9461554	9492035	30482
PPP2R5E	chr14	62911107	63079832	168726
LOC727924	chr15	19779395	19872450	93056
SOLH	chr16	517856	544637	26782
C16orf11	chr16	550422	555530	5109

NHLRC4	chr16	557032	559496	2465
PIGQ	chr16	560004	574110	14107
RAB40C	chr16	579357	619274	39918
WFIKKN1	chr16	621012	624117	3106
C16orf13	chr16	624429	626348	1920
FAM195A	chr16	631849	638475	6627
WDR90	chr16	639363	657830	18468
RHOT2	chr16	658133	664172	6040
RHBDL1	chr16	666075	668268	2194
STUB1	chr16	670115	672769	2655
JMJD8	chr16	671667	674440	2774
WDR24	chr16	674702	680401	5700
EDC4	chr16	66464499	66475907	11409
NRN1L	chr16	66476281	66477772	1492
PSKH1	chr16	66484675	66521082	36408
CTRL	chr16	66520973	66523266	2294
PSMB10	chr16	66525907	66528254	2348
LCAT	chr16	66531287	66535516	4230
SLC12A4	chr16	66534877	66560098	25222
DPEP3	chr16	66567066	66571953	4888
DPEP2	chr16	66578795	66590865	12071
DDX28	chr16	66612679	66615271	2593
DUS2L	chr16	66614704	66670685	55982
WWOX	chr16	76691051	77804065	1113015
FAM57A	chr17	582596	592825	10230
GEMIN4	chr17	594410	602251	7842
ELP2P	chr17	602649	605326	2678
GLOD4	chr17	609299	632321	23023
RNMTL1	chr17	632262	642491	10230
TRPV1	chr17	3415489	3459454	43966
SHPK	chr17	3458304	3486365	28062
CTNS	chr17	3486510	3513146	26637
FSCN2	chr17	77110011	77114631	4621
C17orf70	chr17	77117387	77129871	12485
NPLOC4	chr17	77134356	77214543	80188
TSPAN10	chr17	77219753	77226184	6432
PDE6G	chr17	77227893	77234012	6120
C17orf90	chr17	77242470	77244023	1554
CCDC137	chr17	77244165	77251341	7177
ARL16	chr17	77258628	77261359	2732
HGS	chr17	77261424	77279552	18129
MRPL12	chr17	77280811	77284953	4143
SLC25A10	chr17	77289775	77298447	8673
DYSFIP1	chr17	77384656	77386215	1560
P4HB	chr17	77394322	77411833	17512
ADCYAP1	chr18	894943	902173	7231
FAM38B	chr18	10660243	11138761	478519
TCF4	chr18	51040559	51406858	366300
DEFB127	chr20	86185	87804	1620
TMEM90B	chr20	24397834	24595167	197334
CHMP4B	chr20	31862770	31905834	43065
NCRNA00160	chr21	35017974	35031349	13376
RUNX1	chr21	35081967	35343465	261499
C21orf96	chr21	35332104	35333593	1490
Sep-05	chr22	18081986	18092297	10312
GP1BB	chr22	18091065	18092297	1233
TBX1	chr22	18124225	18151112	26888
GNB1L	chr22	18155933	18222462	66530

APOBEC3A	chr22	37683472	37689134	5663
SPRY3	chrY	57509844	57524511	14668
VAMP7	chrY	57623340	57685826	62487
IL9R	chrY	57739639	57752876	13238

Table 3. GISTIC peak regions in LN-ve HNSCCs with Q bound value<0.25

Gene Symbol	Chromosome	Start	End	Length	Q<0.25
LCE3D	chr1	150818483	150819604	1122	
LCE3C	chr1	150839761	150840186	426	
LCE3B	chr1	150852910	150853198	289	
VSTM2A	chr7	54577512	54604442	26931	
SEC61G	chr7	54787433	54794433	7001	
EGFR	chr7	55054218	55242525	188308	
PDK4	chr7	95050744	95063861	13118	
DYNC1I1	chr7	95239753	95565671	325919	
MIR661	chr8	145091346	145091435	90	
PLEC	chr8	145061308	145122901	61594	
PARP10	chr8	145123307	145132623	9317	
GRINA	chr8	145136213	145139571	3359	
SPATC1	chr8	145158569	145174002	15434	
OPLAH	chr8	145178154	145187572	9419	
EXOSC4	chr8	145205509	145207539	2031	
GPAA1	chr8	145209511	145213107	3597	
CYC1	chr8	145221947	145224416	2470	
SHARPIN	chr8	145225523	145231128	5606	
MAF1	chr8	145231292	145234503	3212	
KIAA1875	chr8	145234616	145245206	10591	
C8orf30A	chr8	145264659	145267608	2950	
HEATR7A	chr8	145274906	145388831	113926	
SCXA	chr8	145393504	145395033	1530	
SCXB	chr8	145393504	145395033	1530	
C8orf30A	chr8	145408687	145411636	2950	
SCXA	chr8	145461410	145462939	1530	
SCXB	chr8	145461410	145462939	1530	
BOP1	chr8	145456863	145485928	29066	
HSF1	chr8	145486077	145509193	23117	
DGAT1	chr8	145509054	145521375	12322	
SCRT1	chr8	145525261	145530751	5491	
C8ORFK29	chr8	145547694	145549313	1620	
FBXL6	chr8	145549898	145552940	3043	
GPR172A	chr8	145553032	145555754	2723	
ADCK5	chr8	145568538	145589261	20724	
MIR939	chr8	145590171	145590253	83	
MIR1234	chr8	145596283	145596367	85	
CPSF1	chr8	145589253	145605541	16289	
SLC39A4	chr8	145608605	145613081	4477	
VPS28	chr8	145619807	145624735	4929	
NFKBIL2	chr8	145624970	145640620	15651	
CYHR1	chr8	145646122	145661839	15718	
EGFL7	chr9	138677197	138686951	9755	
MIR126	chr9	138684874	138684958	85	
AGPAT2	chr9	138687415	138701732	14318	
FAM69B	chr9	138726844	138738990	12147	
SIRPA	chr20	1822812	1868540	45729	
GATA5	chr20	60471947	60484421	12475	
C20orf200	chr20	60551882	60559213	7332	
VAMP7	chrY	57623340	57685826	62487	
IL9R	chrY	57739639	57752876	13238	

Table 4. GISTIC peak regions in LN+ve HNSCCs with Q bound value<0.25

Gene Symbol	Chromosome	Start	End	Length	Q,0.25
LCE3C	chr1	150839761	150840186	426	
LRP1B	chr2	140705465	142605740	1900276	
PARD3B	chr2	205118760	206188782	1070023	
LOC728323	chr2	242679516	242751142	71627	
CNTN4	chr3	2117246	3074645	957400	
SCN5A	chr3	38564556	38666168	101613	
SCN10A	chr3	38713840	38810505	96666	
SCN11A	chr3	38862263	38967056	104794	
SFMBT1	chr3	52913666	53055110	141445	
CCDC50	chr3	192529567	192599152	69586	
SEC24B	chr4	110574419	110681064	106646	
PDZD2	chr5	31834787	32146795	312009	
NSD1	chr5	176492685	176659820	167136	
HLA-DRB5	chr6	32593131	32605984	12854	
HLA-DRB6	chr6	32628467	32635757	7291	
HLA-DRB1	chr6	32654524	32665540	11017	
MICALL2	chr7	1440520	1465635	25116	
INTS1	chr7	1476438	1510544	34107	
MAFK	chr7	1536893	1549205	12313	
TMEM184A	chr7	1548396	1562592	14197	
PSMG3	chr7	1573494	1576194	2701	
KIAA1908	chr7	1576234	1595785	19552	
GATS	chr7	99636215	99707791	71577	
SPDYE3	chr7	99743260	99757754	14495	
PMS2L1	chr7	99756198	99771866	15669	
PILRB	chr7	99771672	99803388	31717	
PILRA	chr7	99809003	99835658	26656	
ZCWPW1	chr7	99836430	99864238	27809	
MEPCE	chr7	99865464	99869677	4214	
C7orf47	chr7	99870847	99872030	1184	
C7orf61	chr7	99892173	99899830	7658	
TSC22D4	chr7	99902077	99914838	12762	
C7orf51	chr7	99919485	99930358	10874	
AGFG2	chr7	99974769	100003779	29011	
SAP25	chr7	100007788	100009206	1419	
LRCH4	chr7	100009569	100021712	12144	
FBXO24	chr7	100021891	100036676	14786	
PCOLCE	chr7	100037817	100043734	5918	
MOSPD3	chr7	100047660	100050936	3277	
TFR2	chr7	100055974	100077109	21136	
ACTL6B	chr7	100078661	100092020	13360	
GNB2	chr7	100109298	100114727	5430	
GIGYF1	chr7	100115065	100124806	9742	
POP7	chr7	100141611	100143059	1449	
EPO	chr7	100156358	100159259	2902	
ZAN	chr7	100169184	100233355	64172	
EPHB4	chr7	100238122	100263079	24958	
MLL3	chr7	151462942	151764023	301082	
CSMD1	chr8	2780282	4839736	2059455	
ADAM5P	chr8	39291338	39379532	88195	
ADAM3A	chr8	39427721	39499627	71907	
MIR661	chr8	145091346	145091435	90	
PLEC	chr8	145061308	145122901	61594	
PARP10	chr8	145123307	145132623	9317	

GRINA	chr8	145136213	145139571	3359
SPATC1	chr8	145158569	145174002	15434
OPLAH	chr8	145178154	145187572	9419
EXOSC4	chr8	145205509	145207539	2031
GPAA1	chr8	145209511	145213107	3597
CYC1	chr8	145221947	145224416	2470
SHARPIN	chr8	145225523	145231128	5606
MAF1	chr8	145231292	145234503	3212
KIAA1875	chr8	145234616	145245206	10591
C8orf30A	chr8	145264659	145267608	2950
HEATR7A	chr8	145274906	145388831	113926
SCXA	chr8	145393504	145395033	1530
SCXB	chr8	145393504	145395033	1530
C8orf30A	chr8	145408687	145411636	2950
GLIS3	chr9	3814127	4290035	475909
C9orf53	chr9	21957137	21957752	616
CDKN2A	chr9	21957750	21984490	26741
NOTCH1	chr9	138508716	138560059	51344
MYEOV	chr11	68818197	68821330	3134
CCND1	chr11	69165053	69178423	13371
ORAOV1	chr11	69189512	69199346	9835
FGF19	chr11	69222186	69228287	6102
FGF4	chr11	69296977	69299352	2376
FGF3	chr11	69333916	69343129	9214
ANO1	chr11	69602055	69713299	111245
FADD	chr11	69726916	69731156	4241
MIR548K	chr11	69807708	69807824	117
PPFIA1	chr11	69794470	69908150	113681
CTTN	chr11	69922259	69960338	38080
SHANK2	chr11	69991609	70613456	621848
TRPC6	chr11	100827504	100959869	132366
ANGPTL5	chr11	101266614	101292463	25850
KIAA1377	chr11	101290955	101377003	86049
C11orf70	chr11	101423408	101459356	35949
YAP1	chr11	101486419	101609364	122946
BIRC3	chr11	101693403	101713675	20273
BIRC2	chr11	101723175	101754611	31437
TMEM123	chr11	101772265	101828985	56721
MMP7	chr11	101896448	101906688	10241
MMP20	chr11	101952775	102001273	48499
MMP27	chr11	102067624	102081678	14055
MMP8	chr11	102087735	102100895	13161
CACNA1C	chr12	2032676	2677376	644701
RPSAP52	chr12	64438069	64507021	68953
HMGA2	chr12	64504506	64646338	141833
GPHN	chr14	66043877	66718278	674402
FAM71D	chr14	66725898	66765020	39123
MPP5	chr14	66777773	66872289	94517
SEC14L1	chr17	72648599	72724774	76176
FAM38B	chr18	10660243	11138761	478519
RPRD1A	chr18	31823789	31901371	77583
SLC39A6	chr18	31942491	31963355	20865
ELP2	chr18	31963884	32008605	44722
MOCOS	chr18	32021477	32102683	81207
FHOD3	chr18	32131699	32614016	482318
RIT2	chr18	38577189	38949655	372467
SYT4	chr18	39101854	39111613	9760
DEFB125	chr20	16350	25296	8947

DEFB126	chr20	71251	74392	3142
DEFB127	chr20	86185	87804	1620
NTSR1	chr20	60810633	60864568	53936
GSTTP1	chr22	22670594	22677258	6665
LOC391322	chr22	22703116	22704043	928
GSTT1	chr22	22706138	22714284	8147
GSTTP2	chr22	22715937	22731899	15963

SUPPLEMENTARY DATA S11 - COPY NUMBER GAINS AND LOSSES OF THE TOP RANKED GENES IN TOP RANKED PEAKS REGIONS OF FOCAL DELETIONS IDENTIFIED BY BEROUKHIM ET AL., 2010

Table 1. Single and homozygous copy number loss of the top ranked genes in top ranked peaks regions of focal deletions identified by Beroukhim et al., 2010 in a panel of 3131 cell lines. Only genes showing >30% frequency of hemizygous deletions in any one group and/or any homozygous deletions are shown. [1]Peak rank from Beroukhim et al., 2010; [2]top rank gene within the peak; [3]includes cell lines for which lymph node metastases status was not available. Genes indicated in bold are those for which expression array data was available from Hunter et al. 2006. *Show correlation of SCNA/LOH with gene expression with nominal significance (p<0.05). **Show correlation of CAN/LOH gene expression after correction for multiple testing (FDR<0.05).

Table 2. Amplification of the top ranked genes in top ranked peaks regions of focal amplifications identified by Beroukhim et al., 2010 in a panel of 3131 cell lines. Only genes showing >30% frequency of low copy number (<2) amplification in any one group and/or any high copy number gain are shown. [1]Peak rank from Beroukhim et al., 2010; [2]top rank gene within the peak; [3]includes cell lines for which lymph node metastases status was not available. Genes indicated in bold are those for which expression array data was available from Hunter et al. 2006. *Show correlation of SCNA/LOH with gene expression with nominal significance (p<0.05). **Show correlation of CAN/LOH gene expression after correction for multiple testing (FDR<0.05).

				Single Copy Loss Frequency (%)				Homozygous Loss Frequency (%)			
				PMOL	OSCC			PMOL	OSCC		
**Rank[1]**	**Chromosome Band**	**Known Target**	**GRAIL Top Target[2]**	**N=7**	**LN+ N=10**	**LN- N=7**	**All[3] N=28**	**N=7**	**LN+ N=10**	**LN- N=7**	**All[3] N=28**
**1**	9p21.3	*CDKN2A/B*	***CDKN2A****	0	20	29	29	29	70	43	50
**2**	3p14.2	*FHIT*	***FHIT***	71	100	71	86	14	20	29	32
**3**	16q23.1	*WWOX*	***WWOX***	29	40	29	32	0	0	14	7
**4**	9p24.1	*PTPRD*	***PTPRD***	43	60	57	61	0	30	14	25
**7**	13q14.2	*RB1*	***RB1****	14	40	29	32	0	0	0	0
**8**	2q22.1	*LRP1B*	***LRP1B***	29	50	57	50	0	20	29	29
**9**	4q35.2		*FRG2*	29	40	29	32	0	0	0	0
**10**	5q11.2	*PDE4D*	*PLK2*	14	10	0	13.7	0	0	0	4
			PDE4D	57	20	29	29	0	0	29	14
**12**	7q34	*TRB*	***PRSS1***	0	20	14	25	0	0	0	0
**14**	19p13.3		***GZMM***	29	40	14	25	0	0	0	0
			THEG	29	30	14	25	0	0	0	0
			PPAP2C*	29	20	14	21	0	0	0	0
			C19orf20	29	40	0	21	0	0	0	0
**16**	8p23.2	*CSMD1*	*CSMD1*	71	70	86	75	29	20	14	18
**19**	18q23		*PARD6G*	14	60	14	50	0	0	0	4
**23**	11q25	*OPCML NTM*	***OPCML***	14	70	29	57	0	10	0	11
			NTM	14	70	29	57	0	10	0	11
**24**	13q12.11		*LATS2*	14	40	29	32	0	0	0	0
**25**	22q13.33		*TUBGCP6*	14	30	14	14	0	0	0	4
**30**	8p23.3		*ZNF596*	14	60	57	54	0	0	0	4
**34**	11q23.1	*ATM*	***CADM1***	14	70	43	61	0	10	0	11
			ATM	14	60	29	43	0	0	0	0
**35**	9p24.3		*FOXD4*	14	40	14	36	0	0	0	0
**40**	3p26.3		***CHL1***	57	80	43	57	0	10	0	11
**47**	8p11.22		***ZMAT4***	43	60	43	47	0	0	0	4
			C8orf4	43	50	43	43	0	0	0	4

**49**	10p15.3		*TUBB8*	0	40	29	50	0	0	0	0
**50**	1p31.1	*NEGR1*	***NEGR1***	14	40	0	29	0	0	0	4
**51**	13q31.3	*GPC6*	***DCT***	14	20	14	18	0	0	0	0
			GPC6	14	40	14	29	0	0	0	0
**61**	7q35	*CNTNAP2*	***CNTNAP2****	0	40	29	46	0	0	0	4
**62**	14q32.12		*PRIMA1*	0	30	14	18	0	0	0	0
**66**	19p12		***ZNF492***	29	50	14	36	0	0	0	0
			ZNF99	14	50	14	40	0	0	0	0
**67**	12q23.1	*ANKS1B*	***ANKS1B***	14	30	14	29	0	0	0	4
	4p16.3		***ZNF141***	14	40	14	36	0	0	0	0
**68**	8p21.2		***DPYSL2****	57	50	71	61	0	10	0	4
			STMN4	57	50	57	57	0	0	0	0
**74**	11q14.1	*DLG2*	*TMEM126A*	14	50	14	32	0	0	0	0
			CCDC89	14	50	14	32	0	0	0	0
			CCDC90B*	14	50	0	29	0	0	0	0
**79**	21q22.2	*DSCAM*	***DSCAM***	14	50	29	46	0	0	0	0
		ERG	***ERG***	0	40	14	36	0	0	0	0
**80**	18q21.2	*SMAD4*	***SMAD4****	14	80	14	54	0	0	0	7
		DCC	***DCC***	29	60	29	54	0	20	0	21

Table 1

				Low Copy Number Gain Frequency (%)				High Copy Number Gain Frequency (%)			
				PMOL	OSCC			PMOL	OSCC		
Rank[1]	Chromosome band	Known target	GRAIL Top Target[2]	N=7	LN+ N=10	LN- N=7	All[3] N=28	N=7	LN+ N=10	LN- N=7	All[3] N=28
1	8q24.21	MYC	MYC*	43	100	71	75	0	0	0	4
				0	20	14	29	0	60	0	36
2	11q13.2	CCND1	ORAOV1	0	20	14	29	0	60	0	36
5	14q13.3	NKX2-1	NKX2-1	14	60	0	36	0	0	0	0
7	7p11.2	EGFR	EGFR**	29	30	71	46	0	0	14	14
13	12q15		LRRC10	0	20	14	11	0	10	0	4
14	12q14.3	HMGA2	HMGA2*	14	40	14	18	0	10	0	4
16	5p15.33	TERT	TERT	29	60	57	54	0	0	0	0
17	3q26.2	PRKCI**	PRKCI**	14	80	43	64	0	20	0	7
19	20q13.2		ZNF217**	43	60	29	57	0	0	0	0
26	11q14.1		GAB2	0	20	14	29	0	20	0	7
27	20q13.33		BIRC7*	43	60	43	61	0	0	0	0
			ZNF704	43	70	29	57	0	0	0	0
30	8q21.13		ZBTB10	43	70	29	54	0	0	0	0
			ID1	57	80	29	61	0	0	0	0
33	20q11.21	BCL2L1	BCL2L1**	71	90	29	68	0	0	0	0
39	17q25.1		GRB2*	14	40	14	32	0	0	0	0
43	8q24.11		NOV	43	90	57	71	0	0	0	0
49	3q28	PIK3CA	PIK3CA**	14	90	43	71	0	10	0	4
50	1p36.33		TP73	0	30	14	0	0	0	0	0
54	8q22.3		RRM2B	43	80	43	61	0	0	0	0
			PTP4A3	43	100	71	82	0	0	0	0
			MAFA	43	90	71	75	0	0	0	0
67	8q24.3		PARP10	57	90	57	79	0	10	14	7

Table 2

SUPPLEMENTARY DATA S12 - FREQUENCY OF COPY NUMBER ALTERATIONS OF INTOGEN-DERIVED CANCER DRIVER GENES IN PPOLS and HNSCC CELL LINES

The following tables show frequency of SCNAs in genes identified as cancer drivers through IntOgen analyses (version 2014.12; http://www.intogen.org).

Abbreviations:

CN- copy number

PPOL- potentially premalignant oral lesions

HNSCC- head and neck squamous cell carcinoma

LN +VE- Lymph node metastases positive

LN-VE- Lymph node metastases negative

Table 1: Cancer drivers showing low copy number gains (≤ 2) ordered by frequency in LN+ve HNSCC cell lines with minimum frequency of 40%

Table 2: Cancer drivers showing low copy number gains (≤2) ordered by frequency in LN-ve HNSCC cell lines with minimum frequency of 40%

Table 3: Cancer drivers showing high copy number gains (>2) ordered by frequency in LN+ve HNSCC cell lines with minimum frequency of 10%

Table 4: Cancer drivers showing high copy number gains (>2) ordered by frequency in LN-ve HNSCC cell lines with minimum frequency of 10%

Table 5: Cancer drivers showing hemizygous loss ordered by frequency in LN+ve HNSCC cell lines with minimum frequency of 40%

Table 6: Cancer drivers showing hemizygous loss ordered by frequency in LN-ve HNSCC cell lines with minimum frequency of 40%

Table 7: Cancer drivers showing homozygous loss ordered by frequency in LN+ve HNSCC cell lines.

Table 1: Cancer drivers showing low copy number gains (≤ 2) ordered by frequency in LN+ve HNSCC cell lines with minimum frequency of 40%

TERM	PPOL %	LN-VE %	LN +VE %	ALL HNSCC %
MECOM	28.57	42.86	100	71.43
MYC	42.86	71.43	100	75
NDRG1	42.86	57.14	100	71.43
PIK3CA	14.29	42.86	90	71.43
FXR1	28.57	42.86	90	71.43
TBL1XR1	14.29	42.86	90	67.86
MUC20	28.57	57.14	90	71.43
EIF4G1	14.29	42.86	90	71.43
RAD21	42.86	57.14	90	71.43
DLG1	28.57	57.14	90	67.86
RFC4	14.29	42.86	90	67.86
EIF4A2	14.29	42.86	90	67.86
TFDP2	14.29	28.57	80	53.57
ARFGEF2	57.14	57.14	80	71.43
STK4	57.14	57.14	80	71.43
CHD6	57.14	42.86	80	67.86
RASA2	14.29	28.57	80	53.57
XRN1	14.29	28.57	70	50
PABPC1	42.86	57.14	70	64.29
ARFGEF1	42.86	42.86	70	60.71
PIK3CB	14.29	28.57	70	50
CRNKL1	71.43	14.29	70	46.43
ATR	14.29	28.57	60	46.43
STAG1	14.29	14.29	60	42.86
PSMA6	14.29	0	60	35.71
TRIO	42.86	57.14	60	60.71
CCT5	28.57	57.14	60	53.57
GNAS	71.43	42.86	60	60.71
PLCG1	71.43	42.86	60	60.71
DHX35	28.57	42.86	60	57.14
PLCB1	71.43	28.57	60	46.43
FOXA2	71.43	28.57	60	42.86
PLXNA1	42.86	28.57	60	46.43
NCK1	14.29	14.29	60	42.86
MAX	0	14.29	50	35.71
MLH3	0	14.29	50	35.71
SOS2	14.29	0	50	32.14
GNG2	0	0	50	32.14
ZFP36L1	0	14.29	50	42.86
ARID4A	14.29	0	50	32.14
MAP3K11	0	28.57	50	39.29
CHD9	14.29	0	50	28.57
FOXA1	14.29	0	50	28.57

HDAC9	42.86	85.71	50	60.71
CSNK2A1	57.14	28.57	50	42.86
ASXL1	28.57	28.57	50	53.57
CTTN	0	14.29	40	28.57
MEN1	0	14.29	40	39.29
AHR	42.86	85.71	40	53.57
ACTG1	14.29	28.57	40	42.86
STIP1	0	14.29	40	35.71
GNAI1	42.86	42.86	40	39.29
TNPO1	42.86	14.29	40	17.86
CEP290	28.57	14.29	40	17.86
EFTUD2	0	14.29	40	32.14
NFATC4	0	0	40	28.57
SPTAN1	14.29	85.71	40	60.71
ARFGAP1	14.29	42.86	40	53.57
SRGAP1	14.29	14.29	40	17.86
KALRN	14.29	14.29	40	28.57

Table 2: Cancer drivers showing low copy number gains (≤2) ordered by frequency in LN-ve HNSCC cell lines with minimum frequency of 40%

TERM	PPOL %	LN-VE %	LN +VE %	ALL HNSCC %
HDAC9	42.86	85.71	50	60.71
AHR	42.86	85.71	40	53.57
SPTAN1	14.29	85.71	40	60.71
RAC1	14.29	85.71	30	64.29
SYK	14.29	85.71	20	50
PTCH1	0	85.71	20	46.43
SVEP1	14.29	85.71	20	50
RGS3	0	85.71	20	46.43
MYC	42.86	71.43	100	75
ACTB	28.57	71.43	30	53.57
PCSK5	0	71.43	30	46.43
TSC1	0	71.43	30	50
EGFR	28.57	71.43	30	46.43
TJP2	14.29	71.43	30	42.86
ADCY1	14.29	71.43	30	46.43
PPP6C	0	71.43	30	50
SCAI	14.29	71.43	30	50
NOTCH1	14.29	71.43	20	53.57
KLF4	14.29	71.43	20	42.86
RAD23B	0	71.43	20	42.86
NTRK2	0	71.43	10	39.29
NDRG1	42.86	57.14	100	71.43
MUC20	28.57	57.14	90	71.43
RAD21	42.86	57.14	90	71.43
DLG1	28.57	57.14	90	67.86
ARFGEF2	57.14	57.14	80	71.43
STK4	57.14	57.14	80	71.43
PABPC1	42.86	57.14	70	64.29
TRIO	42.86	57.14	60	60.71
CCT5	28.57	57.14	60	53.57
PTGS1	0	57.14	30	42.86
MECOM	28.57	42.86	100	71.43
PIK3CA	14.29	42.86	90	71.43
FXR1	28.57	42.86	90	71.43
TBL1XR1	14.29	42.86	90	67.86
EIF4G1	14.29	42.86	90	71.43
RFC4	14.29	42.86	90	67.86
EIF4A2	14.29	42.86	90	67.86
CHD6	57.14	42.86	80	67.86
ARFGEF1	42.86	42.86	70	60.71
GNAS	71.43	42.86	60	60.71
PLCG1	71.43	42.86	60	60.71

DHX35	28.57	42.86	60	57.14
GNAI1	42.86	42.86	40	39.29
ARFGAP1	14.29	42.86	40	53.57
AKAP9	0	42.86	30	35.71
CUX1	0	42.86	10	35.71

Table 3: Cancer drivers showing high copy number gains (>2) ordered by frequency in LN+ve HNSCC cell lines with minimum frequency of 10%

TERM	LN-VE %	LN +VE %	ALL HNSCC %
CCND1	0	60	35.71
CTTN	0	40	35.71
XRN1	0	20	7.14
ATR	0	20	7.14
NOTCH1	0	20	7.14
ACTB	0	10	14.29
INPPL1	0	10	10.71
MAX	0	10	7.14
MLH3	0	10	7.14
RAC1	0	10	7.14
PCSK5	14.29	10	7.14
CUX1	0	10	7.14
MECOM	0	10	3.57
PIK3CA	0	10	3.57
FXR1	0	10	3.57
TBL1XR1	0	10	3.57
TFDP2	0	10	3.57
STAG1	0	10	3.57
SOS2	0	10	3.57
GNG2	0	10	3.57
ZFP36L1	0	10	3.57
ARID4A	0	10	3.57
AKAP9	0	10	3.57
TSC1	0	10	3.57
CEBPA	0	10	3.57
ZNF814	0	10	3.57
SYK	0	10	3.57
PTCH1	0	10	3.57
SRGAP3	0	10	3.57
VHL	0	10	3.57
NTRK2	0	10	3.57
BMPR2	0	10	3.57
NUP107	0	10	3.57
MED17	0	10	3.57
XPO1	0	10	3.57

Table 4: Cancer drivers showing high copy number gains (>2) ordered by frequency in LN-ve HNSCC cell lines with minimum frequency of 10%

TERM	LN-VE %	LN +VE %	ALL HNSCC %
PCSK5	14.29	10	7.14
EGFR	14.29	0	14.29
ERBB2	14.29	0	3.57
KLF4	14.29	0	3.57
RAD23B	14.29	0	3.57

Table 5: Cancer drivers showing hemizygous loss ordered by frequency in LN+ve HNSCC cell lines with minimum frequency of 40%

TERM	PPOL %	LN-VE %	LN+VE %	ALL HNSCC %
FOXP1	42.86	85.71	100	89.27
SETD2	57.14	57.14	100	75
CTNNB1	57.14	42.86	100	75
ITGA9	57.14	42.86	100	67.86
MLH1	57.14	42.86	100	67.86
ROBO2	42.86	85.71	90	82.14
RBM5	57.14	57.14	90	67.86
RHOA	42.86	57.14	90	67.86
CLASP2	57.14	42.86	90	64.29
WNT5A	42.86	42.86	90	67.86
CAPN7	57.14	28.57	90	57.14
MYD88	42.86	28.57	90	60.71
SMAD4	14.29	14.29	80	53.57
NEDD4L	28.57	14.29	80	57.14
PBRM1	57.14	57.14	80	64.29
BAP1	57.14	57.14	80	64.29
SRGAP3	57.14	28.57	80	53.57
TGFBR2	28.57	28.57	80	57.14
TCF4	28.57	28.57	70	53.57
SMAD2	14.29	14.29	70	53.57
ACAD8	14.29	28.57	60	53.57
TET2	28.57	42.86	60	64.29
HSPA8	14.29	42.86	60	50
VHL	42.86	28.57	60	46.43
ATM	14.29	28.57	60	42.86
CASP1	0	14.29	60	35.71
MLL	14.29	14.29	60	42.86
MSR1	57.14	85.71	50	57.14
RUNX1	0	14.29	50	35.71
FAT1	42.86	42.86	50	53.57
PGR	14.29	28.57	50	35.71
NKX3-1	57.14	71.43	50	60.71
BNC2	14.29	57.14	50	42.86
GATA3	0	57.14	50	64.29
EPC1	0	57.14	50	60.71
PSIP1	14.29	42.86	50	39.29
CUL2	0	42.86	50	57.14
IRF2	42.86	42.86	50	46.43
PABPC3	14.29	28.57	50	35.71
DHX15	14.29	14.29	50	39.29
SEC24D	0	14.29	50	46.43
MED17	14.29	0	50	28.57
RPSAP58	28.57	0	50	32.14
PCDH18	28.57	28.57	40	46.43
POLR2B	14.29	28.57	40	42.86
STARD13	14.29	42.86	40	32.14
VIM	0	42.86	40	53.57
LCP1	14.29	28.57	40	32.14
RB1	14.29	28.57	40	32.14
FRG1	28.57	28.57	40	32.14
FLT3	28.57	28.57	40	32.14
BRCA2	28.57	28.57	40	28.57

WASF3	14.29	28.57	40	32.14
CLOCK	14.29	14.29	40	39.29
KIT	14.29	14.29	40	39.29
FIP1L1	28.57	14.29	40	39.29
G3BP2	28.57	14.29	40	46.43
ITSN1	14.29	14.29	40	28.57
FBXW7	28.57	14.29	40	46.43
HNRPDL	14.29	14.29	40	46.43
BRWD1	14.29	14.29	40	35.71
KDR	14.29	0	40	35.71
SEC31A	14.29	0	40	42.86

Table 6: Cancer drivers showing hemizygous loss ordered by frequency in LN-ve HNSCC cell lines with minimum frequency of 40%

TERM	PPOL %	LN-VE %	LN+VE %	ALL HNSCC %
FOXP1	42.86	85.71	100	89.27
ROBO2	42.86	85.71	90	82.14
MSR1	57.14	85.71	50	57.14
NKX3-1	57.14	71.43	50	60.71
ATIC	0	71.43	10	21.43
SETD2	57.14	57.14	100	75
RBM5	57.14	57.14	90	67.86
RHOA	42.86	57.14	90	67.86
PBRM1	57.14	57.14	80	64.29
BAP1	57.14	57.14	80	64.29
BNC2	14.29	57.14	50	42.86
GATA3	0	57.14	50	64.29
EPC1	0	57.14	50	60.71
FN1	0	57.14	10	17.86
IDH1	0	57.14	0	14.29
EEF1B2	0	57.14	0	14.29
CUL3	0	57.14	0	14.29
CTNNB1	57.14	42.86	100	75
ITGA9	57.14	42.86	100	67.86
MLH1	57.14	42.86	100	67.86
CLASP2	57.14	42.86	90	64.29
WNT5A	42.86	42.86	90	67.86
TET2	28.57	42.86	60	64.29
HSPA8	14.29	42.86	60	50
FAT1	42.86	42.86	50	53.57
PSIP1	14.29	42.86	50	39.29
CUL2	0	42.86	50	57.14
IRF2	42.86	42.86	50	46.43
STARD13	14.29	42.86	40	32.14
VIM	0	42.86	40	53.57
KLF6	0	42.86	30	46.43
MAP2K4	28.57	42.86	10	25
EPHA4	0	42.86	10	14.29

Table 7: Cancer drivers showing homozygous loss ordered by frequency in LN+ve HNSCC cell lines.

TERM	PPOL %	LN -VE %	LN+VE %	ALL HNSCC %
CDKN2A	28.57	42.86	70	50
MLL3	0	0	20	10.71
NSD1	0	0	20	7.14
ROBO2	0	0	10	14.29
MSR1	0	0	10	10.71
ACAD8	0	0	10	7.14
ERBB2IP	0	0	10	7.14
TCF4	14.29	0	0	10.71
FOXP1	0	14.29	0	7.14
SMAD4	0	0	0	7.14
RUNX1	0	0	0	7.14
PCDH18	0	0	0	7.14
CDC73	0	0	0	7.14
NEDD4L	0	0	0	3.57
FAT1	14.29	0	0	3.57
PGR	0	0	0	3.57
POLR2B	0	0	0	3.57
WHSC1L1	0	0	0	3.57
FGFR1	0	0	0	3.57
PLXNB2	0	0	0	3.57
MEF2C	0	0	0	3.57
MAP3K1	0	0	0	3.57

SUPPLEMENTARY DATA 13 – DELETIONS IN HNSCC CELL LINES AT THE *CSDM1* LOCUS

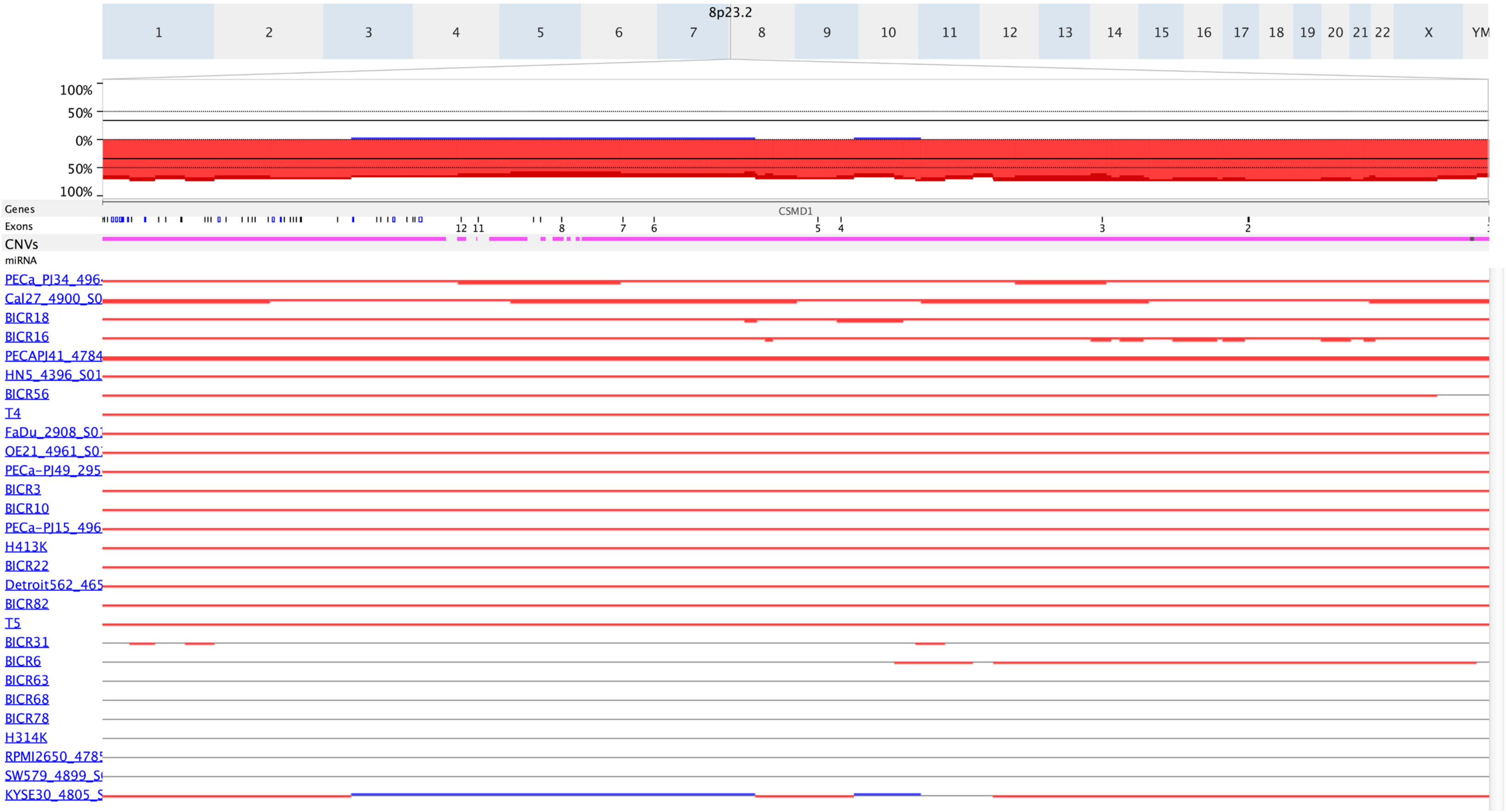

Deletions in HNSCC cell lines at *CSDM1* locus. Red areas indicate extent of deletions. Dark red/thicker lines indicate regions of homozygous deletions. Blue lines indicate regions of amplification and grey lines a lack of SCNA.

SUPPLEMENTARY DATA 14 - MODULATION OF *CSMD1* EXPRESSION IN IMMORTAL HNSCC CELL LINES

A

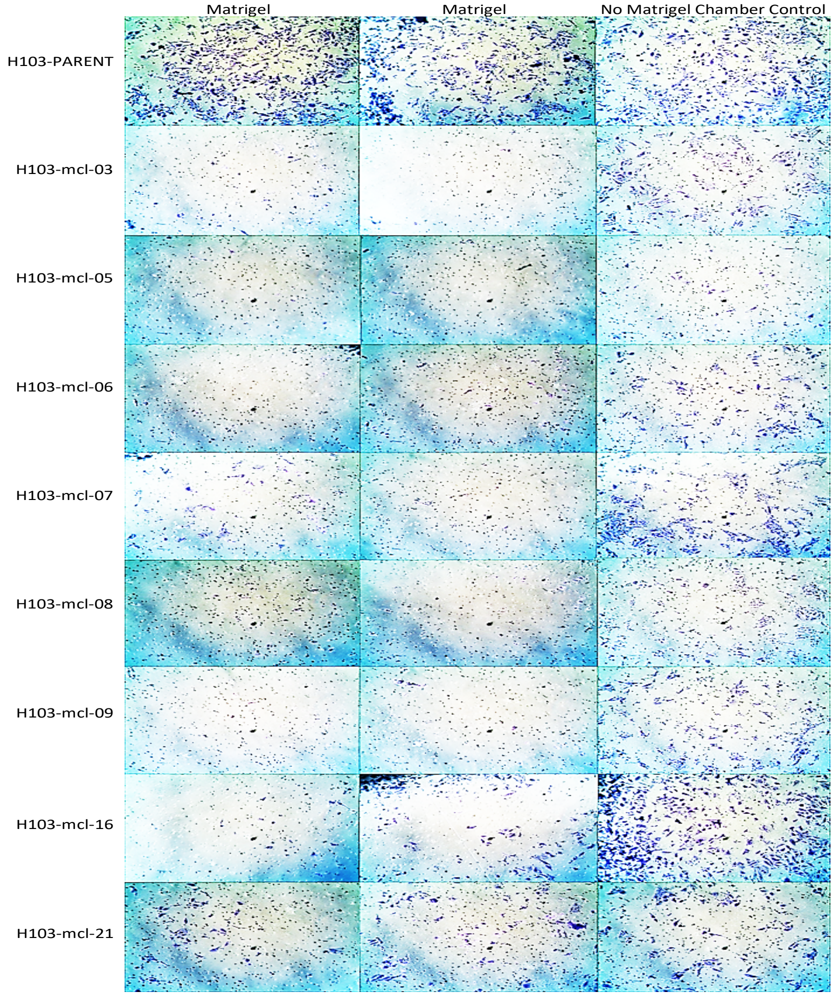
Matrigel
Matrigel
No Matrigel Chamber Control
H103-PARENT
H103-mcl-03
H103-mcl-05
H103-mcl-06
H103-mcl-07
H103-mcl-08
H103-mcl-09
H103-mcl-16
H103-mcl-21

B

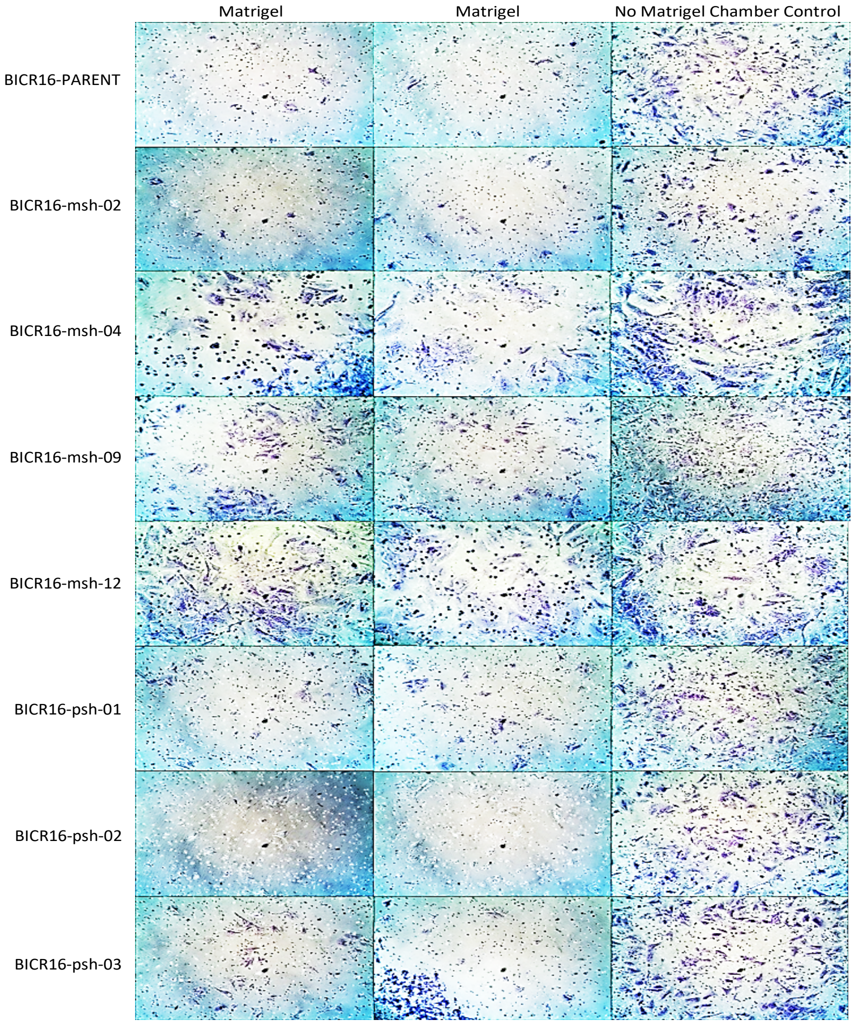

Matrigel
Matrigel
No Matrigel Chamber Control
BICR16-PARENT
BICR16-msh-02
BICR16-msh-04
BICR16-msh-09
BICR16-msh-12
BICR16-psh-01
BICR16-psh-02
BICR16-psh-03

A. Two trans-well chambers of all *CSMD1*-expressing clones and *CSMD1*-negative H103 parent cell line are displayed together with no matrigel controls. *CSMD1* expression results in a marked decrease of gel invasion ($p=5.975x10^{-5}$). B. Two trans-well chambers of all *CSMD1*-silenced clones and *CSMD1*-expressing BICR16 parent cell line are displayed together with no matrigel controls. *CSMD1* silencing results in a variable marked increase in gel invasion ($p=1.822x10^{-05}$).

SUPPLEMENATRY DATA S15 - ANALYSIS OF *NCKAP5*

Deletions in *NCKAP5* in our dataset are shown in Figure 1. Homozygous deletions were present in both immortal PPOL and HNSCC cell lines and the homozygous deletions eliminated one or more exons in 4 out of the 5 cell lines (Fig 1) with the remaining cell line sustaining two intronic deletions. We looked for deletions of *NCKAP5* in our array-CGH dataset of 347 tumour cell lines (unpublished) and in Tumorscape (Release 1.6). In our dataset (not shown), only colorectal cell lines bore homozygous deletions of *NCKAP5* and these were rare (2 of 161 or ~1.2%). Both deletions involved exon 3. In Tumorscape, *NCKAP5* is significantly focally deleted across the entire dataset of 3131 tumours but is not located within a focal peak region of deletion. It is significantly focally deleted in 2 of 14 independent subtypes analyzed in Tumorscape including all epithelial cancers (Q=4.33E6, Frequency=0.12). It is not within the focal peak region of deletion in any of the individual tumour types and deletions are not frequent (<20%), and homozygous deletions are exceedingly rare (<1%). Analyses of variants listed in COSMIC data show few pathological mutations as well as misense mutations predicted to be pathological (Table 1).

We analysed expression of *NCKAP5* transcript in small number of paired tumour and normal HNSCC samples as well as a panel of other tumour types (Figure 2) Marked reduced expression compared to normal tissues was seen in about ~26% of the tumours with remainder showing little change or more commonly overexpression. Analyses of expression using TissueScan Cancer and Normal Cancer Survey 96-I cDNA Arrays (Origene, Maryland, USA)

showed reduced expression in tumours of thyroid, lung, liver and kidney (Figure 3).

Thus, *NCKAP5* is unlikely to be frequent target for inactivation in HNSCC but the related pathways may be more significant. NCKAP5 has been shown to interact with NCK1 (STRING Release 9.1), an adaptor protein important in ligand-induced activation of receptor tyrosine kinases and also APC (BioGRID Release 3.3). Our IntOGene (Release 2014.12) analyses showed a low frequency of pan-cancer gain-of-function mutations in *NCK1*. In this study, *NCK1* was within an extended GISTIC region and copy number gains were seen in 60% of LN+ve cell lines with relatively low frequency (~14%) gains in PPOL and LN-ve cell lines.

Figure 1. Hemizygous and homozygous deletions at *NCKAP5* locus

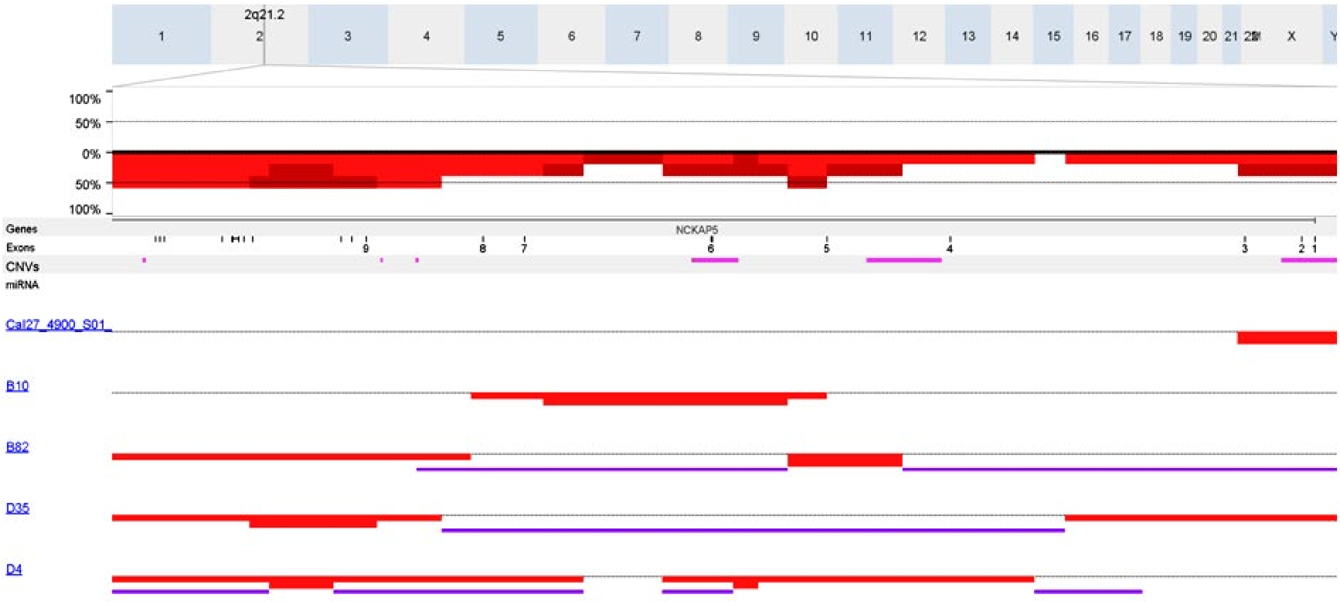

Hemizygous (thin red line) and homozygous deletions (thickred line).

Table 1. Analysis of *NCKAP5* mutations listed in COSMIC

NCKAP5 (PolyPhen-2)			PANTHER	SIFT	Tissue Type	Histology
Amino acid position	Prediction	Score	$P_{deleterious}$			
p.K183R	benign	0.058	0.13865	TOLERATED	haematopoietic and lymphoid tissue	lymphoid neoplasm
p.A198V	benign	0.032	0.72406	DAMAGING	colon	carcinoma
p.P316H	benign	0.023	0.10065	TOLERATED	colon	carcinoma
p.T556M	benign	0.001	0.24661	TOLERATED	colon	carcinoma
p.Q560R	benign	0.01	0.36801	TOLERATED	Not specified	not specified
p.V754A	benign	0	0.09554	TOLERATED	caecum	carcinoma
p.S890G	benign	0.05	0.65865	DAMAGING	colon	carcinoma
p.T1056I	benign	0.012	0.45163	TOLERATED	Not specified	malignant melanoma
p.A1137D	benign	0.435	0.35013	TOLERATED	rectum	carcinoma
p.D1192G	benign	0.01	0.517	TOLERATED	colon	carcinoma
p.I1201V	benign	0	0.19913	TOLERATED	prostate	carcinoma
p.A1329S	benign	0.101	0.09918	TOLERATED	autonomic ganglia	neuroblastoma
p.S1346F	benign	0.002	0.15902	TOLERATED	caecum	carcinoma
p.A1537V	benign	0.021	0.35217	TOLERATED	rectum	carcinoma
p.A1863T	benign	0.007	0.15675	TOLERATED	colon	carcinoma
p.L36P	probably damaging	1	0.61759	*DAMAGING	colon	carcinoma
p.S172R	probably damaging	0.96	0.28558	TOLERATED	Not specified	not specified
p.R212Q	probably damaging	0.993	0.7559	DAMAGING	colon	carcinoma
p.T354M	probably damaging	1	0.6481	*DAMAGING	colon	carcinoma
p.D357N	probably damaging	1	0.41471	*DAMAGING	skin	malignant melanoma
p.S382I	probably damaging	0.986	0.74926	*DAMAGING	caecum	carcinoma
p.G404W	probably damaging	1	0.94376	*DAMAGING	colon	carcinoma
p.T538I	possibly damaging	0.617	0.3067	TOLERATED	colon	carcinoma
p.C544Y	possibly damaging	0.617	0.35693	TOLERATED	brain	atypical teratoid-rhabdoid tumour
p.L620F	probably damaging	0.959	0.52218	*DAMAGING	Not specified	malignant melanoma
p.S662Y	probably damaging	0.999	0.84437	*DAMAGING	rectum	carcinoma
p.K813I	probably damaging	1	0.79085	DAMAGING	skin	malignant melanoma
p.H874R	possibly damaging	0.779	0.14482	TOLERATED	breast	carcinoma
p.K1272Q	probably damaging	1	0.56744	TOLERATED	larynx	carcinoma
p.D1284Y	probably damaging	0.993	0.64532	DAMAGING	colon	carcinoma
p.T1288M	probably damaging	0.961	0.52143	TOLERATED	caecum	carcinoma
p.G1293W	probably damaging	1	0.96618	DAMAGING	prostate	carcinoma
p.S1311Y	probably damaging	1	0.90495	DAMAGING	caecum	carcinoma
p.P1558R	possibly damaging	0.834	0.18314	TOLERATED	rectum	carcinoma
p.S1650Y	probably damaging	1	0.66785	DAMAGING	rectum	carcinoma
p.H356H	silent mutation			TOLERATED	caecum	carcinoma
p.T610T	silent mutation			TOLERATED	caecum	carcinoma
p.C540C	silent mutation			TOLERATED	prostate	carcinoma
p.A1674A	silent mutation			TOLERATED	rectum	carcinoma
p.D1681D	silent mutation			TOLERATED	breast	carcinoma
p.S1872S	silent mutation			TOLERATED	colon	carcinoma
p.L1478*	Nonsense mutation			DAMAGING	colon	carcinoma
p.C1654*	Nonsense mutation			DAMAGING	caecum	carcinoma
p.C452fs*21	Frameshift deletion			DAMAGING	colon	carcinoma

*Stransky et al. , Science 333 (6046) 1157-1160

Figure 2. Expression analyses of *NCKAP5* in HNSCC

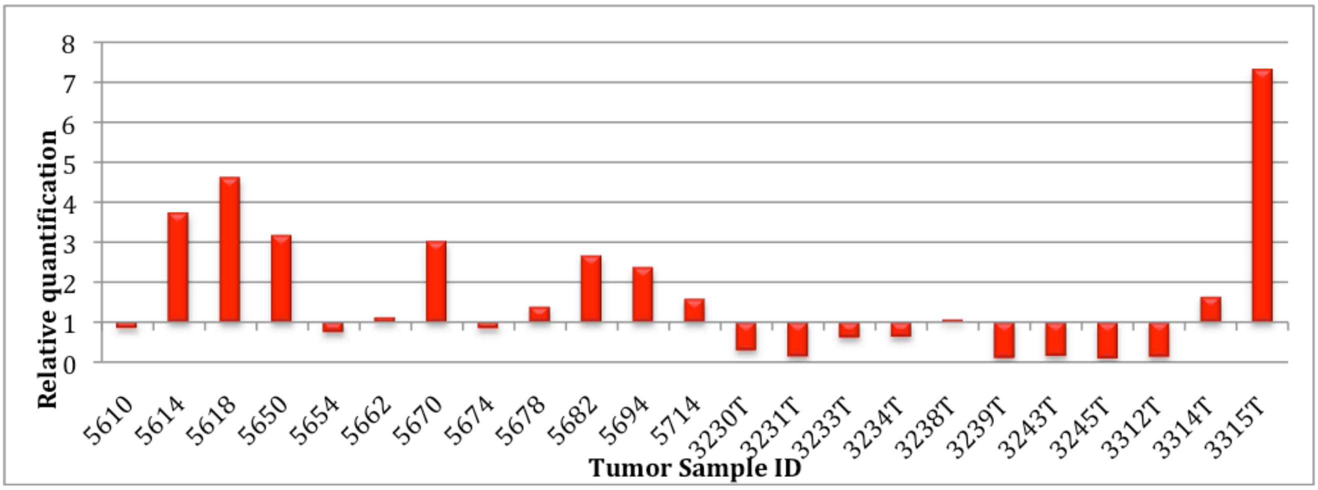

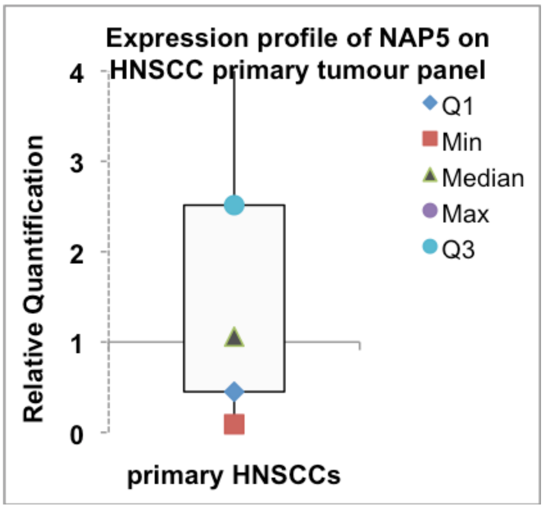

Analysis of *NCKAP5* . Relative quantification of expression shown as fold change was done by comparing the tumour samples against the available normal samples (which is set at value 1).

Top Panel: Expression profile of *NCKAP5* in primary HNSCC and normal oral mucosa. The y-axis represents the change in the relative fold expression of the tumour samples over the mean value of the normal tissue. A relative decrease in expression of *NCKAP5* was seen in 48% (11/23) of primary HNSCCs when compared with their adjacent normal mucosa samples. However, significant reduction was observed in only 26% (6/23) of primary HNSCCs

Bottom Panel: Expression profile of *NCKAP5* in primary HNSCCs compared with the normal tissue. The x-axis represents the primary HNSCC samples in the study. The y-axis represents the change in the relative fold expression compared to the mean expression in the normal samples. The mean expression values for the normal samples is averaged and normalized to 1 (Line on horizontal x-axis at value 1) against which, expression values for the

primary HNSCCs are compared and an average fold change calculated. The box-plots show the first quartile, minimum, median, maximum and third quartile for each group of samples.

Figure 3. Expression analyses of *NCKAP5* in other tumour types

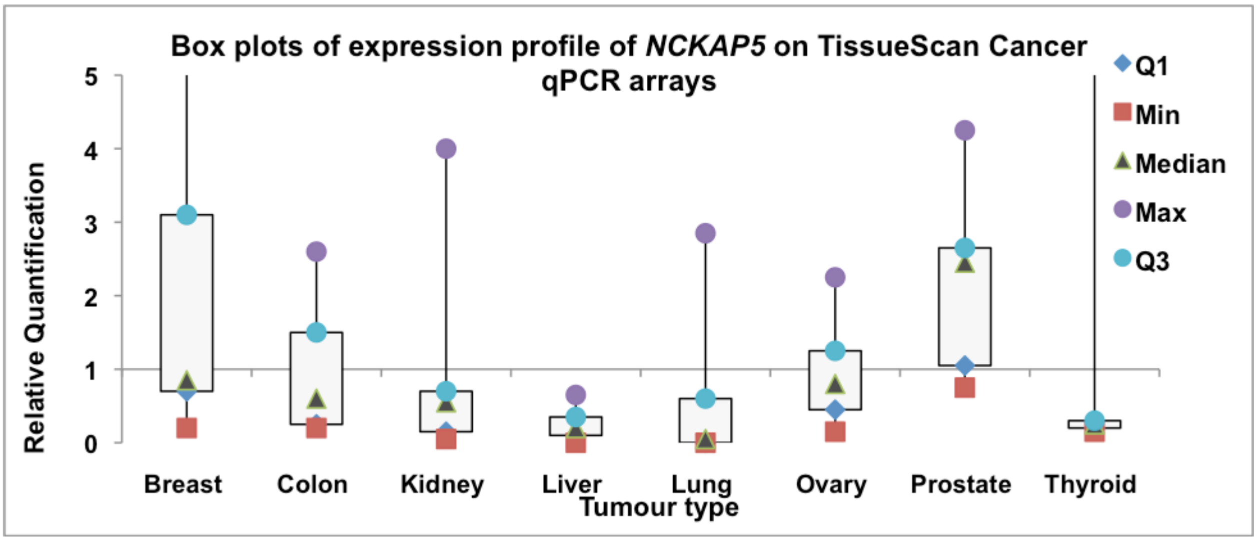

Normalised expression profile of *NCKAP5* on TissueScan Cancer and Normal Tissue Cancer Survey 96-I cDNA Arrays (Origene Maryland, USA). The panel includes 12 tumour and 3 normal tissue samples from each of 8 different sites. The y-axis represents the change in the relative fold expression compared to the mean expression in the 3 normal tissues for each tumour type. The box-plots show the first quartile, minimum, median, maximum and third quartile for each group of samples. There is a relative decrease in expression of *NCKAP5* in all tumours except for those from breast and prostate.

SUPPLEMENTARY DATA S16 - INTEGRATIVE ANALYSIS OF SOMATIC COPY NUMBER CHANGES AND GENE EXPRESSION

Table 1. Copy number gains in PPOLS and HNSCC of genes that show correlation with expression in SCNA regions showing significant difference between PPOLS and HNSCCs.

Chromosomal coordinates	Genes ordered by strength of correlation	Copy number gain Frequency (%) PPOL	Copy number gain Frequency (%) OSCC LN+	Copy number gain Frequency (%) OSCC LN-	Copy number gain Frequency (%) OSCC All[3]	High copy number gain Frequency (%) PPOL	High copy number gain Frequency (%) OSCC LN+	High copy number gain Frequency (%) OSCC LN-	High copy number gain Frequency (%) OSCC All[3]
		N=7	N=10	N=7	N=28	N=7	N=10	N=7	N=28
chr3:57677987-	*FLNB**	0	0	0	0	0	0	0	0
chr3:61422777-	*UBA3**	14.29	0	0	0	0	0	0	0
	SUCLG2	14.29	0	0	0	0	0	0	0
	*ARL6IP5**	14.29	0	0	0	0	0	0	0
	ATXN7	14.29	0	0	0	0	0	0	0
	*THOC7**	14.29	0	0	0	0	0	0	0
	C3ORF64	14.29	0	0	0	0	0	0	0
	*PSMD6**	14.29	0	0	0	0	0	0	0
chr3:87035206-	*CHMP2B**	14.29	0	14.29	7.14	0	0	0	0
	*CGGBP1**	14.29	0	14.29	3.57	0	0	0	0
chr3:151842842-	*SIAH2**	14.29	80	28.57	60.71	0	0	0	0
chr3:157160169-	*CCNL1***	14.29	70	42.86	57.14	0	10	0	7.14
chr3:160599782-	*SCHIP1**	14.29	90	42.86	67.86	0	10	0	3.57
chr3:161705726-	*KPNA4**	14.29	70	42.86	60.71	0	10	0	3.57
chr3:170691970-	*MYNN**	14.29	80	42.86	64.29	0	10	0	3.57
	*TNIK**	14.29	100	42.86	71.43	0	10	0	3.57
	SLC31A1	0	30	85.71	50	0	0	0	0
	*SEC62**	14.29	90	42.86	67.86	0	0	0	0
	*EIF5A2**	14.29	90	42.86	67.86	0	10	0	3.57
chr3:177707736-	*PIK3CA***	14.29	90	42.86	71.43	0	10	0	3.57
chr3:185035692-	*AP2M1*	14.29	90	42.86	71.43	0	0	0	0
	PARL	14.29	90	42.86	67.86	0	0	0	0
	*ALG3**	14.29	90	42.86	71.43	0	0	0	3.57
	*EIF4G1***	14.29	90	42.86	71.43	0	0	0	3.57
	*DVL3***	14.29	90	42.86	67.86	0	0	0	0
	*ABCF3**	14.29	90	42.86	71.43	0	0	0	0
chr3:198578155-	*RPL35A**	14.29	90	42.86	64.29	0	0	0	0
chr7:1631815-7317208	*EIF2AK1**	14.29	40	85.71	67.86	0	10	0	7.14
	C7ORF28A	28.57	40	85.71	67.86	0	10	0	7.14
	*RAC1***	14.29	30	85.71	64.29	0	10	0	7.14
	*CHST12**	14.29	30	71.43	60.71	0	10	0	10.71
	KDELR2	14.29	40	85.71	67.86	0	10	0	10.71
	*RNF216**	28.57	40	71.43	64.29	0	10	0	14.29
	*AIMP2**	14.29	40	85.71	67.86	0	10	0	7.14

	*NUDT1**	14.29	30	71.43	60.71	0	10	0	7.14
	WIPI2	14.29	40	85.71	67.86	0	10	0	7.14
	PMS2CL	14.29	30	85.71	60.71	0	10	0	7.14
chr9:85367221-	*RMI1**	0	20	71.43	39.29	0	10	0	3.57
	*HNRNPK***	0	20	71.43	39.29	0	10	0	3.57
chr9:97596715-	*CDC14B**	0	40	71.43	53.57	0	10	14.29	7.14
chr9:99427940-	*ANP32B**	0	20	85.71	46.43	0	0	0	0
chr9:101504820-	*ERP44***	14.29	30	71.43	46.43	0	0	14.29	3.57
chr9:123376788-	*RABGAP1*	0	30	57.14	42.86	0	0	0	0
	RC3H2	0	30	57.14	42.86	0	0	0	0
chr9:126825879-	*GAPVD1*	14.29	30	71.43	46.43	0	0	0	0
	*PPP6C**	0	30	71.43	50	0	0	0	0
	RABEPK	0	30	71.43	50	0	0	0	0
chr9:129518474-	*PTGES2**	14.29	40	85.71	57.14	0	0	0	0
	FPGS	14.29	50	85.71	67.86	0	0	0	0
chr9:132985780-	*NUP214**	14.29	40	85.71	60.71	0	10	0	3.57
	*BRD3**	0	20	71.43	53.57	0	10	0	3.57
	BAT2L1	0	40	71.43	57.14	0	10	0	3.57
	*DDX31**	0	40	71.43	53.57	0	0	0	0
chr9:137291321-	*KIAA0649**	0	20	71.43	53.57	0	10	0	3.57
	*SDCCAG3**	0	20	71.43	53.57	0	10	0	3.57
	*MAN1B1**	14.29	20	85.71	57.14	0	10	0	7.14
	*ANAPC2**	14.29	20	85.71	60.71	0	10	0	3.57
	NELFB	14.29	20	85.71	57.14	0	10	0	3.57
	*NOTCH1***	14.29	20	71.43	53.57	0	20	0	7.14
Chr14:62865875-	*HSPA2**	14.29	50	14.29	32.14	0	10	0	7.14
	*PPP2R5E**	0	50	14.29	35.71	0	10	0	7.14
Chr3:78706269-	*ROBO1***	14.29	0	0	3.57	0	0	0	0
chr3:186575268-	*SENP2**	28.57	90	42.86	67.86	0	0	0	0
chr10:16585949-	*RSU1**	0	0	0	0	0	0	0	0
chr10:26833288-	*ABI1**	0	20	0	10.71	0	0	0	0
	*YME1L1**	0	20	0	10.71	0	0	0	0
chr17:8028078-9207567	*STX8**	0	20	0	17.86	0	0	0	0

Genes showing significant correlation with gene expression in integrative analyses after correction for multiple testing (adj. p<0.05), are indicated by double asterisks (**); genes showing nominal significance (p<0.05) only, are indicated by a single asterisk (*).

Table 2. Copy number losses in PPOLS and HNSCC of genes that show correlation with expression in SCNA regions showing significant difference between PPOLS and HNSCCs.

Chromosomal coordinates	Genes ordered by strength of correlation	Single Copy Loss Frequency (%) PPOL	HNSCC			Homozygous Loss Frequency (%) PPOL	HNSCC		
			LN+	LN-	All[3]		LN+	LN-	All[3]
		N=7	N=10	N=7	N=28	N=7	N=10	N=7	N=28
chr3:57677987-58154068	*FLNB**	28.57	90	42.86	71.43	0	0	0	0
chr3:61422777-73764765	*UBA3**	14.29	100	71.43	85.71	0	0	0	0
	SUCLG2	42.86	90	85.71	85.71	0	10	0	14.29
	*ARL6IP5**	28.57	100	71.43	85.71	0	0	0	0
	ATXN7	28.57	100	85.71	85.71	0	0	0	0
	*THOC7**	28.57	100	85.71	85.71	0	0	0	0
	C3ORF64	28.57	100	71.43	85.71	0	0	0	0
	*PSMD6**	28.57	100	85.71	85.71	0	0	0	0
chr3:87035206-88461236	*CHMP2B**	14.29	80	71.43	64.29	0	10	0	7.14
	*CGGBP1**	28.57	90	71.43	67.86	0	10	0	7.14
chr3:151842842-152984767	*SIAH2**	0	0	0	0	0	0	0	0
chr3:157160169-158933894	*CCNL1***	0	0	0	0	0	0	0	0
chr3:160599782-161161335	*SCHIP1**	0	0	0	0	0	0	0	0
chr3:161705726-162566403	*KPNA4**	0	0	0	0	0	0	0	0
chr3:170691970-173911476	*MYNN**	0	0	0	0	0	0	0	0
	*TNIK**	0	0	0	0	0	0	0	0
	SLC31A1	0	0	0	3.57	0	0	0	0
	*SEC62**	0	0	0	0	0	0	0	0
	*EIF5A2**	0	0	0	0	0	0	0	0
chr3:177707736-180451354	*PIK3CA***	0	0	0	0	0	0	0	0
chr3:185035692-185529164	*AP2M1*	0	0	0	0	0	0	0	0
	PARL	0	0	0	0	0	0	0	0
	*ALG3**	0	0	0	0	0	0	0	0
	*EIF4G1***	0	0	0	0	0	0	0	0
	*DVL3***	0	0	0	0	0	0	0	0
	*ABCF3**	0	0	0	0	0	0	0	0
chr3:198578155-199298372	*RPL35A**	0	0	0	0	0	0	0	0
chr7:1631815-7317208	*EIF2AK1**	0	0	0	0	0	0	0	0
	C7ORF28A	0	0	0	0	0	0	0	0
	*RAC1***	0	0	0	0	0	0	0	0
	*CHST12**	0	0	0	0	0	0	0	0
	KDELR2	0	0	0	0	0	0	0	0
	*RNF216**	0	0	0	0	0	0	0	0
	*AIMP2**	0	0	0	0	0	0	0	0
	*NUDT1**	0	0	0	0	0	0	0	0
	WIPI2	0	0	0	0	0	0	0	0
	PMS2CL	0	0	0	0	0	0	0	0
chr9:85367221-85868737	*RMI1**	0	0	0	3.57	0	0	0	0
	*HNRNPK***	0	0	0	3.57	0	0	0	0
chr9:97596715-98774190	*CDC14B**	0	0	0	0	0	0	0	0

chr9:99427940-100373274	*ANP32B**	0	0	0	0	0	0	0	0
chr9:101504820-101852863	*ERP44***	0	0	0	3.57	0	0	0	0
chr9:123376788-125083831	*RABGAP1*	0	0	0	0	0	0	0	0
	RC3H2	0	0	0	0	0	0	0	0
chr9:126825879-127177239	*GAPVD1*	0	0	0	0	0	0	0	0
	*PPP6C**	0	0	0	0	0	0	0	0
	RABEPK	0	0	0	0	0	0	0	0
chr9:129518474-129927677	*PTGES2**	0	0	0	0	0	0	0	0
	FPGS	0	0	0	0	0	0	0	0
chr9:132985780-136636113	*NUP214**	0	0	0	0	0	0	0	0
	*BRD3**	0	0	0	0	0	0	0	0
	BAT2L1	0	0	0	0	0	0	0	0
	*DDX31**	0	0	0	0	0	0	0	0
chr9:137291321-139534231	*KIAA0649**	0	0	0	0	0	0	0	0
	*SDCCAG3**	0	0	0	0	0	0	0	0
	*MAN1B1**	0	0	0	0	0	0	0	0
	*ANAPC2**	0	0	0	0	0	0	0	0
	NELFB	0	0	0	0	0	0	0	0
	*NOTCH1***	0	0	0	0	0	0	0	0
Chr14:62865875-63862093	*HSPA2**	0	0	0	0	0	0	0	0
	*PPP2R5E**	0	0	0	0	0	0	0	0
Chr3:78706269-79206160	*ROBO1***	42.86	80	85.71	78.57	0	10	0	14.29
chr3:186575268-187080482	*SENP2**	0	0	0	0	0	0	0	0
chr10:16585949-17022250	*RSU1**	0	50	57.14	60.71	0	0	0	0
chr10:26833288-28028304	*ABI1**	0	50	57.14	60.71	0	0	0	0
	*YME1L1**	0	50	57.14	64.29	0	0	0	0
chr17:8028078-9207567	*STX8**	28.57	10	42.86	21.43	0	0	0	0

Genes showing significant correlation with gene expression in integrative analyses after correction for multiple testing (adj. $p<0.05$), are indicated by double asterisks (**); genes showing nominal significance ($p<0.05$) only, are indicated by a single asterisk (*).

Further Information: Correlating gene expression to somatic copy number alterations

To identify potential drivers of somatic copy number alterations we considered the correlation between gene expression and copy number for each gene across all samples. This analysis is based on the hypothesis that a consistent phenotypic effect should be observed on the gene expression of CNA driver genes, while passenger genes might remain unaffected.

To do so the gene expression data provided by (Hunter et al., 2006) was used and integrated to our copy number data resulting in a dataset of 29 samples with both gene expression and copy number values (18 PPOL and 11 HNSCC cell lines). We used PANP (Warren P., Bioconductor) to reduce the number of genes considered for further analysis to 8255 unique genes expressed in at least one sample.

Before correlating the SCNA and gene expression values corrections were applied for polyploidy and heterogeneity.

When considered a by-product of instability rather than a response of biological significance, the copy number (CN) values were altered to remove the ubiquitous chromosomal amplification observed in polyploid samples. To do so, all the CN values inferred for SNPs in chromosome arms with mean CN larger than 2.5 were reduced by one unit. The reduction was however rejected in the cases of heterozygous copy neutral calls (CN2 LOH0), copy losses (CN1) and homozygous deletions (CN0). These states were not altered.

Due to heterogeneity, the gene expression values obtained did not represent the expression of the CN-altered cells solely. For each genomic region, a non-negligible proportion of cells do not harbour any alteration. The proportion of cells with normal heterozygous copy number in each region was estimated using OncoSNP (Yau C., et al., 2010). When investigating the correlation between SCNAs and mRNA expression the weighted mean CN and LOH values of this mixture were used.

The resulting correlation coefficients across genes displayed a tendency for positive correlation of expression with copy number and negative correlation with LOH (i.e. higher gene expression in amplified regions and lower expression in LOH regions). Furthermore an analysis of auto-correlation of the correlation coefficients along the genome revealed significant level of auto-correlation across the positively correlated genes in the CN analysis, but no auto-correlation in the negatively correlated genes. The reverse was observed in the LOH analysis. Thus only genes displaying higher expression when amplified or lower expression when undergoing loss of heterozygosity were further considered. The significance of the correlation of the filtered list were corrected for multiple testing using a Sidak procedure resulting in 484 CN vs GE and 130 LOH vs GE genes (51 genes overlapping) with p-value below 0.05.

Identifying regions of differential CN and LOH between PPOLS an HNSCC

In order to find regions of copy number alterations typical of PPOLS or HNSCC samples, we examine the common genetic pattern of each group and searched for significant differences.

In the case of differential CN events we considered the contingency table displaying the number of samples by group (i.e. PPOL/HNSCC) and amplification status (i.e. amplification/neutral/deletion) and performing a Chi-squared test for each unique genomic region. The same was performed to identify differential LOHs by considering the LOH status instead of the amplification status. This yielded 208 significant CN regions and 192 significant LOH regions ($p < 0.05$).

Integrating differentially altered regions with significant CN vs GE genes to identify candidate driver genes

The genes with expression identified as responding significantly to underlying alterations, both CNA and LOH, are suggested as potential driver genes of the regions with distinct patterns of alteration between PPOL and HNSCC.

The overlap of those two lists identified 23 CN regions with 61 potential driver genes and 6 LOH regions with 6 potential driver genes. Those are proposed as candidate differentiators of carcinoma versus dysplasia.

The genes underlying these regions are presented in Tables 1-2 showing copy number gains (Table 1) and losses (Table 2) these loci in PPOLS and HNSCC overall as well as in LN^{+ve} and LN^{-ve} HNSCC where these data were available.

References

Hunter KD1, Thurlow JK, Fleming J, Drake PJ, Vass JK, Kalna G, Higham DJ, Herzyk P, Macdonald DG, Parkinson EK, Harrison PR. Divergent routes to oral cancer. Cancer Res. 2006 Aug 1;66(15):7405-13.

Warren P. panp: Presence-Absence Calls from Negative Strand Matching Probesets. (Bioconductor; https://bioconductor.riken.jp/packages/3.2/bioc/html/panp.html).

Christopher Yau, Dmitri Mouradov, Robert N Jorissen, Stefano Colella, Ghazala Mirza, Graham Steers, Adrian Harris, Jiannis Ragoussis, Oliver Sieber, Christopher C Holmes
A statistical approach for detecting genomic aberrations in heterogeneous tumor samples from single nucleotide polymorphism genotyping data. Genome Biol. 2010; 11(9): R92. Published online 2010 Sep 21. doi: 10.1186/gb-2010-11-9-r92

SUPPLEMENTARY DATA 17 - ANALYSIS OF CLDN1 AND BCL2L1 EXPRESSION IN PRIMARY HNSCC AND PPOL

Figure 1. Expression of CLDN1 in PPOLS and HNSC

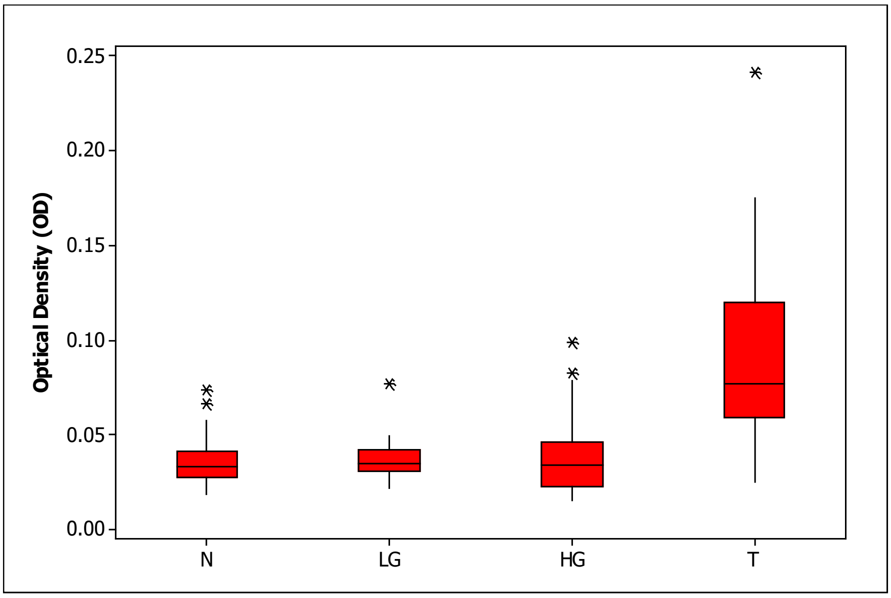

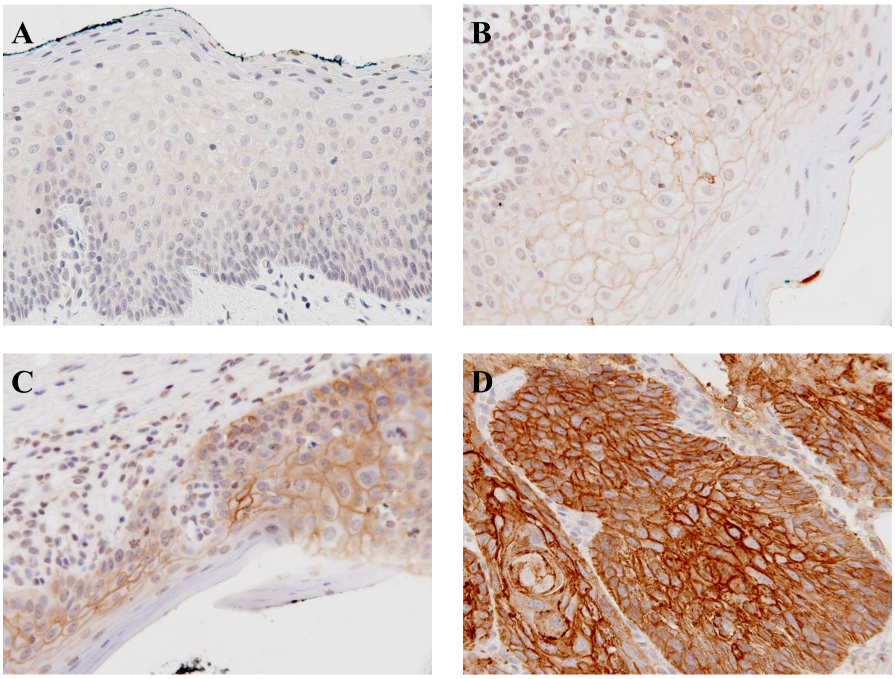

Top Panel: Box plots of optical density (OD) readings of CLDN1 immunostaining of normal oral epithelium (N), Low grade PPOL (LG), High grade PPOL(HG), and HNSCC (T). Within each box the top line represents the third quartile, the middle line represents the median and the bottom line represents the first quartile. Median OD scores for each tissue type were as follows; N = 0.034, LG = 0.035, HG = 0.035, T = 0.077. HNSCC showed significantly higher expression compared to other groups ($p<0.00001$, Mann Whitney U Test).

Bottom Panel: Representative images of CLDN1 immunostaining in normal oral epithelium (A), Low grade PPOL (B), High grade PPOL(C), and HNSCC (D).

Figure 2. Expression of BCL-XL in PPOLS and HNSCC

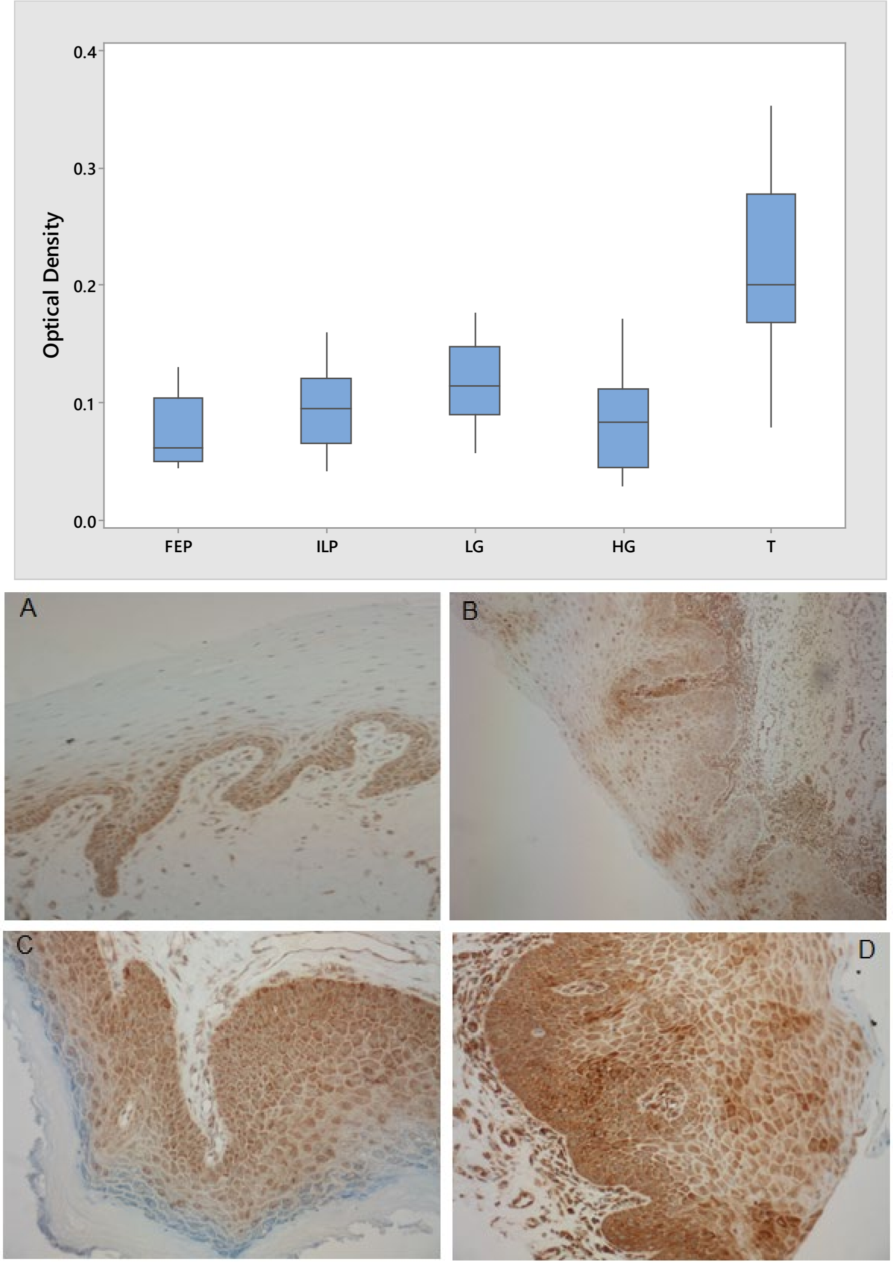

Top Panel. Box plots of optical density (OD) readings of Bcl-XL IHC staining in fibroepithelial polyps (FEP), lichen planus (ILP), low grade PPOL (LG), high grade PPOL (HG) and HNSCC (T). Parametric one-way ANOVA testing showed significant differences between the groups ($p<0.0001$); post hoc T test confirmed significant differences ($p<0.0001$) between immunostaining density in HNSCC compared to all other groups. There were no differences between the other groups.

Bottom Panel. Representative images of BCL-XL immunostaining in normal oral epithelium (A), Low grade PPOL (B), High grade PPOL(C), and HNSCC (D).

Further information:

Analysis of CLDN1 Expression
Materials and Methods
HNSCC and PPOLs diagnosed between 2008 and 2010 were retrieved from the Central Manchester Foundation Trust (CMFT) pathology database. All cases reviewed by a consultant pathologist to confirm the diagnosis. PPOLs were categorised into high grade (HG) including moderate and high dysplasia and low grade (LG) including mild dysplasia, using the information from the patient report and an additional review of the histology by a consultant pathologist. All samples were coded and anonymised.

Sample panel consisted of 42 HNSCC, 19 HG PPOLs biopsies and 24 LG PPOLs. Normal epithelium adjacent to HNSCC within 39 of the 42 tumour blocks was also analysed. Clinical parameters from all of the HNSCC cases were retrieved from each case report. These included the patient sex and age along with the tumour differentiation, stage, lymph node invasion, lympho-vascular invasion, and peri-neural invasion status.

Standard protocols for immunohistochemistry were followed with an optimised, rabbit polyclonal IgG antibody to Claudin 1, (Abcam, Cambridge, UK) at a dilution of 1:500. IHC staining was performed on a Ventana Benchmark XT Automated Immunostaining Module (Ventana Medical Systems, Tucson, AZ). All other reagents used were also supplied from Ventana Medical Systems. Optimisation of Claudin 1 also involved the used of Protease 1 (Ventana, 760-2018). Positive (normal human colon) and negative (minus the primary antibody) controls were processed with each batch.

Intensity and area of immunostaining of Claudin 1 was measured using a semi-automated image analysis method. This incorporated a standard light microscope attached to a CRI Nuance spectral analyzer (CRI, Woburn, MA), which was supported by Nuance Alpha, 1.6.2.368 software. This system used spectral unmixing of the IHC stained sections to identify and measure the areas of DAB positive staining. This method of IHC quantification has been as validated in recent publications (Das, et al. 2004, Taylor and Levenson 2006). Five random, conventional bright-field image cubes of the epithelium in each IHC stained section were collected at 10nm wavelength intervals from 480 nm (blue) to 700 nm (red) at a 40x magnification. In addition 38 of the 42 HNSCC tissue slides also had five random image cubes taken of its adjacent normal epithelium.

The DAB spectral profile of each image cube was unmixed from the haematoxylin spectral profile in each cube using Nuance 2.6.0 software (CRI, Woburn, MA), creating a composite false colour image cube. This enabled the intensity of the DAB staining per pixel of each positively stained site within the image cube to be measured as an average optical density (OD). The average of the ODs recorded in all five images cubes taken per slide was calculated to produce an overall OD score for that particular tissue sample. Further description of this methodology can be found in Byers et al 2008.

Statistical analysis was performed using the statistical software packages Minitab 16 2010 (Minitab Inc, USA) and Medcalc version 12.3.0 (Medcalc Software, Belgium). For all of the statistical tests a p value of < 0.05 was considered to be statistically significant. Basic statistical analysis and Anderson-Darling tests

were performed to test the data's normality in Minitab. The data sets were not normally distributed and so non-parametric statistical analysis was performed. The significance of differences in Claudin 1 immunostaining optical density between the different sample groups was tested for significance using the Kruskal Wallis test for non-parametric data and post-hoc analysis with Mann-Whitney U test. Spearman's rank correlation coefficient for non-parametric data was used to test for significance of the correlation between Claudin 1 immunostaining and progression towards HNSCC and clinical parameters.

Analysis of BCL2L1 Expression in Primary HNSCC and PPO
Materials and methods
HNSCC and PPOLs diagnosed between 2012 and 2014 were retrieved from the Central Manchester Foundation Trust (CMFT) pathology database. All samples were coded and anonymised. Sample panel consisted of 36 HNSCC, 29 HG PPOLs biopsies, 27 LG PPOLs, 10 ILP and 10 FEP. Clinical parameters from all of the HNSCC cases were retrieved from each case report. These included the patient sex and age along with the tumour differentiation, stage, lymph node invasion, lympho-vascular invasion, and peri-neural invasion status.
All IHC staining was performed on the Ventana Benchmark XT automated IHC staining module with reagents also supplied by Ventana Medical Systems. Heat mediated antigen retrieval was performed for 36 minutes. Bcl-XL antibody was prepared at a 1/400 dilution and incubated at room temperature for 36 minutes.

Bcl-XL IHC Staining intensity was scored using the CRI Nuance Multi-spectral Imaging System. The DAB and haematoxylin spectral profiles were identified and unmixed in order to accurately measure the intensity of the DAB substrate reaction of bound primary antibody to Bcl-XL only. Five random, bright-field image cubes in each stained section were collected at 10nm wavelength intervals from 500 nm to 720 nm at a 40x magnification. For each cube area, the intensity of the DAB positive staining per pixel was measured as an average optical density (OD) value. The average OD recorded in all five image cubes taken per slide were calculated to produce an overall OD score for that particular tissue block.

Statistical analysis was performed using the statistical software package Minitab version 17.2.1 (Minitab Inc, USA). For all of the statistical tests a p value of < 0.05 was considered to be statistically significant, allowing the null hypothesis to be rejected and the alternative hypothesis to be accepted.

SUPPLEMENTARY DATA S18– KEGG PATHWAY GENE ENRICHMENT IN GISTIC REGIONS

PATHWAY	IMMORTAL PPOLS	LN-VE HNSCC	LN+VE HNSCC	ALL HNSCC	ALL GISTIC REGION GENES CORRELATING WITH EXPRESSION IN SAMPLES WHERE DATA AVAILABLE
Pathways in cancer	2.46E-06	2.90E-05	1.13E-12	1.97E-12	5.01E-17
Endocytosis	1.61E-07	0.0011	1.54E-12	2.89E-09	9.12E-14
Small cell lung cancer	X	7.52E-06	5.11E-05	6.00E-07	2.52E-07
Ubuiquitin mediated proteolysis	X	X	0.0001	1.52E-06	1.41E-06
Focal adhesion	0.0002	0.0006	2.18E-10	1.14E-05	7.22E-11
Wnt signaling pathway	0.0007	X	1.47E-11	1.14E-05	1.02E-07
Apoptosis	X	X	0.001	2.96E-05	0.0005
Regulation of actin cytoskeleton	7.62E-05	X	2.20E-08	3.71E-05	1.02E-09
Colorectal cancer	2.33E-05	X	0.0002	4.84E-05	2.83E-05
Pancreatic cancer	X	X	0.006	5.31E-05	1.21E-07
MAPK signaling pathway	5.34E-09	X	1.63E-12	0.0001	3.38E-09
Phagosome	X	X	3.67E-13	0.0002	X
Cell adhesion molecules (CAMs)	X	X	2.79E-11	0.0002	X
mTOR signalling pathway	X	X	X	0.0003	0.0002
Melanoma	0.0004	X	0.0005	0.0005	0.0055
Non-small cell lung cancer	0.0004	X	X	0.0005	8.92E-06
Axon guidance	0.0002	X	8.40E-08	0.0013	6.80E-09
p53 signaling pathway	2.00E-04	X	4.73E-05	0.0013	X
Cell cycle	X	0.0046	8.46E-06	0.002	3.38E-09
Endometrial cancer	0.0004	X	X	0.0038	X
Glioma	X	0.0074	0.0024	0.0066	4.16E-05
TGF-beta signaling pathway	0.0007	X	1.52E-05	0.0075	X
Bladder cancer	X	X	0.0052	0.0075	X
Erb signalling pathway	0.0002	X	X	0.0098	2.73E-10
Toll-like receptor signaling pathway	8.45E-05	X	1.11E-07	X	X
Prostate cancer	0.0003	X	0.0013	X	3.72E-05
PPAR signaling pathway	0.0007	X	0.006	X	X
Notch signaling pathway	X	0.0066	0.0003	X	0.0021
Jak-STAT signaling pathway	X	X	5.76E-14	X	0.0019
Regulation of autophagy	X	X	3.75E-07	X	X
Mismatch repair	X	X	0.0083	X	0.0003

SUPPLEMENTARY DATA S19 - SCNA OF GENES IN CANCER REALTED KEGG PATHWAYS ENRICHED IN GISTIC REGIONS

The following bar charts illustrate SCNAs of cancer-relevant pathway genes that are significantly enriched in the GISTIC regions in PPOLs and HNSCC. For each pathway, two charts are shown illustrating frequency of copy number gains (top panel) and losses (bottom panel) in PPOLs, all HNSCC, and HNSCCs with and without nodal metastases (LN+ve and LN-ve respectively). Genes showing significant correlation with expression in integrative analyses after correction for multiple testing (adj. $p<0.05$), are indicated by double asterisks (**); genes showing nominal significance ($p<0.05$) only are indicated by a single asterisk (*). Only genes showing at least 40% frequency of SCNA in at least one subgroup are shown

Figure 1. Pathways in Cancer

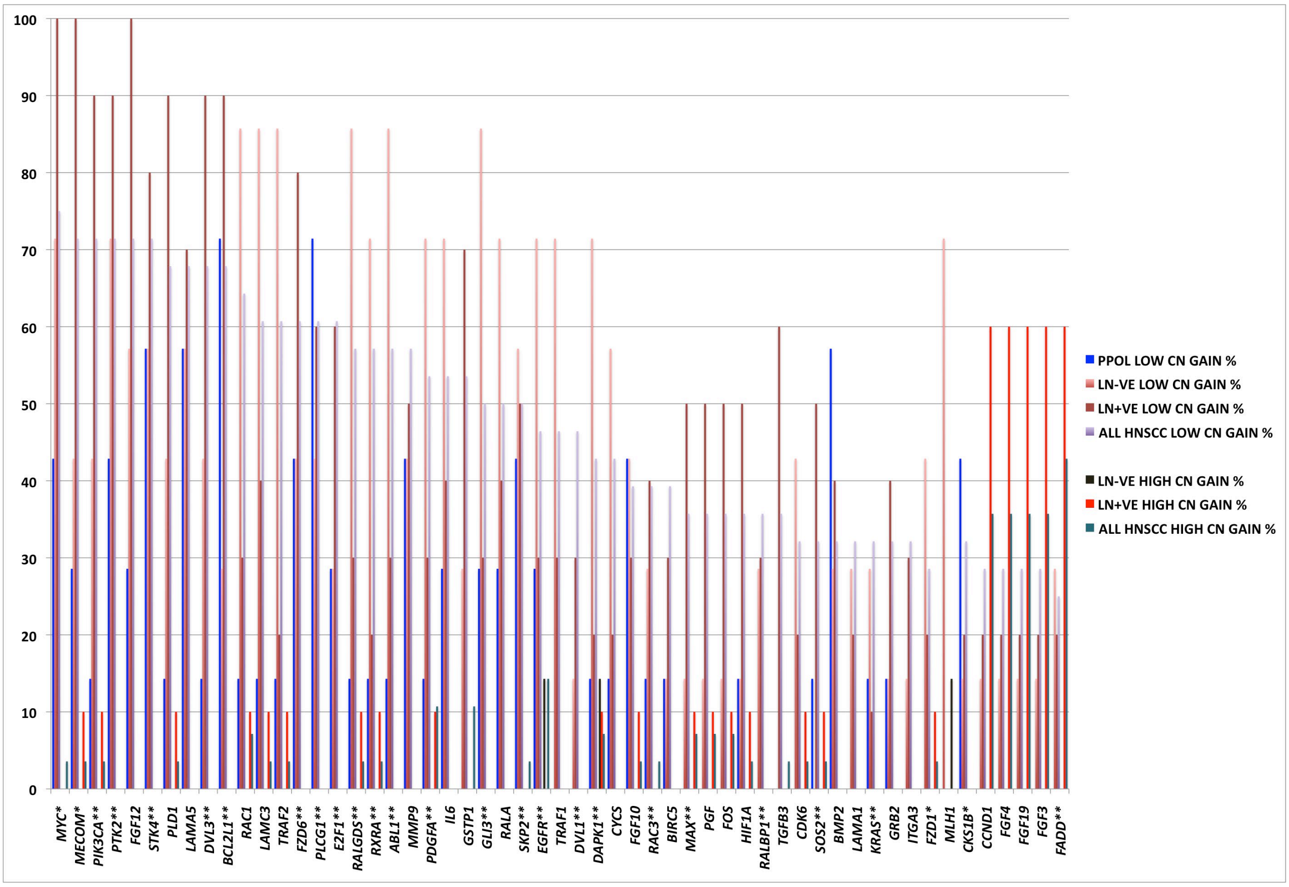

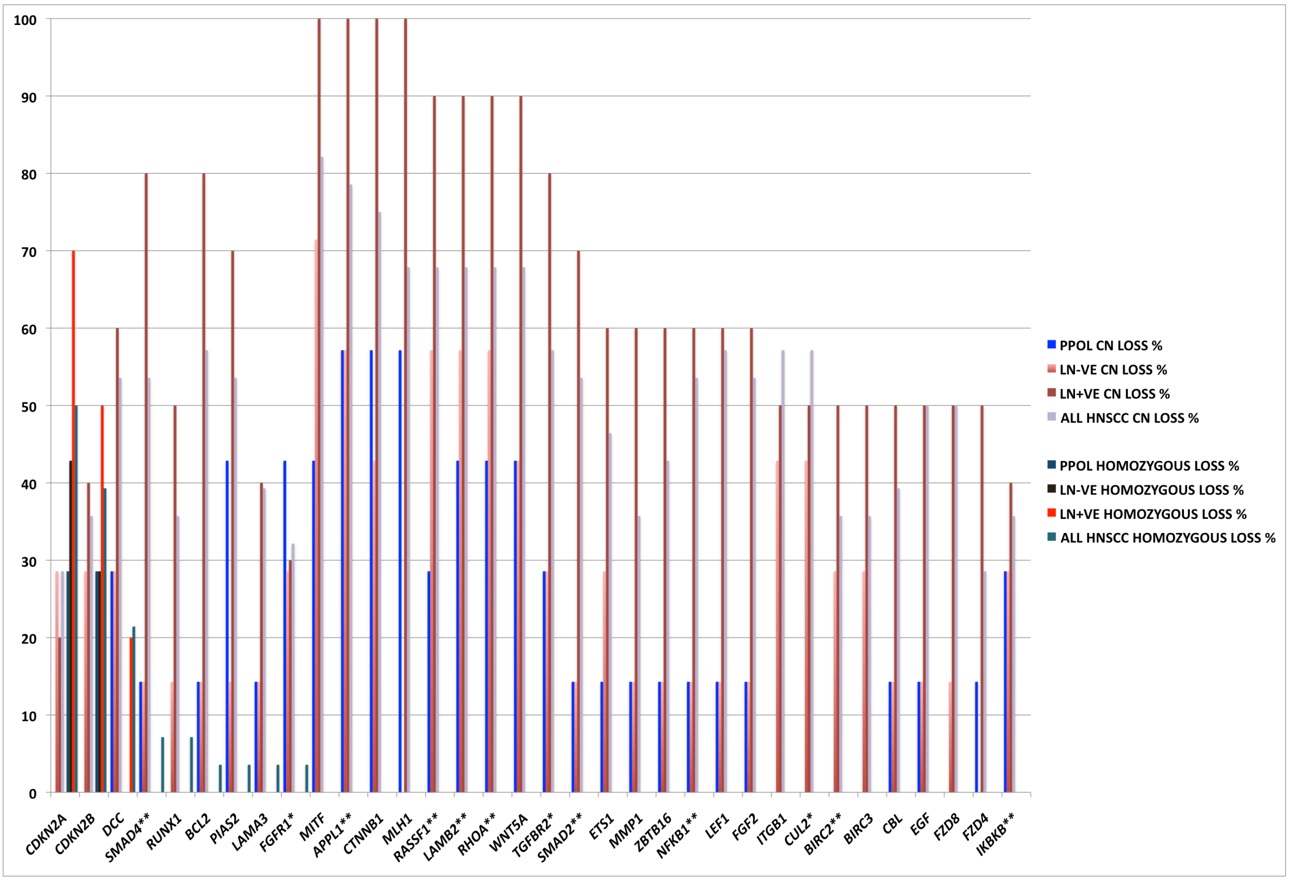

Figure 2. Apoptosis Pathway

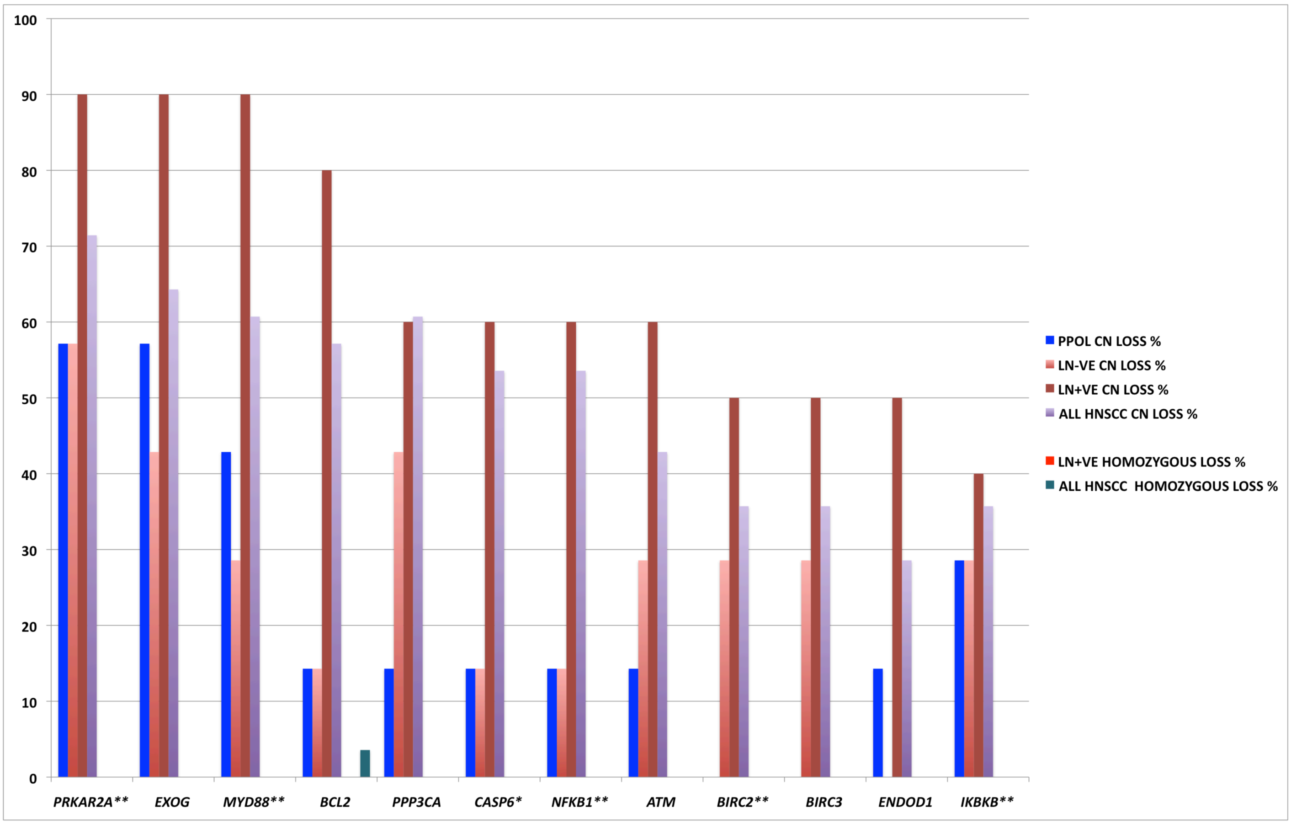

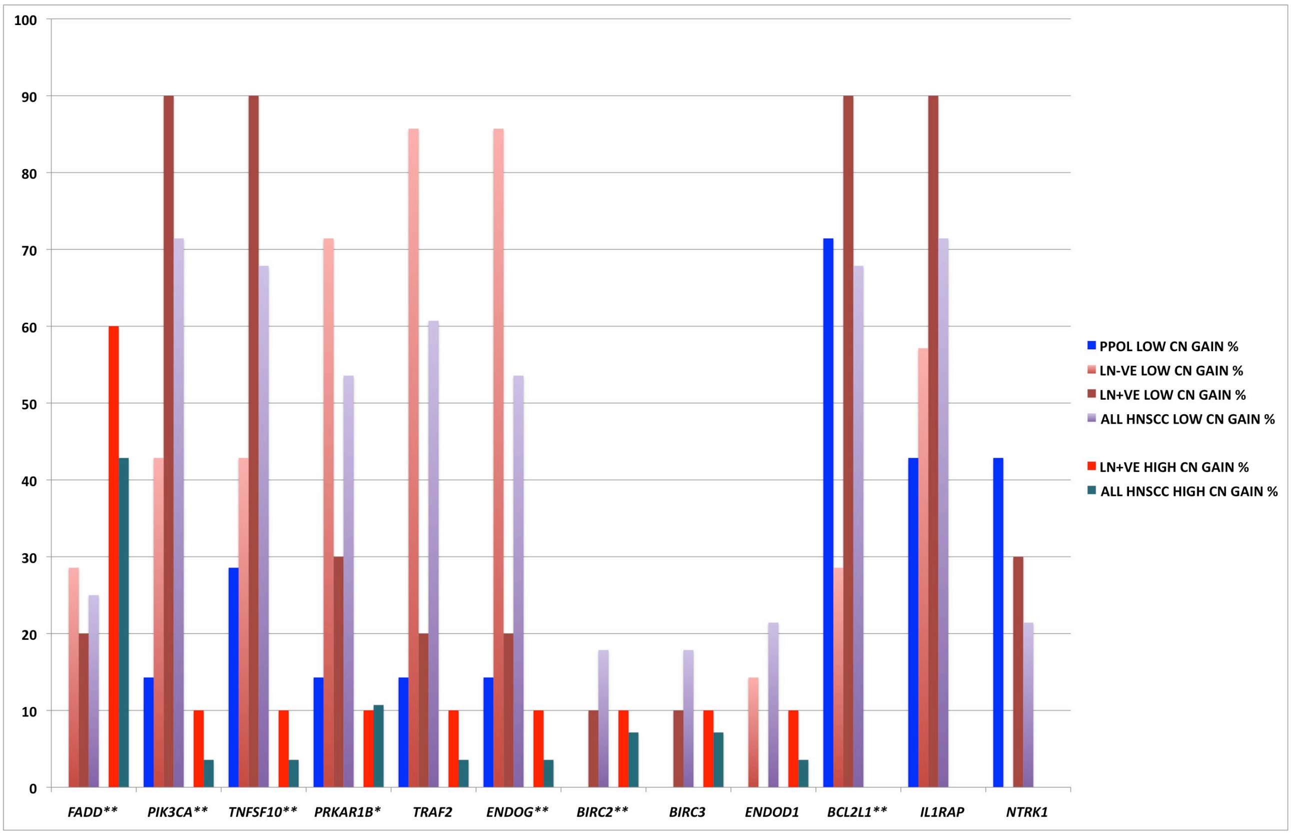

Figure 3. Axon Guidance Pathway

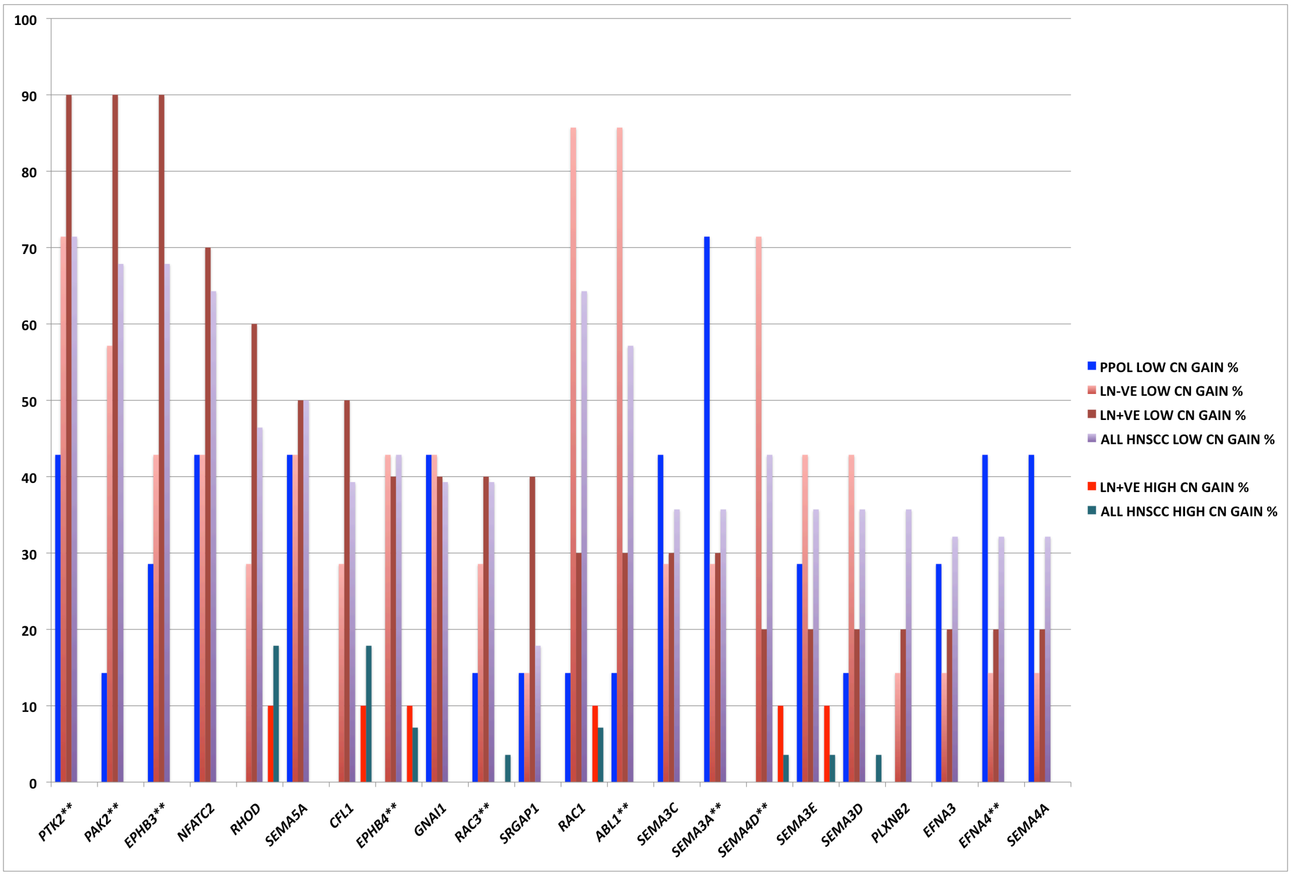

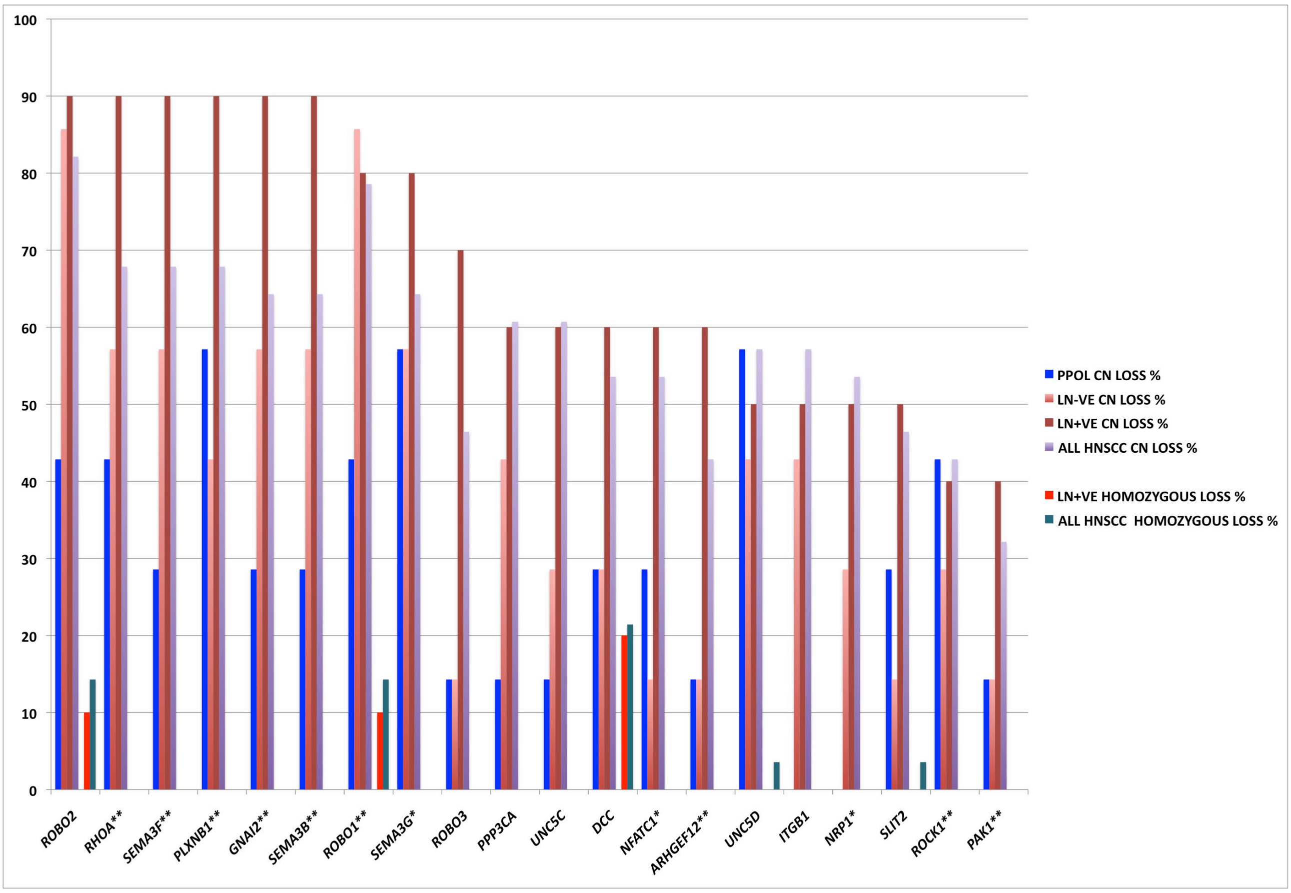

Figure 4. Cell Adhesion Pathway

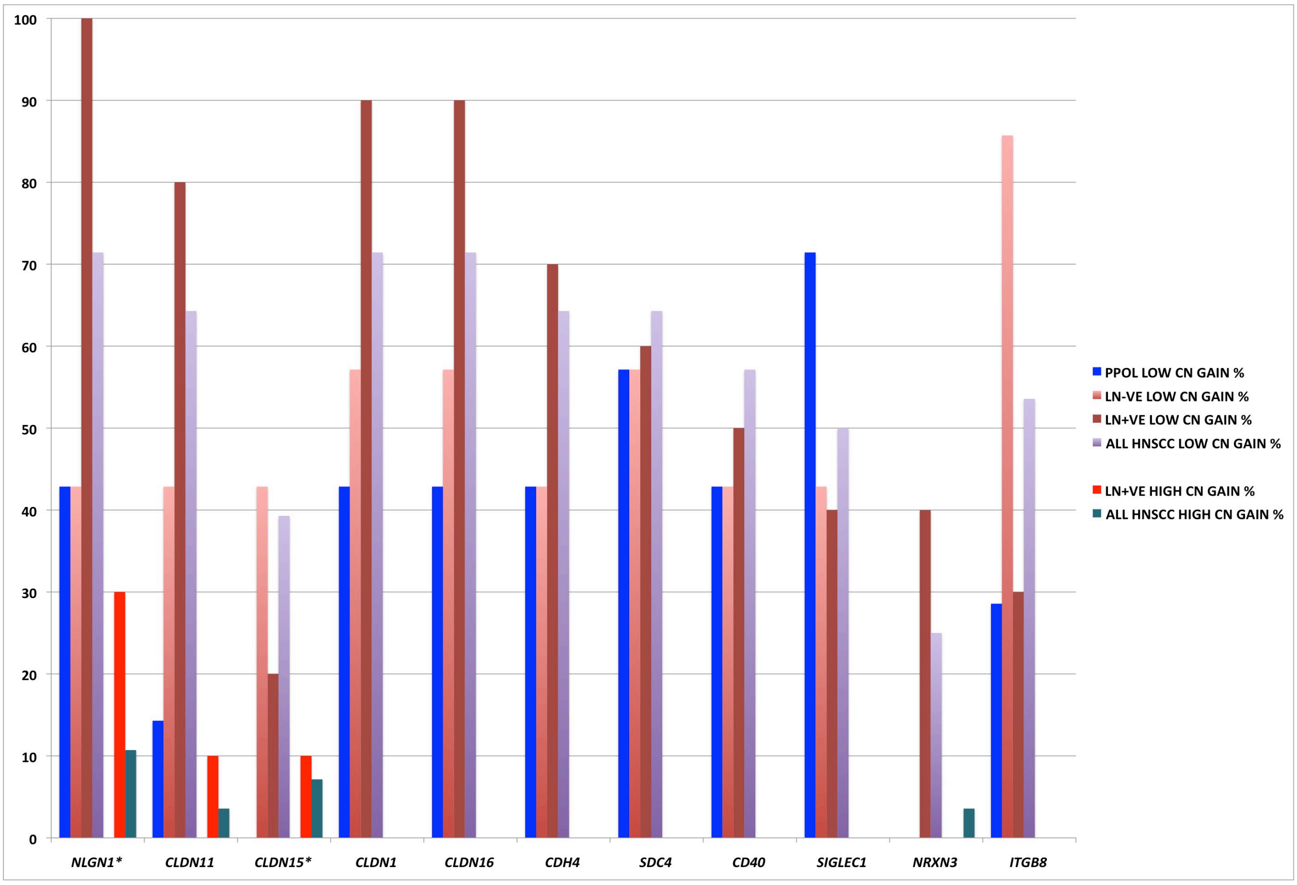

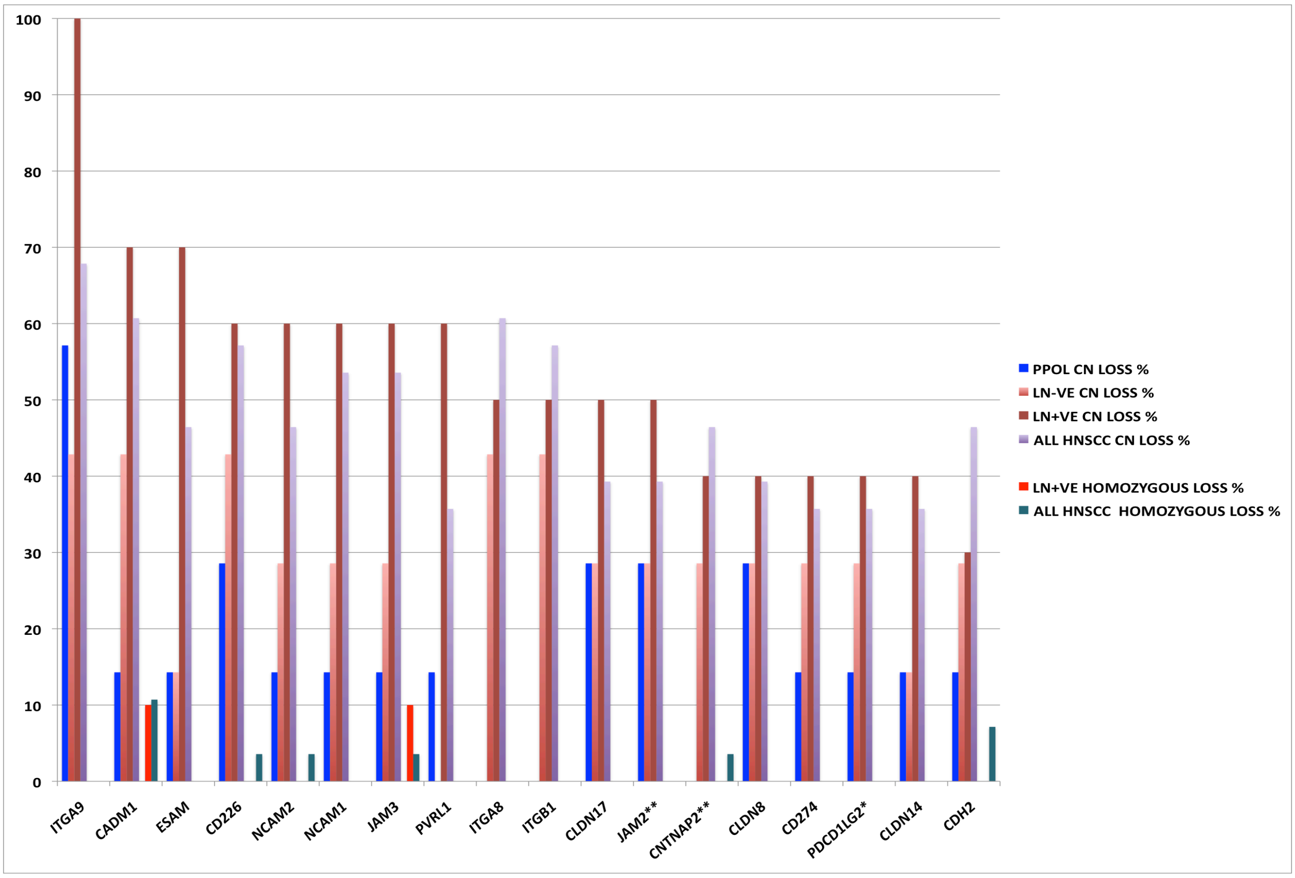

Figure 5. Cell Cycle Pathway

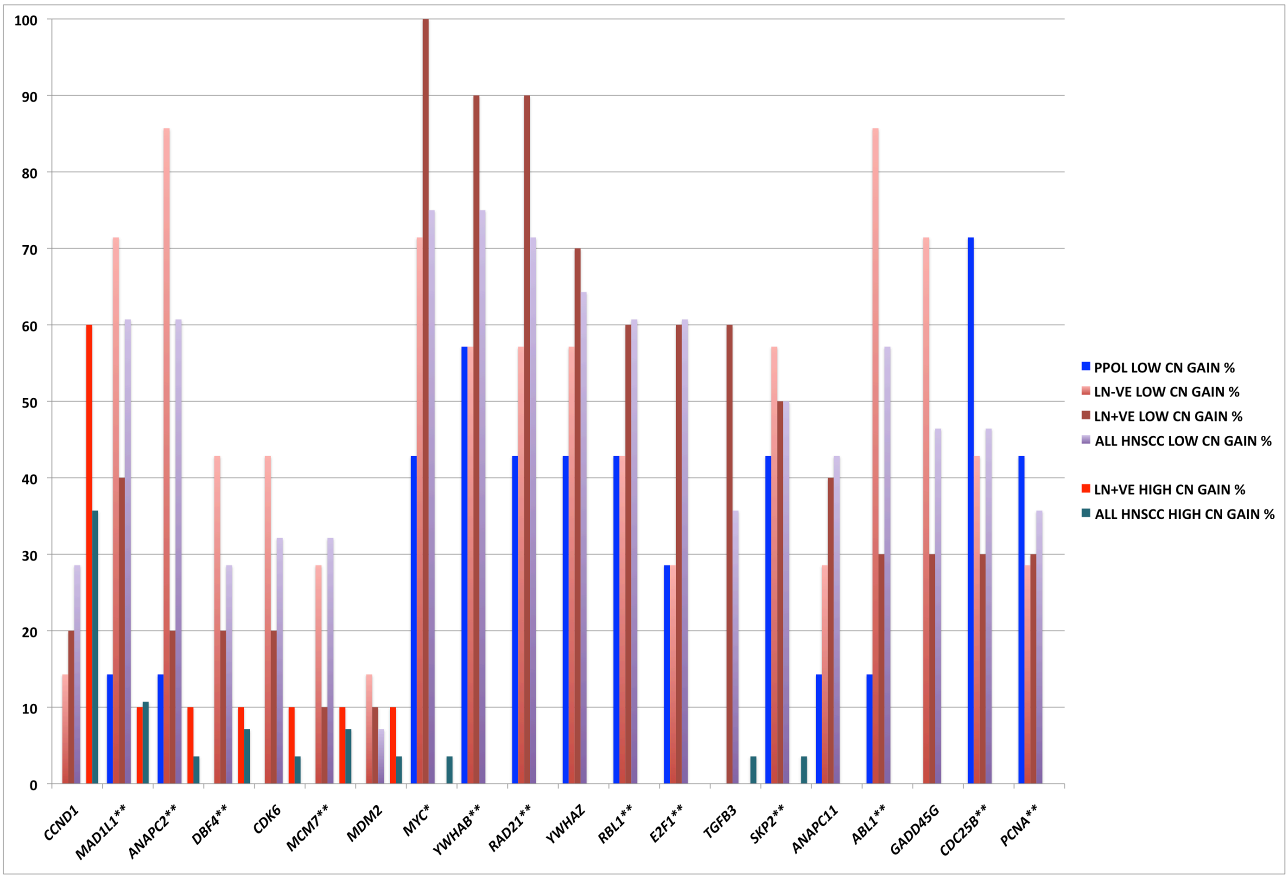

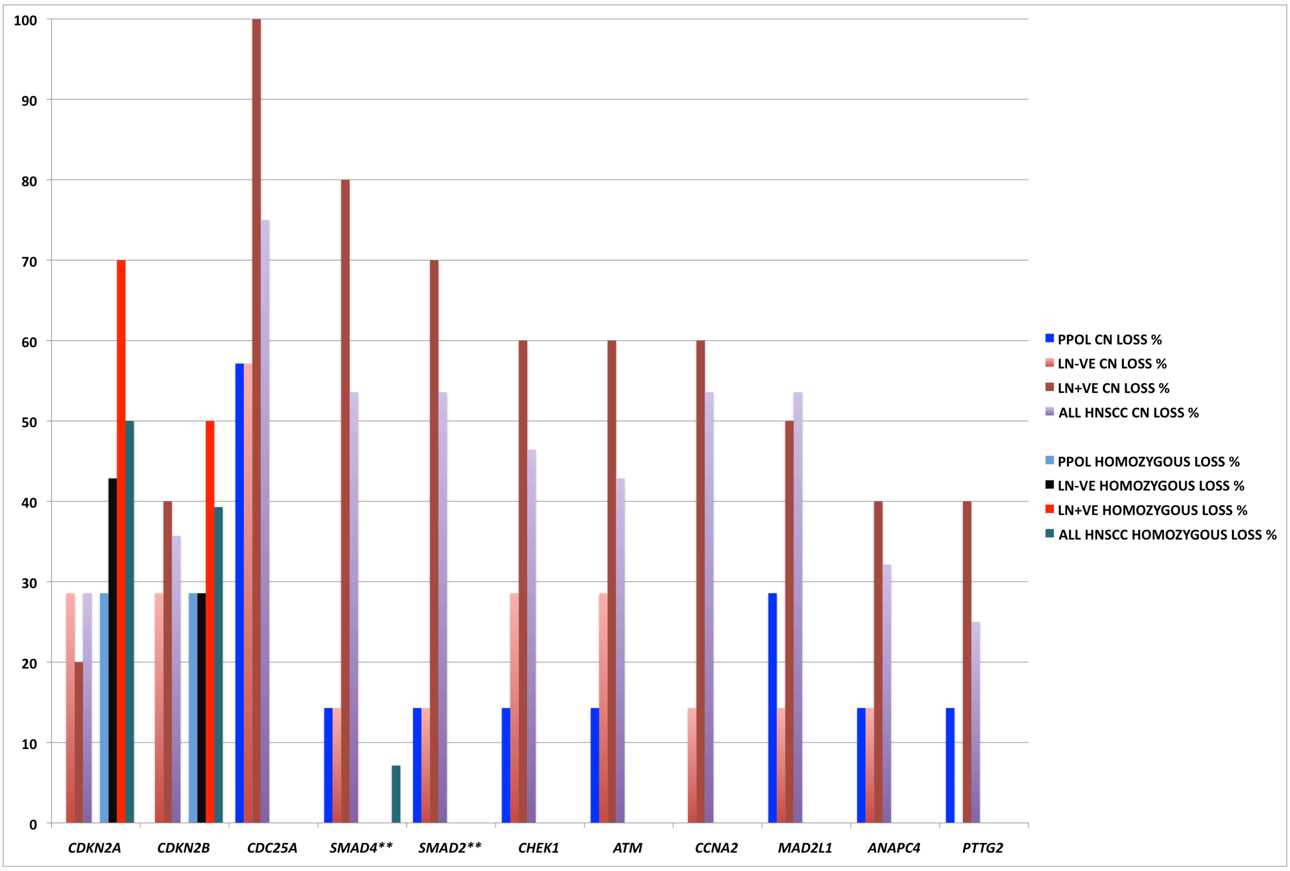

Figure 6. Endocytosis Pathway

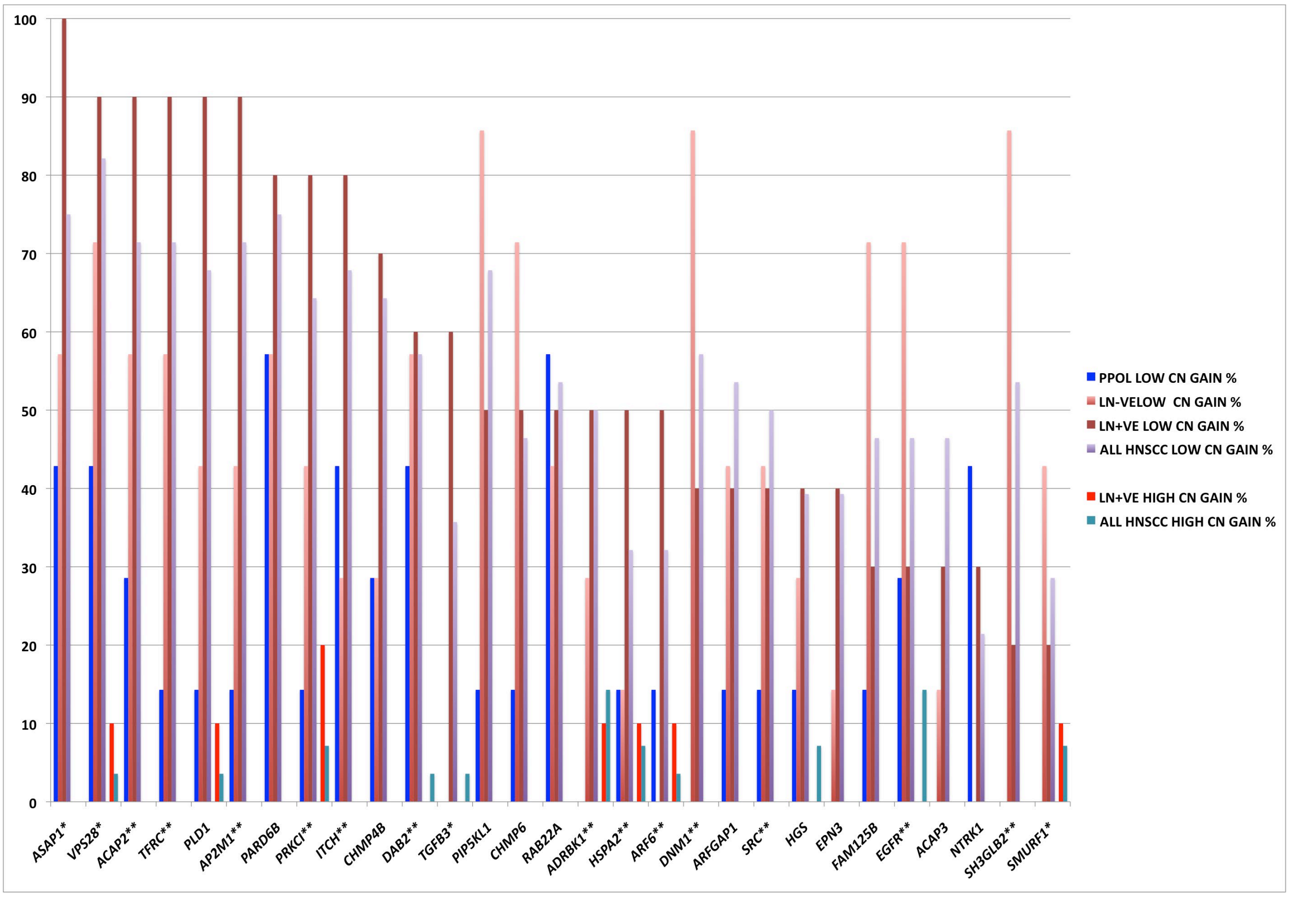

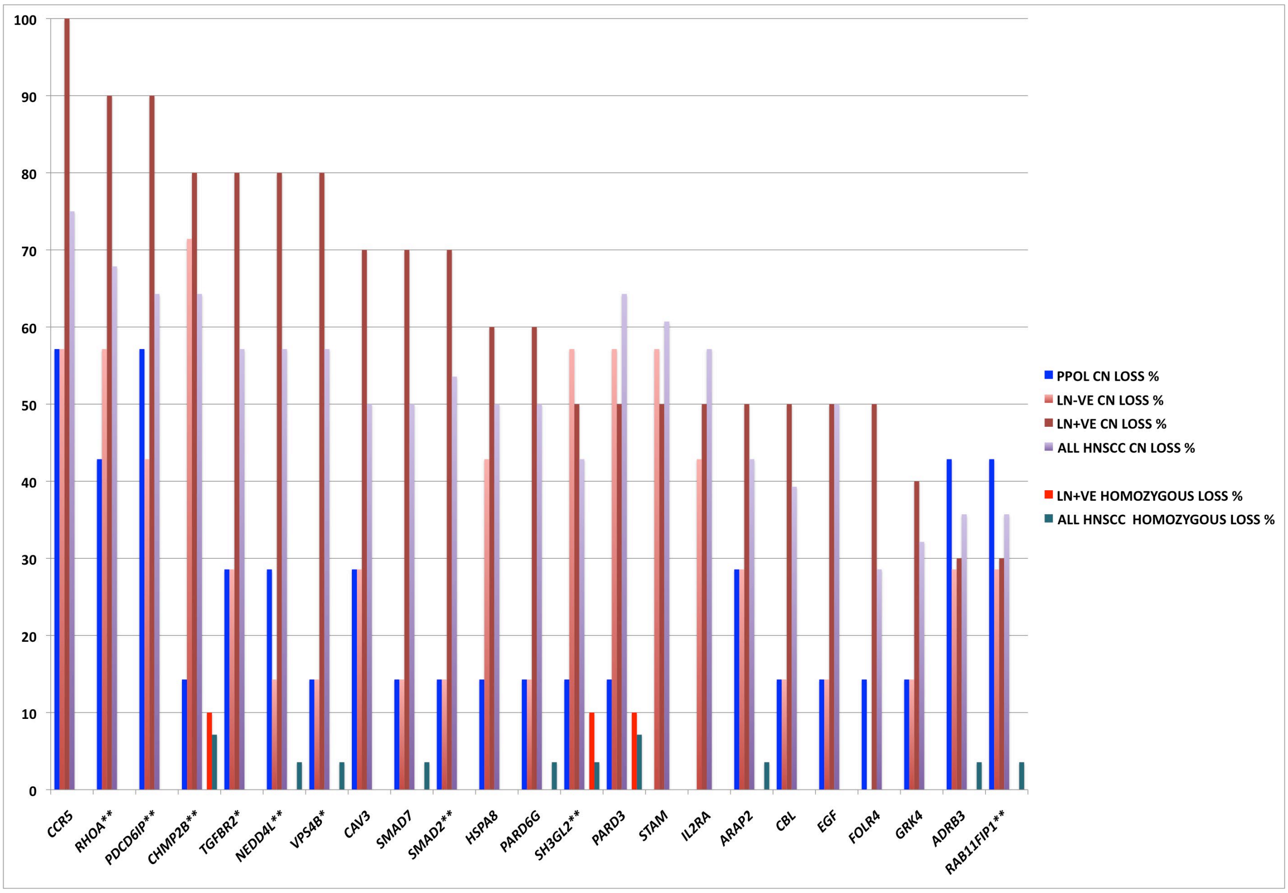

Figure 7. Jak-STAT Pathway

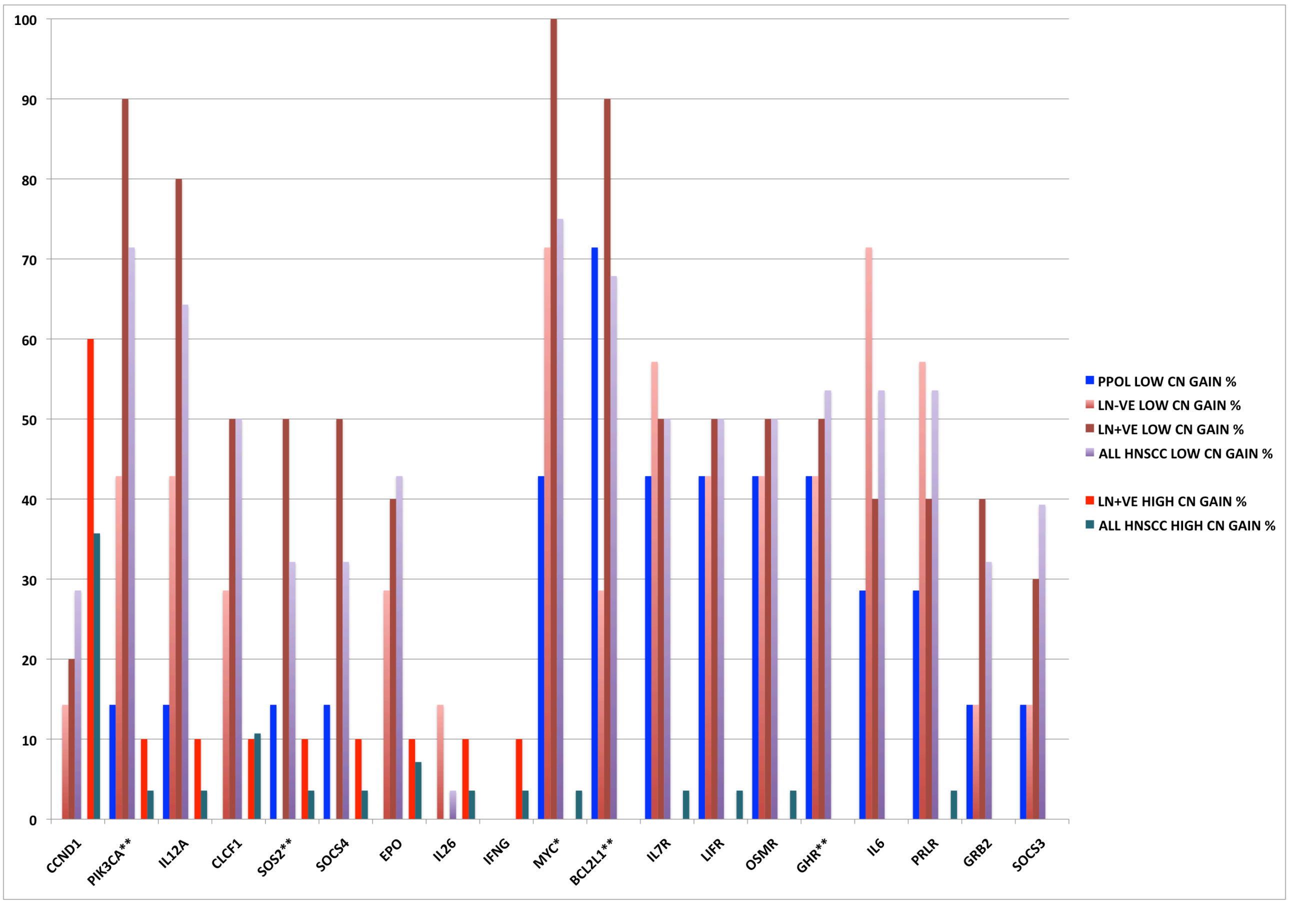

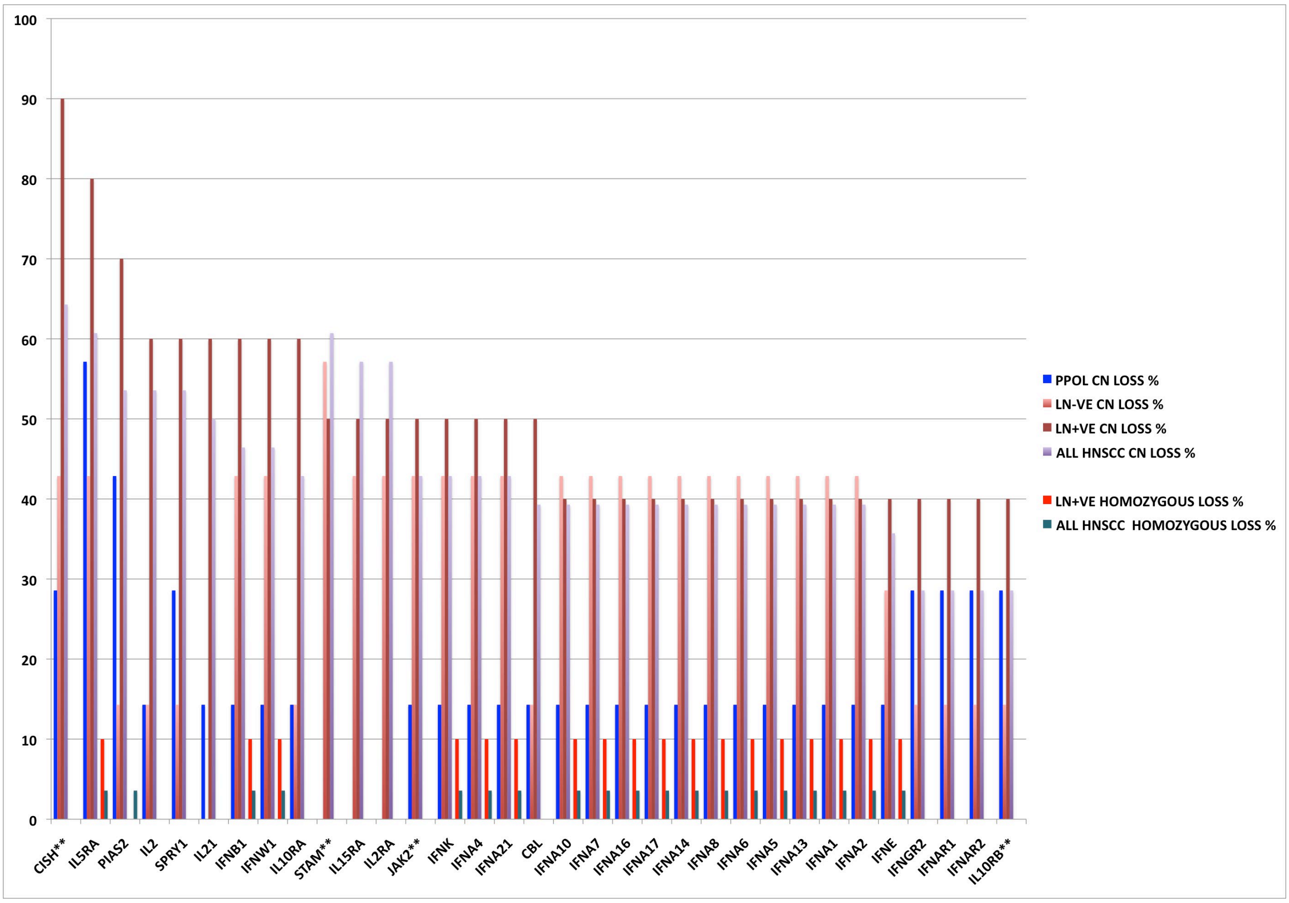

Figure 8. MAPK Pathway

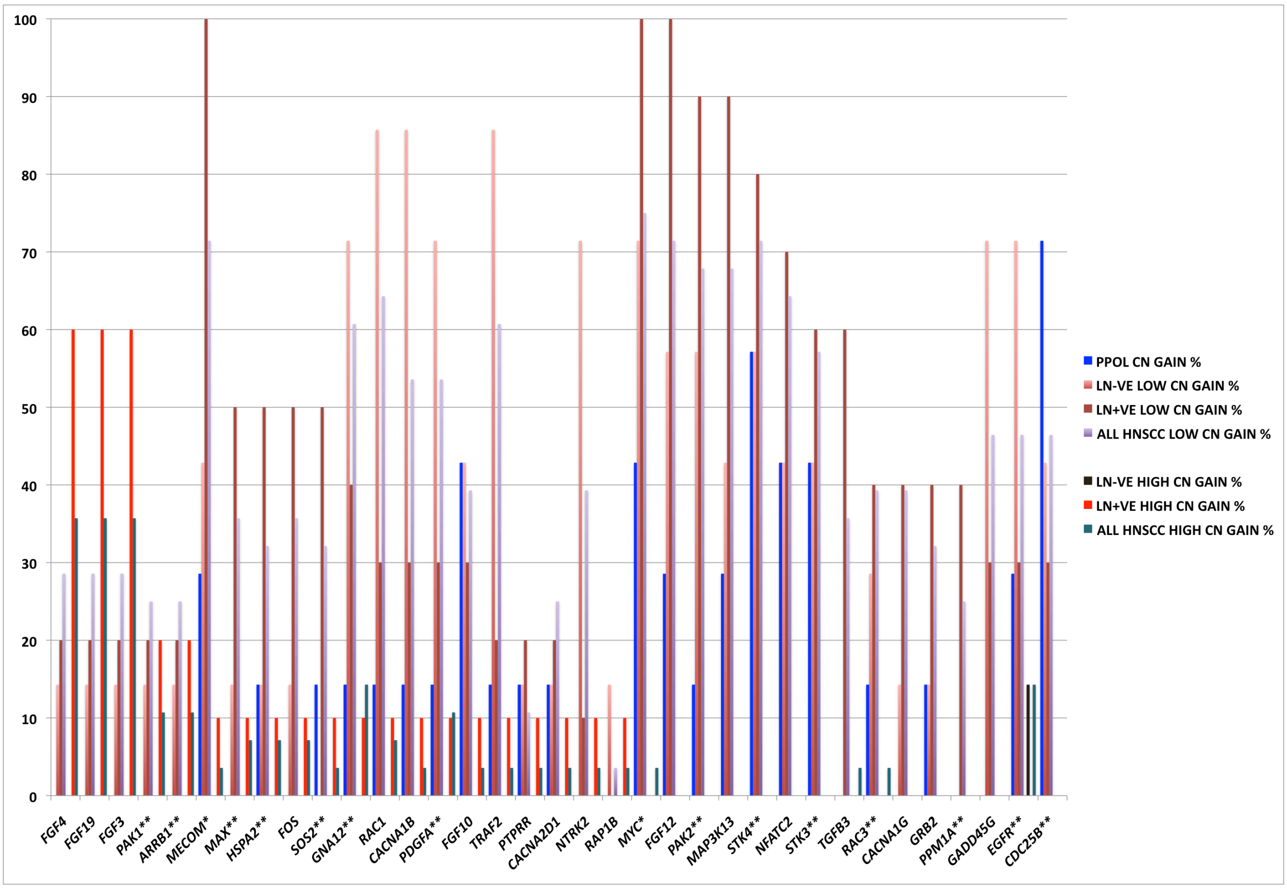

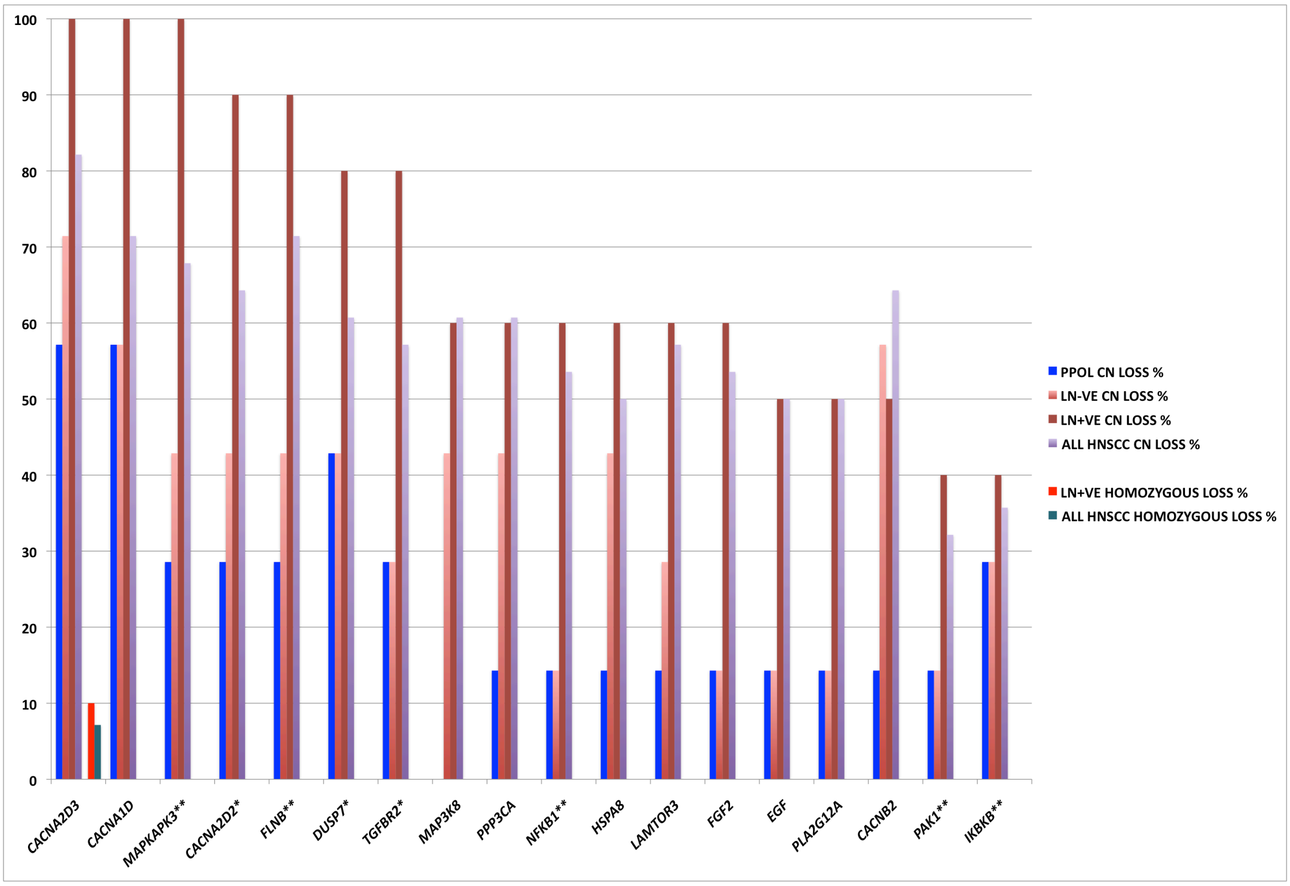

Figure 9. Ubuiquitin-Proteosome Pathway

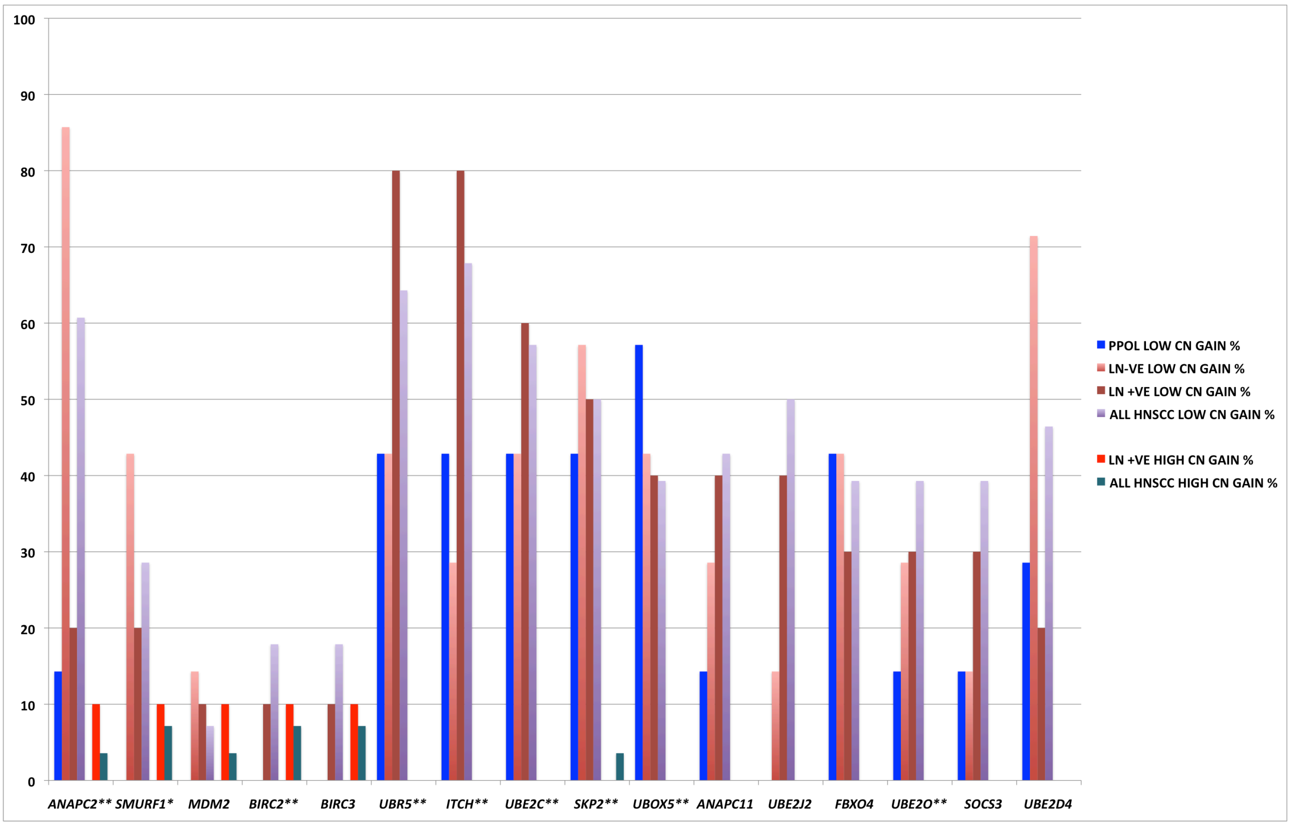

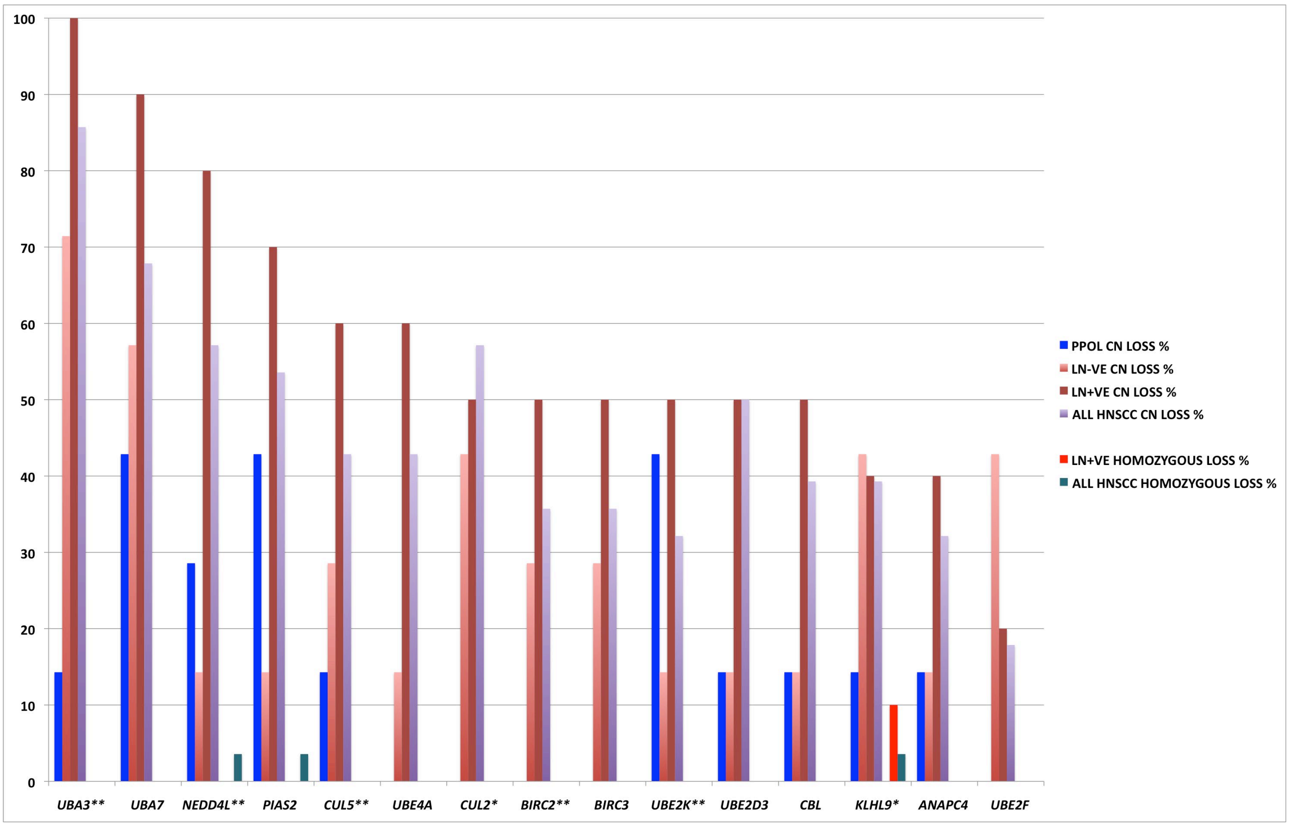

Figure 10. WNT Pathway

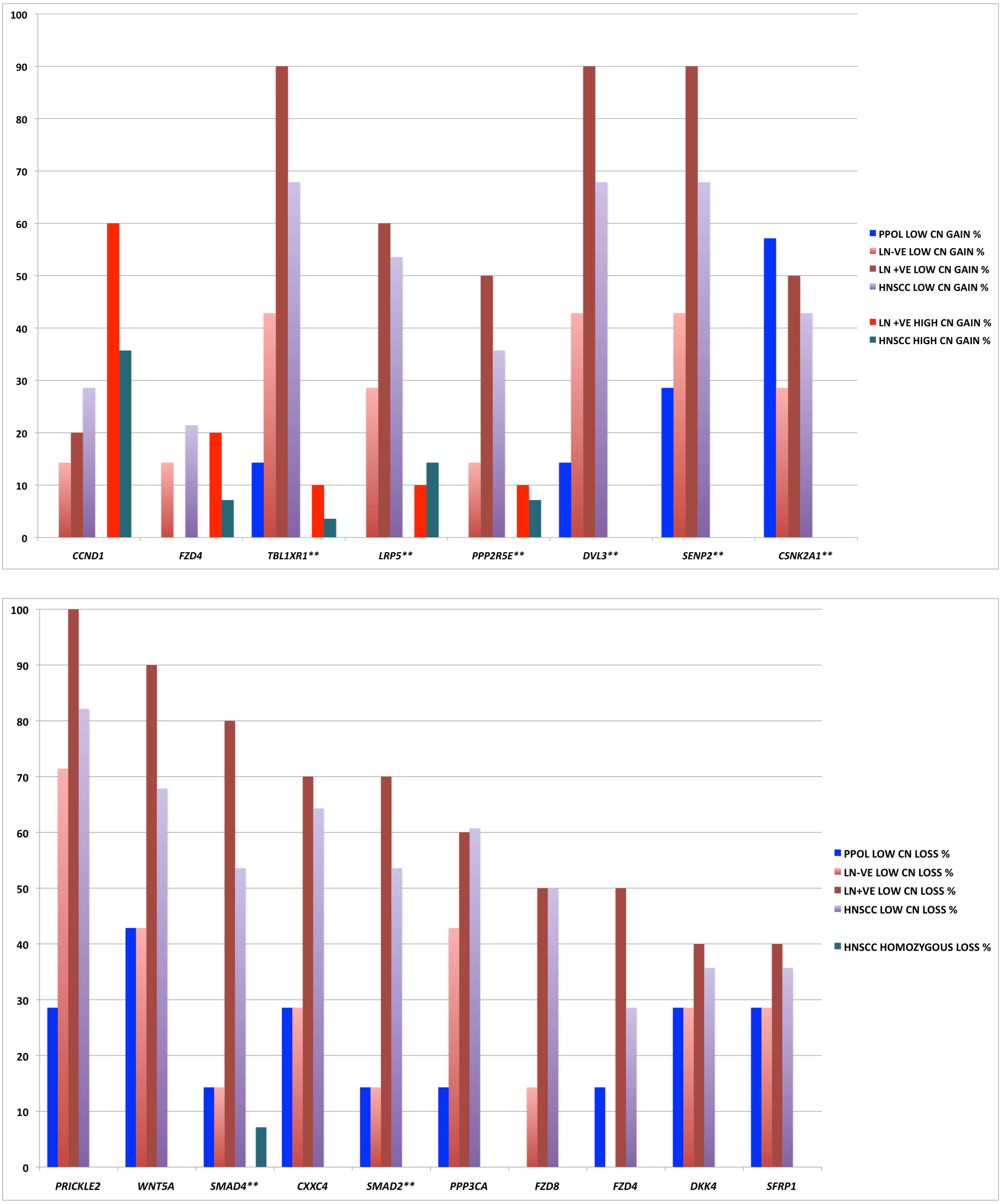

Figure 10. TGFB Pathway

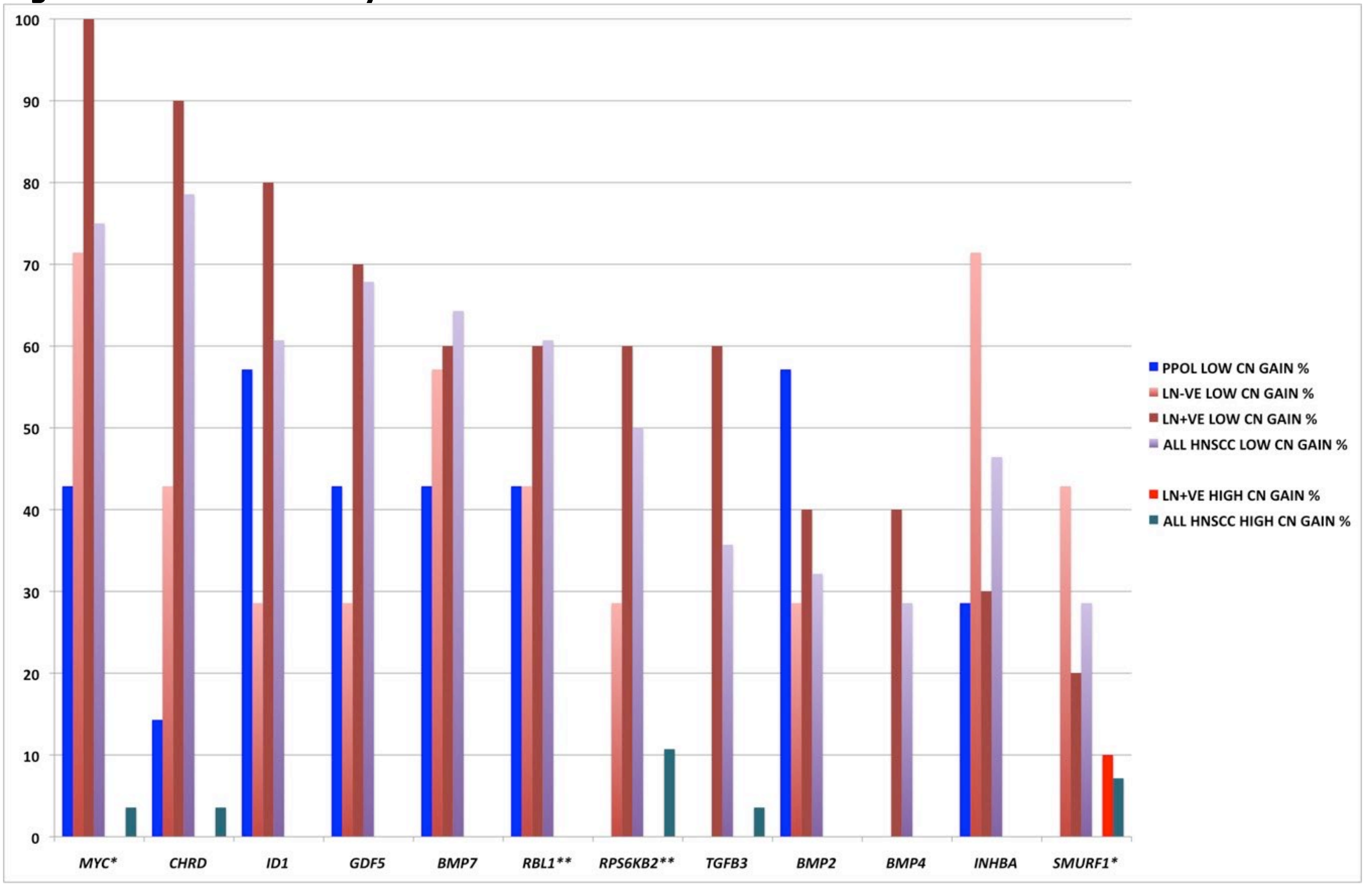

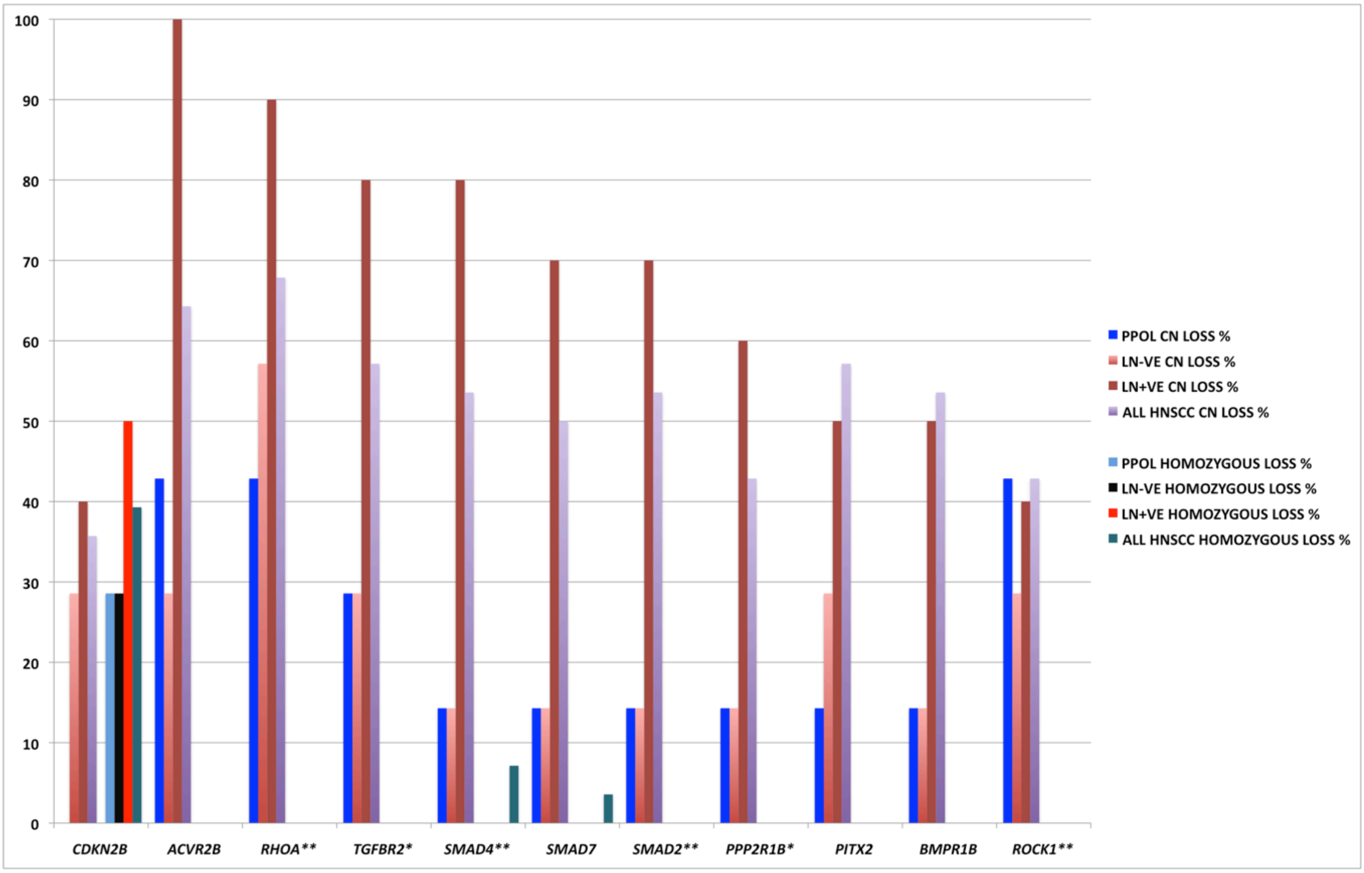

Supplement: Supplementary file 2 — Supplementary Data S5 [file 41598_2019_48229_MOESM2_ESM.pdf]
